# Stereoselective synthesis of macrocyclic peptides *via* a dual olefin metathesis and ethenolysis approach†
†Electronic supplementary information (ESI) available. See DOI: 10.1039/c5sc01507c



**DOI:** 10.1039/c5sc01507c

**Published:** 2015-05-21

**Authors:** Shane L. Mangold, Robert H. Grubbs

**Affiliations:** a Arnold and Mabel Beckman Laboratories of Chemical Synthesis , Division of Chemistry and Chemical Engineering , California Institute of Technology , Pasadena , California 91125 , USA . Email: rhg@caltech.edu ; Fax: +1-626-564-9297

## Abstract

Macrocyclic compounds occupy an important chemical space between small molecules and biologics and are prevalent in many natural products and pharmaceuticals. The growing interest in macrocycles has been fueled, in part, by the design of novel synthetic methods to these compounds. One appealing strategy is ring-closing metathesis (RCM) that seeks to construct macrocycles from acyclic diene precursors using defined transition-metal alkylidene catalysts. Despite its broad utility, RCM generally gives rise to a mixture of *E*- and *Z*-olefin isomers that can hinder efforts for the large-scale production and isolation of such complex molecules. To address this issue, we aimed to develop methods that can selectively enrich macrocycles in *E*- or *Z*-olefin isomers using an RCM/ethenolysis strategy. The utility of this methodology was demonstrated in the stereoselective formation of macrocyclic peptides, a class of compounds that have gained prominence as therapeutics in drug discovery. Herein, we report an assessment of various factors that promote catalyst-directed RCM and ethenolysis on a variety of peptide substrates by varying the olefin type, peptide sequence, and placement of the olefin in macrocycle formation. These methods allow for control over olefin geometry in peptides, facilitating their isolation and characterization. The studies outlined in this report seek to expand the scope of stereoselective olefin metathesis in general RCM.

## Abstract 2

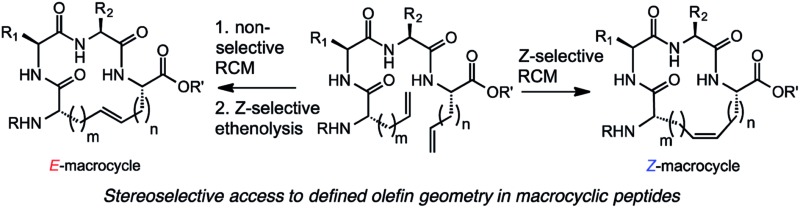
A metathesis strategy for controlling olefin geometry within macrocyclic peptides has been achieved using catalyst-directed RCM and ethenolysis.

## Introduction

**Fig. 1:**
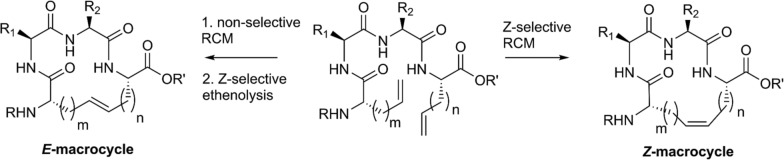
A strategy for controlling olefin geometry in macrocyclic peptides using catalyst-directed RCM and ethenolysis.

The emergence of chemical strategies for accessing macrocyclic motifs has fostered a renewed interest in their development and macrocycles now fulfill roles in diverse applications from natural products^1,2^ and therapeutics^3,4^ to platforms in supramolecular chemistry.^5^ Contemporary strategies for macrocycle formation often rely on the use of macrolactonization,^6–8^ macrolactamization,^9–11^ “click” cyclization,^12–14^ or transition-metal catalyzed reactions including olefin metathesis^15,16^ and intramolecular cross coupling.^1,17,18^ Among these strategies, ring-closing metathesis (RCM) has assumed a prominent role in macrocycle formation, in part, as a consequence of the selectivity and functional group compatibility of select olefin metathesis catalysts.^19,20^ Such chemoselectivity has offered new strategies for retrosynthetic disconnections in complex molecule synthesis and many active pharmaceuticals have been developed around the use of RCM.^21,22^ One promising application of RCM involves macrocyclization on peptides, often conferring beneficial properties to these compounds including enhanced activity^23–25^ and improved proteolytic stability.^26,27^ While RCM has found utility across many disciplines, an outstanding challenge in this transformation has been the ability to control olefin geometry in the product. Although indirect methods have been developed, including alkyne metathesis followed by partial reduction^28–31^ or substrate-controlled RCM of vinylsiloxanes followed by desilylation,^32,33^ the scope of these transformations is limited. We envisioned that a more streamlined route could be devised by modulating the equilibrium of olefin metathesis with control over both RCM and the reverse, ring-opening metathesis (ROM) using ethylene and olefin selective metathesis catalysts (Fig. 1).

We recently explored the use of *Z*-selective cyclometalated ruthenium catalysts for the derivatization of commodity chemical feedstocks using catalyst-controlled ethenolysis.^34,35^ These studies led us to consider whether *Z*-selective ethenolysis could serve as a practical tool for the purification of *E*-olefins from stereoisomeric mixtures of *E*- and *Z*-olefins in complex substrates bearing multiple functionality. Such a strategy could have value in the synthesis and isolation of natural products, peptides, and pharmaceuticals as even small amounts of stereoisomeric impurities can affect their physical or biological properties. As such, we sought to employ a dual RCM/ethenolysis strategy as a means to control olefin geometry in macrocycles. As a rigorous test of our methodology, we focused on the generation of macrocyclic peptides, a class of compounds that are traditionally difficult substrates to synthesize and isolate with defined olefin geometry.^1,15^ Herein, we provide detailed comparative studies of a variety of ruthenium catalysts in promoting RCM on peptides and assess the role of catalyst structure in controlling the stereoselectivity of RCM. Moreover, through the combined efforts of RCM and catalyst-directed ethenolysis, we offer methods for the selective formation of *E*- or *Z*-olefin geometry within macrocyclic peptides.

## Results and discussion

### Diastereoselective RCM on macrocyclic peptides bearing *i*, *i* + 2 olefin crosslinks

**Fig. 2:**
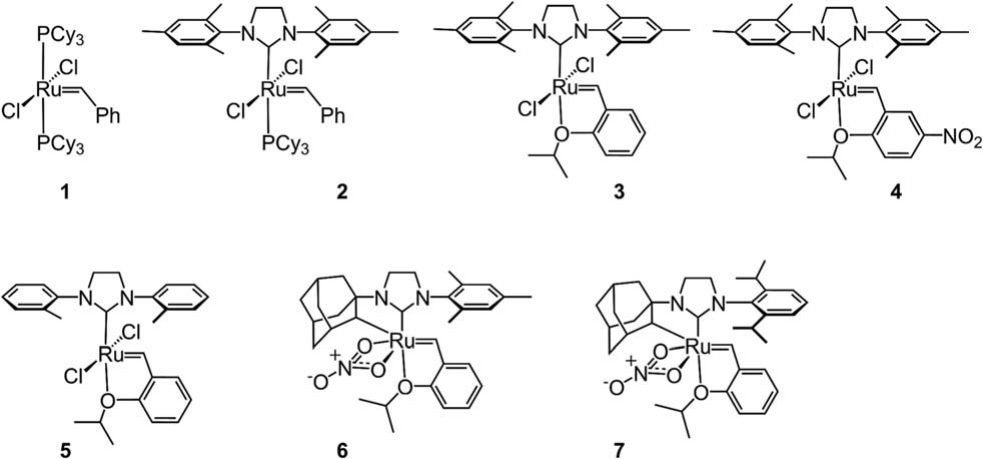
A survey of ruthenium catalysts used to promote RCM on macrocyclic peptides.

Despite the therapeutic potential of macrocyclic peptides, they represent a relatively underdeveloped class of compounds due, in part, to their complex structures and limited methods for their synthesis.^36–38^ We sought to apply RCM as a strategy to streamline the synthesis of cyclic peptides and to investigate the influence of olefin type, position, and size of the macrocycle on the efficiency and stereoselectivity of RCM. Moreover, we aimed to provide detailed comparative studies of a variety of ruthenium catalysts in promoting RCM on peptides (Fig. 2).

Our initial studies began with the optimization of RCM on dienes **8a–c** that contain olefins spanning across *i*, *i* + 2 residues using ruthenium catalysts **1–5** and *Z*-selective cyclometalated catalysts **6** and **7** (Table 1). Using this strategy, we could assess the intrinsic *E*/*Z* stereoselectivity of each catalyst in macrocycle formation and gain an understanding of the relative activity of each catalyst to promote RCM. Exposing diene **8a** to the first-generation ruthenium catalyst **1** under dilute conditions to promote macrocycle formation afforded the RCM product **9a** in 58% yield and with 80% selectivity for the *E*-olefin isomer. The use of the more active second-generation catalyst **2** afforded **9a** in 71% yield and 90% *E*-selectivity. We next examined the use of chelated isopropoxy catalysts in RCM. Exposing diene **8a** to catalyst **3** afforded macrocycle **9a** in 63% yield and with 90% *E*-selectivity. Comparable yields and diastereoselectivities were observed in the presence of the faster initiating catalyst **4**, affording **9a** in 66% yield and 82% *E*-selectivity.^39^ We also explored the use of catalysts bearing less sterically encumbering substituents around the ruthenium center (*i.e.*, tolyl catalyst **5** (ref. 40)) but conversions to **9a** were typically low, affording the product in 45% yield.^41^ The use of *Z*-selective cyclometalated catalysts **6** and **7** afforded **9a** in 47% and 41% yield, respectively. Notably, the olefin selectivity could be reversed to afford macrocycles highly enriched in the *Z*-olefin isomer.

**Table 1:** Ring-closing metathesis of peptides bearing *i*, *i* + 2 olefin crosslinks

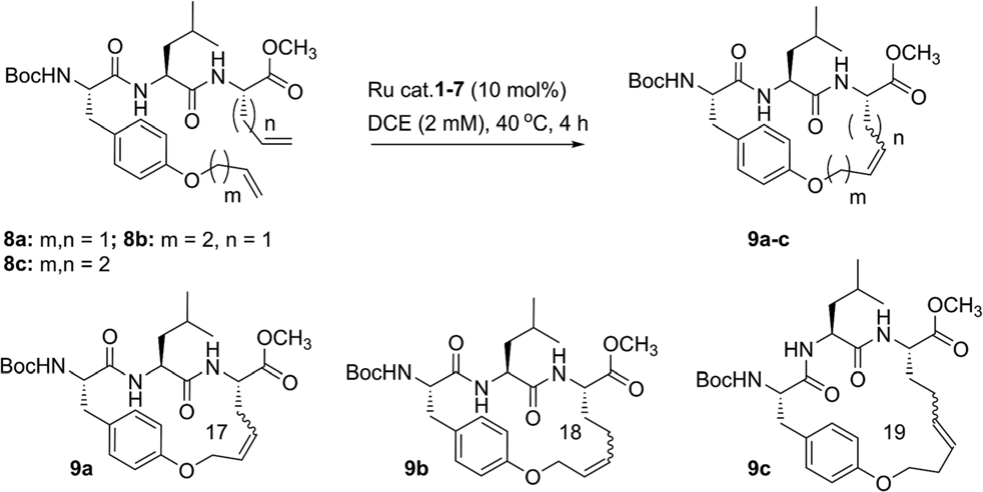						
Catalyst	Yield^*a*^ (%)			Selectivity^*b*^ (*E* : *Z*)		
	**9a**	**9b**	**9c**	**9a**	**9b**	**9c**
	*m* = 1, *n* = 1	*m* = 2, *n* = 1	*m* = 2, *n* = 2	*m* = 1, *n* = 1	*m* = 2, *n* = 1	*m* = 2, *n* = 2
1	58	54	68	81 : 19	72 : 28	66 : 34
2	71	61	77	91 : 9	88 : 12	82 : 18
3	63	55	70	90 : 10	85 : 15	83 : 17
4	66	60	66	82 : 18	85 : 15	78 : 22
5	45	24	44	81 : 19	n.d.	81 : 19
6	47	21	26	13 : 87	n.d.	15 : 85
7	41	17	24	7 : 93	n.d.	5 : 95

^*a*^Isolated yields.

^*b*^Determined by ^1^H and ^13^C NMR spectroscopy.

To probe the influence of macrocycle size on the stereoselectivity of RCM, we incorporated an additional methylene unit into one (*i.e.*, peptide **8b**) or both (**8c**) positions of the olefin-bearing amino acids. We anticipated that such modifications might influence the *E*/*Z* ratio of olefin geometry in the product due to the varying ring sizes that form upon macrocyclization.^42^ Moreover, we could determine if the identity of the olefin (*i.e.*, allylic or homoallylic) had any influence on the efficiency of RCM using catalysts **1–7**. Exposing substrate **8b** to catalysts **1–5** resulted in variable yields and *E*/*Z* ratios for the formation of macrocycle **9b**, from 54% yield and 70% diastereoselectivity for catalyst **1** to 24% yield and 80% *E*-selectivity for catalyst **5**. The cyclometalated ruthenium catalysts **6** and **7** were less active than catalysts **1–5** in RCM of **8b**, with conversions below 25% for the formation of **9b**. Interestingly, formation of the 18-membered macrocycle **9b** was consistently lower than formation of **9a**,^43^ and this finding prompted us to examine the structurally analogous peptide **8c**, bearing an additional methylene that would give rise to the 19-membered macrocycle **9c** upon RCM. In general, the conversion of peptide **8c** to macrocycle **9c** (66–77%) was improved relative to the conversion of **8b** to macrocycle **9b** (50–60%). In the presence of the first-generation catalyst **1**, the selectivity for the *E*-isomer was lower for **9c** (66%) compared to **9a** (81%) and **9b** (77%) and this trend was consistent with catalyst **2** in RCM. As with macrocycles **9a** and **9b**, catalysts **3** and **4** were more active than catalyst **5** in RCM, affording the desired macrocycle **9c** in 70% yield and 80% *E*-olefin selectivity as compared to 44% yield and 80% selectivity in the presence of catalyst **5**. Interestingly, the propensity of macrocycles **9b** and **9c** to form with greater *Z*-selectivity relative to **9a** using non-selective catalysts **1–5** did not facilitate RCM in the presence of *Z*-selective catalysts **6** and **7** as shown by the comparatively low yields of **9b** (21%) and **9c** (26%) to **9a** (41%) with **6** or **7**. This finding is consistent with previous reports suggesting that cyclometalated ruthenium catalysts, in some cases, cannot overcome any substrate bias that may favor the formation of *Z*-olefin geometry during metathesis.^44^ These studies provide evidence that subtle variations of catalyst structure and macrocycle size can greatly impact the yield and diastereoselectivity of RCM on olefin-bearing peptides. For macrocycles bearing homoallylic olefin tethers (*i.e.*, **9c**), the *E*/*Z* diastereoselectivity of RCM was generally lower than for dienes consisting of allylic olefin tethers (*i.e.*, **9a**). In this regard, macrocyclization of **9c** was improved relative to **9a**, mostly notably in the presence of phosphine-containing catalysts **1** and **2**.

### The influence of heteroatoms and peptide sequence in stereoselective RCM on peptides bearing *i*, *i* + 3 olefin crosslinks

Our studies regarding the activity of catalysts **1–7** in RCM on substrates **8a–c** suggests that the size of the macrocycle can influence *E*/*Z* diastereoselectivity. To explore this further, we synthesized peptides bearing an additional amino acid between olefin crosslinks. This would enable access to additional cyclic structures and provide insight into the effect of varying the position of olefin-containing amino acids along the peptide in RCM. Moreover, we could investigate a larger variety of amino acids, including those bearing allylic heteroatoms in the side chain, in macrocyclic ring closure. These studies were motivated by our observation that both the peptide sequence and identity of the olefin can profoundly affect the efficiency of metathesis in homodimerization and cross metathesis on peptides and we wondered if these trends would extend to RCM.^44^

We first evaluated the influence of allylic heteroatoms in facilitating RCM and their influence on *E*/*Z* diastereoselectivity. Exposing the allyl-modified peptide **10** to our optimized reaction conditions afforded macrocycle **15** in 70% yield with 74% *E*-selectivity using catalyst **1** (Table 2). By comparison, the *O*-allyl serine (**11**) and *S*-allyl cysteine (**12**) modified peptides gave the desired macrocycles **16** and **17** in 73% and 77% yield and with 92% and 90% *E*-selectivity, respectively.^45^ These trends were observed in the presence of the second-generation ruthenium catalyst **2**; in this instance, the formation of macrocycle **15** could be obtained in 74% yield, as compared to macrocycles **16** (76%) and **17** (78%) in RCM. We next exposed peptides **10–12** to catalysts **3** and **4**. The *O*-allyl modified peptide **11** was converted to macrocycle **16** in 73% yield, compared to 61% yield for the conversion of allyl peptide **10** to **15** using catalyst **3**. Similar yields and selectivities were observed with catalyst **4**, whereby peptide **11** afforded slightly higher yields of the RCM product **16** (74%) relative to the conversion of **10** to **15** (64%). We next exposed the allyl cysteine-modified peptide **12** to RCM. The formation of macrocycle **17** was generally improved relative to **15** or **16**, occurring in 80% yield and with 90% *E*-selectivity using catalysts **1–3**. As observed with substrates **8a–c**, the use of tolyl catalyst **5** under the RCM conditions led to lower conversions to macrocycles **15–17** (39–44%). By comparison, the use of *Z*-selective catalyst **6** and **7** in RCM resulted in yields ranging from 32–40% for formation of **15–17** but with reversal of olefin selectivity. Unlike with the use of isopropoxy catalysts **3** and **4** in RCM, an absence of a pronounced heteroatom effect was observed with cyclometalated catalysts **6** and **7** that may be attributable to their comparatively lower reactivity in RCM. These studies imply that the identity of the olefin can influence the efficiency and diastereoselectivity of RCM.

**Table 2:** Ring-closing metathesis on peptides bearing *i*, *i* + 3 olefin crosslinks

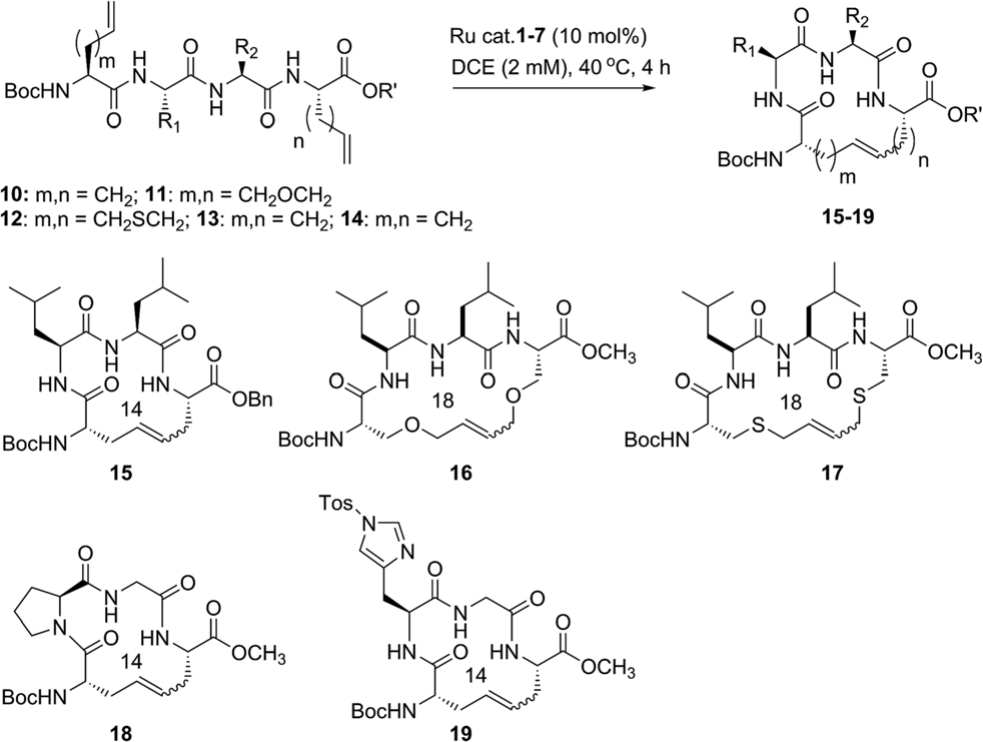										
Catalyst	Yield^*a*^ (%)					Selectivity^*b*^ (*E* : *Z*)				
	**15**	**16**	**17**	**18**	**19**	**15**	**16**	**17**	**18**	**19**
1	70	73	77	37	14	74 : 27	92 : 8	90 : 10	77 : 23	80 : 20
2	74	76	81	48	18	90 : 10	93 : 7	92 : 8	91 : 9	89 : 11
3	61	73	79	41	<10	86 : 14	90 : 10	90 : 10	80 : 20	n.d.
4	64	74	83	36	12	82 : 18	81 : 19	85 : 15	82 : 18	n.d.
5	39	44	42	20	<5	85 : 15	90 : 10	91 : 9	81 : 19	n.d.
6	32	36	40	17	<5	12 : 88	16 : 84	13 : 87	n.d.	n.d.
7	30	34	33	18	<5	10 : 90	9 : 91	5 : 95	n.d.	n.d.

^*a*^Isolated yield.

^*b*^Determined by ^1^H and ^13^C NMR spectroscopy.

We next examined peptides of varying amino acid sequence in RCM. Our previous work regarding the activity of catalysts **6** and **7** in homodimerization and cross metathesis revealed that a subset of olefin-bearing amino acids had a deactivating effect on olefin metathesis.^44^ Specifically glycine, proline, and histidine were shown to be unreactive in homodimerization and cross metathesis. We set out to discover if such amino acids generally inhibit metathesis by using a broader range of catalysts and whether incorporation of these amino acids within a larger peptide could override their apparent inactivity. To test this, we generated peptide **13** containing the amino acids proline and glycine at positions along the peptide proximal to the olefin-bearing amino acids. In the presence of catalysts **6** and **7**, conversions of **13** to **18** were less than 20%. For comparison, we examined catalysts **1–5** in RCM on diene **13**. Yields to the corresponding macrocycle **18** were variable, ranging from 20% in the presence of catalyst **5** to 48% with catalyst **2**. For those catalysts that could achieve reasonable yields of **22**, the selectivity was above 80% in favor of the *E*-olefin isomer. As a further test, we synthesized peptide **14** bearing histidine in place of proline and evaluated its activity in RCM. Conversions of **14** to macrocycle **19** were consistently below 25% in the presence of catalysts **1–7**. Under these conditions, formation of the 14-membered ring may be hindered by the proximity of histidine,^46^ which has been shown to have a deactivating effect on metathesis activity.^47^ Taken together, such results point to the importance of olefin identity, peptide sequence, and catalyst structure in controlling both the efficiency and diastereoselectivity of RCM on macrocyclic peptides. For those peptides bearing *i*, *i* + 3 olefin crosslinks, incorporation of allylic heteroatoms into the amino acid side-chain generally favored RCM, most notably in the presence of isopropoxy catalysts **3** and **4** and to a lesser extent with phosphine containing catalysts **1** and **2** and cyclometalated ruthenium catalysts **6** and **7**. These observations reflect the importance of directly comparing various catalyst structures in RCM and seek to guide further strategies for optimizing olefin metathesis on peptide-containing substrates.

### *Z*-Selective ethenolysis for the enrichment of macrocyclic peptides in *E*-olefin geometry

Encouraged by the success of RCM on a variety of peptide substrates, we next evaluated strategies to transform macrocyclic peptides having a mixture of olefin isomers into those bearing a single olefin isomer. Such a strategy could facilitate the isolation and characterization of olefin-containing macrocycles and offer a means to more easily investigate the influence of olefin geometry on the stability, activity, and conformation of this important class of compounds.

**Fig. 3:**
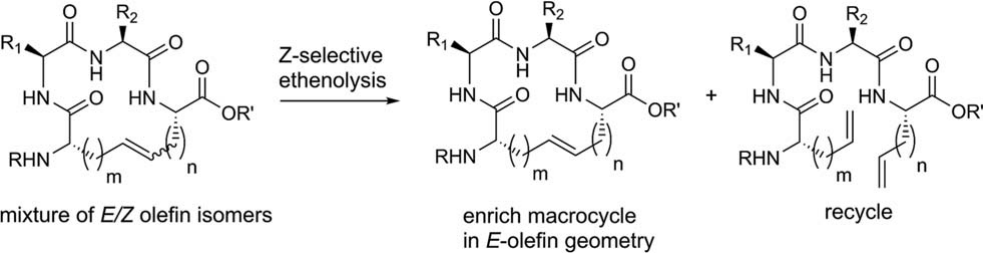
Catalyst-controlled ethenolysis as a strategy to selectively enrich olefin geometry within macrocyclic peptides.

We envisioned an olefin enrichment strategy using a catalyst-controlled ethenolysis pathway (Fig. 3). This approach capitalizes on the inherent reversibility of olefin metathesis by using ethylene to drive ring-opening metathesis.^48,49^ By having a catalyst that is selective for the formation of one olefin isomer (*e.g.*, catalysts **6** and **7**) it should be possible to selectivity degrade olefin isomers from the corresponding mixtures. We imagined that this strategy could serve as a valuable tool to form cyclic peptides having a single olefin isomer which, to date, has been a synthetic challenge using olefin metathesis.

For our initial studies, we examined catalyst **6** in *Z*-selective ethenolysis using macrocycles bearing *i*, *i* + 2 or *i*, *i* + 3 crosslinks and having variable ratios of olefin isomers. In this way, we could determine if the *E*/*Z* ratio in macrocyclic peptides affect the efficiency of ethenolysis. Under our optimized ethenolysis conditions, nearly complete *Z*-degradation of substrate **9a** occurred in the presence of ethylene (1 atm) and catalyst **6** (5 mol%), affording enrichment of **9a** in the *E*-olefin isomer in greater than 98% (entry 1, Table 3).^50^ More significantly, the ethenolysis conditions were able to transform macrocycle **9b** from a 80 : 20 mixture of isomers to those bearing almost exclusive formation of the *E*-isomer (entry 2). To test the generality of the method, these conditions were applied to macrocyclic peptides **9c** and **15–18**. Complete consumption of the *Z*-isomer was observed for **9c** and **15–17**, affording the pure *E* macrocycles with enrichment above 98% (entries 3–6). A notable exception was compound **18** that resulted in comparatively low enrichment (entry 7).^51^ From these studies, the efficiency of *Z*-selective ethenolysis does not appear to be greatly influenced by the initial *E*/*Z* ratio of olefins in macrocycles **9** and **15–18**. For those peptides that underwent efficient ethenolysis, the resulting starting material could be recovered and resubjected to the RCM conditions, providing a method for increasing the overall yield of product through iterative RCM/ethenolysis metathesis events. These results suggest that catalyst-controlled ethenolysis can serve as a practical method for the selective formation of *E*-olefins in macrocyclic peptides. This strategy, when coupled to *Z*-selective RCM can afford macrocycles predominantly enriched in *E* or *Z* olefin geometry.

**Table 3:** Catalyst-controlled ethenolysis for the enrichment of olefin geometry in macrocyclic peptides

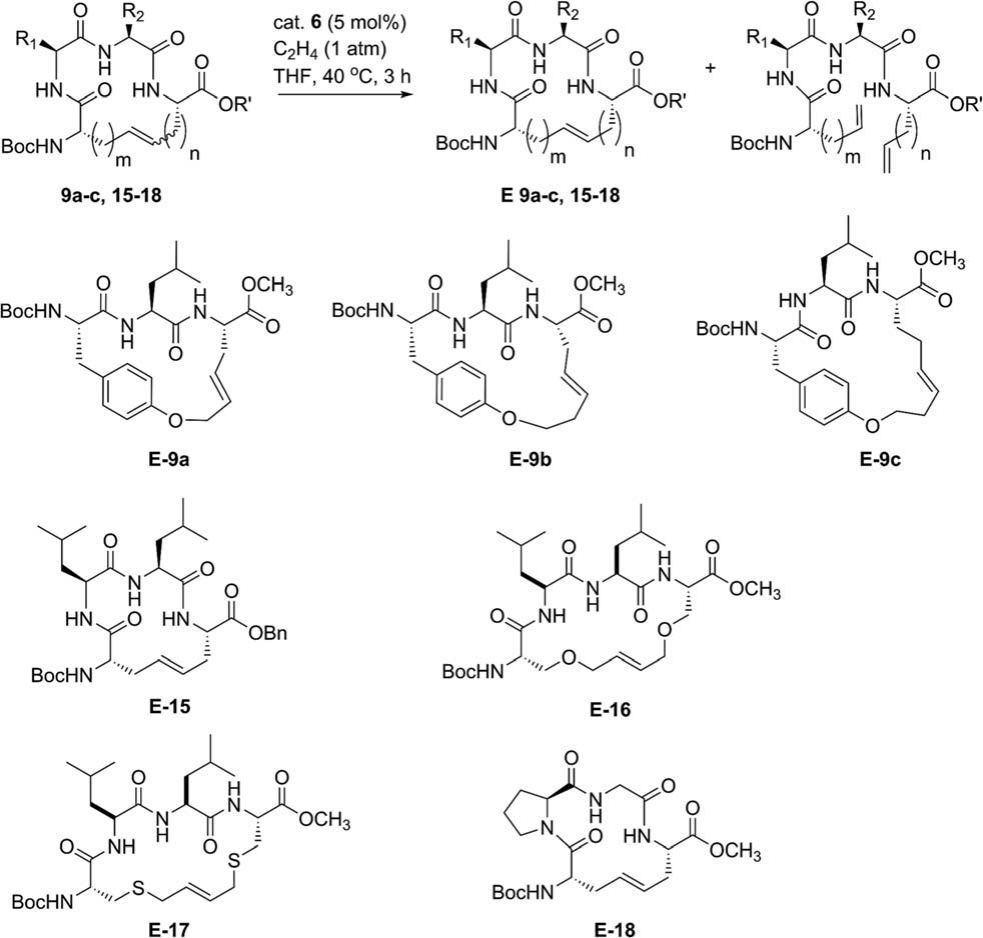				
Entry	Compound	Initial *E* : *Z* ^*a*^	Final *E* : *Z* ^*a*^	Yield^*b*^
1	**9a**	96 : 4	99 : 1	62
2	**9b**	80 : 20	97 : 3	74
3	**9c**	82 : 18	96 : 4	80
4	**15**	90 : 10	>99 : 1	77
5	**16**	81 : 19	98 : 2	64
6	**17**	90 : 10	99 : 1	67
7	**18**	77 : 23	88 : 12	45

^*a*^Determined by ^1^H, ^13^C NMR spectroscopy and analytical HPLC/MS.

^*b*^Isolated yield.

### RCM of resin-bound α-helical peptides bearing *i*, *i* + 4 and *i*, *i* + 7 crosslinks

The investigation of macrocyclic ring closure on peptides containing *i*, *i* + 2 or *i*, *i* + 3 olefinic crosslinks revealed that the peptide sequence and olefin identity can influence the efficiency and diastereoselectivity of RCM. Moreover, macrocycles consisting of a mixture of olefin isomers could be enriched in *E*-olefin geometry using *Z*-selective ethenolysis and that the initial *E*/*Z* ratio did not appear to influence the efficiency of ethenolysis. We sought to further expand these studies to α-helical peptides bearing *i*, *i* + 4 or *i*, *i* + 7 olefinic tethers. Such compounds, often referred to as stapled peptides,^52–54^ have gained attention as potential therapeutics in a variety of areas including cancer, infectious disease, and metabolism.^25–27,55^

To date, most strategies for macrocyclization on α-helical peptides rely on RCM and subsequent hydrogenation to generate a fully saturated hydrocarbon tether along the peptide helix.^55,56^ In this sense, little attention has been focused on examining the role of olefin geometry on the conformation or biological activity of macrocyclic peptides. We wanted to explore if the RCM/ethenolysis reaction manifold could provide a method to synthesize stapled peptides with defined olefin geometry. Moreover, we sought to extend the methodology to peptides on resin for enabling a more streamlined and high-throughput method of peptide synthesis, identification, and purification. In choosing the peptide sequences, we were attracted to those having a variety of amino acids and olefin crosslinks and our studies began with the optimization of RCM on peptides bearing *i*, *i* + 4 crosslinks that afford a 21-membered macrocycle (Table 4). We first evaluated RCM on resin-bound peptide **20**,^55^ using the first-generation catalyst **1**. After extensive optimization, conversions to the desired cyclic peptide **26** could be achieved in 94% conversion and with 66% *E*-selectivity. By comparison, the second-generation catalyst **2** afforded **26** in 97% conversion and 80% *E*-selectivity under the same reaction conditions. Exposing **20** to catalysts **3** and **4** led to conversions of 84% and 88%, respectively. As observed with other olefin-containing peptides, the tolyl catalyst **5** was less active in RCM, with 75% conversion of **20** to **26**. Applying the cyclometalated catalysts **6** and **7** to the RCM conditions afforded **26** in 83% and 81% conversion, respectively. In these instances, the selectivity was in favor of the *Z*-olefin isomer. We also examined peptides containing the amino acids proline^44^ and histidine^57^ that were shown to reduce the efficiency of RCM on peptides bearing *i*, *i* + 3 olefin crosslinks (*i.e.*, peptides **13** and **14**). We reasoned that incorporating olefin tethers at the *i*, *i* + 4 positions might facilitate RCM on-resin by serving to preorganize the reactive side chains on the same face of the α-helix. Such preorganization of the olefins, in addition to expanding the size of the macrocycle, might favor RCM over competing deactivation by amino acid side chains. To test this, we synthesized peptides containing proline and histidine at positions distal from the olefin crosslinks (*i.e.*, peptide **21**) or proximal to the crosslinks (**22**) and examined their activity in RCM. Exposing peptide **21** to catalysts **1** and **2** led to nearly full conversion (90%) of **21** to macrocycle **27**. The use of catalysts **3** and **4** in RCM of **21** resulted in slightly lower conversion (80%) and with 75% *E*-selectivity. Exposing **21** to catalyst **5** afforded macrocycle **27** in 70% conversion, comparable to that of catalysts **6** and **7** (75%). To probe further the role of amino acid sequence in RCM of peptides bearing *i*, *i* + 4 crosslinks, we exposed the histidine-containing peptide **22** to similar reaction conditions. In the presence of catalysts **1–4** conversions to macrocycle **28** ranged from 80% with catalyst **4** to 90% in the presence of catalyst **1**. For these cases, the diastereoselectivity of macrocycle formation ranged from 62% in the presence of **1** to 80% *E*-selectivity in the presence of catalysts **2–4**. A slight decrease in conversion to **28** was seen in the presence of catalysts **6** and **7** but with reversal of olefin selectivity. These results imply that the efficiencies of RCM on resin-bound peptides **26–28** are comparable, even for wide variation in peptide sequence.

**Table 4:** RCM on peptides bearing *i*, *i* + 4 and *i*, *i* + 7 olefin crosslinks

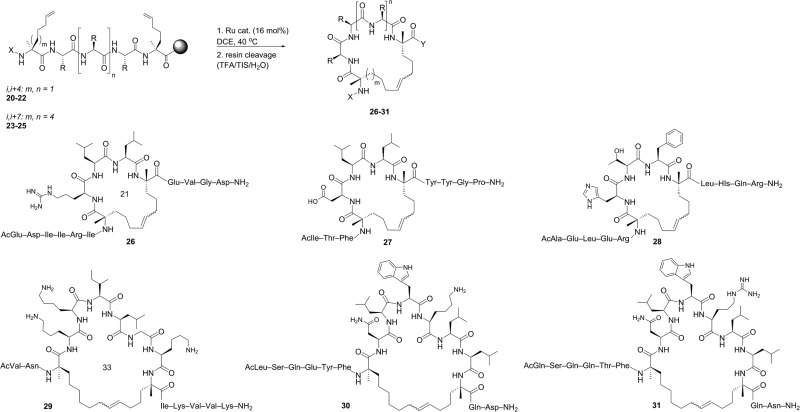												
Catalyst	Conversion^*a*^ (%)						Selectivity^*a*^ (*E* : *Z*)					
	**26**	**27**	**28**	**29**	**30**	**31**	**26**	**27**	**28**	**29**	**30**	**31**
	*m*, *n* = 1	*m*, *n* = 1	*m*, *n* = 1	*m*, *n* = 4	*m*, *n* = 4	*m*, *n* = 4	*m*, *n* = 1	*m*, *n* = 1	*m*, *n* = 1	*m*, *n* = 4	*m*, *n* = 4	*m*, *n* = 4
1	94	90	93	88	96	92	66 : 23	64 : 36	70 : 30	58 : 42	62 : 38	65 : 35
2	97	90	85	94	95	90	80 : 20	75 : 25	83 : 17	80 : 20	75 : 25	79 : 21
3	84	81	88	80	84	85	80 : 20	75 : 25	74 : 26	78 : 22	78 : 22	80 : 20
4	88	80	92	85	88	85	72 : 28	74 : 26	72 : 28	71 : 29	81 : 19	81 : 19
5	76	70	70	60	84	80	66 : 33	70 : 30	74 : 26	n.d.	71 : 29	74 : 26
6	83	75	80	70	80	75	20 : 80	22 : 78	18 : 82	20 : 80	21 : 79	17 : 83
7	81	75	70	55	75	70	17 : 83	19 : 81	23 : 77	n.d.	18 : 82	20 : 80

^*a*^Determined by analytical HPLC-MS.

We next evaluated α-helical peptides bearing *i*, *i* + 7 olefin crosslinks for the formation of 33-membered macrocycles, as our goal was to compare the influence of macrocycle size on olefin diastereoselectivity in RCM for resin-bound peptides. For our initial studies, we monitored the conversion of peptide **23** to macrocycle **29** (ref. 24) using the first- and second-generation ruthenium catalysts **1** and **2**. The conversion to macrocycle **29** was achieved in 88% in the presence of **1** and 94% with the use of catalyst **2**, respectively. Under these conditions, the selectivity of the *E*-olefin was only 58% in the presence of **1** but increased to 80% in the presence of catalyst **2**. The use of isopropoxy catalysts **3** and **4** afforded **29** in 3 : 1 *E* : *Z* selectivity at 80% conversion, trends that were similar to peptides bearing *i*, *i* + 4 crosslinks. By comparison, the conversions were typically lower in the presence of catalyst **5** (80%), **6** (83%) and **7** (81%). As observed in the formation of macrocycles bearing *i*, *i* + 2 or *i*, *i* + 4 crosslinks, the ability to form macrocycles with greater *Z*-olefin content using non-selective catalysts **1–5** did not facilitate RCM in the presence of *Z*-selective catalysts **6** and **7**. As further validation, we examined the use of RCM for formation of macrocycles **30** and **31**. Conversions of **24** (ref. 58) to **30** reached a maximum of 95% with catalyst **1** with slightly lower conversions in the presence of **2** (90%), **3** (84%) or **4** (86%). In these cases, the selectivity ranged from 60% with **1** to 80% in favor of the *E*-isomer with the use of catalyst **4**. These trends were observed in the formation of macrocycle **31** using catalysts **1–4**, with conversions greater than 85% and comparable diastereoselectivity. The use of catalysts **5–7** in macrocyclization of **25** (ref. 44) afforded the desired cyclic peptide **31** with slightly lower conversions (60–70%) relative to the formation of macrocycle **30** (75–80%). These comparative studies suggest that increasing the macrocycle size and/or preorganizing the olefins on the same face of the α-helix may facilitate RCM even in the presence of amino acids that normally reduce the efficiency of olefin metathesis. Interestingly, such trends seem be consistent in the presence of phosphine-containing catalysts **1** and **2** or isopropoxy catalysts **3–7**.

### *Z*-Selective ethenolysis on resin-bound α-helical peptides

Our results regarding RCM on a variety of olefin-bearing peptides revealed that the diastereoselectivity of macrocyclic ring closure was dictated both by the choice of catalyst and size of the macrocycle. In cases involving peptides bearing *i*, *i* + 2 or *i*, *i* + 3 olefin crosslinks, RCM generally favored the formation of the *E*-isomer (∼80% *E*) in the presence of catalysts **1–5**. Alternatively, the use of cyclometalated catalyst **6** or **7** gave rise to macrocycles predominantly of *Z*-olefin geometry, but at lower yields or conversions as expected for substrates where the *E*-isomer is normally favored. For macrocycles **8** and **15–18**, *Z*-selective ethenolysis provided a method for further enrichment of *E*-olefin geometry. While these studies provide a framework for enabling the formation of *E* or *Z* olefins in cyclic peptides, we sought to extend our studies of ethenolysis to resin-bound peptides. Such experiments would prove particularly useful as the diastereoselectivity of RCM to form macrocycles **26–31** was typically low. In this sense, the ability to selectively perform ethenolysis on these macrocycles could streamline methods for their identification and purification.

Our initial studies began with *Z*-selective ethenolysis on macrocycle **26** (Table 5). Conversion of **26** to the olefin-enriched macrocycle ***E*-26** occurred in 86%, transforming **26** from an initial ratio of 72% *E*-olefin to greater than 90% *E* (entry 1). This trend was observed for the selective ethenolysis of macrocycle **27** which occurred in 65% conversion and transformed an 80% mixture of *E*/*Z* isomers of **27** to a macrocycle having greater than 90% selectivity for the *E*-olefin (entry 2). To probe the general utility of the method, we exposed macrocycles **28–31** containing *i*, *i* + 7 crosslinks to the ethenolysis conditions (entries 3–6). Conversions to the enriched macrocycles varied from 78% for **29** to 93% for the formation of **31**. As with macrocycles **26** and **27**, enrichment of **28–31** to the *E*-olefin could occur in greater than 90%. These studies, in parallel with ethenolysis on macrocycles **8** and **15–18**, point to the utility of RCM and ethenolysis as a practical means of olefin enrichment in cyclic peptides.

**Table 5:** *Z*-Selective ethenolysis on stapled peptides bearing *i*, *i* + 4 and *i*, *i* + 7 crosslinks

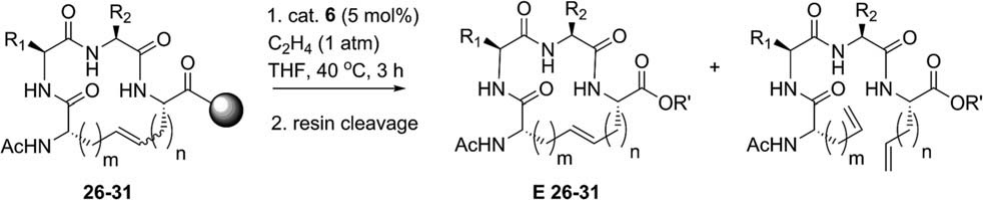				
Entry	Compound	Initial *E* : *Z* ^*a*^	Final *E* : *Z* ^*a*^	Conversion^*a*^%
1	**26**	72 : 28	95 : 5	86%
2	**27**	83 : 17	93 : 7	65%
3	**28**	71 : 29	96 : 4	91%
4	**29**	79 : 21	94 : 6	78%
5	**30**	64 : 36	98 : 2	83%
6	**31**	81 : 19	98 : 2	93%

^*a*^Determined by analytical HPLC/MS of cleaved peptide.

### Assessing the role of olefin geometry on the conformation of α-helical peptides

Our studies of RCM in tandem with catalyst-directed ethenolysis provided access to macrocyclic peptides enriched in *E*- or *Z*-olefin isomers. We sought to examine if changes in olefin geometry induced measureable differences in the overall fold or conformation of macrocyclic peptides. For our initial studies, we examined the α-helical content between non-stapled peptide **21** and the corresponding *E* or *Z* macrocycle **27** using circular dichroism (Table 6). The linear peptide **21** was measured to have an α-helical content of 21% (entry 1) which increased upon macrocyclization to **27** affording an α-helicity of 80% for the *E*-olefin and 71% for the *Z*-olefin, respectively (entries 2 and 3). These results are in agreement with the observation that macrocyclization by RCM generally induces greater α-helicity within stapled peptides.^25,26,59,60^ To further expand our studies, we next examined the role of olefin-geometry on the α-helical content within a larger macrocycle and chose peptide **23** containing olefins at *i*, *i* + 7 positions. For this peptide, the helical content was 7.5% for the non-cyclized peptide (entry 4) but upon macrocyclization to **29**, the α-helicity increased to 21% and 23% for the *E*- and *Z*-olefin isomers, respectively (entries 5 and 6). As observed with macrocycle **27**, the difference in the helicity between the *Z*- and *E*-olefin isomers in **29** was minimal, suggesting that the olefin geometry in **27** and **29** does not contribute substantially to the overall secondary structure of the macrocycles bearing olefin tethers of such lengths. These studies seek to inform further explorations into examining the role of olefin geometry on the stability or biological activity of macrocyclic compounds.

**Table 6:** Assessment of olefin geometry on α-helicity of linear peptides **21** and **23** and corresponding macrocycles **27** and **29**

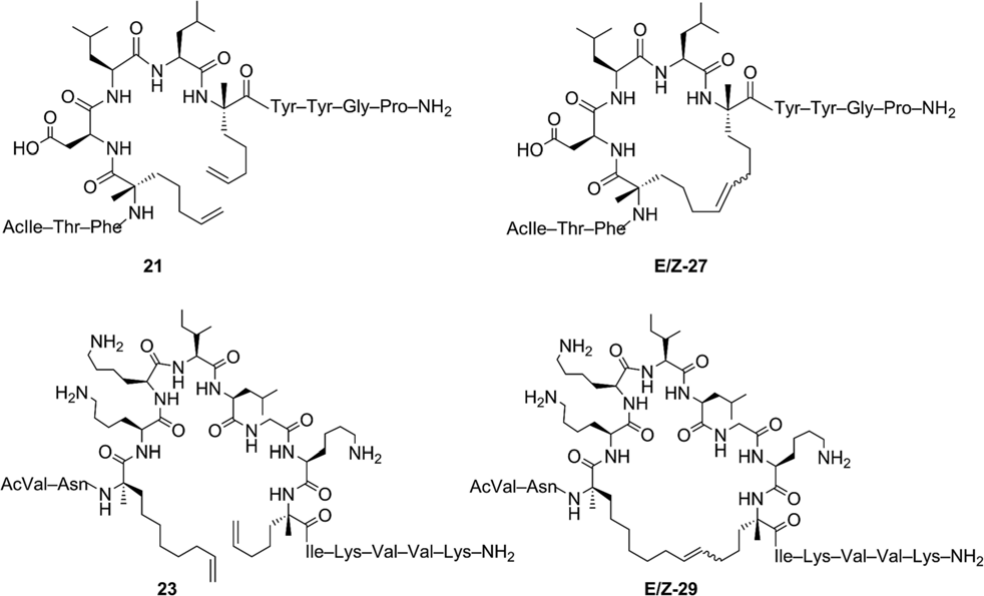		
Entry	Compound	% α-helicity^*a*^
1	**21**	20.8
2	***E*-27**	80.9
3	***Z*-27**	71.0
4	**23**	7.5
5	***E*-29**	21.2
6	***Z*-29**	23.1

^*a*^Determined by circular dichroism.

## Conclusions

In summary, we report a method for the stereoselective synthesis of macrocyclic peptides using RCM in tandem with catalyst-controlled ethenolysis. The utility of the method was demonstrated on a variety of peptide sequences and olefin crosslinks to enrich macrocycles in *E* or *Z* olefin geometry. The strategies outlined herein can facilitate the synthesis and isolation of macrocylic peptides and this approach allowed for the examination of olefin geometry on the conformation of α-helical peptide secondary structures. Notably, these studies provide a comprehensive evaluation of a variety of ruthenium catalysts in facilitating RCM on peptides and highlight the use of cyclometalated ruthenium catalysts to control diastereoselectivity in macrocycle synthesis. It is envisioned that these studies will enable strategies for accessing novel macrocyclic architectures and help elucidate the role of olefin geometry on the stability or biological activity of cyclic peptides. Progress in the design of catalysts that provide such dual capabilities will continue to broaden the scope and applications of olefin metathesis in areas from chemical biology to natural product synthesis and pharmaceutical development.
