# Supplementary material for: Stereoselective synthesis of macrocyclic peptides via a dual olefin metathesis and ethenolysis approach
Source: Chem Sci. 2015 May 21;6(8):4561–9. doi: 10.1039/c5sc01507c (PMC4618480; doi:10.1039/c5sc01507c)

Electronic Supplementary Material (ESI) for Chemical Science.
This journal is © The Royal Society of Chemistry 2015

Stereoselective synthesis of macrocyclic peptides via a dual olefin metathesis and ethenolysis approach

Shane L. Mangold and Robert H. Grubbs*,†

†Arnold and Mabel Beckman Laboratories for Chemical Synthesis, Division of Chemistry and Chemical Engineering, California Institute of Technology, Pasadena, California 91125, United States and

Supporting Information

Table of Contents

**General Information**	**S3–S4**
**General procedure for the synthesis of allyl-modified amino acids**	**S5**
**Characterization data of allyl–modified amino acids**	**S4–S8**
**General procedure for homoallyl modification of amino acids**	**S8**
**Characterization data for homoallyl modification of amino acids**	**S8–S10**
**General synthesis of peptides bearing i, i+2 olefin crosslinks**	**S10**
**Characterization data for peptides bearing i, i+2 olefin crosslinks**	**S10-S12**
**General synthesis of peptides bearing i, i+3 olefin crosslinks**	**S12**
**Characterization data for peptides bearing i, i+3 olefin crosslinks**	**S12–S16**
**General procedure for RCM on peptides bearing i, i+2 and i, i+3 olefin crosslinks**	**S17**
**Characterization data for macrocyclic peptides bearing i, i+2 or i, i+3 crosslinks**	**S17–S20**
**General procedure for Z-selective ethenolysis on macrocyclic peptides 8a-c, 15-18**	**S20**
**Characterization data for ethenolysis of macrocyclic peptides 8a-c, 15-18**	**S21–S27**
**Solid-phase synthesis of olefin-containing peptides**	**S28**
**General procedure for RCM on resin-bound olefinic peptides**	**S29**
**Monitoring the conversion of RCM on resin-bound olefinic peptides**	**S29**
Conversion of peptide **21** to macrocycle **27**	S30
Conversion of peptide **22** to macrocycle **28**	S31
Conversion of peptide **23** to macrocycle **29**	S32
Conversion of peptide **25** to macrocycle **31**	S33

**General procedure for Z-selective ethenolysis on resin-bound peptides 26-31**	**S34**
**Characterization data for ethenolysis on peptides 26-31**	**S35–S40**
**MALDI-TOF characterization data of peptides**	**S41**
Macrocycle **26**	S42
Macrocycle **27**	S42
Macrocycle **28**	S43
Macrocycle **29**	S43
Macrocycle **30**	S44
Macrocycle **31**	S44
**HPLC traces of macrocyclic peptides**	**S45**
Macrocycle **27**	S45
Macrocycle **29**	S46
**Circular dichroism of α-helical peptides**	**S47**
**NMR spectra**	**S48–S99**

General Information

All reactions were carried out in dry glassware under an atmosphere of argon using standard Schlenk line techniques. Ruthenium catalysts **1-3, 5** and cyclometalated ruthenium catalysts **6** and **7** were obtained from Materia, Inc. and used as received. Catalyst 4 was synthesized as previously described.[1] All solvents were purified by passage through solvent purification columns and further degassed by bubbling argon. Ethylene gas was purchased and used as received from Matheson, and was either Ultra high Purity (99.95% or Matheson Purity (99.995%). Commercially available reagents were used as received unless otherwise noted. Solid substrates were used after purification by column chromatography (SiO_2; (230-400 mesh)). Thin-layer chromatography utilized EMD Sciences silica gel 60 F254 pre-cast glass plates (Cat. No. 1.05714.0001). MBHA resin were purchased from Novabiochem. All Boc-protected or Fmoc-protected amino acids were purchased from ChemImpex or synthesized as described. Fmoc-(S)-2-(4-pentenyl)alanine or Fmoc-(R)-2-(7-octenyl)alanine were synthesized as previously described[2] and confirmed by spectroscopic analysis (NMR). HBTU (N,N,N',N'-tetramethyl-O-(1H-benzotriazol-1-yl)uranium hexafluorophosphate), HATU (1-[Bis(dimethylamino)methylene]-1H-1,2,3,-triazolo[4,5-b]pyridinium 3-oxid hexafluorophosphate) and HOBt (1-hydroxybenzotriazole) were purchased from NovaBioChem. N,N'-Diisopropylcarbodiimide (DIC), Piperidine, trifluoroacetic acid (TFA), triisopropylsilane (TIPS), and N,N'-dimethylformamide (DMF) were purchased from Sigma-

[1] Grela, K.; Harutyunyan, S.; Michrowska, A. *Angew. Chem. Int. Ed*. **2002**, *41*, 4038
[2] Bird, G.H.; Crannell, W.C..; Walensky, L.D. *Curr. Protoc. Chem. Biol*. **2011**, *3*, 99.

Aldrich. Triethylamine (TEA) or N,N-diisopropylethylamine (DIEA) were purchased from Sigma-Aldrich and distilled prior to use.

Standard NMR spectroscopy experiments were conducted on a Varian INOVA 500 (^{1}H: 500 MHz, ^{13}C: 125 MHz) or Varian INOVA 300 (^{1}H: 300 MHz, ^{13}C: 75 MHz) spectrometer. NMR spectra are reported as δ values in ppm relative to the reported solvent ($CDCl_3$ referenced to 7.27, CD_3OD referenced to 3.31). Splitting patterns are abbreviated as follows: singlet (s), doublet (d), triplet (t), quartet (q), multiplet (m), broad (b), apparent (app), and combinations thereof. Spectra were analyzed and processed using MestReNova.

High-resolution mass spectra (HRMS) data was obtained on a JEOL JMS-600H high resolution mass spectrometer operating in FAB^+ or positive-ion ESI mode. MALDI-TOF spectra were recorded on a Voyager DE-PRO MALDI TOF-MS spectrometer (Applied Biosystems) operating in reflector ion mode using α-cyano-4-hydroxycinnamic acid as the matrix.

Analytical HPLC was performed on an Agilent 1200 Series TOF with an Agilent G1978A Multimode source in electrospray ionization (ESI), or mixed (MM) ionization mode equipped with an Eclipse Plus C_8 column (1.8 μm, 2.1 x 50 mm). Preparative HPLC was performed with an Agilent 1100 Series HPLC utilizing an Agilent Eclipse XDB-C_{18} column (5 μm, 9.4 x 250 mm) or an Agilent Zorbax RX-SIL column (5 μm, 9.4 x 250 mm) using a gradient of double distilled water and HPLC grade acetonitrile containing 0.1% TFA or 0.1% acetic acid (AcOH). LCMS was performed on an Agilent 1200 Series LCMS equipped with a Quadrupole 6120 MS detector and an Eclipse XDB-C_{18} reverse-phase column (5, 4.6 μm x 150 mm).

Circular Dichroism (CD) spectroscopy was acquired on a CD spectrophotometer (Aviv Biomedical, Inc., Model 430) at 20 ^{o}C. The spectra were collected using a 0.1 cm pathlength quartz cuvette with the following measurement parameters: wavelength 190-255 nm; step resolution 1.0 nm, averaging time 1 sec. All peptide samples were dissolved in deionized water to a final concentration of 40 μM and filtered through a 0.22 μm syringe filter (Pall Life Sceinces). The helical content of each peptide was calculated as previously reported.[3]

[3] Forood, B.; Feliciano, E.J.; Nambiar, K.P. *Proc. Natl. Acad. Sci.* **1993**, *90*, 838

Synthesis of Allyl-Modified Amino Acids

Methyl (S)-2-((tert-butoxycarbonyl)amino)pent-4-enoate (S1)

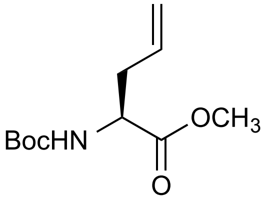

S1: $C_{11}H_{19}NO_4$
Exact Mass: 229.1314

The Boc-protected allyl glycine **S1** was synthesized using a two-step procedure starting from allyl glycine. Briefly, to a stirring suspension of (S)-allyl glycine (4.0 g, 34.6 mmol) in CH_2Cl_2 (50 mL) was added triethylamine (TEA, 3.8 mL, 52.0 mmol, 1.5 eq.) under Ar(g). The solution was cooled to 0 °C by immersion in an ice bath. Di-*tert*-butyl dicarbonate (11.2 g, 52.0 mmol, 1.5 eq.) was dissolved in CH_2Cl_2 (20 mL) and added dropwise to the stirring solution. The reaction was removed from the ice bath and allowed to stir at room temperature for 12 h. The crude mixtures was diluted with H_2O (20 mL) and extracted with 1 M HCl (3 x 20 mL), brine (3 x 20 mL), and dried over Na_2SO_4. The solvent was removed *in vacuo* to afford a light yellow oil which was carried on to the next step without further purification.

To the oil was added acetone (40 mL) and solid K_2CO_3 (9.6 g, 69.2 mmol, 2 eq.) at rt. The reaction was stirred for 10 min, followed by the addition of iodomethane (4.4 mL, 69.2 mmol, 2 eq.) and the mixture stirred for 12 h. The solvent was evaporated and the residue taken up in EtOAc (50 mL) and washed with saturated $Na_2S_2O_3$ (2 x 50 mL), brine (2 x 50 mL), and dried over Na_2SO_4. The solvent was removed *in vacuo* and the crude residue was purified by flash chromatography (3:1 Hex:EtOAc) to afford 6.2 g (78%) of **S1** as a colorless oil. ^{1}H NMR (500 MHz, $CDCl_3$) δ 5.64 (ddt, *J* = 16.5, 10.7, 7.2 Hz, 1H), 5.15–4.99 (m, 3H), 4.39– 4.25 (m, 1H), 3.68 (s, 3H), 2.56–2.35 (m, 2H), 1.39 (s, 9H); ^{13}C NMR (126 MHz, $CDCl_3$) δ 172.47, 155.13, 132.29, 118.95, 79.76, 52.86, 52.14, 36.69, 28.22 (3C). HRMS (ESI) m/z calcd for $C_{11}H_{19}NO_4$ $[M+H]^+$: 230.1386, found 230.1391

Methyl O-allyl-N-(tert-butoxycarbonyl)-L-serine (S2)

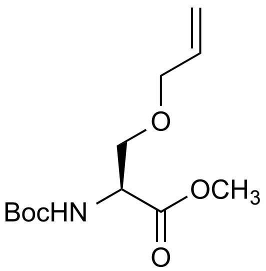

S2: $C_{12}H_{21}NO_5$
Exact Mass: 259.1420

A solution of Boc-Ser-OMe (4.0 g, 18.2 mmol) in anhydrous THF (80 mL) was degassed and treated with allylmethyl carbonate (2.8 mL, 25.4 mmol, 1.4 eq). Tetrakis(triphenylphosphine)palladium (0.42 g, 0.36 mmol, 0.02 eq.) was added and the reaction mixture heated to 60 °C for 4 h upon which TLC (2:1 EtOAc:hexanes) indicated loss of starting material. The solvent was removed under reduced pressure and the residue was diluted with EtOAc (60 mL) and washed with $NaHCO_3$ (2 x 60 mL) and brine (60 mL). The organic layer was dried over $MgSO_4$, filtered, and concentrated under reduced pressure. The residue was purified by column chromatography (SiO_2; 0% to 66% EtOAc in hexane) to afford 3.2 g (68%) of the product **S2** as a clear oil. ^{1}H NMR (500 MHz, $CDCl_3$) δ 5.79 (ddt, *J* = 17.3, 10.4, 5.6 Hz, 1H), 5.41–5.31 (m, 1H), 5.25–5.10 (m, 2H), 4.40-4.37 (m, 1H), 3.95-3.92 (m, 2H), 3.80 (dd, *J* = 9.5, 3.3 Hz, 1H), 3.71 (s, 3H), 3.61 (dd, *J* = 9.5, 3.4 Hz, 1H), 1.41 (s, 9H); ^{13}C NMR (126 MHz, $CDCl_3$) δ 171.11, 155.42, 134.01, 117.29, 79.85, 72.14, 69.86, 53.92, 52.37, 28.25 (3C). HRMS (ESI) m/z calcd for $C_{12}H_{21}N_5O_5$ $[M+H]^+$: 260.1492, found 260.1492

Methyl S-allyl-N-(tert-butoxycarbonyl)-L-cysteine (S3)

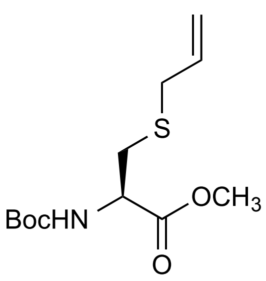

S3: $C_{12}H_{21}NO_4S$
Exact Mass: 275.1191

Following the procedure for **S2**, the allyl-protected cysteine **S3** was obtained when Boc-Cys-OMe (3.6 g, 15.2 mmol) was treated with allylmethyl carbonate (2.4 mL, 21.4 mmol, 1.4 eq.) and tetrakis(triphenylphosphine)palladium (0.34 g, 0.30 mmol, 0.02 eq.) in THF (30 mL). The residue was purified by column chromatography (SiO_2; 0% to 25% EtOAc in hexane) to afford 2.8 g (69%) of the product **S3** as a clear oil. ^1H NMR (500 MHz, $CDCl_3$) δ 5.70 (ddt, *J* = 16.9, 9.6, 7.2 Hz, 1H), 5.38–5.29 (m, 1H), 5.12–5.04 (m, 2H), 4.48-4.46 (m, 1H), 3.72 (s, 3H), 3.13–3.03 (m, 2H), 2.88 (dd, *J* = 13.9, 5.0 Hz, 1H), 2.80 (dd, *J* = 13.9, 5.7 Hz, 1H), 1.41 (s, 9H); ^{13}C NMR (126 MHz, $CDCl_3$) δ 171.55, 155.06, 133.62, 117.78, 80.00, 53.10, 52.45, 35.07, 32.76, 28.25 (3C). HRMS (ESI) m/z calcd for $C_{12}H_{21}NO_4S$ $[M+H]^+$: 276.1263, found 276.1269

Benzyl (S)-2-((tert-butoxycarbonyl)amino)pent-4-enoate (S4)

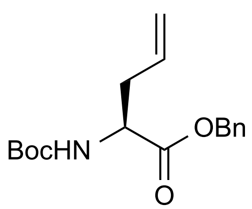

S4: $C_{17}H_{23}NO_4$
Exact Mass: 305.1627

A flask was charged with Boc-allylglycine (5.0 g, 23.2 mmol) and K_2CO_3 (4.8 g, 34.9 mmol, 1.5 eq.) under Ar(g). To this was added DMF (20 mL) and the solution stirred at rt for 10 min. Benzyl bromide (4.1 mL, 34.9 mmol, 1.5 eq.) was added dropwise to the stirring suspension. The reaction was heated to 50 °C and stirred for 8 h upon which TLC (5:1 Hexanes:EtOAc) indicated loss of starting material. H_2O (20 mL) was added, followed by EtOAc (100 mL) and the organic layer separated. The aqueous layer was extracted with EtOAc (2 x 100 mL) and the combined organic layers washed with brine (4 x 100 mL) and dried over Na_2SO_4, filtered and concentrated *in vacuo*. The crude residue was purified by column chromatography (SiO_2; 0% to 20% EtOAc in hexane) to afford 6.1 g (86%) of **S4** as a clear oil. ^1H NMR (500 MHz, $CDCl_3$) δ 7.43–7.35 (m, 5H), 5.69 (ddt, *J* = 16.1, 10.8, 7.2 Hz, 1H), 5.26–5.09 (m, 5H), 4.47 (app. q, *J* = 6.1 Hz, 1H), 2.63–2.51 (m, 2H), 1.49 (s, 9H). ^{13}C NMR (126 MHz, $CDCl_3$) δ 171.93, 155.19, 135.36, 132.18, 128.58 (2C), 128.42, 128.33 (2C), 119.21, 79.90, 67.06, 52.96, 36.73, 28.30 (3C). HRMS (ESI) m/z calcd for $C_{17}H_{23}NO_4$ $[M+H]^+$: 306.1699, found 306.1709

Methyl O-allyl-N-(tert-butoxycarbonyl)-L-tyrosine (S5)

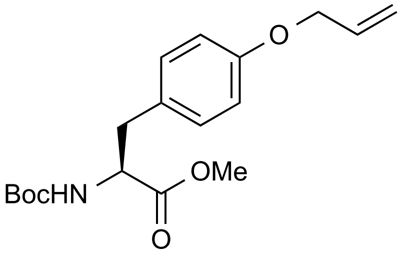

S5: $C_{18}H_{25}NO_5$
Exact Mass: 335.1733

In a typical procedure, a flask was charged with Boc-Tyr-OMe (5.0 g, 16.9 mmol) and K_2CO_3 (3.5 g, 25.4 mmol, 1.5 eq.) under Ar(g). To this was added DMF (20 mL) and the solution stirred at rt for 10 min. Allyl bromide (2.2 mL, 25.4 mmol, 1.5 eq.) was added dropwise to the stirring suspension. The reaction was heated to 50 °C and stirred for 8 h upon which TLC (3:1 Hexanes:EtOAc) indicated loss of starting material. H_2O (20 mL) was added, followed by EtOAc (100 mL) and the organic layer separated. The

aqueous layer was extracted with EtOAc (2 x 100 mL) and the combined organic layers washed with brine (4 x 100 mL) and dried over Na_2SO_4, filtered and concentrated *in vacuo*. The crude residue was purified by column chromatography (SiO_2; 0% to 25% EtOAc in hexanes) to afford 4.1 g (73%) of the product **S5** as a white solid. ^1H NMR (500 MHz, $CDCl_3$) δ 7.06 (d, *J* = 8.4 Hz, 2H), 6.89–6.86 (m, 2H), 6.12–6.04 (m, 1H), 5.46–5.42 (m, 1H), 5.33–5.30 (m, 1H), 5.03 (d, *J* = 7.5 Hz, 1H), 4.59–4.53 (m, 3H), 3.74 (s, 3H), 3.06 (m, 2H), 1.46 (s, 9H). ^{13}C NMR (126 MHz, $CDCl_3$) δ 172.41, 157.66, 155.08, 133.27, 130.25 (3C), 128.12, 117.62, 114.76, 79.84, 68.76, 54.52, 52.17, 37.45, 28.30 (3C). HRMS (ESI) m/z calcd for $C_{18}H_{25}NO_5$ $[M+H]^+$: 336.1805, found 336.1818

O-allyl-N-(tert-butoxycarbonyl)-L-serine (S6)

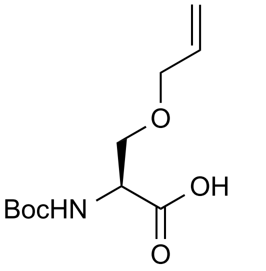

S6: $C_{11}H_{19}NO_5$
Exact Mass: 245.1263

Boc-Ser-OH (3.1 g, 15.1 mmol) was dissolved in DMF (40 mL) and cooled to 0 °C in an ice bath. NaH (1.51 g, 37.8 mmol, 2.5 eq.) was added portionwise and the reaction allowed to stir at 0 °C for 30 min. Allyl bromide (1.3 mL, 15.1 mmol, 1.0 eq) was added dropwise and the reaction allowed to warm to rt and stirred for 12 h. The reaction mixture was quenched with H_2O (15 mL) and the solvent removed *in vacuo*. The crude mixture was portioned between H_2O (20 mL) and EtOAc (20 mL). The organic layer was removed and the aqueous layer was acidified to pH 2 with 1 N HCl and extracted with EtOAc (30 mL). The combined organic layers were washed with brine (1 x 30 mL), dried over $MgSO_4$, filtered, and concentrated to dryness to afford the viscous oil **S6** (2.3 g, 61%) which was found to be of sufficient purity to be used in subsequent reactions. ^1H NMR (500 MHz, $CDCl_3$) δ 10.77 (s, 1H), 5.89 (ddt, *J* = 16.2, 10.9, 5.6 Hz, 1H), 5.46 (d, *J* = 8.4 Hz, 1H), 5.34–5.22 (m, 2H), 4.53–4.47 (m, 1H), 4.05 (dt, *J* = 5.6, 1.2 Hz, 2H), 3.95 (dd, *J* = 9.4, 2.9 Hz, 1H), 3.72 (dd, *J* = 9.5, 3.6 Hz, 1H), 1.50 (s, 9H), ^{13}C NMR (126 MHz, $CDCl_3$) δ 175.53, 155.71, 133.86, 117.73, 80.32, 72.37, 69.59, 53.77, 28.29 (3C). HRMS (ESI) m/z calcd for $C_{11}H_{19}NO_5$ $[M+H]^+$: 246.1335, found 246.1348

S-allyl-N-(tert-butoxycarbonyl)-L-cysteine (S7)

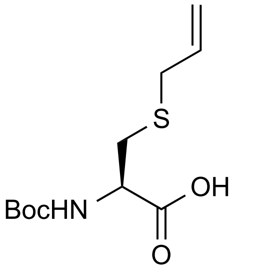

S7: $C_{11}H_{19}NO_4S$
Exact Mass: 261.1035

To a solution of compound **S3** (0.70 g, 2.4 mmol) in THF (20 mL) was added LiOH (0.14 g, 6.0 mmol, 2.5 eq.), followed by H_2O (20 mL). The reaction mixture was heated to 60 °C and stirred for 6 h upon which TLC (4:1 Hexanes:EtOAc) indicated loss of starting material. The solvent was removed *in vacuo* and partitioned between EtOAc (30 mL) and H_2O (30 mL). The organic layer was removed, and the aqueous layer was acidified to pH 2 and extracted with EtOAc (3 x 30 mL). The combined organic layers were washed with brine (1 x 30 mL) and dried over Na_2SO_4, filtered, and concentrated to dryness to afford 0.51 g (88%) of **S7** as a clear oil. The product was found to be of sufficient purity to be used in subsequent reactions. ^1H NMR (500 MHz, $CDCl_3$) δ 10.62 (s, 1H), 5.85–5.74 (m, 1H), 5.43 (d, *J* = 7.5 Hz, 1H), 5.20–5.07 (m, 2H), 4.58–4.55 (m, 1H), 3.19 (d, *J* = 7.2 Hz, 2H), 3.03–2.91 (m, 2H), 1.49 (s, 9H). ^{13}C NMR (126 MHz, $CDCl_3$) δ 175.39,

155.40, 133.58, 118.00, 77.26, 53.00, 35.19, 32.55, 28.29 (3C). HRMS (ESI) m/z calcd for $C_{11}H_{19}NO_4S$ [M+H]$^+$: 262.1107, found 262.1114

O-allyl-N-(tert-butoxycarbonyl)-L-tyrosine (S8)

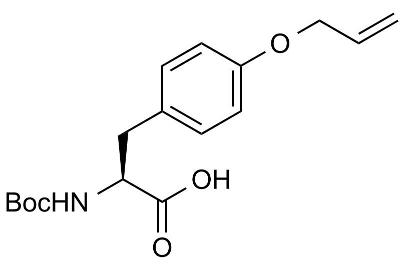

S8: $C_{17}H_{23}NO_5$
Exact Mass: 321.1576

In a typical procedure, a flask was charged with compound **S5** (1.6 g, 4.6 mmol) and to this was added THF (10 mL). An aqueous solution of 1M LiOH (11 mL, 11.4 mmol, 2.5 eq.) was added and the reaction heated to 65 °C and stirred for 7 h. The solvent was removed *in vacuo* and the solution partitioned between EtOAc (50 mL) and H_2O (50 mL). The aqueous layer was acidified to pH 2 and extracted with EtOAc (2 x 50 mL). The combined organic layers were washed with H_2O (2 x 20 mL), brine (1 x 20 mL), dried over $MgSO_4$, filtered and concentrated to dryness to afford 1.3 g (89%) of **S8** as a clear oil. The product was found to be of sufficient purity to be used in subsequent reactions. ^{1}H NMR (500 MHz, $CDCl_3$) δ 10.37 (s, 1H), 7.13 (d, J = 8.5 Hz, 2H), 6.88 (d, J = 8.6 Hz, 2H), 6.09 (ddt, J = 17.2, 10.5, 5.3 Hz, 1H), 5.22–5.13 (m, 2H), 5.01 (d, J = 7.6 Hz, 1H), 4.62–4.56 (m, 1H), 4.03 (t, J = 7.6 Hz, 2H), 3.19–2.89 (m, 2H), 2.60–2.56 (m, 2H), 1.47 (s, 9H). ^{13}C NMR (126 MHz, $CDCl_3$) δ 157.76, 155.44, 133.25, 130.36 (2C), 127.83, 117.69, 114.87 (2C), 80.33, 68.81, 54.38, 36.86, 28.29 (3C), 28.06. HRMS (ESI) m/z calcd for $C_{17}H_{23}NO_5$ [M+H]$^+$: 322.1648, found 322.1648

General procedure for homoallyl modification of amino acids

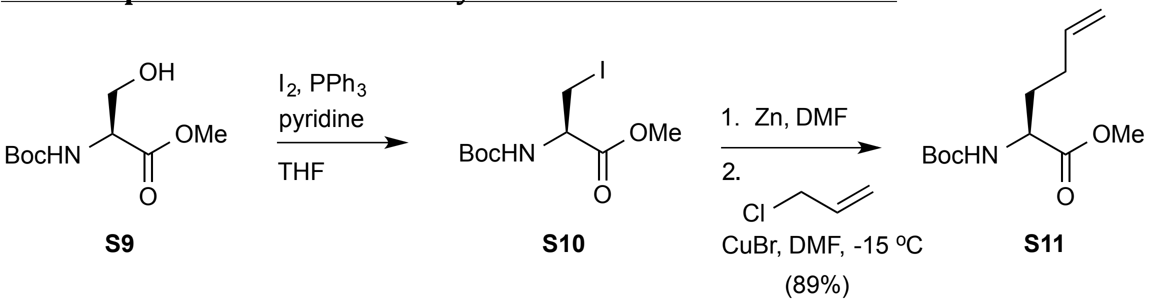

Methyl (S)-2-((tert-butoxycarbonyl)amino)hex-5-enoate (S11)

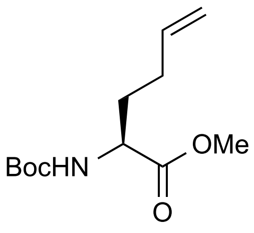

S11: $C_{13}H_{25}NO_4$
Exact Mass: 259.1784

Boc-homoallyl glycine-OMe **S11** was synthesized using a three-step protocol from commercially available Boc-Ser-OMe **S9**. In a typical procedure, a flask was charged with Boc-Ser-OMe (2.0 g, 9.1 mmol) and triphenylphosphine (3.6 g, 13.7 mmol, 1.5 eq.) under Ar(g). To this was added THF (20 mL) and the solution cooled to 0 °C by immersion in an ice bath. Pyridine (1.5 mL, 18.2 mmol, 2 eq.) was added dropwise, followed by solid iodine (3.5 g, 13.7 mmol, 1.5 eq.) in three portions at 0 °C. The ice bath was removed and stirring was continued for 4 h at rt. The mixture was extracted with Et_2O (3 x 20 mL). The combined organic layers were washed with 1M HCl (3 x 20 mL), 1M $Na_2S_2O_3$ (2 x

20 mL), brine (2 x 20 mL) and dried over Na_2SO_4**,** filtered and concentrated *in vacuo*. The crude residue was of sufficient purity to be used in the next step without further purification.

The iodopropanoate **S10** was dissolved in DMF (5 mL) and added dropwise to a flask containing activated zinc (2.4 g, 36.4 mmol, 4 eq.) at 0 °C under Ar(g). The reaction mixture was removed from the ice bath and allowed to stir at rt for 3 h, upon which TLC (4:1 petroleum ether: EtOAc) indicated loss of starting material and formation of a lower R_f spot. At this point, the reaction mixture was stopped to let the solid settle to the bottom. The supernatant was then carefully transferred by syringe to a suspension of copper(I) bromide (0.26 g, 1.8 mmol) in DMF (mL) at -15 °C that also contained allyl chloride (1.3 mL, 15.5 mmol, 1.7 eq.). After complete addition, the cooling bath was removed and stirring was continued overnight. At this point, EtOAc (20 mL) was added to the reaction mixture and stirring was continued for 15 min. To the mixture was added H_2O (20 mL), the organic layer was removed and successively washed with 1M $Na_2S_2O_3$ (2 x 20 mL), H_2O (2 x 20 mL), brine (2 x 20 mL), and dried over Na_2SO_4, filtered and concentrated *in vacuo*. The crude residue was purified by flash chromatography (SiO_2, 8:1 petroleum ether:EtOAc) to afford 2.0 g (90%) of **S11** as a colorless oil**.** ^{1}H NMR (500 MHz, $CDCl_3$) δ 5.72 (ddt, *J* = 16.9, 10.2, 6.6 Hz, 1H), 5.18–5.07 (m, 1H), 5.01–4.90 (m, 2H), 4.26-4.23 (m, 1H), 3.67 (s, 3H), 2.08–2.01 (m, 2H), 1.88–1.79 (m, 1H), 1.70–1.61 (m, 1H), 1.37 (s, 9H); ^{13}C NMR (126 MHz, $CDCl_3$) δ 173.11, 155.23, 136.87, 115.50, 79.64, 52.09, 51.99, 31.85, 29.39, 28.21 (3C). HRMS (ESI) m/z calcd for $C_{13}H_{25}NO_4$ $[M+H]^+$: 260.1856, found 260.1866

Methyl-O-homoallyl-N-(tert-butoxycarbonyl)-L-tyrosine (S12)

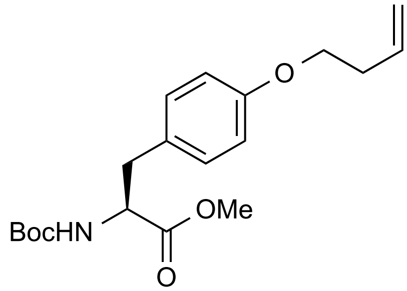

S12: $C_{19}H_{27}NO_5$
Exact Mass: 349.1889

In a typical procedure, a flask was charged with Boc-Tyr-OMe (4.0 g, 13.5 mmol) and K_2CO_3 (2.8 g, 20.2 mmol, 1.5 eq.) under Ar(g). To this was added DMF (15 mL) and the solution stirred at room temperature for 10 min. 4-bromo-1-butene (2.1 mL, 20.2 mmol, 1.5 eq.) was added dropwise to the stirring suspension. The reaction was heated to 50 °C and stirred for 8 h. H_2O (15 mL) was added, followed by EtOAc (80 mL) and the organic layer separated. The aqueous layer was extracted with EtOAc (2 x 100 mL) and the combined organic layers washed with brine (2 x 100 mL) and dried over Na_2SO_4, filtered and concentrated *in vacuo*. The crude residue was purified by column chromatography (SiO_2; 0% to 25% EtOAc in hexanes) to afford 1.8 g (38%) of the product **S12** as a white solid. ^{1}H NMR (500 MHz, $CDCl_3$) δ 7.05 (d, *J* = 8.4 Hz, 2H), 6.91–6.80 (m, 2H), 5.93 (ddt, *J* = 17.0, 10.2, 6.7 Hz, 1H), 5.24–5.12 (m, 2H), 5.02 (d, *J* = 8.0 Hz, 1H), 4.59–4.55 (m, 1H), 4.02 (t, *J* = 6.7 Hz, 2H), 3.74 (s, 3H), 3.11–3.01 (m, 2H), 2.57 (m, 2H), 1.46 (s, 9H). ^{13}C NMR (126 MHz, $CDCl_3$) δ 172.42, 157.96, 155.09, 134.43, 130.32, 130.25, 127.93, 117.00, 114.59, 79.83, 67.15, 61.27, 54.53, 52.17, 37.44, 33.64, 28.30 (3C). HRMS (ESI) m/z calcd for $C_{19}H_{27}NO_5$ $[M+H]^+$: 350.1961, found 350.1966

O-homoallyl-N-(tert-butoxycarbonyl)-L-tyrosine (S13)

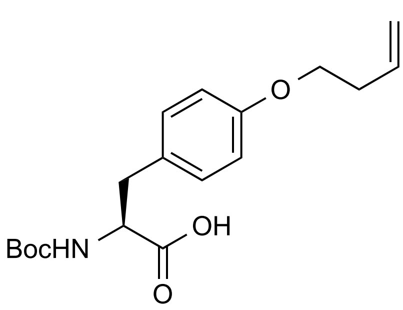

S13: $C_{18}H_{25}NO_5$
Exact Mass: 335.1733

Compound **S12** (1.6 g, 4.6 mmol) was dissolved in THF (10 mL). An aqueous solution of 1M LiOH (11 mL, 11.4 mmol, 2.5 eq.) was added and the reaction heated to 65 °C and stirred for 7 h. The solvent was removed *in vacuo* and the solution partitioned between EtOAc (50 mL) and H_2O (50 mL). The aqueous layer was acidified to pH 2 and extracted with EtOAc (2 x 50 mL). The combined organic layers were washed with H_2O (2 x 20 mL) and brine (1 x 20 mL) and dried over $MgSO_4$. The product **S13** was found to be of sufficient purity to be used in subsequent reactions. ^1H NMR (500 MHz, $CDCl_3$) δ 10.37 (s, 1H), 7.12 (d, *J* = 8.5 Hz, 2H), 6.88 (d, *J* = 8.4 Hz, 2H), 5.94 (ddt, *J* = 17.0, 10.2, 6.7 Hz, 1H), 5.22–5.13 (m, 2H), 5.01 (d, *J* = 7.7 Hz, 1H), 4.62–4.56 (m, 1H), 4.03 (t, *J* = 6.7 Hz, 2H), 3.19–2.89 (m, 2H), 2.60–2.56 (m, 2H), 1.47 (s, 9H) ^{13}C NMR (126 MHz, $CDCl_3$) δ 176.62, 158.00, 155.38, 134.43, 130.38, 130.34, 127.77, 117.01, 114.67, 80.22, 67.19, 54.37, 36.93, 33.64, 28.31 (3C). HRMS (ESI) m/z calcd for $C_{18}H_{25}NO_5$ $[M+H]^+$: 336.1805, found 335.1811

General synthesis of peptides bearing i, i+2 olefin crosslinks

N-Boc-Tyr(O-allyl)-Leu-Allylglycine methyl ester (8a)

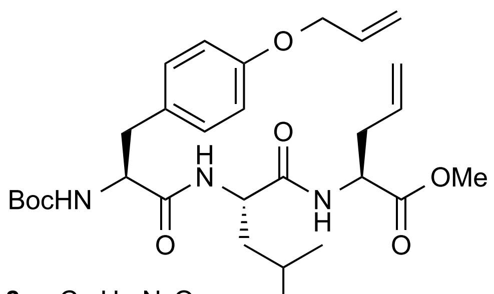

8a: $C_{29}H_{43}N_3O_7$
Exact Mass: 545.3101

Synthesis of peptide **8a** was achieved using a C-terminal to N-terminal modification strategy. Briefly, Boc-allylglycine-OMe **S1** (1.0 g, 4.4 mmol) was dissolved in CH_2Cl_2 (44 mL). To this was added TFA (10 mL, 132 mmol, 30 eq.) and the reaction stirred at rt for 2 h. The solution was concentrated *in vacuo* and the crude residue dissolved in DMF (6 mL), followed by the addition of DIEA (3.8 mL, 22 mmol, 5 eq.). The reaction mixture was stirred at rt for 10 min. A solution of Boc-Leu-OH (1.1 g, 4.8 mmol, 1.1 eq), HOBt (0.73 g, 4.8 mmol, 1.1 eq), HBTU (1.8 g, 4.8 mmol, 1.1 eq) in DMF (5 mL) and DIEA (1 mL) was added to the stirring solution and the reaction mixture allowed to stir at rt for 4 h. H_2O (20 mL) was added, followed by EtOAc (20 mL). The organic layer was separated and the aqueous layer washed with EtOAc (3 x 20 mL). The combined organic layers were washed with an aqueous solution of 1 M LiCl (3 x 20 mL) and dried over $MgSO_4$, filtered, and concentrated to dryness to afford a clear oil that was sufficiently pure (judged by NMR and LCMS) to be carried on to the next step. At this point, the crude residue was dissolved in CH_2Cl_2 (40 mL) and to this was added TFA (10 mL, 30 eq.). The reaction was stirred at rt for 2 h and concentrated *in vacuo*. The residue was dissolved in DMF (6 mL) and DIEA (3.8 mL) and allowed to stir for 10 min. A solution of Boc-protected Tyrosine **S8** (1.5 g, 4.8 mmol, 1,1 eq.), HOBt (0.73 g, 4.8 mmol, 1.1 eq.), HBTU (1.8 g, 4.8 mmol, 1.1 eq.) in DMF (5 mL) and DIEA (1 mL) was added and the reaction mixture allowed

to stir at rt for 4 h. H_2O (20 mL) was added, followed by EtOAc (20 mL). The organic layer was separated and the aqueous layer washed with EtOAc (3 x 20 mL). The combined organic layers were washed with 1 M LiCl (3 x 20 mL) and dried over $MgSO_4$, filtered, and concentrated to dryness. The crude residue was purified by column chromatography (SiO_2; 0% to 33% EtOAc in hexanes) to afford 0.11 g (45%) of product **8a** as a clear oil. ^1H NMR (500 MHz, $CDCl_3$) δ ^1H NMR (500 MHz, $CDCl_3$) δ 7.09 (d, *J* = 8.6 Hz, 2H), 6.86–6.79 (m, 2H), 6.76 (d, *J* = 7.6 Hz, 1H), 6.57 (d, *J* = 7.6 Hz, 1H), 6.03 (ddt, *J* = 17.2, 10.5, 5.3 Hz, 1H), 5.73–5.61 (m, 1H), 5.41–5.37 (m, 1H), 5.28–5.25 (m, 1H), 5.12–5.07 (m, 3H), 4.63–4.56 (m, 1H), 4.51–4.41 (m, 3H), 4.34 (bs, 1H), 3.73 (s, 3H), 3.03–2.95 (m, 2H), 2.58–2.44 (m, 2H), 1.66–1.43 (m, 3H), 1.39 (s, 9H), 0.90–0.88 (m, 6H). ^{13}C NMR (126 MHz, $CDCl_3$) δ 172.22, 171.89, 157.25, 155.60, 133.37, 132.60, 130.43, 129.29, 118.59, 117.25, 114.40, 79.19, 77.46, 68.54, 55.41, 52.06, 52.00, 51.70, 41.36, 37.76, 36.18, 28.33 (3C), 24.53, 22.72, 22.39. HRMS (ESI) m/z calcd for $C_{29}H_{43}N_3O_7$ $[M+H]^+$: 546.3173, found 546.3188

N-Boc-Tyr(O-homoallyl)-Leu-Allylglycine methyl ester (8b)

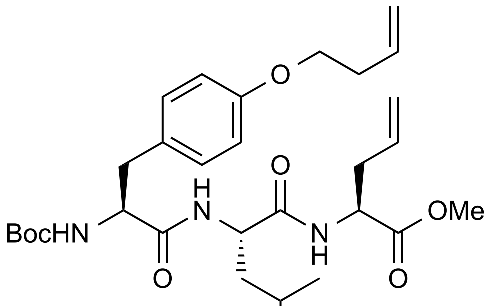

8b: $C_{30}H_{45}N_3O_7$
Exact Mass: 559.3258

Following the procedure for the synthesis of **8a**, the peptide **8b** was obtained following a C- to N-terminal modification strategy. Briefly, Boc-allylglycine-OMe **S1** (0.62 g, 2.7 mmol) was deprotected in TFA (6 mL, 30 eq.) and CH_2Cl_2 (25 mL), followed by the addition of Boc-Leu-OH (0.69 g, 3.0 mmol, 1.1 eq.) HOBt (0.46 g, 3.0 mmol), HBTU (1.1 g, 3.0 mmol), in DMF (5 mL) and DIEA (1 mL). After completion of the reaction, H_2O (20 mL) was added followed by extraction with EtOAc (3 x 20 mL) and drying with $MgSO_4$. The residue was then deprotected with TFA (6 mL) in CH_2Cl_2 (25 mL), from which a solution of Boc-homoallyl-Tyrosine **S13** (1.2 g, 3.0 mmol, 1.1 eq.) HOBt (0.46 g, 3.0 mmol), HBTU (1.1 g, 3.0 mmol) in DMF (5 mL) and DIEA (1 mL) was added and the reaction stirred for 4 h. H_2O (20 mL) was added and the crude mixture extracted with EtOAc (3 x 20 mL). The organic layers were washed with 1 M LiCl (2 x 30 mL), dried over $MgSO_4$, filtered and concentrated to dryness. The crude tripeptide was purified by column chromatography (SiO_2; 0% to 25% EtOAc in hexanes) to afford 0.88 g (58%) of the product **8b** as a white solid. ^1H NMR (500 MHz, $CDCl_3$) δ 7.12 (d, *J* = 8.5 Hz, 2H), 6.89–6.82 (m, 2H), 5.92 (ddt, *J* = 17.0, 10.2, 6.7 Hz, 1H), 5.77–5.64 (m, 1H), 5.23–5.09 (m, 5H), 4.66–4.61 (m, 1H), 4.53–4.49 (m, 1H), 4.38–4.29 (m, 1H), 4.00 (t, *J* = 6.7 Hz, 2H), 3.77–3.73 (m, 3H), 3.07–2.98 (m, 2H), 2.64–2.49 (m, 4H), 1.69–1.47 (m, 2H), 1.43 (s, 9H), 0.93–0.87 (m, 6H). ^{13}C NMR (126 MHz, $CDCl_3$) δ 171.74, 171.48, 171.33, 157.92, 157.89, 134.39, 130.30, 130.18, 128.42, 117.00, 114.72, 114.69, 114.67, 67.15, 52.32, 51.81, 51.76, 51.57, 51.39, 41.04, 40.71, 36.31, 36.23, 33.63, 28.24 (3C), 24.48, 22.87, 22.01. HRMS (ESI) m/z calcd for $C_{30}H_{45}N_3O_7$ $[M+H]^+$: 560.3330, found 560.3341

N-Boc-Tyr(O-homoallyl)-Leu-Homoallylglycine methyl ester (8c)

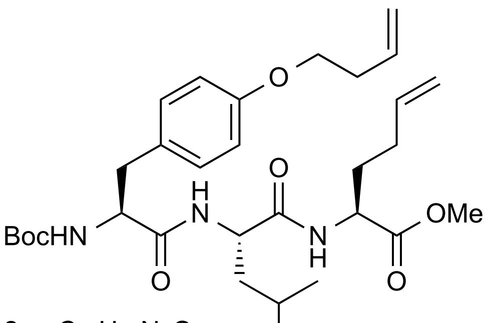

8c: $C_{31}H_{47}N_3O_7$
Exact Mass: 573.3414

Following the procedure for the synthesis of **8a**, the peptide **8c** was obtained following a C- to N-terminal modification strategy. Briefly, Boc-homoallylglycine-OMe **S11** (0.50 g, 2.1 mmol) was deprotected in TFA (5 mL, 30 eq.) and CH_2Cl_2 (21 mL), followed by the addition of Boc-Leu-OH (0.53 g, 2.3 mmol, 1.1 eq.) HOBt (0.35 g, 2.3 mmol), HBTU (0.84 g, 2.3 mmol, 1.1 eq.), in DMF (4 mL) and DIEA (0.5 mL). After completion of the reaction, H_2O (15 mL) was added followed by extraction with EtOAc (3 x 15 mL) and drying with $MgSO_4$. The residue was then deprotected with TFA (5 mL) in CH_2Cl_2 (21 mL), from which a solution of Boc-homoallyl-Tyrosine **S13** (0.77 g, 2.3 mmol, 1.1 eq.) HOBt (0.35 g, 2.3 mmol), HBTU (0.84 g, 2.3 mmol) in DMF (4 mL) and DIEA (0.5 mL) was added and the reaction stirred for 4 h. H_2O (15 mL) was added and the crude mixture extracted with EtOAc (3 x 15 mL). The organic layers were washed with 1 M LiCl (2 x 20 mL), dried over $MgSO_4$, filtered and concentrated to dryness. The crude tripeptide was purified by column chromatography (SiO_2; 0% to 33% EtOAc in hexanes) to afford 0.80 g (67%) of the product **8c** as a clear oil. ^1H NMR (500 MHz, $CDCl_3$) δ ^1H NMR (500 MHz, $CDCl_3$) δ 7.23 (d, *J* = 7.0 Hz, 1H), 7.08–7.03 (m, 2H), 6.79 –6.76 (m, 2H), 5.88 (ddt, *J* = 17.0, 10.2, 6.7 Hz, 1H), 5.75 (ddt, *J* = 16.8, 10.2, 6.6 Hz, 1H), 5.44 (d, *J* = 7.8 Hz, 1H), 5.19–4.94 (m, 4H), 4.58–4.52 (m, 2H), 4.44 (bs, 1H), 3.94 (t, *J* = 6.7 Hz, 2H), 3.73 (s, 3H), 3.42–3.35 (m, 1H), 3.01–2.91 (m, 2H), 2.53–2.49 (m, 2H), 2.38 (t, *J* = 8.1 Hz, 1H), 2.11– 1.89 (m, 4H), 1.81–1.47 (m, 4H), 1.38 (s, 9H), 0.89 (d, *J* = 6.4 Hz, 6H). ^{13}C NMR (126 MHz, $CDCl_3$) δ 175.10, 172.52, 171.74, 157.76, 155.53, 136.75, 134.40, 130.31, 128.62, 116.92, 115.86, 114.51, 79.85, 67.08, 55.62, 52.21, 51.71, 49.43, 41.15, 37.26, 33.60, 31.20, 30.64, 29.54, 28.25 (3C), 24.49, 22.68, 22.28, 17.62. HRMS (ESI) m/z calcd for $C_{31}H_{47}N_3O_7$ $[M+H]^+$: 574.3486, found 574.3499

Procedure for synthesis of alkene modified peptides bearing i, i+3 crosslinks

N-Boc-allylglycine-Leu-Leu-allylglycine benzyl ester (10)

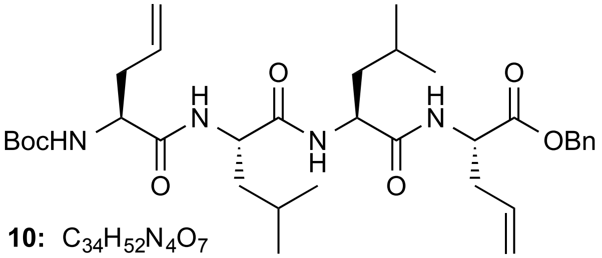

10: $C_{34}H_{52}N_4O_7$
Exact Mass: 628.3836

Synthesis of peptide **10** was achieved using a C-terminal to N-terminal modification strategy. Briefly, Boc-allylglycine-OBn **S4** (0.90 g, 2.9 mmol) was dissolved in CH_2Cl_2 (29 mL). To this was added TFA (7 mL, 88 mmol, 30 eq.) and the reaction stirred at rt for 2 h. The solution was concentrated *in vacuo* and the crude residue dissolved in DMF (5 mL), followed by the addition of DIEA (2.6 mL, 15 mmol, 5 eq.). The reaction mixture was stirred at rt for 10 min. A solution of Boc-Leu-OH (0.73 g, 3.2 mmol, 1.1 eq), HOBt (0.49 g, 3.2 mmol, 1.1 eq), HBTU (1.2 g, 3.2 mmol, 1.1 eq) in DMF (5 mL) and DIEA (1 mL) was added to the stirring solution and the

reaction mixture allowed to stir at rt for 4 h. H_2O (20 mL) was added, followed by EtOAc (15 mL). The organic layer was separated and the aqueous layer washed with EtOAc (3 x 15 mL). The combined organic layers were washed with an aqueous solution of 1 M LiCl (3 x 15 mL) and dried over $MgSO_4$, filtered, and concentrated to dryness to afford a clear oil that was sufficiently pure (judged by LCMS) to be carried on to the next step. At this point, the crude residue was dissolved in CH_2Cl_2 (30 mL) and to this was added TFA (7 mL, 30 eq.). The reaction was stirred at rt for 2 h and concentrated *in vacuo*. The residue was dissolved in DMF (5 mL) and DIEA (2.6 mL) and allowed to stir for 10 min. A solution of Boc-Leu-OH (0.73 g, 3.2 mmol, 1,1 eq.), HOBt (0.49 g, 3.2 mmol, 1.1 eq.), HBTU (1.2 g, 3.2 mmol, 1.1 eq.) in DMF (5 mL) and DIEA (1 mL) was added and the reaction mixture allowed to stir at rt for 4 h. H_2O (15 mL) was added, followed by EtOAc (15 mL). The organic layer was separated and the aqueous layer washed with EtOAc (3 x 15 mL). The combined organic layers were washed with 1 M LiCl (3 x 15 mL) and dried over $MgSO_4$, filtered, and concentrated to dryness. For the final amino acid coupling, Boc-allylglycine-OH (0.69 g, 3.2 mmol, 1.1 eq.), HOBt (0.49 g, 3.2 mmol, 1.1 eq.) HBTU (1.2 g, 3.2 mmol, 1.1 eq.) in DMF (5 mL) and DIEA (1 mL) was added to a stirring solution of the deprotected tripeptide in DMF (5 mL) and DIEA (1 mL). The reaction was stirred at rt for 4 hr. At this point, H_2O (15 mL) was added, followed by EtOAc (15 mL). The organic layer was removed and the aqueous layer extracted with EtOAc (3 x 20 mL). The combined organic layers were washed with 1 M LiCl (3 x 20 mL), brine (1 x 20 mL), and dried over $MgSO_4$, filtered, and concentrated *in vacuo*. The crude residue was purified by column chromatography (SiO_2; 0% to 20% EtOAc in hexanes) to afford 0.81 g (45%) of product **10** as a clear oil. ^1H NMR (500 MHz, $CDCl_3$) δ 7.73 (d, *J* = 7.7 Hz, 2H), 7.37–7.33 (m, 5H), 5.77–5.65 (m, 3H), 5.26–5.00 (m, 6H), 4.82–4.70 (m, 1H), 2.64–2.38 (m, 4H), 1.78–1.51 (m, 6H), 1.45 (s, 9H), 0.94–0.87 (m, 12H). ^{13}C NMR (126 MHz, $CDCl_3$) δ 171.93, 171.87, 171.56, 171.35, 155.71, 135.40, 133.12, 132.45, 128.51 (2C), 128.32 (2C), 118.66, 118.50, 79.73, 67.04, 53.64, 51.79, 51.57, 51.46, 41.88, 41.22, 37.70, 36.33, 28.35 (3C), 24.72, 24.71, 22.85, 22.59, 22.52, 22.37. HRMS (ESI) m/z calcd for $C_{34}H_{52}N_4O_7$ $[M+H]^+$: 629.3908, found 629.3986

N-Boc-allylserine-Leu-Leu-allylserine methyl ester (11)

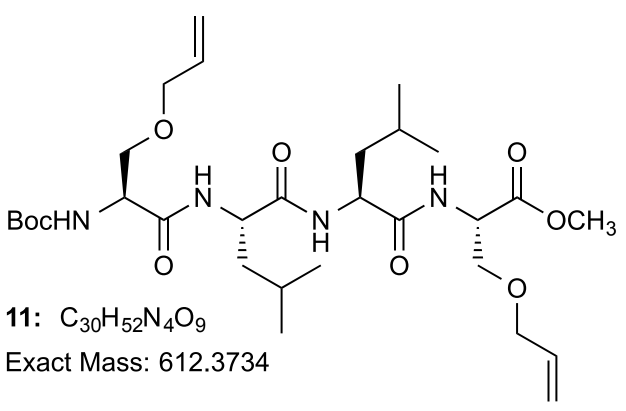

Following the procedure for the synthesis of **10**, the peptide **11** was obtained following a C- to N-terminal modification strategy. Briefly, Boc-O-allylserine-OMe **S2** (0.44 g, 1.7 mmol) was deprotected in TFA (4 mL, 30 eq.) and CH_2Cl_2 (17 mL), followed by the addition of Boc-Leu-OH (0.44 g, 1.9 mmol, 1.1 eq.) HOBt (0.29 g, 1.9 mmol), HBTU (0.72 g, 1.9 mmol, 1.1 eq.), in DMF (4 mL) and DIEA (0.5 mL). After completion of the reaction, H_2O (15 mL) was added followed by extraction with EtOAc (3 x 15 mL) and drying with $MgSO_4$. The residue was then deprotected with TFA (4 mL) in CH_2Cl_2 (17 mL), from which a solution of Boc-Leu-OH (0.44 g, 1.9 mmol, 1.1 eq.) HOBt (0.29 g, 1.9 mmol), HBTU (0.72 g, 1.9 mmol) in DMF (4 mL)

and DIEA (0.5 mL) was added and the reaction stirred for 4 h. H_2O (15 mL) was added and the crude mixture extracted with EtOAc (3 x 15 mL). The organic layers were washed with 1 M LiCl (2 x 20 mL), dried over $MgSO_4$, filtered and concentrated to dryness. The crude residue was dissolved in CH_2Cl_2 (17 mL) and TFA (4 mL) and allowed to stir for 2 h, concentrated *in vacuo* and to this was added a solution of Boc-allylserine-OH **S6** (0.46 g, 1.9 mmol, 1.1 eq.) HOBt (0.29 g, 1.9 mmol), HBTU (0.72 g, 1.9 mmol), DIEA (0.5 mL) and DMF (4 mL). After completion of the reaction as judged by LCMS, H_2O (15 mL) was added, followed by EtOAc (15 mL). The organic layer was removed and the aqueous layer extracted with EtOAc (3 x 15 mL). The combined organic layers were washed with 1 M LiCl (3 x 15 mL), dried over $MgSO_4$, filtered, and concentrated to dryness. The crude residue was purified by column chromatography (SiO_2; 0% to 25% EtOAc in CH_2Cl_2) to afford 0.43 g (42%) of product **11** as a clear, colorless oil; ^1H NMR (500 MHz, $CDCl_3$) δ 7.11 (d, *J* = 7.2 Hz, 2H), 6.99 (d, *J* = 7.1 Hz, 1H), 5.89–5.78 (m, 2H), 5.58 (d, *J* = 6.5 Hz, 1H), 5.29–5.13 (m, 4H), 4.72 (m, 1H), 4.58–4.54 (m, 1H), 4.50–4.44 (m, 1H), 4.31 (bs, 1H), 4.04–3.92 (m, 4H), 3.91–3.85 (m, 1H), 3.79–3.77 (m, 1H), 3.75 (s, 3H), 3.65–3.57 (m, 2H), 1.75–1.52 (m, 6H), 1.45 (s, 9H), 0.93–0.89 (m, 12H). ^{13}C NMR (126 MHz, $CDCl_3$) δ 172.03, 171.90, 170.72, 170.48, 155.76, 134.04, 133.85, 117.60, 117.44, 80.41, 72.20, 72.12, 69.44, 69.30, 54.46, 53.78, 52.61, 52.52, 52.23, 51.58, 40.83, 38.59, 28.23 (3C), 24.65, 23.00, 22.85, 22.78, 22.03, 21.76. HRMS (ESI) m/z calcd for $C_{30}H_{52}N_4O_9$ $[M+H]^+$: 613.3806, found 613.3815

N-Boc-allylcysteine-Leu-Leu-allylcysteine methyl ester (12)

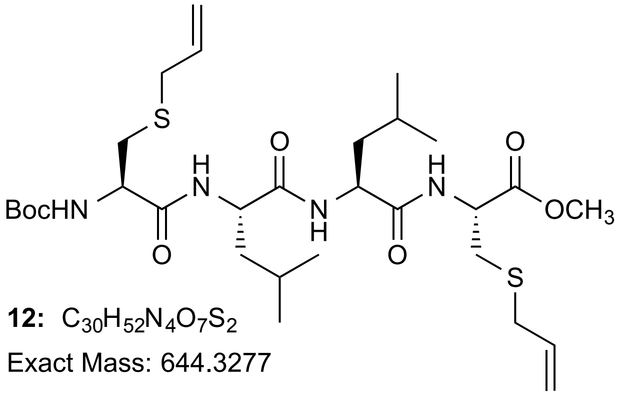

12: $C_{30}H_{52}N_4O_7S_2$
Exact Mass: 644.3277

Following the procedure for the synthesis of **10**, the peptide **12** was obtained following a C- to N-terminal modification strategy. Briefly, Boc-allylcysteine-OMe **S3** (0.40 g, 1.4 mmol) was deprotected in TFA (4 mL, 30 eq.) and CH_2Cl_2 (15 mL), followed by the addition of Boc-Leu-OH (0.35 g, 1.5 mmol, 1.1 eq.) HOBt (0.23 g, 1.5 mmol), HBTU (0.57 g, 1.5 mmol, 1.1 eq.), in DMF (4 mL) and DIEA (0.5 mL). After completion of the reaction, H_2O (15 mL) was added followed by extraction with EtOAc (3 x 15 mL) and drying with $MgSO_4$. The residue was then deprotected with TFA (4 mL) in CH_2Cl_2 (15 mL), from which a solution of Boc-Leu-OH (0.35 g, 1.5 mmol, 1.1 eq.) HOBt (0.23 g, 1.5 mmol, 1.1 eq.), HBTU (0.57 g, 1.5 mmol, 1.1 eq.) in DMF (4 mL) and DIEA (0.5 mL) was added and the reaction stirred for 4 h. H_2O (15 mL) was added and the crude mixture extracted with EtOAc (3 x 15 mL). The organic layers were washed with 1 M LiCl (2 x 20 mL), dried over $MgSO_4$, filtered and concentrated to dryness. The crude residue was dissolved in CH_2Cl_2 (15 mL) and TFA (4 mL) and allowed to stir for 2 h, concentrated *in vacuo* and to this was added a solution of Boc-S-allylcysteine-OH **S7** (0.39 g, 1.5 mmol, 1.1 eq.) HOBt (0.23 g, 1.5 mmol), HBTU (0.57 g, 1.5 mmol), DIEA (0.5 mL) and DMF (4 mL). After completion of the reaction as judged by LCMS, H_2O (15 mL) was added, followed by EtOAc (15 mL). The organic layer was removed and the aqueous layer extracted with EtOAc (3 x 15 mL). The combined organic layers were washed with 1 M LiCl (3 x 15 mL), dried over $MgSO_4$, filtered, and concentrated to dryness. The crude

residue was purified by column chromatography (SiO_2; 0% to 20% EtOAc in CH_2Cl_2) to afford 0.46 g (51%) the product **12** as a clear oil. ^1H NMR (500 MHz, $CDCl_3$) δ 7.73 (d, J = 7.6 Hz, 1H), 7.56–7.49 (m, 2H), 5.80 (d, J = 7.6 Hz, 1H), 5.76–5.64 (m, 2H), 5.27–5.04 (m, 4H), 4.85–4.58 (m, 3H), 4.52–4.51 (m, 1H), 3.72 (s, 3H), 3.11–3.02 (m, 4H), 2.92–2.88 (m, 1H), 2.85–2.68 (m, 3H), 1.75–1.46 (m, 6H), 1.40 (s, 9H), 0.89–0.84 (m, 12H). ^{13}C NMR (126 MHz, $CDCl_3$) δ 172.09, 171.56, 171.20, 170.84, 155.55, 133.81, 133.68, 117.79, 117.73, 79.95, 77.31, 54.00, 52.47, 51.95, 51.86, 51.51, 41.73, 41.30, 35.17, 34.89, 33.55, 32.20, 28.31 (3C), 24.80, 24.73, 22.85, 22.65, 22.58, 22.39. HRMS (ESI) m/z calcd for $C_{30}H_{52}N_4O_7S_2$ $[M+H]^+$: 645.3349, found 645.3356

N-Boc-allylglycine-Pro-Gly-allylglycine methyl ester (13)

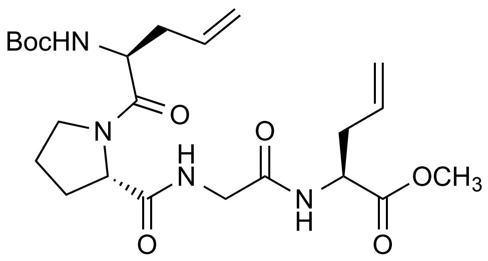

13: $C_{23}H_{36}N_4O_7$
Exact Mass: 480.2584

Following the procedure for the synthesis of **10**, the peptide **13** was obtained following a C- to N-terminal modification strategy. Briefly, Boc-allylglycine-OMe **S1** (0.50 g, 2.2 mmol) was deprotected in TFA (5 mL, 30 eq.) and CH_2Cl_2 (22 mL), followed by the addition of Boc-Gly-OH (0.42 g, 2.4 mmol, 1.1 eq.) HOBt (0.37 g, 2.4 mmol), HBTU (0.91 g, 2.4 mmol, 1.1 eq.), in DMF (5 mL) and DIEA (1.0 mL). After completion of the reaction, H_2O (20 mL) was added followed by extraction with EtOAc (3 x 20 mL) and drying with $MgSO_4$. The residue was then deprotected with TFA (5 mL) in CH_2Cl_2 (22 mL), from which a solution of Boc-Pro-OH (0.52 g, 2.4 mmol, 1.1 eq.) HOBt (0.37 g, 2.4 mmol, 1.1 eq.), HBTU (0.91 g, 2.4 mmol, 1.1 eq.) in DMF (5 mL) and DIEA (1.0 mL) was added and the reaction stirred for 4 h. H_2O (20 mL) was added and the crude mixture extracted with EtOAc (3 x 20 mL). The organic layers were washed with 1 M LiCl (2 x 20 mL), dried over $MgSO_4$, filtered and concentrated to dryness. The crude residue was dissolved in CH_2Cl_2 (22 mL) and TFA (5 mL) and allowed to stir for 2 h, concentrated *in vacuo* and to this was added a solution of Boc-allylglycine-OH (0.52 g, 2.4 mmol, 1.1 eq.) HOBt (0.37 g, 2.4 mmol), HBTU (0.91 g, 2.4 mmol), DIEA (1.0 mL) and DMF (5 mL). After completion of the reaction as judged by LCMS, H_2O (20 mL) was added, followed by EtOAc (20 mL). The organic layer was removed and the aqueous layer extracted with EtOAc (3 x 20 mL). The combined organic layers were washed with 1 M LiCl (3 x 20 mL), dried over $MgSO_4$, filtered, and concentrated to dryness. The crude residue was purified by column chromatography (SiO_2; 50% EtOAc in CH_2Cl_2 + 5% MeOH) to afford 0.39 g (37%) of the product **13** as a clear oil. ^1H NMR (500 MHz, $CDCl_3$, *cis and trans rotamers*) δ 7.53–7.35 (m, 1H), 7.31–7.28 (m, 1H), 5.81–5.68 (m, 2H), 5.35-5.33 (m, 1H), 5.19–5.07 (m, 4H), 4.62–4.44 (m, 3H), 4.13–4.08 (m, 1H), 3.94–3.75 (m, 3H), 3.71 (s, 3H), 3.66–3.63 (m, 1H), 2.61–2.34 (m, 4H), 2.24–1.95 (m, 4H), 1.42 (s, 9H). ^{13}C NMR (126 MHz, $CDCl_3$ *cis and trans rotamers*) δ 172.04, 171.89, 171.77, 171.70, 171.64, 171.49, 169.26, 169.00, 155.95, 155.28, 133.22, 132.79, 132.54, 132.30, 119.37, 118.79, 118.63, 118.51, 80.36, 79.69, 77.32, 60.88, 60.58, 52.24, 52.07, 51.56, 47.53, 47.46, 43.19, 42.99, 36.92, 36.04, 35.95, 29.04, 28.29 (3C), 28.25, 28.20, 25.19. HRMS (ESI) m/z calcd for $C_{23}H_{36}N_4O_7$ $[M+H]^+$: 481.2656, found 481.2664

N-Boc-allylglycine-His(Tos)-Gly-allylglycine methyl ester (14)

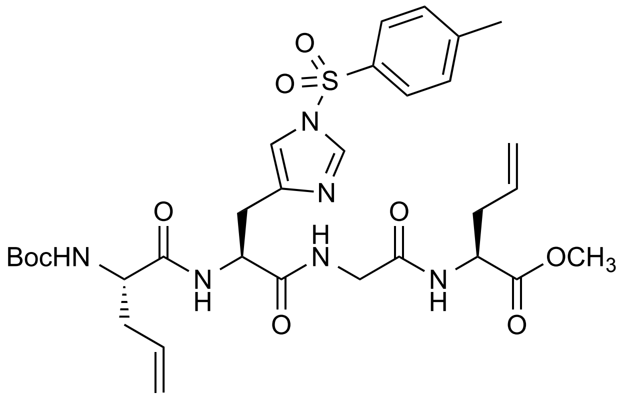

14: $C_{31}H_{42}N_6O_9S$
Exact Mass: 674.2734

Following the procedure for the synthesis of **10**, the peptide **14** was obtained following a C- to N-terminal modification strategy. Briefly, Boc-allylglycine-OMe **S1** (0.41 g, 1.8 mmol) was deprotected in TFA (4 mL, 30 eq.) and CH_2Cl_2 (18 mL), followed by the addition of Boc-Gly-OH (0.35 g, 2.0 mmol, 1.1 eq.) HOBt (0.31 g, 2.0 mmol), HBTU (0.76 g, 2.0 mmol, 1.1 eq.), in DMF (4 mL) and DIEA (0.5 mL). After completion of the reaction, H_2O (15 mL) was added followed by extraction with EtOAc (3 x 15 mL) and drying with $MgSO_4$. The residue was then deprotected with TFA (4 mL) in CH_2Cl_2 (18 mL), from which a solution of Boc-His(Tos)-OH (0.82 g, 2.0 mmol, 1.1 eq.) HOBt (0.31 g, 2.0 mmol, 1.1 eq.), HBTU (0.76 g, 2.0 mmol, 1.1 eq.) in DMF (4 mL) and DIEA (0.5 mL) was added and the reaction stirred for 4 h. H_2O (15 mL) was added and the crude mixture extracted with EtOAc (3 x 15 mL). The organic layers were washed with 1 M LiCl (2 x 15 mL), dried over $MgSO_4$, filtered and concentrated to dryness. The crude residue was dissolved in CH_2Cl_2 (18 mL) and TFA (4 mL) and allowed to stir for 2 h, concentrated *in vacuo* and to this was added a solution of Boc-allylglycine-OH (0.43 g, 2.0 mmol, 1.1 eq.) HOBt (0.31 g, 2.0 mmol), HBTU (0.76 g, 2.0 mmol), DIEA (0.50 mL) and DMF (4.0 mL). After completion of the reaction as judged by LCMS, H_2O (15 mL) was added, followed by EtOAc (15 mL). The organic layer was removed and the aqueous layer extracted with EtOAc (3 x 15 mL). The combined organic layers were washed with 1 M LiCl (3 x 15 mL), dried over $MgSO_4$, filtered, and concentrated to dryness. The crude residue was purified by column chromatography (SiO_2; 50% EtOAc in CH_2Cl_2 + 5% MeOH) to afford 0.29 g (24%) of the product **14** as a clear oil. ^1H NMR (500 MHz, $CDCl_3$) δ 8.09–7.96 (m, 1H), 7.91–7.83 (m, 2H), 7.46–7.40 (m, 2H), 7.17 (d, J = 7.5 Hz, 1H), 5.79–5.70 (m, 2H), 5.26–5.06 (m, 5H), 4.73–4.59 (m, 2H), 4.12–3.94 (m, 2H), 3.77 (s, 3H), 3.44–3.19 (m, 2H), 3.01–2.87 (m, 1H), 2.85 (s, 3H), 2.70–2.42 (m, 7H), 1.46 (s, 9H). ^{13}C NMR (126 MHz, $CDCl_3$) δ 172.34, 171.99, 171.01, 168.79, 156.48, 146.75, 140.19, 136.55, 134.60, 132.98, 132.78, 132.57, 132.52, 130.53, 130.51, 127.42, 127.40, 119.65, 115.22, 81.00, 61.73, 55.19, 53.65, 52.90, 51.97, 43.18, 38.61, 36.30, 36.09, 28.26 (3C), 21.77. HRMS (ESI) m/z calcd for $C_{31}H_{42}N_6O_9S$ $[M+H]^+$: 675.2815, found 675.2831

General procedure for RCM on peptides bearing i, i+2 and i, i+3 crosslinks

(7*S*,10*S*,13*S*)-13-((*tert*-butoxycarbonyl)amino)-10-isobutyl-9,12-dioxo-2-oxa-8,11-diaza-1(1,4)-benzenacyclotetradecaphan-4-ene-7 methyl ester (9a)

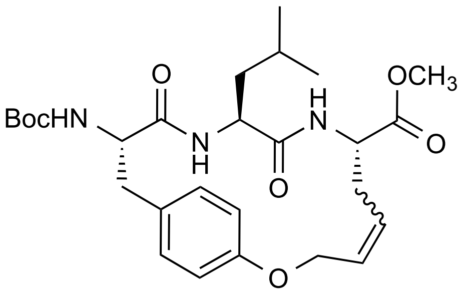

9a: $C_{27}H_{39}N_3O_7$
Exact Mass: 517.2788

In a typical procedure, a flask was charged with a solution of compound **8a** (0.10 g, 0.18 mmol) in anhydrous and degassed DCE (2 mM, 90 mL) under Ar(g). A solution of catalyst **1-7** (10 mol %) in DCE (3 mL) was added. The reaction mixture was heated at 40 ^{o}C for 4 h, allowed to cool, and quenched with an excess of ethyl vinyl ether (5 mL). The reaction was concentrated *in vacuo* and the crude residue purified by column chromatography (SiO_2; 0% to 33% EtOAc in hexane) (R_f = 0.32 in 1:1 Hexanes:EtOAc) to afford 80.0 mg (86%) of the product **9a** as a white solid. ^1H NMR (500 MHz, $CDCl_3$) δ 7.10 (bs, 2H), 6.77 (bs, 2H), 6.03 (d, *J* = 8.7 Hz, 1H), 5.91 (d, *J* = 7.3 Hz, 1H), 5.59–5.47 (m, 2H), 5.44 (d, J = 7.4 Hz, 1H), 4.80–4.75 (m, 1H), 4.69–4.59 (m, 2H), 4.27–4.20 (m, 2H), 3.78 (s, 3H), 3.12 (dd, *J* = 12.7, 4.9 Hz, 1H), 2.78–2.69 (m, 2H), 2.38–2.31 (m, 1H), 1.61–1.59 (m, 2H), 1.49 (s, 9H), 0.93–0.87 (m, 6H). ^{13}C NMR (126 MHz, $CDCl_3$) δ 171.95, 171.02, 170.69, 156.09, 155.08, 129.73 (2C), 128.48, 128.12, 127.48, 115.56 (2C), 79.85, 66.41, 57.12, 52.60, 51.81, 51.68, 42.91, 38.86, 34.88, 28.33 (3C), 24.34, 22.75, 22.49. HRMS (ESI) m/z calcd for $C_{27}H_{39}N_3O_7$ $[M+H]^+$: 518.2860, found 518.2877

(8*S*,11*S*,14*S*)-14-((*tert*-butoxycarbonyl)amino)-11-isobutyl-10,13-dioxo-2-oxa-9,12-diaza-1(1,4)-benzenacyclopentadecaphan-4-ene-8 methyl ester (9b)

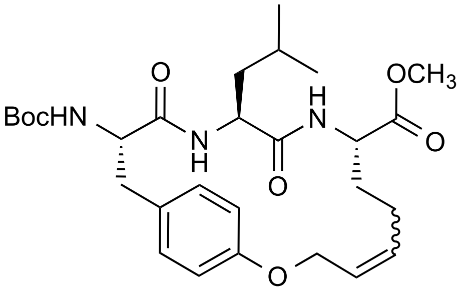

9b: $C_{28}H_{41}N_3O_7$
Exact Mass: 531.2945

Following the general procedure for **9a**, the peptide **8b** (0.10 g, 0.18 mmol) was reacted with catalysts **1-7** (10 mol%) in DCE (90 mL) to afford product **9b** (74.3 mg, 79%) as a colorless glass and as a 2:1 mixture of isomers after purification by flash chromatography (SiO_2; 1:1 DCM:EtOAc + 1 to 5% MeOH) (R_f = 0.35 in 4:1 DCM:EtOAc). ^1H NMR for *major isomer* (500 MHz, $CDCl_3$) δ 7.13 (d, *J* = 8.4 Hz, 2H), 6.89–6.76 (m, 2H), 6.07 (d, *J* = 7.4 Hz, 1H), 6.01 (d, *J* = 7.8 Hz, 1H), 5.66–5.58 (m, 2H), 5.30 (d, *J* = 8.4 Hz, 1H), 5.02–4.96 (m, 1H), 4.52–4.48 (m, 1H), 4.37–4.31 (m, 1H), 4.29–4.13 (m, 4H), 3.79 (s, 3H), 3.09–3.02 (m, 1H), 2.96–2.87 (m, 1H), 2.64–2.53 (m, 1H), 2.49–2.44 (m, 2H), 2.40–2.28 (m, 1H), 2.10–1.91 (m, 1H), 1.67–1.52 (m, 2H), 1.50 (s, 9H), 0.93–0.90 (m, 6H). ^{13}C NMR for *major isomer* (126 MHz, $CDCl_3$) δ 171.90, 170.83, 170.46, 156.72, 155.16, 130.21, 129.99 (2C), 128.95, 128.19, 126.06, 115.78 (2C), 80.04, 65.54, 56.90, 52.49, 51.65, 50.83, 42.23, 38.07, 35.50, 30.59, 28.32 (3C), 24.35, 22.77. HRMS (ESI) m/z calcd for $C_{28}H_{41}N_3O_7$ $[M+H]^+$: 532.3017, found 532.3018

(9*S*,12*S*,15*S*)-15-((*tert*-butoxycarbonyl)amino)-12-isobutyl-11,14-dioxo-2-oxa-10,13-diaza-1(1,4)-benzenacyclohexadecaphan-5-ene-9 methyl ester (9c)

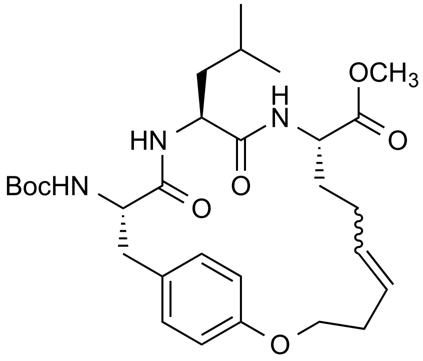

9c: $C_{29}H_{43}N_3O_7$
Exact Mass: 545.3101

Following the general procedure for **9a**, the peptide **8c** (0.10 g, 17 mmol) was reacted with catalysts **1-7** (10 mol%) in DCE (90 mL) to afford product **9c** (64.6 mg, 68%) as a colorless glass after purification by flash chromatography (SiO_2; 1:1 DCM:EtOAc + 1 to 4% MeOH) (R_f = 0.45 in 1:1 Hexanes:EtOAc). ^1H NMR (500 MHz, $CDCl_3$) δ 7.08 (d, *J* = 8.4 Hz, 2H), 6.80–6.73 (m, 2H), 6.14 (d, *J* = 7.8 Hz, 1H), 6.04 (d, *J* = 7.7 Hz, 1H), 5.56–5.46 (m, 2H), 5.29 (d, *J* = 8.4 Hz, 1H), 4.38 (td, *J* = 7.9, 5.8 Hz, 1H), 4.30–4.14 (m, 4H), 4.10–4.01 (m, 1H), 3.77 (s, 3H), 3.07–2.86 (m, 2H), 2.51–2.34 (m, 2H), 2.10–1.82 (m, 4H), 1.67–1.54 (m, 4H), 1.50 (s, 9H), 0.94 (dd, *J* = 8.6, 6.6 Hz, 6H). ^{13}C NMR (126 MHz, $CDCl_3$) δ 172.67, 170.67, 170.42, 158.46, 155.21, 131.00, 129.98, 128.90, 128.02, 115.74, 115.23, 80.04, 67.36, 56.34, 52.30, 51.11, 51.06, 41.86, 37.86, 33.04, 32.12, 28.33 (3C), 27.66, 24.37, 22.96, 22.12. HRMS (ESI) m/z calcd for $C_{29}H_{43}N_3O_7$ $[M+H]^+$: 546.3173, found 546.3188

(2*S*,5*S*,8*S*,13*S*)-13-((*tert*-butoxycarbonyl)amino)-2,5-diisobutyl-3,6,14-trioxo-1,4,7-triazacyclotetradec-10-ene-8 benzyl ester (15)

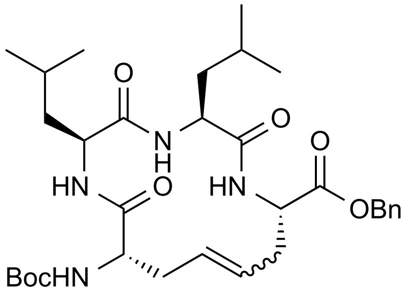

15: $C_{32}H_{48}N_4O_7$
Exact Mass: 600.3523

Following the general procedure for **9a**, the peptide **10** (0.10 g, 16 mmol) was reacted with catalysts **1-7** (10 mol%) in DCE (90 mL) to afford product **15** (80.2 mg, 84%) as a white solid after purification by flash chromatography (SiO_2; 0 to 50% hexanes in EtOAc) (R_f = 0.55 in 4:1 DCM:EtOAc). ^1H NMR (500 MHz, $CDCl_3$) δ 7.45–7.32 (m, 5H), 7.13 (bs, 1H), 6.99 (d, *J* = 8.6 Hz, 1H), 5.87 (d, *J* = 9.9 Hz, 1H), 5.48–5.39 (m, 1H), 5.35–5.30 (m, 1H), 5.25 (d, *J* = 12.6 Hz, 1H), 5.18 (d, *J* = 12.6 Hz, 1H), 5.10 (d, *J* = 8.0 Hz, 1H), 4.78–4.73 (m, 1H), 4.67–4.62 (m, 1H), 4.17–4.07 (m, 2H), 3.14–3.02 (m, 2H), 2.52–2.48 (m, 1H), 2.07 (m, 1H), 1.97 (d, *J* = 10.6 Hz, 1H), 1.85–1.78 (m, 2H), 1.75–1.70 (m, 1H), 1.64–1.51 (m, 2H), 1.49 (s, 9H), 1.03–0.92 (m, 12H). ^{13}C NMR (126 MHz, $CDCl_3$) δ 173.36, 171.72, 171.20, 171.08, 156.08, 135.68, 130.47, 128.53 (2C), 128.07, 127.77, 126.15 (2C), 81.10, 66.78, 55.46, 53.05, 51.64, 50.58, 40.10, 39.73, 28.95, 28.19 (3C), 27.97, 24.95, 24.60, 23.38, 23.31, 21.18, 20.96. HRMS (ESI) m/z calcd for $C_{32}H_{48}N_4O_7$ $[M+H]^+$: 601.3595, found 601.3609

(3*S*,6*S*,9*S*,12*S*)-12-((*tert*-butoxycarbonyl)amino)-6,9-diisobutyl-5,8,11-trioxo-1,14-dioxa-4,7,10-triazacyclooctadec-16-ene-3 methyl ester (16)

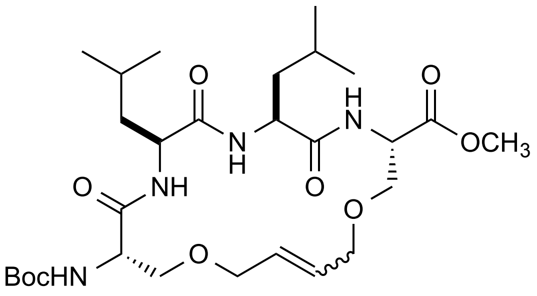

16: $C_{28}H_{48}N_4O_9$
Exact Mass: 584.3421

Following the general procedure for **9a**, the peptide **11** (0.10 g, 16 mmol) was reacted with catalysts **1-7** (10 mol%) in DCE (90 mL) to afford product **16** (83.0 mg, 88%) as a white solid after purification by flash chromatography (SiO_2; 0 to 33% EtOAc in hexanes) (R_f = 0.26 in 2:1 DCM:EtOAc). ^1H NMR (500 MHz, $CDCl_3$) δ 7.27 (d, *J* = 7.4 Hz, 1H), 7.24 (d, J =7.5 Hz, 1H), 6.47 (d, *J* = 7.3 Hz, 1H), 5.95–5.80 (m, 2H), 5.50 (d, *J* = 5.8 Hz, 1H), 4.74–4.71 (m, 1H), 4.51–4.42 (m, 2H), 4.34 (bs, 1H), 4.26–4.13 (m, 2H), 4.04–3.98 (m, 1H), 3.89–3.84 (m, 2H), 3.80 (s, 3H), 3.76–3.65 (m, 2H), 3.50 (bs, 1H), 1.90–1.86 (m, 1H), 1.79–1.50 (m, 5H), 1.48 (s, 9H), 0.99–0.94 (m, 12H). ^{13}C NMR (126 MHz, $CDCl_3$) δ 172.14, 171.39, 170.78, 170.27, 155.67, 129.47, 127.79, 80.34, 71.14, 70.24, 69.61, 69.48, 53.58, 52.81, 52.63, 52.52, 52.44, 40.85, 39.33, 28.26 (3C), 24.86, 24.81, 23.16, 22.91, 21.78, 21.55. HRMS (ESI) m/z calcd for $C_{28}H_{48}N_4O_9$ $[M+H]^+$: 585.3493, found 585.3509

(3*R*,6*S*,9*S*,12*R*)-12-((*tert*-butoxycarbonyl)amino)-6,9-diisobutyl-5,8,11-trioxo-1,14-dithia-4,7,10-triazacyclooctadec-16-ene-3 methyl ester (17)

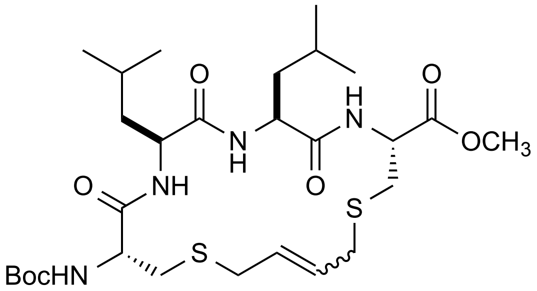

17: $C_{28}H_{48}N_4O_7S_2$
Exact Mass: 616.2964

Following the general procedure for **9a**, the peptide **12** (0.10 g, 15 mmol) was reacted with catalysts **1-7** (10 mol%) in DCE (90 mL) to afford product **17** (77.4 mg, 81%) as a colorless glass after purification by flash chromatography (SiO_2; 0 to 20% EtOAc in hexanes) (R_f = 0.28 in 3:1 DCM:EtOAc). ^1H NMR (500 MHz, $CDCl_3$) δ 7.18 (d, *J* = 8.5 Hz, 1H), 7.06 (d, *J* = 8.8 Hz, 1H), 6.71 (d, *J* = 4.6 Hz, 1H), 5.71–5.53 (m, 2H), 5.33 (d, *J* = 4.2 Hz, 1H), 4.84–4.72 (m, 1H), 4.65–4.61 (m, 1H), 4.36–4.24 (m, 2H), 3.79 (s, 3H), 3.31–3.18 (m, 4H), 3.09–3.01 (m, 2H), 2.76–2.69 (m, 1H), 1.98–1.92 (m, 1H), 1.83–1.72 (m, 2H), 1.66–1.55 (m, 4H), 1.51 (s, 9H), 1.02–0.95 (m, 12H). ^{13}C NMR (126 MHz, $CDCl_3$) δ 172.25, 171.79, 171.39, 171.02, 156.56, 129.58, 129.25, 81.48, 55.15, 54.20, 52.64, 51.75, 51.21, 40.29, 40.01, 35.19, 33.31, 32.53, 28.17 (3C), 25.09, 24.95, 23.25, 23.13, 21.44, 21.16. HRMS (ESI) m/z calcd for $C_{28}H_{48}N_4O_7S_2$ $[M+H]^+$: 617.3036, found 617.3039

methyl (6*S*,11*S*,16a*S*)-11-((*tert*-butoxycarbonyl)amino)-1,4,12-trioxo-1,2,3,4,5,6,7,10,11,12,14,15,16,16a-tetradecahydropyrrolo[1,2-*a*][1,4,7]triazacyclotetradecine-6 methyl ester (18)

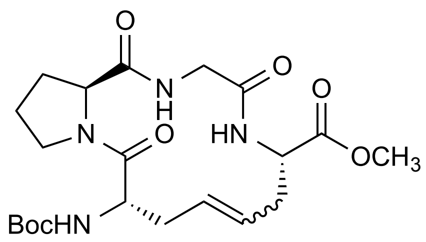

18: $C_{21}H_{32}N_4O_7$
Exact Mass: 452.2271

Following the general procedure for **9a**, the peptide **13** (0.10 g, 21 mmol) was reacted with catalysts **1-7** (10 mol%) in DCE (90 mL) to afford product **18** (52.7 mg, 56%) as a colorless oil after purification by flash chromatography (SiO_2; 1:1 CH_2Cl_2 in EtOAc + 2 to 5% MeOH) (R_f = 0.28 and 0.26 in 1:1 DCM:EtOAc + 10% MeOH).^{1}H NMR (500 MHz, $CDCl_3$, *cis and trans rotamers*) δ 7.45 (d, *J* = 7.8 Hz, 1H), 7.05–7.03 (m, 1H), 5.69 (d, *J* = 7.9 Hz, 1H), 5.47–5.44 (m, 1H), 5.38–5.36 (m, 2H), 4.90–4.86 (m, 1H), 4.69–4.64 (m, 2H), 4.44–4.37 (m, 2H), 3.84 (s, 2H), 3.78 (s, 3H), 3.66–3.64 (m, 2H), 2.65–2.64 (m, 1H), 2.53–2.47 (m, 2H), 2.38–2.30 (m, 2H), 2.17–2.12 (m, 2H), 2.03–1.89 (m, 6H), 1.49 (s, 9H). ^{13}C NMR (126 MHz, $CDCl_3$ *cis and trans rotamers*) δ 172.18, 171.77, 170.80, 168.63, 154.95, 154.84, 128.86, 128.77, 128.26, 127.99, 80.11, 79.69, 60.64, 59.44, 52.72, 52.25, 51.77, 51.25, 47.40, 43.87, 33.74, 33.69, 28.39, 28.34, 27.68, 26.75, 26.65, 26.11, 25.91, 25.84, 25.39, 25.35. HRMS (ESI) m/z calcd for $C_{21}H_{32}N_4O_7$ $[M+H]^+$: 453.2343, found 453.2348

General procedure for Z-selective ethenolysis on macrocyclic peptides

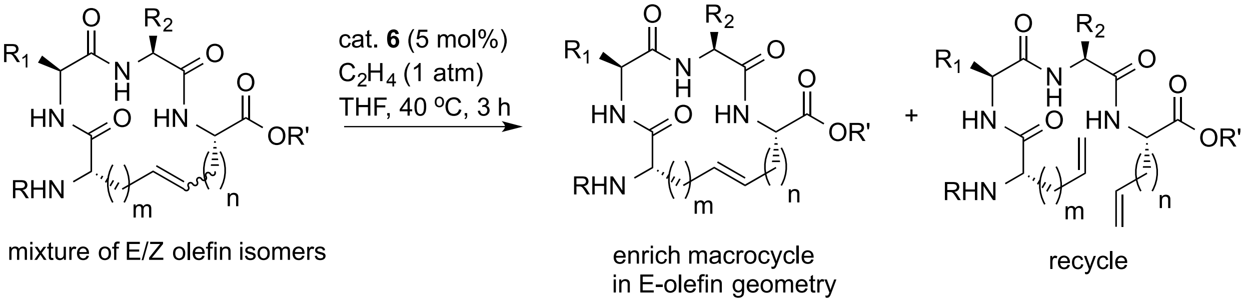

A solution of macrocycle **8a-c** or **15-18** (25.0 mg, mmol) in THF (1 mL) was prepared in a 4 mL vial and sealed with a septum cap. Catalyst **6** (5 mol%) was added as a solution in THF (0.5 mL). The sealed vial was evacuated with ethylene (3 x) and then stirred under an ethylene atmosphere. The reaction was heated to 40 °C for 3 h, then quenched with ethyl vinyl ether and concentrated. The progress of ethenolysis and its influence on olefin selectivity was monitored by LC/MS (Agilent 1100, XDB-C_{18} 3 μm, 4.6 x 50 mm) after independently confirming the product distribution and ratio by NMR. After ethenolysis, the residue was purified by flash chromatography to provide the product enriched highly in the E-isomer. Isolated yields of the pure macrocycles and recovered diene were determined and the recovered diene could be re-exposed to the RCM and ethenolysis

conditions to provide highly enriched macrocyclic peptides in the *E*-olefin geometry. One notable exception was the lack of efficient ethenolysis for compound **18**.

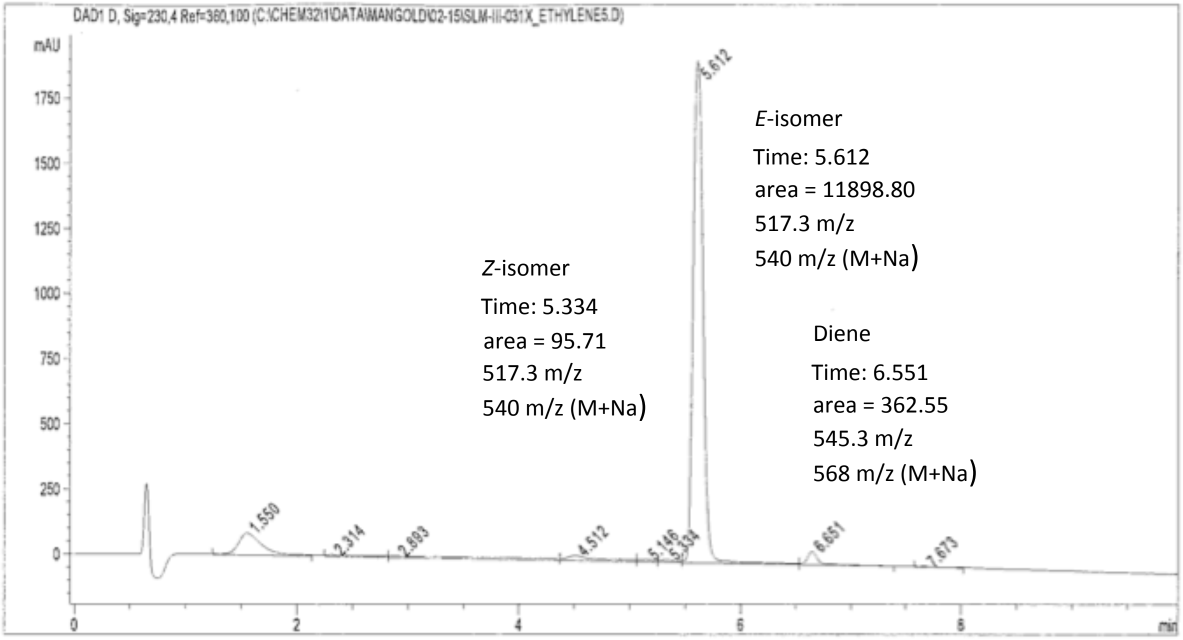

Peak	Start (min)	R_t (min)	End (min)	Height (mAu)	Area (mAu*S)	Area%
1	1.241	1.550	2.135	84.91529	1275.97473	
2	2.248	2.314	2.822	7.22604	159.40733	
3	2.822	2.893	4.372	5.68447	541.43036	
4	4.372	4.512	5.065	18.47627	384.04123	
5	5.065	5.146	5.252	8.47814	82.83534	
6 *Z* isomer	5.262	5.334	5.470	9.17596	95.70734	0.774
7 *E* isomer	5.470	5.612	6.534	1930.46619	11898.8011	96.292
8 Diene	6.534	6.651	7.395	50.67454	362.54980	2.933
9	7.395	7.673	8.021	1.35541	17.83647	

Figure S1: LCMS to assess the conversion and olefin selectivity for Z-selective ethenolysis on macrocyclic peptide **9a**. The percentage of the *E*-isomer, *Z*-isomer, and resultant diene from ethenolysis was calculated from automatic integrations of each peak area. Column conditions: 20-95% acetonitrile:H_2O + 0.1% AcOH

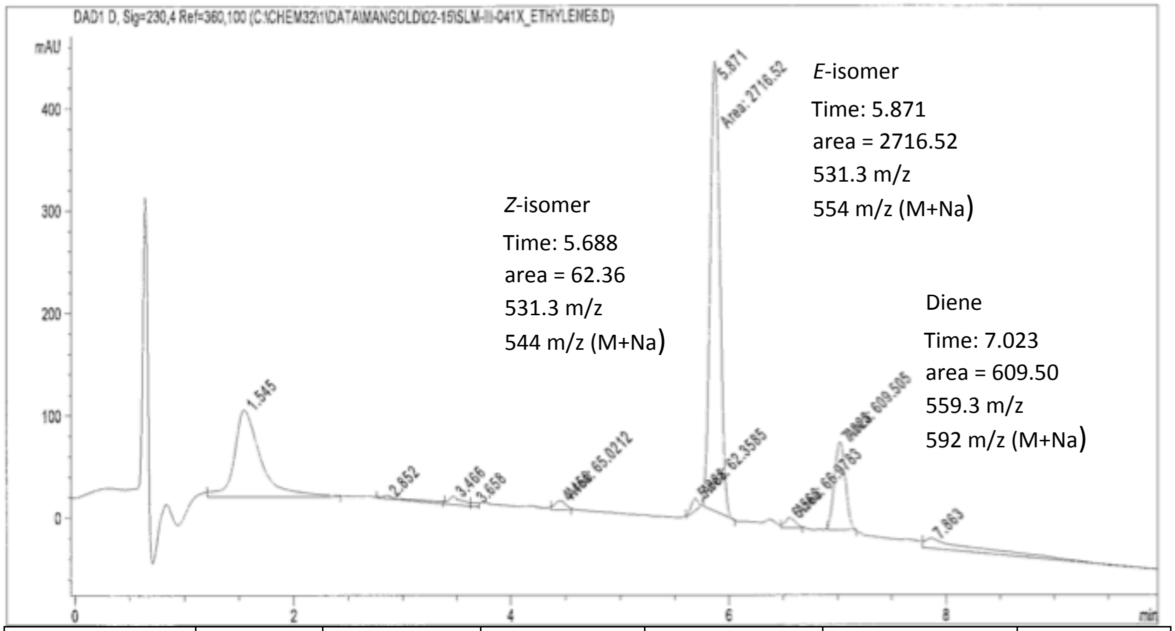

Peak	Start (min)	Rt (min)	End (min)	Height (mAu)	Area (mAu*S)	Area%
1	1.209	1.545	2.428	85.03123	1413.18762	
2	2.761	2.852	3.368	2.85201	58.02650	
3	3.388	3.466	3.626	8.17150	70.54388	
4	3.626	3.658	3.703	4.08593	18.15219	
5	4.365	4.450	4.550	9.13793	65.02124	
6 *Z* isomer	5.600	5.688	5.764	12.36887	62.35853	1.840
7 *E* isomer	5.764	5.871	6.052	441.34851	2716.52002	80.172
8	6.474	6.563	6.680	10.01819	66.97835	17.988
9 Diene	6.904	7.023	7.174	86.09809	609.50470	
10	7.783	7.863	9.962	10.28052	354.32645	

Figure S2: LCMS to assess the conversion and olefin selectivity for Z-selective ethenolysis on macrocyclic peptide **9b**. The percentage of the *E*-isomer, *Z*-isomer, and resultant diene from ethenolysis was calculated from automatic integrations of each peak area. Column conditions: 20-95% acetonitrile:H_2O + 0.1% AcOH

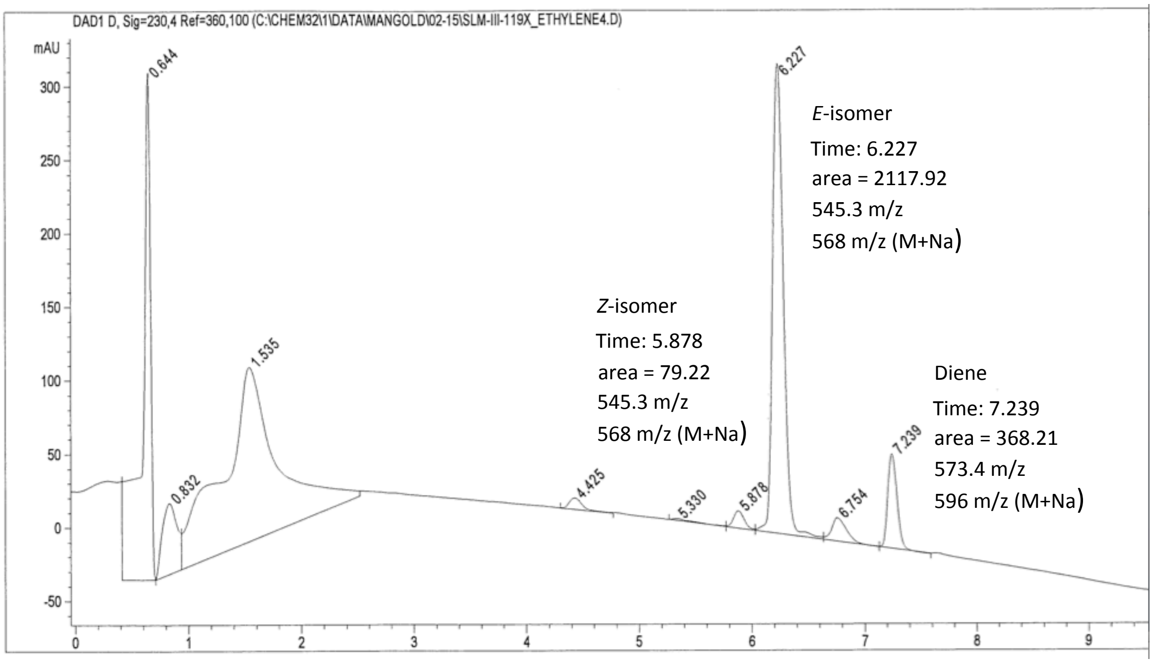

Peak	Start (min)	R_t (min)	End (min)	Height (mAu)	Area (mAu*S)	Area%
1	0.413	0.644	0.708	346.55182	1879.64746	
2	0.708	0.832	0.937	48.39107	441.70938	
3	0.937	1.535	2.520	118.63337	3999.27710	
4	4.299	4.425	4.766	7.96042	64.57545	
5	5.266	5.330	5.770	1.18655	12.70663	
6 Z-isomer	5.770	5.878	6.028	11.95232	79.22633	3.088
7 E-isomer	6.028	6.227	6.630	319.69931	2117.91870	82.558
8	6.630	6.754	7.126	16.02355	143.06432	
9 Diene	7.126	7.239	7.586	64.14093	368.21265	14.353

Figure S3: LCMS to assess the conversion and olefin selectivity for Z-selective ethenolysis on macrocyclic peptide **9c**. The percentage of the E-isomer, Z-isomer, and resultant diene from ethenolysis was calculated from automatic integrations of each peak area. Column conditions: 20-95% acetonitrile:H_2O + 0.1% AcOH

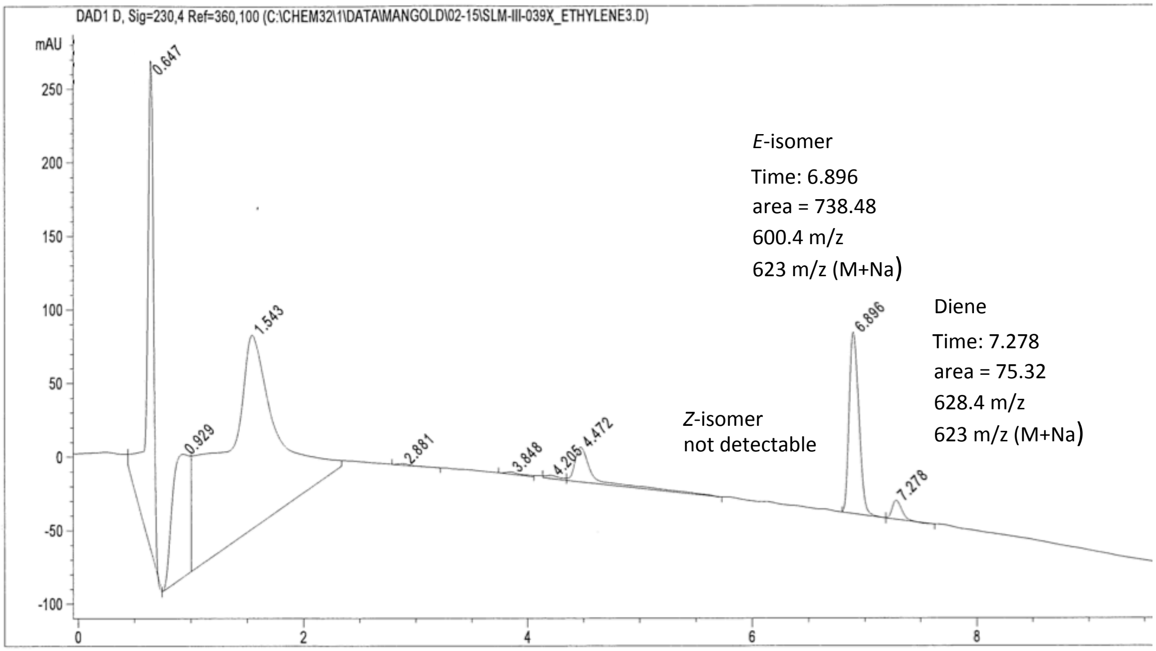

Peak	Start (min)	Rt (min)	End (min)	Height (mAu)	Area (mAu*S)	Area%
1	0.444	0.647	0.745	336.47571	1372.63660	
2	0.745	0.929	1.003	83.83356	903.89703	
3	1.003	1.543	2.337	131.85921	4563.25537	
4	2.789	2.881	3.217	1.07480	5.95085	
5	3.738	3.848	4.057	1.46307	15.35340	
6	4.132	4.205	4.344	2.21722	20.45081	
7	4.344	4.472	5.730	23.62760	255.38373	
8 E isomer	6.796	6.896	7.188	123.37890	738.48621	90.744
9 Diene	7.188	7.278	7.623	12.95432	75.32767	9.256

Figure S4: LCMS to assess the conversion and olefin selectivity for Z-selective ethenolysis on macrocyclic peptide **15**. The percentage of the *E*-isomer, *Z*-isomer, and resultant diene from ethenolysis was calculated from automatic integrations of each peak area. Column conditions: 20-95% acetonitrile:H_2O + 0.1% AcOH

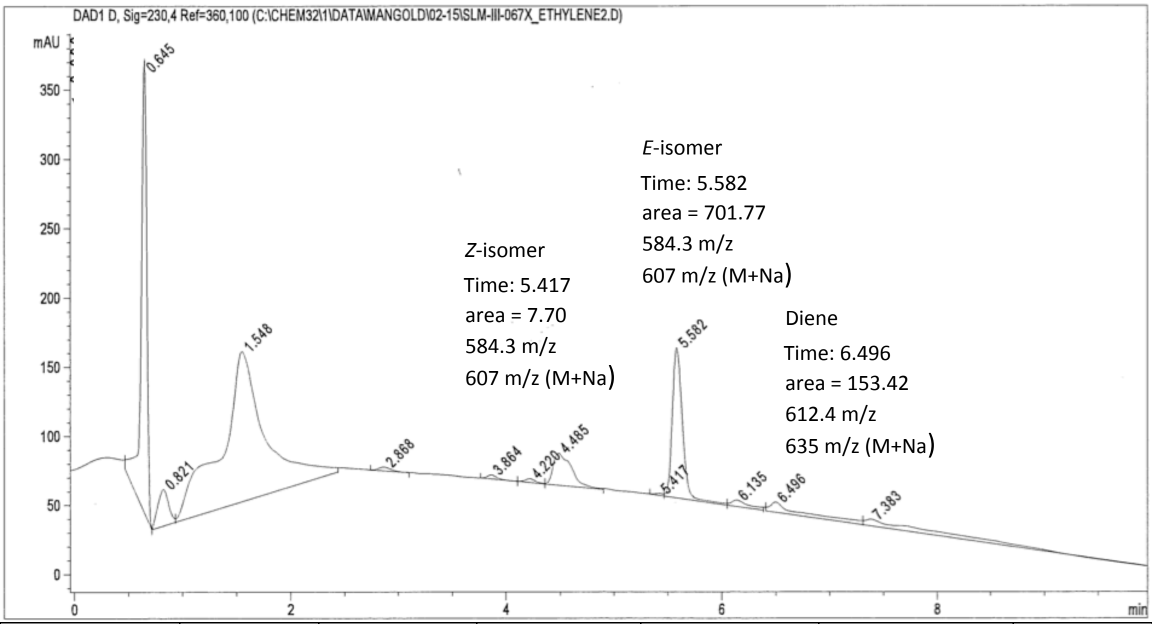

Peak	Start (min)	Rt (min)	End (min)	Height (mAu)	Area (mAu*S)	Area%
1	0.466	0.645	0.712	328.62842	1207.89722	
2	0.712	0.821	0.931	26.75960	188.20444	
3	0.931	1.548	2.439	108.73872	3083.64160	
4	2.741	2.868	3.099	2.80984	21.33830	
5	3.764	3.864	4.105	3.18188	19.36922	
6	4.105	4.220	4.359	3.03480	19.91151	
7	4.359	4.485	4.899	23.28951	271.30936	
8 *Z* isomer	5.333	5.417	5.464	1.34658	7.70495	0.893
9 *E* isomer	5.464	5.582	6.050	108.83810	701.77484	81.327
10	6.050	6.135	6.386	4.74384	55.14216	
11 Diene	6.406	6.496	7.308	7.33192	153.42302	17.780
12	7.308	7.383	9.959	5.06042	248.79102	

Figure S5: LCMS to assess the conversion and olefin selectivity for Z-selective ethenolysis on macrocyclic peptide **16**. The percentage of the *E*-isomer, *Z*-isomer, and resultant diene from ethenolysis was calculated from automatic integrations of each peak area. Column conditions: 20-95% acetonitrile:H_2O + 0.1% AcOH.

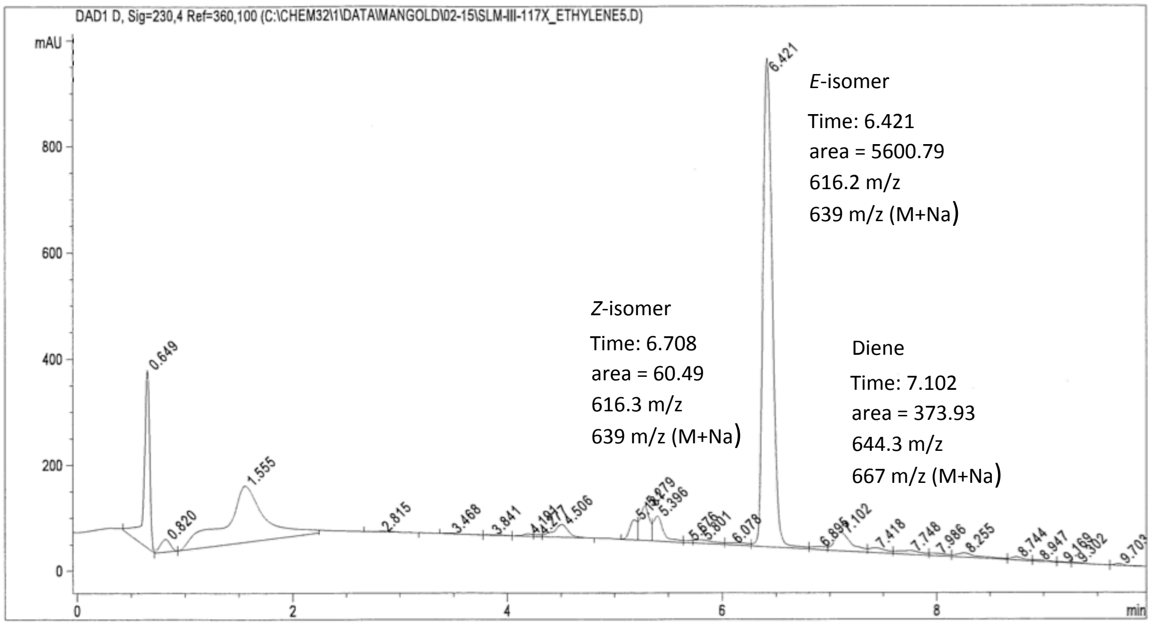

**Peak**	**Start (min)**	**R_t (min)**	**End (min)**	**Height (mAu)**	**Area (mAu*S)**	**Area%**
1	0.420	0.649	0.715	336.21109	1241.97314	
2	0.715	0.820	0.931	24.67960	159.74443	
3	0.931	1.555	2.247	106.90371	2754.27417	
4	4.044	4.191	4.242	4.43836	25.76578	
5	4.242	4.277	4.326	4.05187	18.65500	
6	4.326	4.506	4.807	24.19786	220.44963	
7	5.061	5.181	5.213	38.58390	181.25850	
8	5.213	5.279	5.346	65.12208	391.30566	
9	5.346	5.396	5.639	47.43619	319.13754	
10	5.639	5.676	5.725	4.20501	21.50261	
11	5.725	5.801	6.024	6.89706	86.88933	
12 *Z* isomer	6.024	6.078	6.268	4.05960	60.49083	1.003
13 *E* isomer	6.268	6.421	6.811	921.42847	5600.79346	92.802
14	6.811	6.895	6.984	5.87108	55.82880	
15 Diene	6.984	7.102	7.350	34.27073	373.93195	6.195
16	7.350	7.418	7.592	9.25945	97.96252	
17	7.592	7.748	7.926	7.32276	109.53992	
18	7.926	7.986	8.140	4.98148	53.86921	
19	8.140	8.255	8.662	9.04783	125.62745	

Figure S6: LCMS to assess the conversion and olefin selectivity for Z-selective ethenolysis on macrocyclic peptide **17**. The percentage of the *E*-isomer, *Z*-isomer, and resultant diene from ethenolysis was calculated from automatic integrations of each peak area. Column conditions: 20-95% acetonitrile:H_2O + 0.1% AcOH.

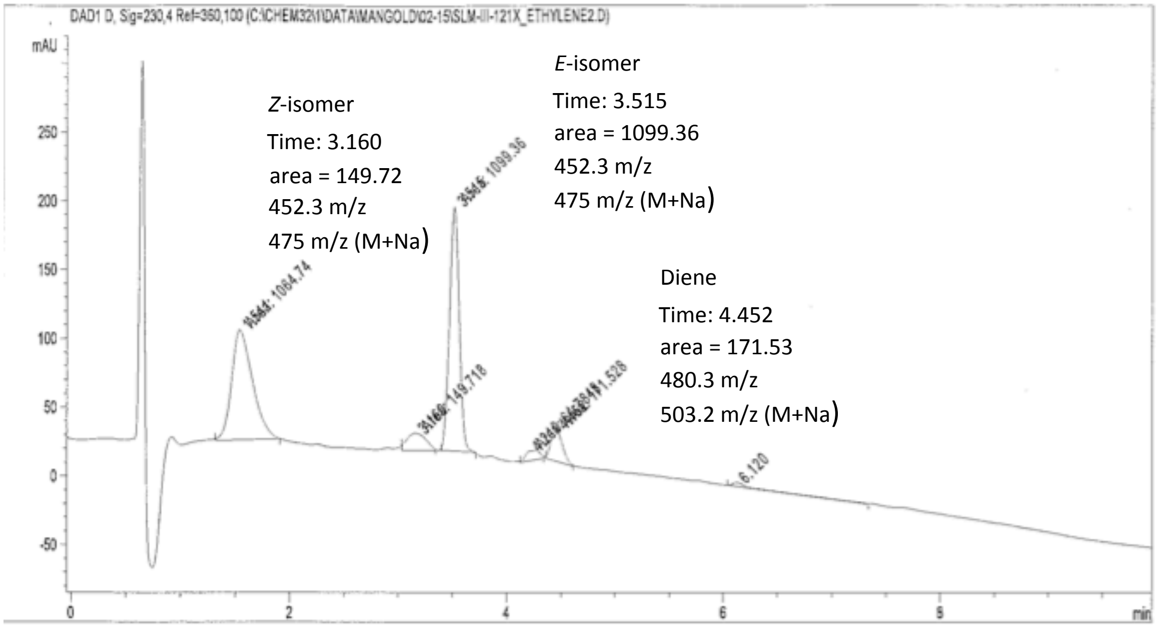

Peak	Start (min)	R_t (min)	End (min)	Height (mAu)	Area (mAu*S)	Area%
1	1.317	1.541	1.913	80.00210	1064.74316	
2 *Z* isomer	3.035	3.160	3.343	12.34278	149.71841	10.539
3 *E* isomer	3.395	3.515	3.714	178.45915	1099.35583	77.387
4	4.125	4.210	4.341	7.06685	61.78477	
5 Diene	4.362	4.452	4.609	23.17986	171.52794	12.074
6	6.036	6.120	7.339	2.85999	34.18794	

Figure S7: LCMS to assess the conversion and olefin selectivity for Z-selective ethenolysis on macrocyclic peptide **18**. The percentage of the E-isomer, Z-isomer, and resultant diene from ethenolysis was calculated from automatic integrations of each peak area. Column conditions: 20-95% acetonitrile:H_2O + 0.1% AcOH

Percentage enrichment of macrocycles 9a-c and 15-18 by Z-selective ethenolysis

Compound	Initial E (%)	Final E (%)
9a	96	99
9b	80	97
9c	82	96
15	90	>99
16	81	98
17	90	99
18	77	88

Solid phase synthesis of peptides

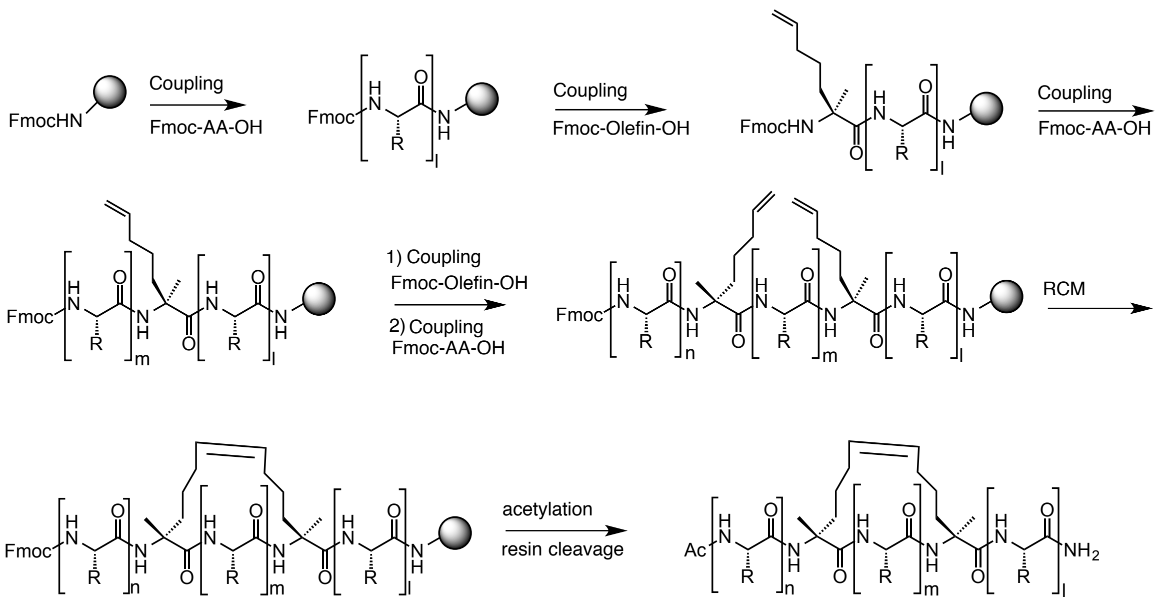

Peptides were synthesized manually or produced on a Titan 357 (AAPPTec, Louisville, KY) automated peptide synthesizer using Rink Amide MBHA resin (NovaBioChem, 0.4 mmol/g resin), at 80 μmol scale. The resin was swelled with N-Methyl 2-pyrrolidinone (NMP, 10 mL) for 30 min before use. To load the first amino acid onto the resin, the resin-bound Fmoc-protecting group was removed by treatment with 25% (vol/vol) piperidine in NMP (2 x 10 min). Standard amino acids were coupled for 1.5 h using HATU as the activating agent (4 eq. based on loading capacity), Fmoc-protected amino acid (5 eq.), and N,N-diisopropylethylamine (DIEA, 10 eq.) in NMP (2 mL). After each coupling or deprotection reaction, the resin was washed successively with DCM (1 x 1 min), NMP (1 x 1 min), DCM (1 x 1 min) and NMP (1 x 1 min). For the coupling of olefinic amino acids, a reaction time of 8 h was used with Fmoc-(S)-2-(4-pentenyl)alanine (3 eq.) or Fmoc-(R)-2-(7-octenyl)alanine (3 eq.), DIC (3 eq.), HOBt (6 eq.) in NMP (2 mL). After the final amino acid coupling, the resin was washed with DCM (2 x 1 min) and dried *in vacuo* overnight.

For N-terminal acetylation of the peptide, the resin was swelled with NMP (1 mL) for 20 min and then washed with NMP (2 x 1 min). The resin was treated with 25% (vol/vol) piperidine in NMP (2 mL), gently agitated for 20 min, and then drained. The resin was washed with DCM (5 x 2 min) and allowed to dry to afford the amine-terminated peptide. To this was added NMP (1 mL), and the solvent drained. Acetic anhydride (60 μL, 0.6 mmol, 30 eq.) in NMP (1.0 mL) was added, followed by N,N-diisopropylethylamine (DIEA, 208 μL, 60 eq.) and the resin was agitated for 45 min at rt. The resin was then washed with DCM (1 x 1 min), NMP (1 x 1 min), DCM (1 x 1 min) and dried under a stream of argon for 4 h.

Cleavage of the peptide from the resin and global deprotection were achieved by reacting the resin with 95% TFA, 2.5% triisopropylsilane (TIS), 2.5% H_2O (vol/vol/vol) for 4 h. The TFA and other volatiles were removed by evaporation under a stream of argon. The peptides were precipitated with cold diethyl ether (4 mL), vortexed, and collected by centrifugation. The pellet was dried under a stream of argon and subsequently dissolved in

a mixture of 50% acetonitrile, 50% H_2O (vol/vol) and the resin removed by filtration. The cleaved peptides were purified by reverse-phase HPLC using a Zorbax C_8 or C_{18} column (Agilent, 5 um, 9.4 x 250 mm) and characterized by LC/MS TOF using a Zorbax C_8 column (Agilent, 3.5 um, 2.1 x 150 mm) or matrix-assisted laser desorption ionization time-of-flight (MALDI-TOF)

General procedure for RCM on resin-bound olefinic peptides

The N-terminal modified peptide on resin (50 mg, 0.02 mmol) was dissolved in degassed dichloroethane (DCE, 4.0 mL). To this was added a stock solution of ruthenium catalyst **1-7** in degassed DCE (40 μL of a 0.05 M solution in DCE). The reaction was stirred under a gentle stream of Ar(g) for 2 h, the catalyst was filtered off, and the resin washed first with DCE (5 x 2 min) and then with DMF (2 x 2 min). Exposure of the resin bound peptide to an additional round of catalyst stock solution (40 μL) for 4 h ensured nearly quantitative conversion. Upon completion of RCM, the resin bound peptide was washed with DCE (2 x 2 min), DMF (2 x 2 min), and DCM (2 x 2 min) and dried under vacuum.

Cleavage of the peptide from the resin and global deprotection were achieved by reacting the resin with 95% TFA, 2.5% triisopropylsilane, 2.5% H_2O (vol/vol/vol) for 2 h. The TFA and other volatiles were removed by evaporation under a stream of argon. The peptides were precipitated with cold diethyl ether (4 mL), vortexed, and collected by centrifugation. The pellet was dried under a gentle stream of argon and subsequently dissolved in a mixture of 50% acetonitrile, 50% H_2O (vol/vol) and the resin was removed by filtration. The cleaved peptides were purified by reverse-phase HPLC using a Zorbax C_8 or C_{18} column (Agilent, 5 μm, 9.4 x 250 mm) and characterized by LC/MS TOF using a Zorbax C_8 column (Agilent, 3.5 μm , 2.1 x 150 mm) or matrix-assisted laser desorption ionization time-of-flight (MALDI-TOF).

Monitoring the conversion of RCM on resin-bound olefinic peptides

The percentage conversion of RCM on Fmoc-protected peptides was achieved by taking aliquots of the resin suspension (25 μL) from the reaction mixture at variable time points, quenching with ethyl vinyl ether (50 μL), filtering, and washing with DCE (300 μL). The resin was dried under a stream of argon and suspended in 500 μL of the cleavage cocktail TFA/TIS/H_2O (95:2.5:2.5) and allowed to stir at room temperature for 1 h. The TFA and other volatiles were removed by evaporation and the crude residue dissolved in diethyl ether (200 μL), vortexed, and centrifuged. The ether was carefully decanted and the pellet was dried under a stream of argon. The pellet was dissolved in 100 μL of 50% (vol/vol) aqueous acetonitrile and filtered to afford the crude peptide. For LC/MS TOF analysis, 5 μL of dissolved peptide was injected onto an analytical column (Eclipse Plus C_8 column (1.8 μm, 2.1 x 50 mm)) operating in positive electrospray ionization (ESI) mode.

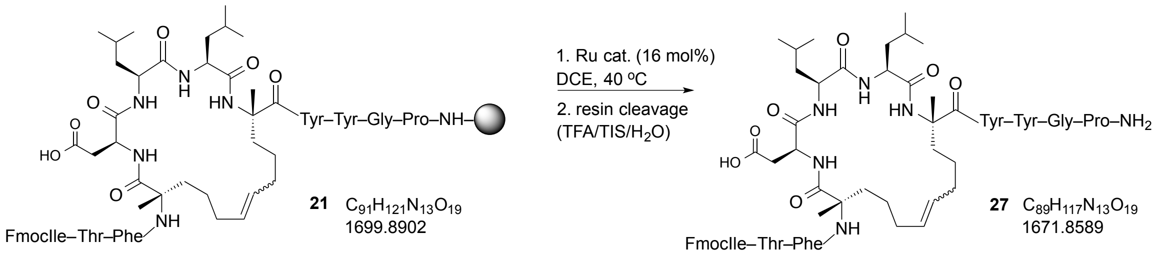

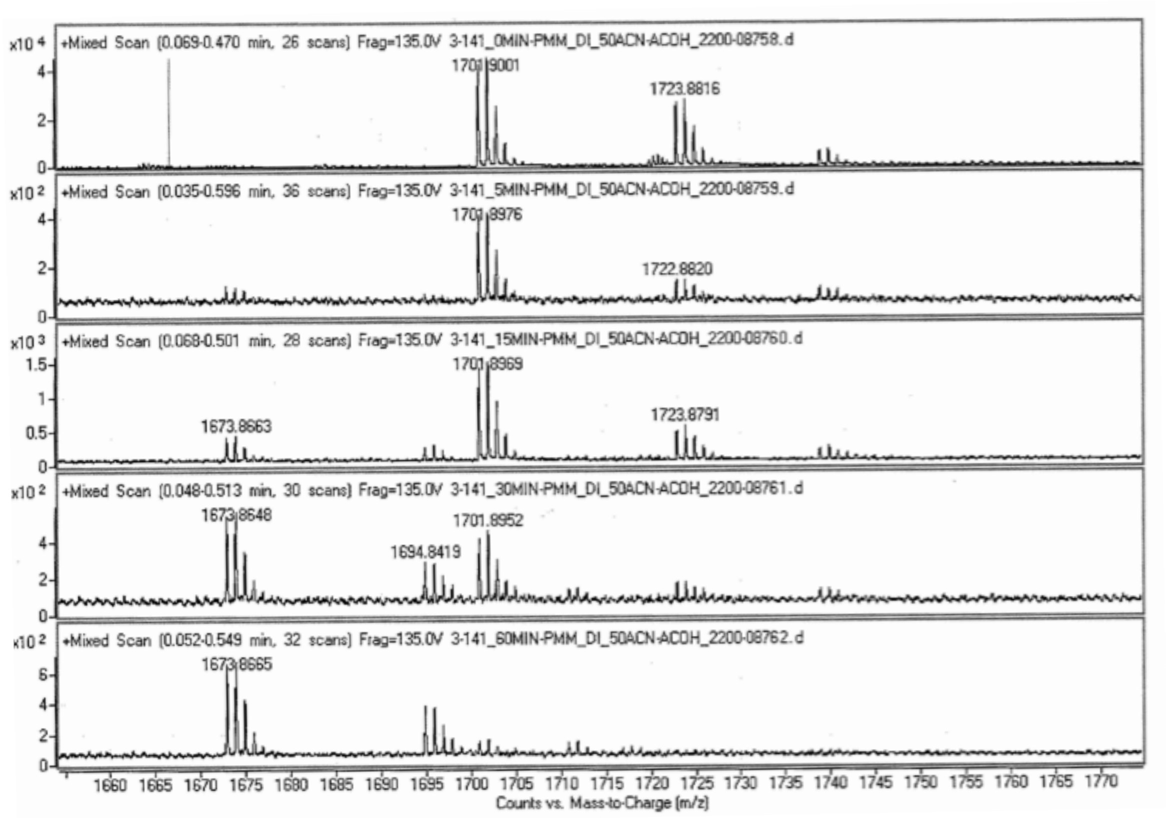

Figure S8*:* Evaluation of RCM on peptide **21** as a function of time (t = 0 to 60 min). The indicated masses corresponding to starting material **21** (1701.9001) and product **27** (1673.8665) is observed as the $[M+H]^+$ ion as measured by LC/MS TOF

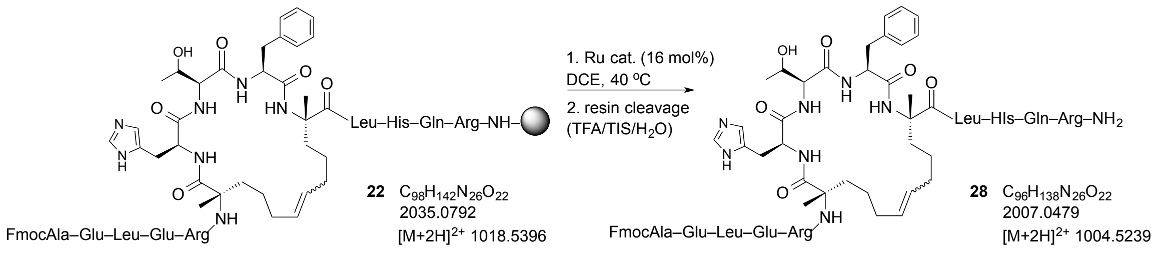

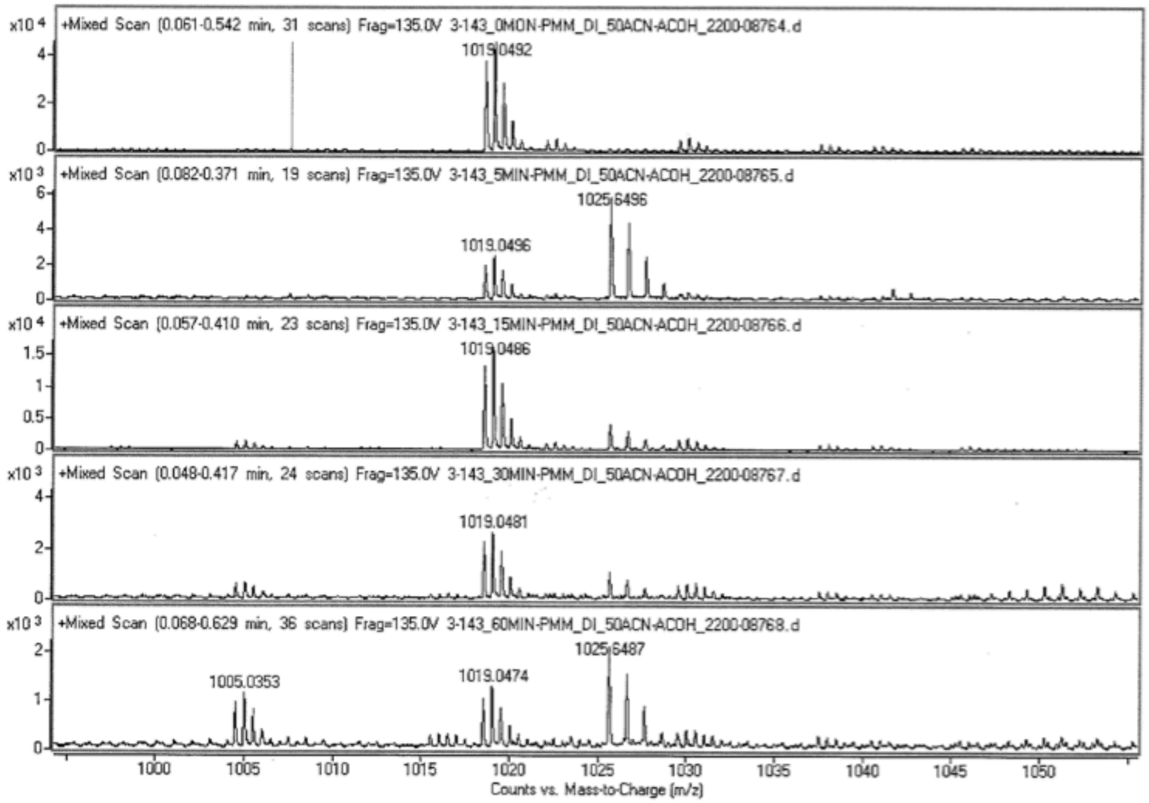

Figure S9*:* Evaluation of RCM on peptide **22** as a function of time (t = 0 to 60 min). The indicated mass corresponding to starting material **22** (1019.0492) and product **28** (1005.0353) is observed as the $[M+2H]^{2+}$ ion as measured by LC/MS TOF

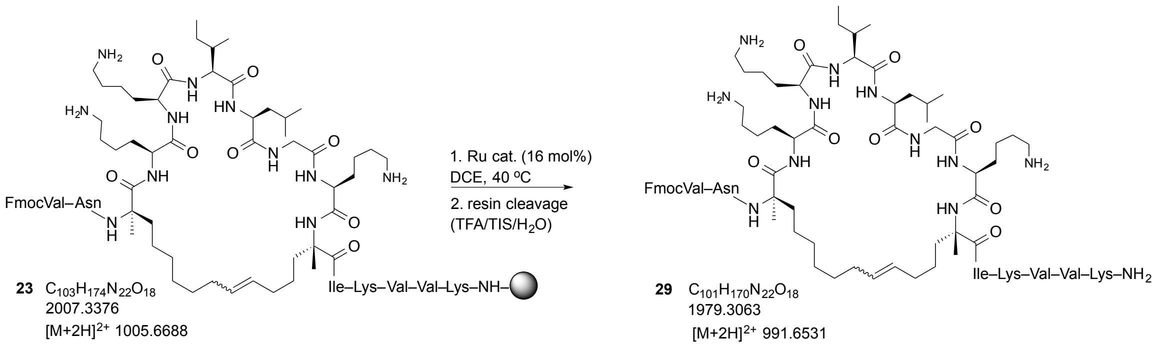

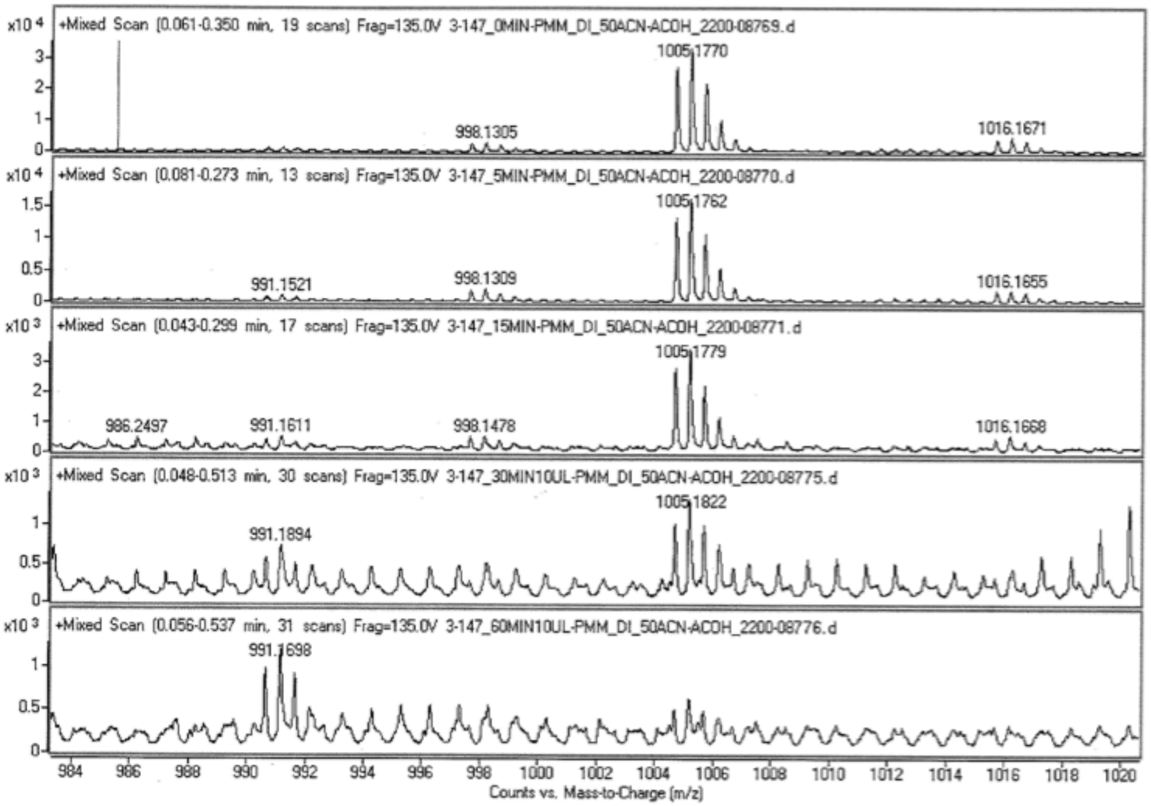

Figure S10*:* Evaluation of RCM on peptide **23** as a function of time (t = 0 to 60 min). The indicated mass corresponding to starting material **23** (1005.1770) and product **29** (991.1698) is observed as the $[M+2H]^{2+}$ ion as measured by LC/MS TOF

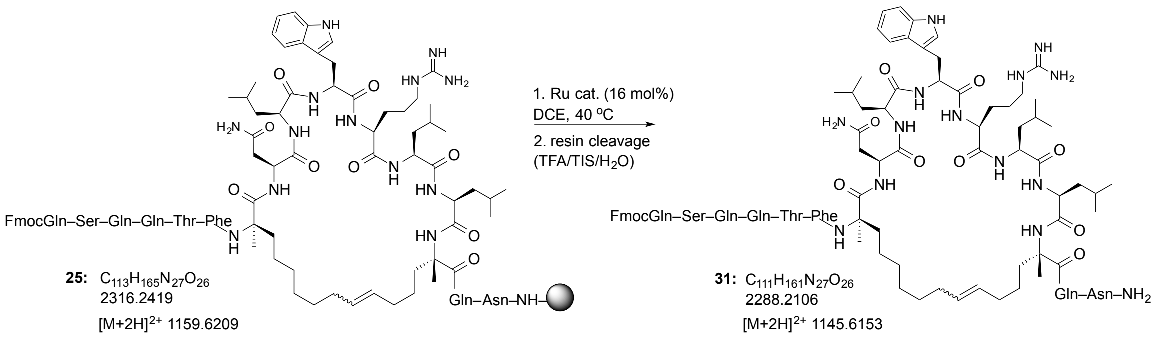

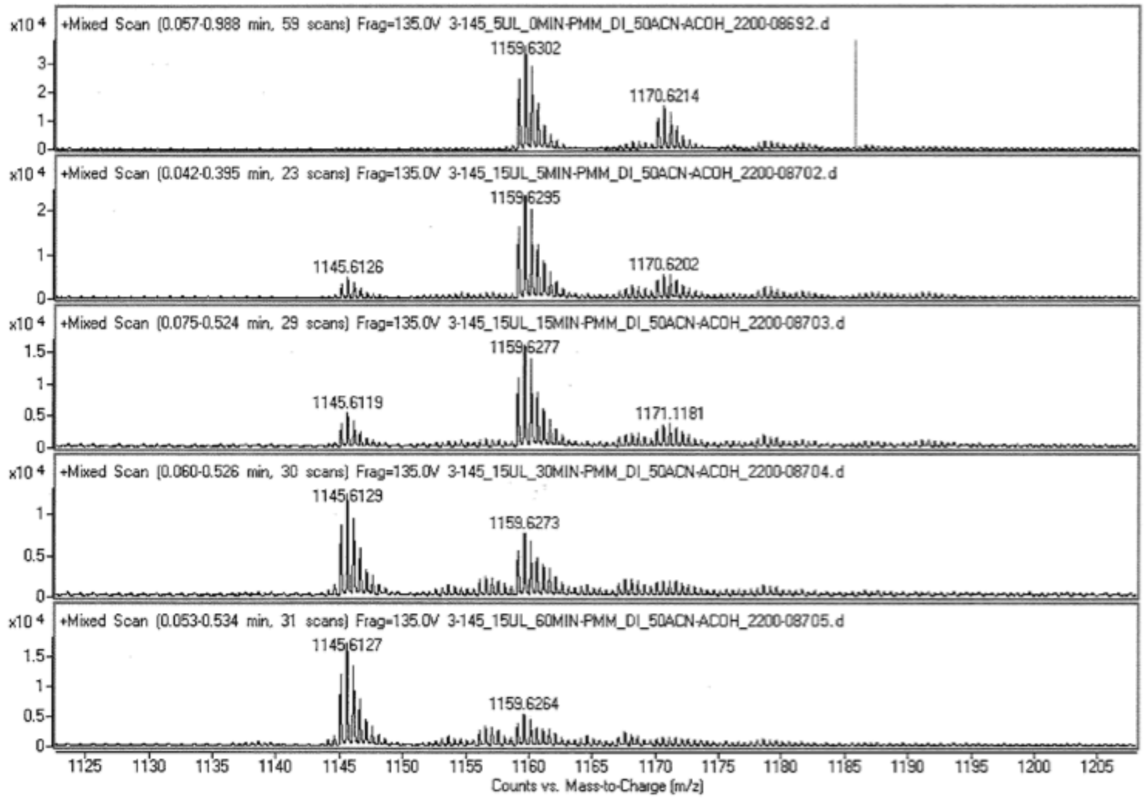

Figure S11*:* Evaluation of RCM on Fmoc-protected peptide **25** as a function of time (t = 0 to 60 min). The indicated mass corresponding to starting material **25** (1159.6264) and product **31** (1145.6127) is observed as the $[M+2H]^{2+}$ ion as measured by LC/MS TOF

General procedure for Z-selective ethenolysis on resin-bound peptides

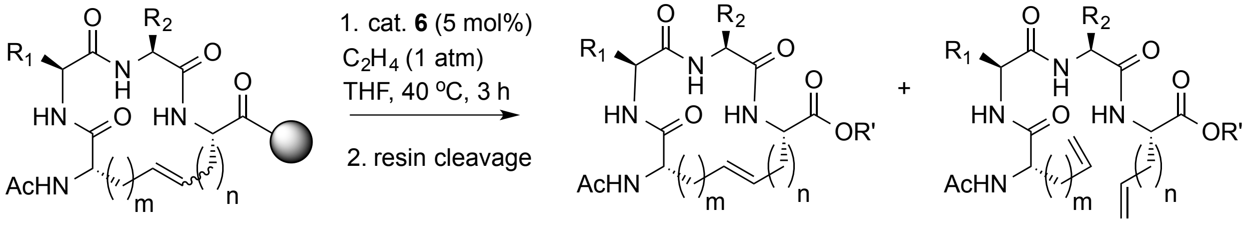

The procedure for ethenolysis on resin-bound peptides **26-31** was conducted in a manner similar to conditions for Z-selective ethenolysis on peptides **8a-c** and **15-19** with minor modification. Briefly, 50 mg of resin bearing the N-terminal acetylated peptide was added to a 4 mL vial equipped with a septum. THF (1 mL) was added, followed by catalyst **6** (20 μL of a 0.05 M stock solution in THF) and the reaction flask evacuated with ethylene (3x) and stirred at an ethylene pressure at 40 °C for 3 h. At this point, the solvent was filtered and the resin washed with THF (2 x 1 mL). To the resin was added a solution of TFA/H_2O/triisopropylsilane (95:2.5:2.5 v/v/v, 500 μL) and the resin agitated for 1 h. The TFA and other volatiles were removed via a stream of argon, and the crude peptide and resin were suspended in aqueous acetonitrile (50:50 v/v, 100 μL), and the resin was filtered off. For LC/MS analysis, 10 μL of the filtrate was diluted with 1:1 (v/v) aqueous acetonitrile (500 μL).

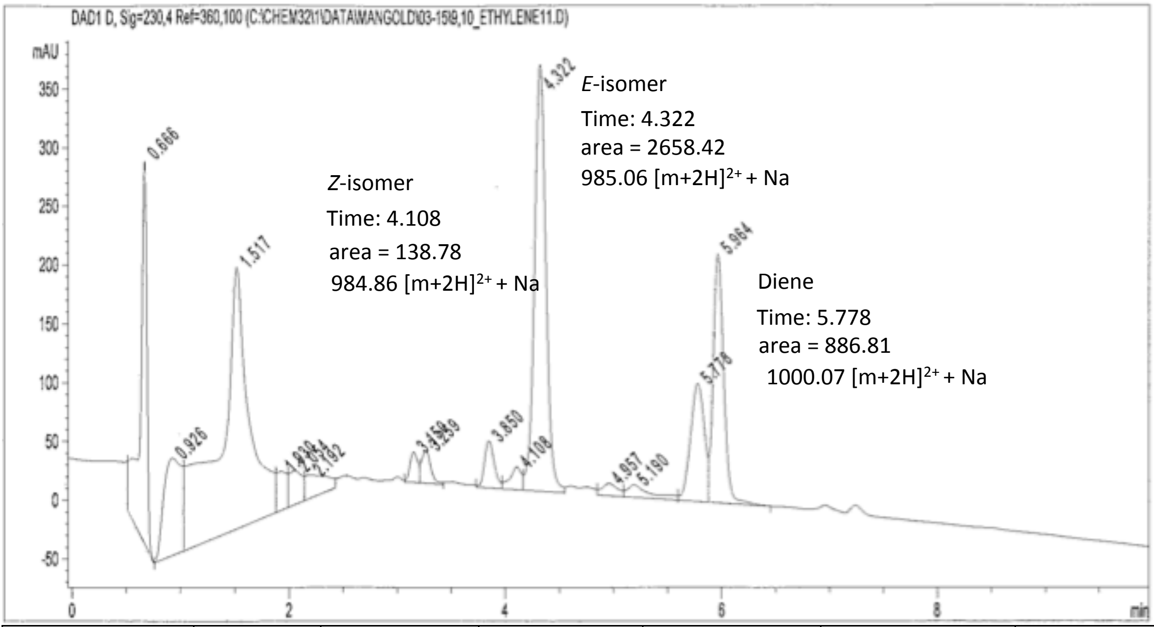

Peak	Start (min)	R_t (min)	End (min)	Height (mAu)	Area (mAu*S)	Area%
1	0.509	0.666	0.756	328.06650	1390.04016	
2	0.756	0.926	1.028	82.98902	943.99445	
3	1.028	1.517	1.882	222.95676	4288.41797	
4	1.882	1.930	1.990	33.20190	207.45451	
5	1.990	2.054	2.139	29.15939	238.06068	
6	2.139	2.192	2.428	20.00443	266.20630	
7	3.062	3.150	3.202	26.24897	127.78748	
8	3.202	3.259	3.422	28.63281	169.41917	
9	3.726	3.850	3.974	40.15965	257.21872	
10 Z isomer	3.974	4.108	4.167	18.44371	138.78113	3.767
11 E isomer	4.167	4.322	4.549	363.16830	2658.42139	72.161
12	4.851	4.957	5.092	10.55256	107.82548	
13	5.092	5.190	5.596	10.96348	165.98024	
14 Diene	5.596	5.778	5.874	100.87401	886.81006	24.072
15	5.874	5.964	6.449	212.34502	1452.62390	

Figure S12: LCMS to assess the conversion and olefin selectivity for Z-selective ethenolysis on macrocyclic peptide **26**. The percentage of the E-isomer, Z-isomer, and resultant diene from ethenolysis was calculated from automatic integrations of each peak area. Column conditions: 5-95% acetonitrile:H_2O + 0.1% AcOH

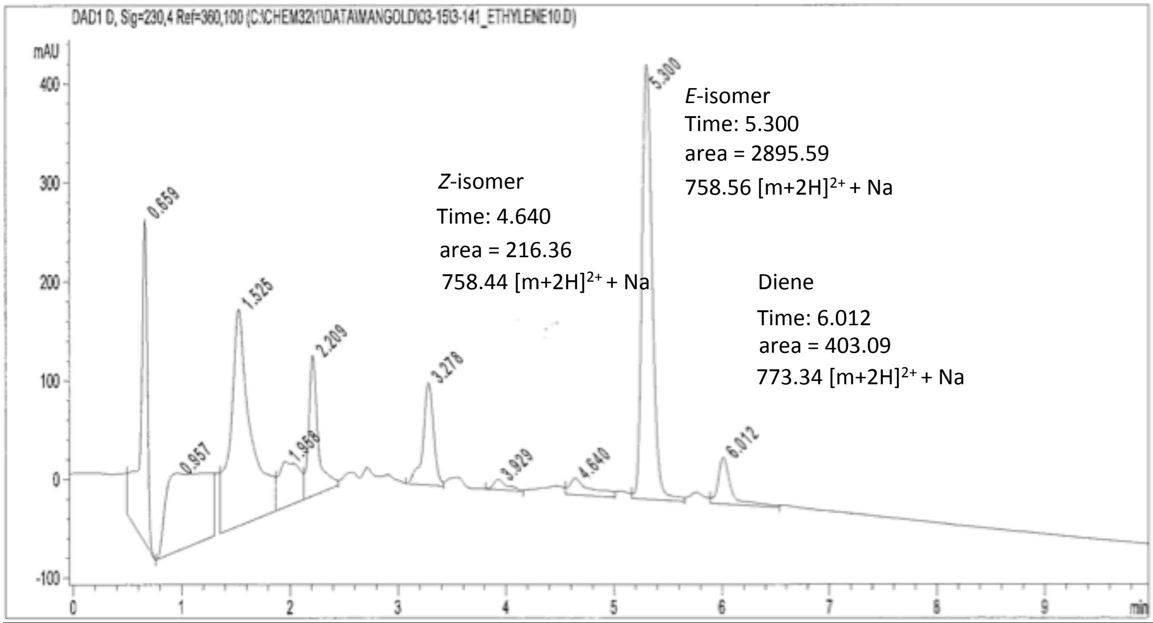

Peak	Start (min)	R_t (min)	End (min)	Height (mAu)	Area (mAu*S)	Area%
1	0.495	0.659	0.760	329.17676	1456.77148	
2	0.760	0.957	1.320	78.93261	2048.06274	
3	1.355	1.525	1.867	220.29582	2850.60815	
4	1.867	1.958	2.123	46.43423	591.10437	
5	2.123	2.209	2.446	141.96553	865.81653	
6	3.074	3.279	3.418	104.29708	708.66333	
7	3.808	3.929	4.159	10.90910	95.11992	
8 Z isomer	4.549	4.640	5.006	17.12723	216.36253	6.155
9 E isomer	5.163	5.300	5.655	440.54013	2895.59058	82.377
10 Diene	5.891	6.012	6.535	47.50585	403.09421	11.467

Figure S13: LCMS to assess the conversion and olefin selectivity for Z-selective ethenolysis on macrocyclic peptide **27**. The percentage of the E-isomer, Z-isomer, and resultant diene from ethenolysis was calculated from automatic integrations of each peak area. Column conditions: 5-95% acetonitrile:H_2O + 0.1% AcOH

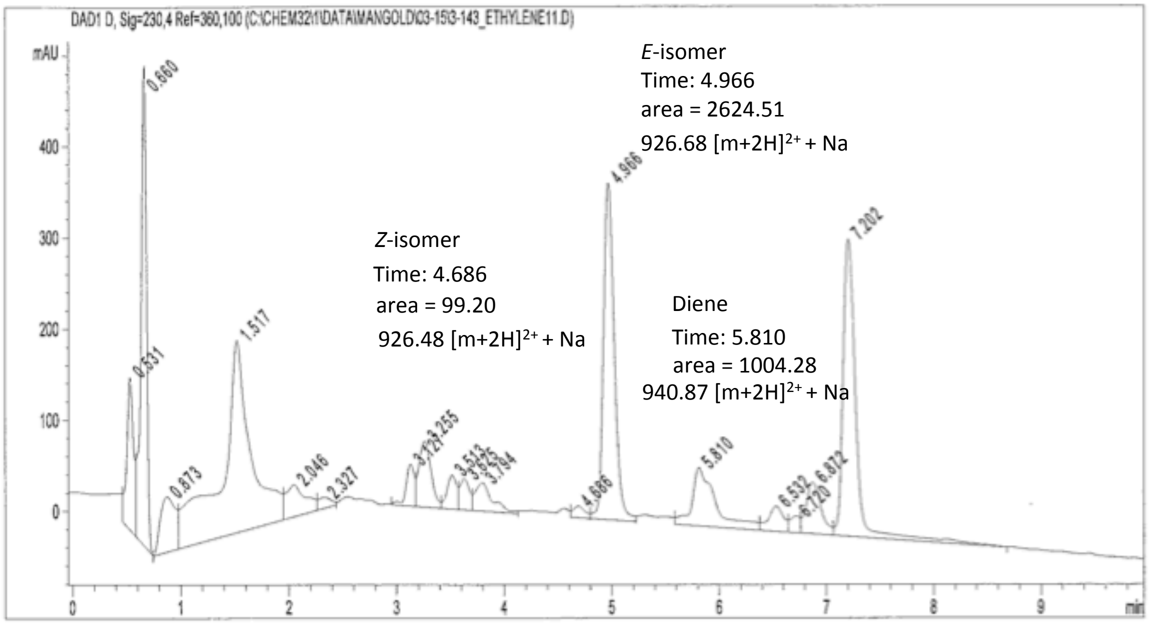

Peak	Start (min)	R_t (min)	End (min)	Height (mAu)	Area (mAu*S)	Area%
1	0.462	0.531	0.584	167.92915	705.33002	
2	0.584	0.660	0.745	528.87872	1868.56311	
3	0.745	0.873	0.974	60.94785	577.99658	
4	0.974	1.517	1.945	211.31032	4026.08667	
5	1.945	2.046	2.258	35.25732	447.11069	
6	2.258	2.327	2.438	12.11003	99.69940	
7	2.949	3.127	3.173	46.12304	244.60857	
8	3.173	3.255	3.408	72.28084	573.46991	
9	3.408	3.513	3.572	37.38420	235.83276	
10	3.572	3.625	3.701	34.31423	209.67151	
11	3.701	3.794	4.122	31.37045	350.17886	
12 Z isomer	4.614	4.686	4.798	12.77934	99.20309	2.661
13 E isomer	4.798	4.966	5.226	369.50021	2624.51563	70.400
14 Diene	5.583	5.810	6.376	64.16768	1004.27820	26.939
15	6.376	6.532	6.642	27.74984	274.77026	
16	6.642	6.720	6.761	18.47508	117.75485	
17	6.761	6.872	7.061	57.09723	544.13873	
18	7.061	7.202	8.682	326.45541	2733.85864	

Figure S14: LCMS to assess the conversion and olefin selectivity for Z-selective ethenolysis on macrocyclic peptide **28**. The percentage of the E-isomer, Z-isomer, and resultant diene from ethenolysis was calculated from automatic integrations of each peak area. Column conditions: 5-95% acetonitrile:H_2O + 0.1% AcOH

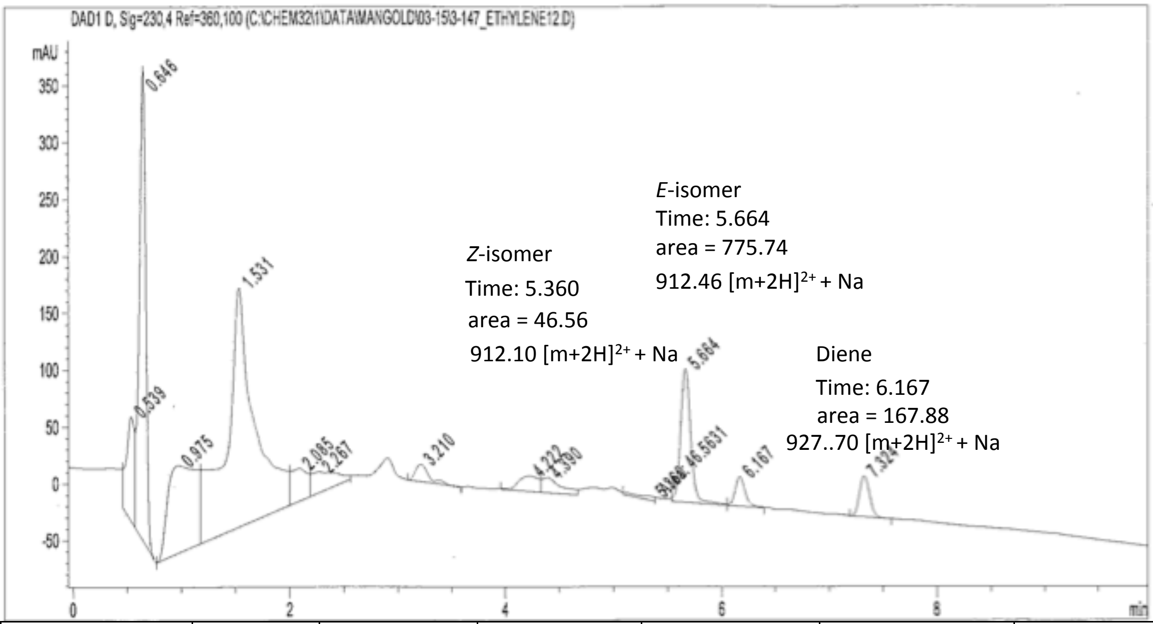

Peak	Start (min)	R_t (min)	End (min)	Height (mAu)	Area (mAu*S)	Area%
1	0.460	0.539	0.570	92.57001	450.46036	
2	0.570	0.646	0.768	417.97043	1720.84460	
3	0.765	0.975	1.176	77.11630	1406.33704	
4	1.176	1.531	1.998	210.80536	3862.95581	
5	1.998	2.085	2.186	29.16211	300.56073	
6	2.186	2.267	2.557	18.48628	283.28735	
7	3.092	3.210	3.586	15.28884	131.22969	
8	3.960	4.222	4.328	13.59650	172.75735	
9	4.328	4.390	4.668	13.28523	165.70427	
10 Z isomer	5.088	5.360	5.386	3.56803	46.56314	4.702
11 E isomer	5.533	5.664	6.049	117.7833	775.74634	78.343
12 Diene	6.049	6.167	6.393	26.00421	167.88136	16.954
13	7.190	7.324	7.573	35.94275	235.39198	

Figure S15: LCMS to assess the conversion and olefin selectivity for Z-selective ethenolysis on macrocyclic peptide **29**. The percentage of the E-isomer, Z-isomer, and resultant diene from ethenolysis was calculated from automatic integrations of each peak area. Column conditions: 5-95% acetonitrile:H_2O + 0.1% AcOH

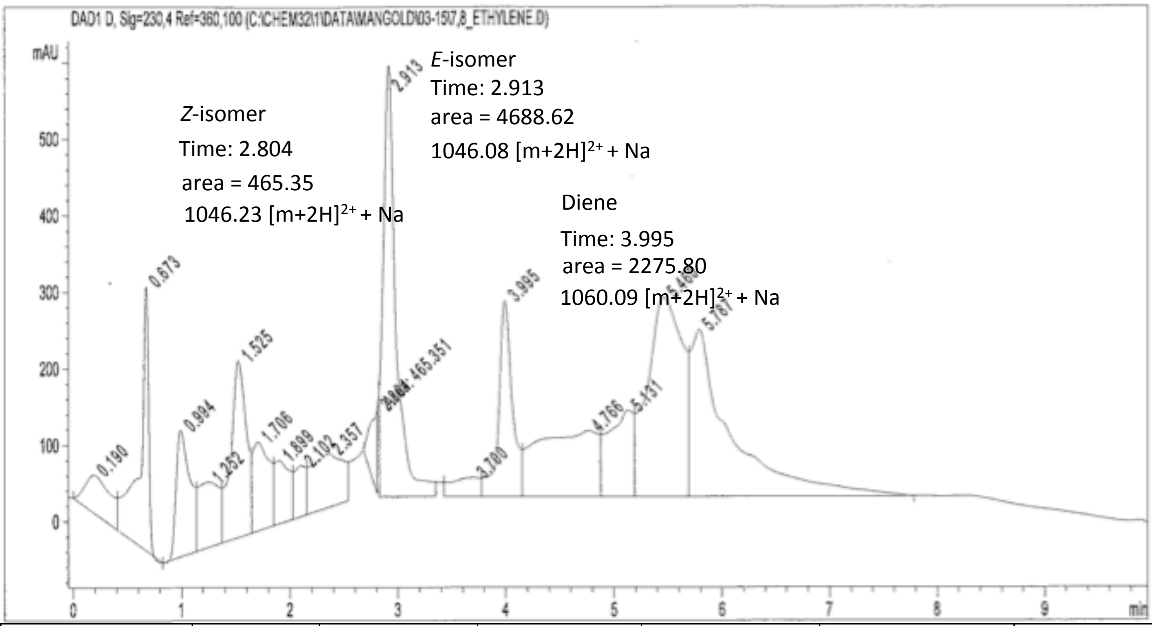

Peak	Start (min)	R_t (min)	End (min)	Height (mAu)	Area (mAu*S)	Area%
1	0.000	0.190	0.411	49.50397	866.64758	
2	0.411	0.673	0.829	345.84637	1980.53406	
3	0.829	0.994	1.137	165.26991	1479.47595	
4	1.137	1.252	1.372	85.72522	1158.23145	
5	1.372	1.525	1.646	231.50479	2337.08350	
6	1.646	1.706	1.850	116.38415	1236.74084	
7	1.850	1.899	2.023	83.05311	778.40820	
8	2.023	2.102	2.152	66.59915	498.29349	
9	2.152	2.357	2.534	70.39770	1441.54053	
10 Z isomer	2.681	2.804	2.810	109.36814	465.35052	6.263
11 E isomer	2.824	2.913	3.350	565.48346	4688.62109	63.105
12	3.424	3.700	3.776	25.38783	476.28745	
13 Diene	3.776	3.995	4.155	256.55038	2275.80347	30.631
14	4.155	4.766	4.878	86.66356	3346.67285	
15	4.878	5.131	5.194	113.33173	1830.36438	
16	5.194	5.460	5.689	261.37427	5829.7660	
17	5.689	5.787	7.784	218.59744	5794.22803	

Figure S16: LCMS to assess the conversion and olefin selectivity for Z-selective ethenolysis on macrocyclic peptide **30.** The percentage of the E-isomer, Z-isomer, and resultant diene from ethenolysis was calculated from automatic integrations of each peak area. Column conditions: 5-95% acetonitrile:H_2O + 0.1% AcOH

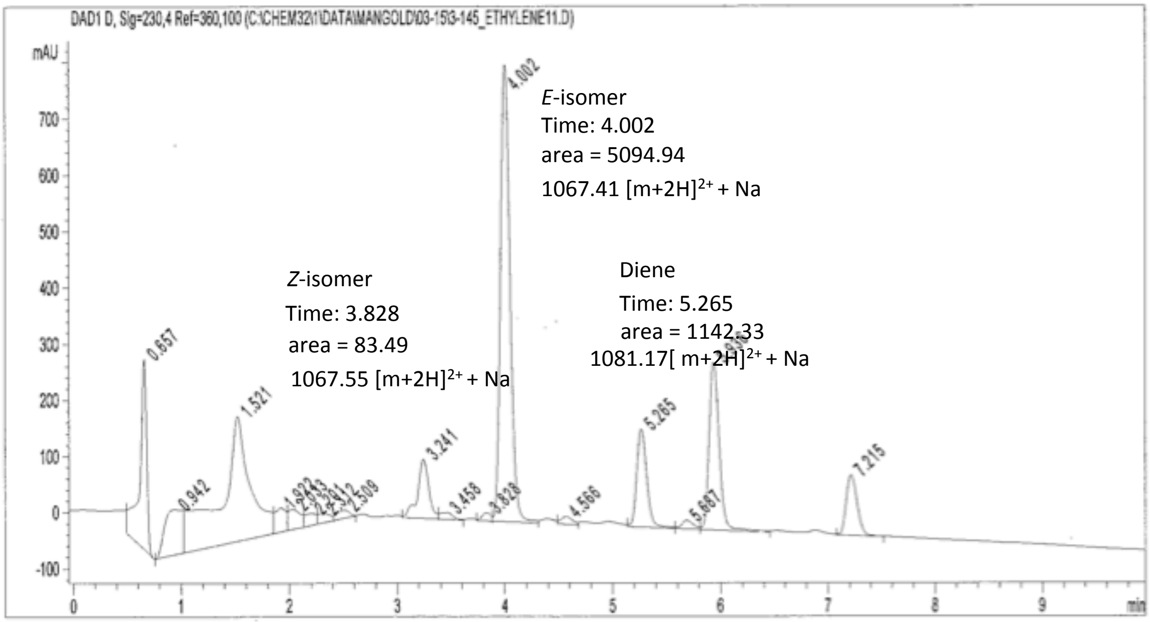

Peak	Start (min)	R_t (min)	End (min)	Height (mAu)	Area (mAu*S)	Area%
1	0.491	0.657	0.754	338.71182	1543.24329	
2	0.754	0.942	1.020	80.09177	905.25452	
3	1.020	1.521	1.849	222.35681	4322.25391	
4	1.849	1.922	1.979	43.20468	306.77618	
5	1.979	2.033	2.131	35.63584	281.65182	
6	2.131	2.201	2.260	21.46295	159.15390	
7	2.260	2.312	2.405	16.39495	124.08040	
8	2.405	2.509	2.612	14.84283	120.20170	
9	3.045	3.241	3.380	105.79904	768.17249	
10	3.380	3.458	3.609	11.90909	98.96686	
11 *Z* isomer	3.737	3.828	3.882	14.97733	83.49461	1.321
12 *E* isomer	3.882	4.002	4.307	813.08643	5094.94629	80.606
13	4.484	4.566	4.677	14.04178	103.66424	
14 Diene	5.136	5.265	5.577	175.19185	1142.33435	18.073
15	5.577	5.687	5.810	15.50419	115.46684	
16	5.810	5.936	6.458	298.65598	1950.84619	
17	7.077	7.215	7.518	107.71246	763.00409	

Figure S17: LCMS to assess the conversion and olefin selectivity for Z-selective ethenolysis on macrocyclic peptide **31**. The percentage of the E-isomer, Z-isomer, and resultant diene from ethenolysis was calculated from automatic integrations of each peak area. Column conditions: 5-95% acetonitrile:H_2O + 0.1% AcOH

Percentage enrichment of macrocycles 26-31 by Z-selective ethenolysis

Compound	Initial E:Z (%)	Final E:Z (%)
26	72:28	95:5
27	83:17	93:7
28	71:29	96:4
29	79:21	94:6
30	64:36	98:2
31	81:19	98:2

MALDI-TOF spectra of macrocycles 26-31

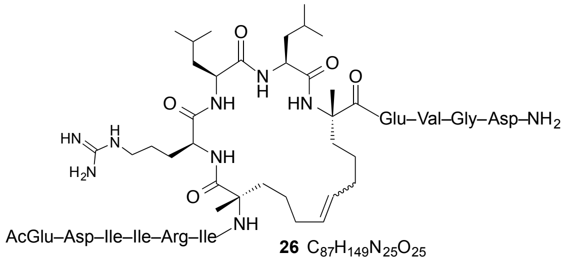

26 $C_{87}H_{149}N_{25}O_{25}$
1944.1200

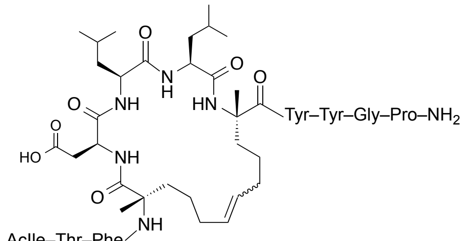

27 $C_{76}H_{109}N_{13}O_{18}$
1492.7810

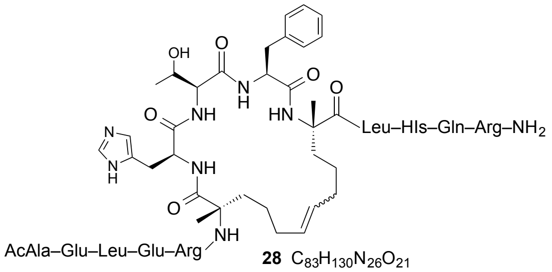

28 $C_{83}H_{130}N_{26}O_{21}$
1826.9904

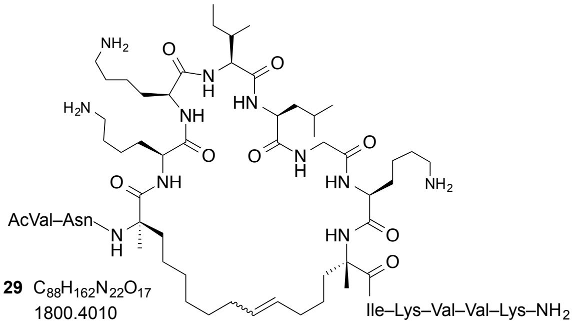

29 $C_{88}H_{162}N_{22}O_{17}$
1800.4010

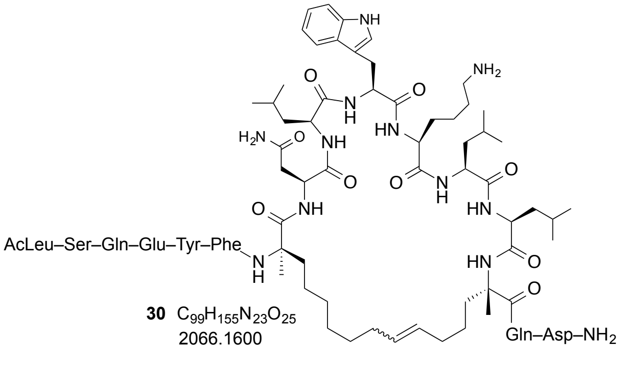

30 $C_{99}H_{155}N_{23}O_{25}$
2066.1600

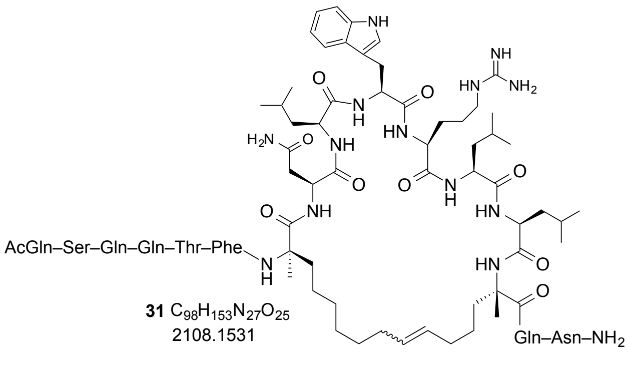

31 $C_{98}H_{153}N_{27}O_{25}$
2108.1531

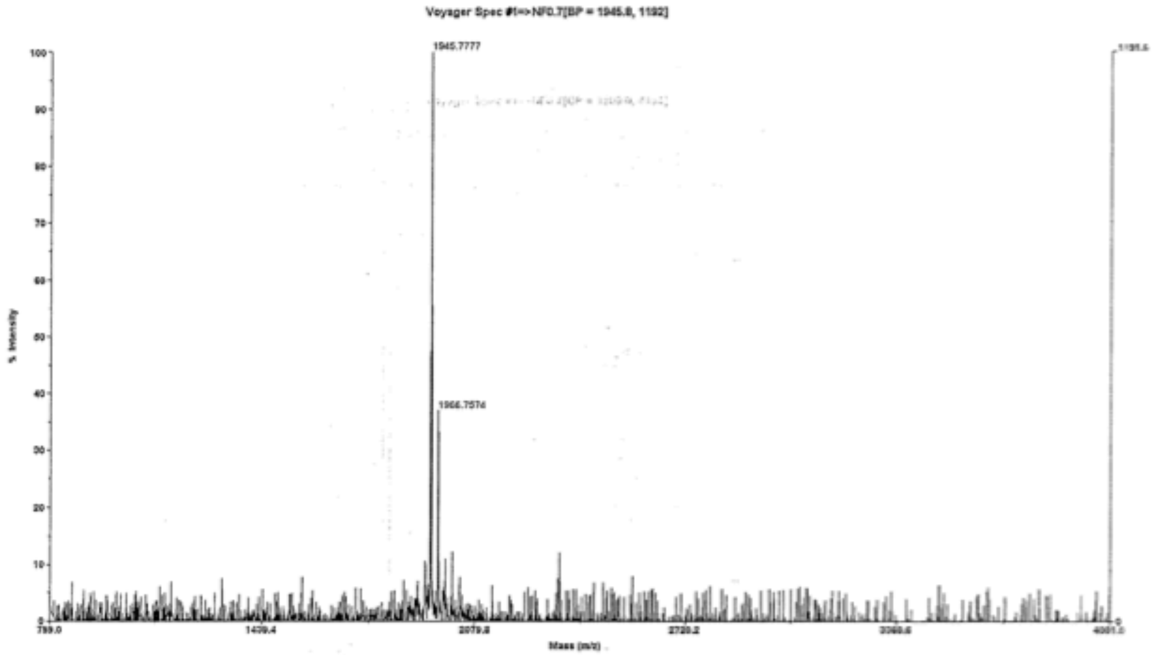

Figure S18: MALDI-TOF of purified peptide **26**. Indicated masses correspond to 1945.7777 [M+H]+ and 1966.7574 [m+Na]+ for the product of RCM.

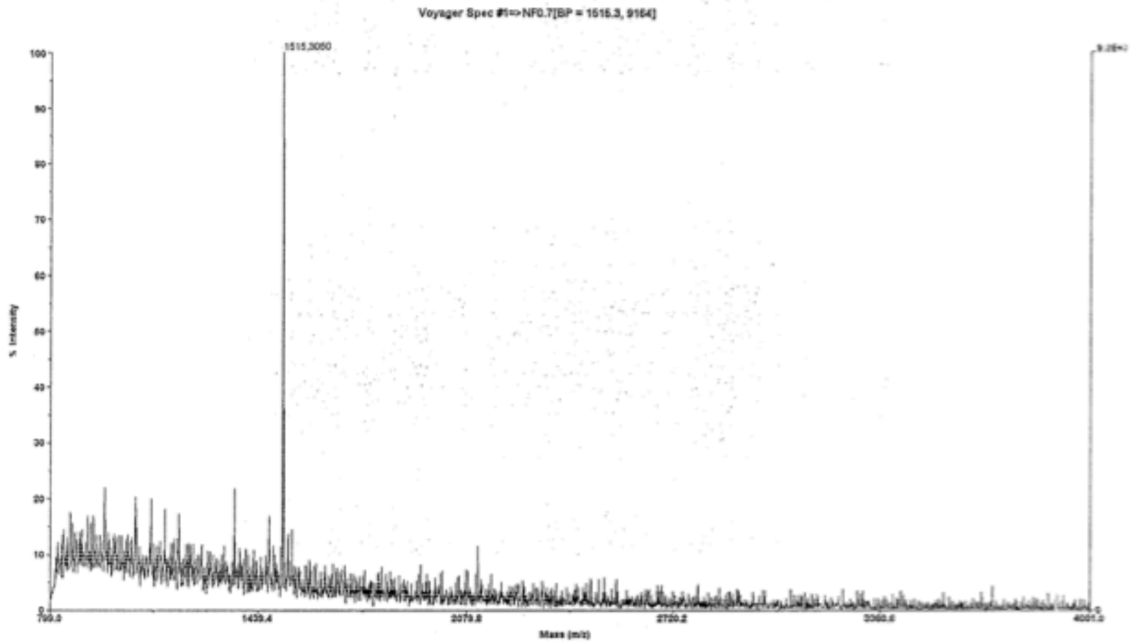

Figure S19: MALDI-TOF of purified peptide **27**. Indicated mass corresponds to 1515.3050 [M+Na]+ for the product of RCM.

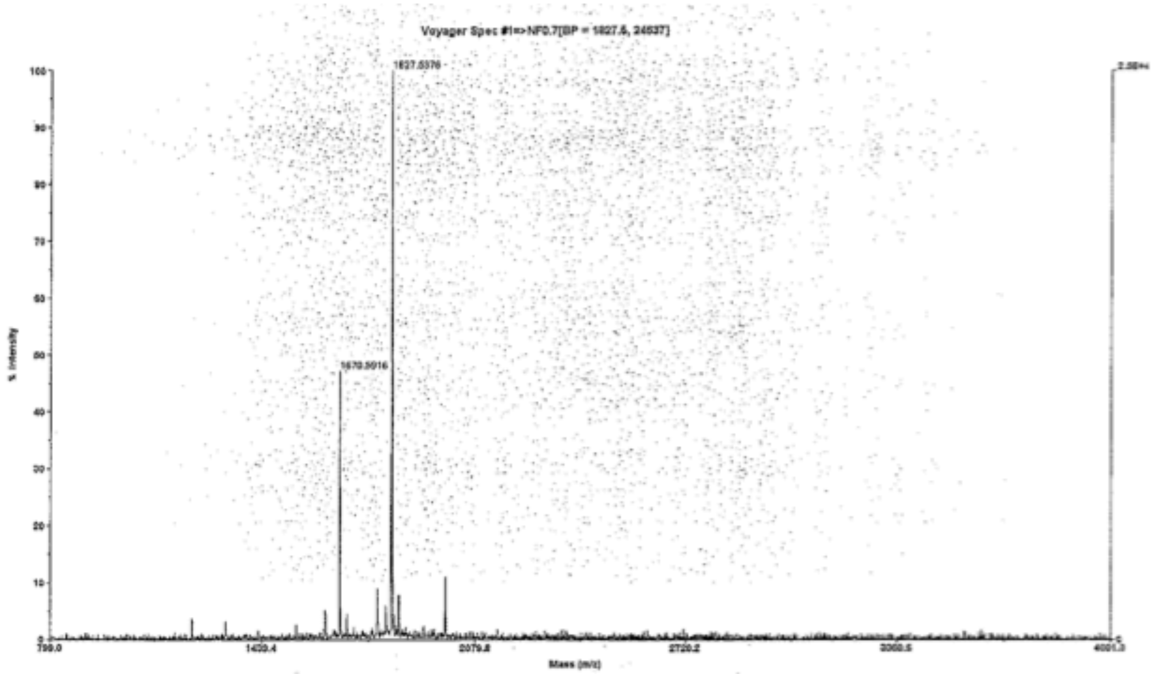

Figure S20: MALDI-TOF of purified peptide **28**. Indicated masses correspond to product 1827.5376 $[M+H]^+$ and unknown mass 1670.5916 for the product of RCM.

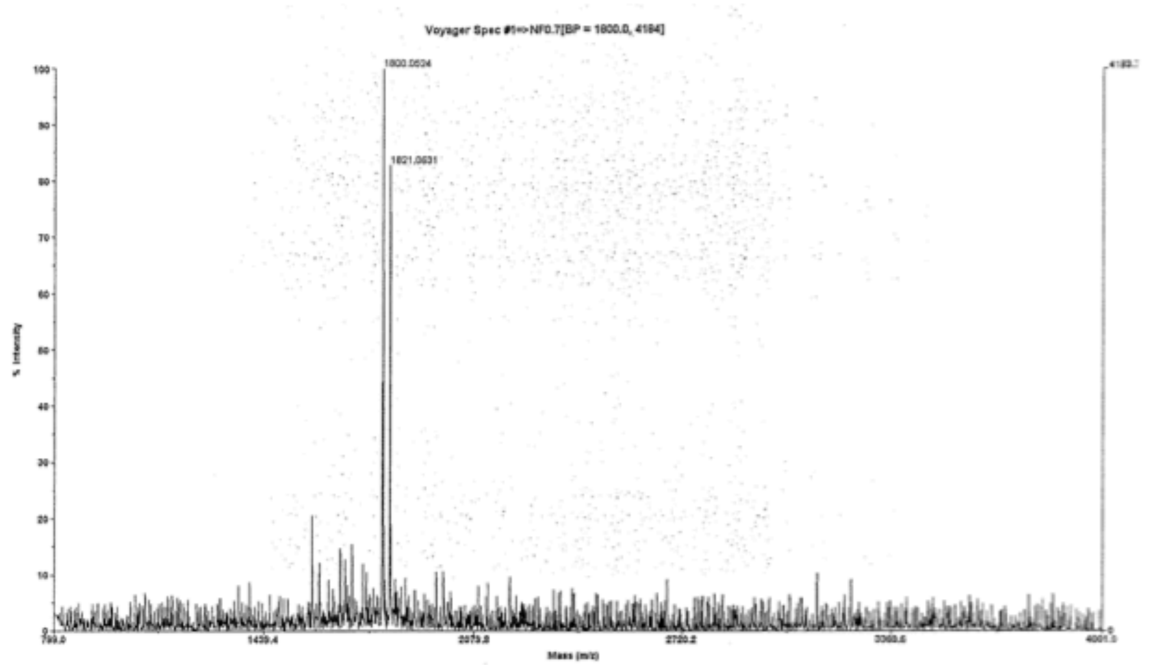

Figure S21: MALDI-TOF of purified peptide **29**. Indicated masses correspond to 1800.0524 $[M+H]^+$ and 1821.0631 $[M+Na]^+$ for the product of RCM.

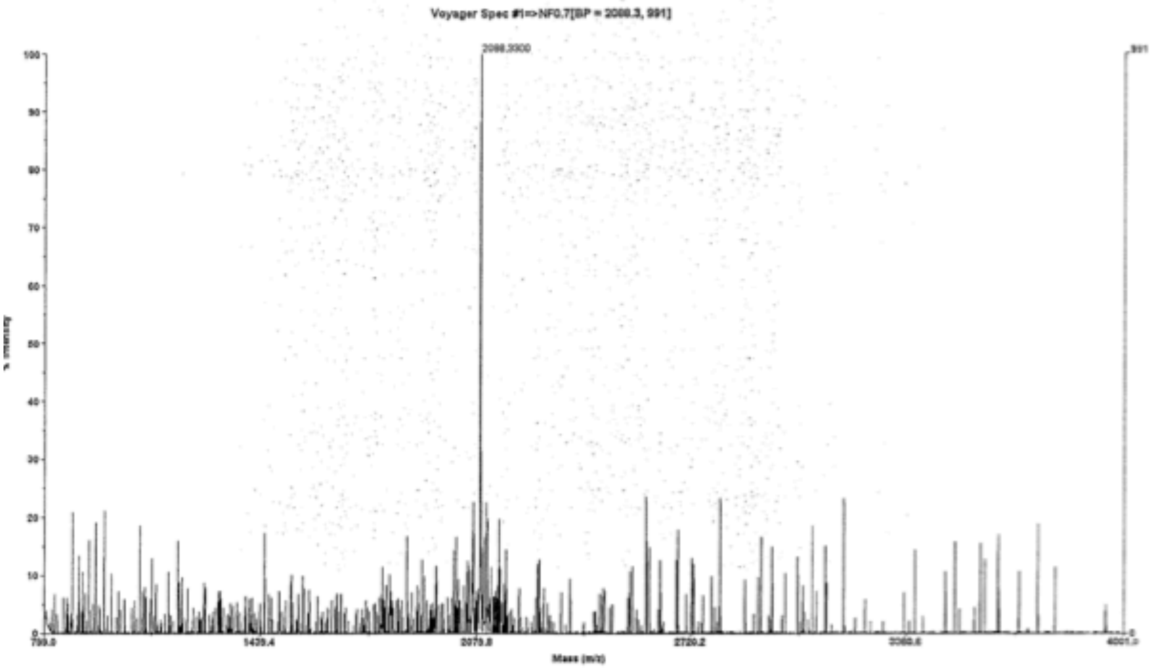

Figure S22: MALDI-TOF of purified peptide **30**. Indicated mass corresponds to 2088.3300 $[M+Na]^+$ for the product of RCM.

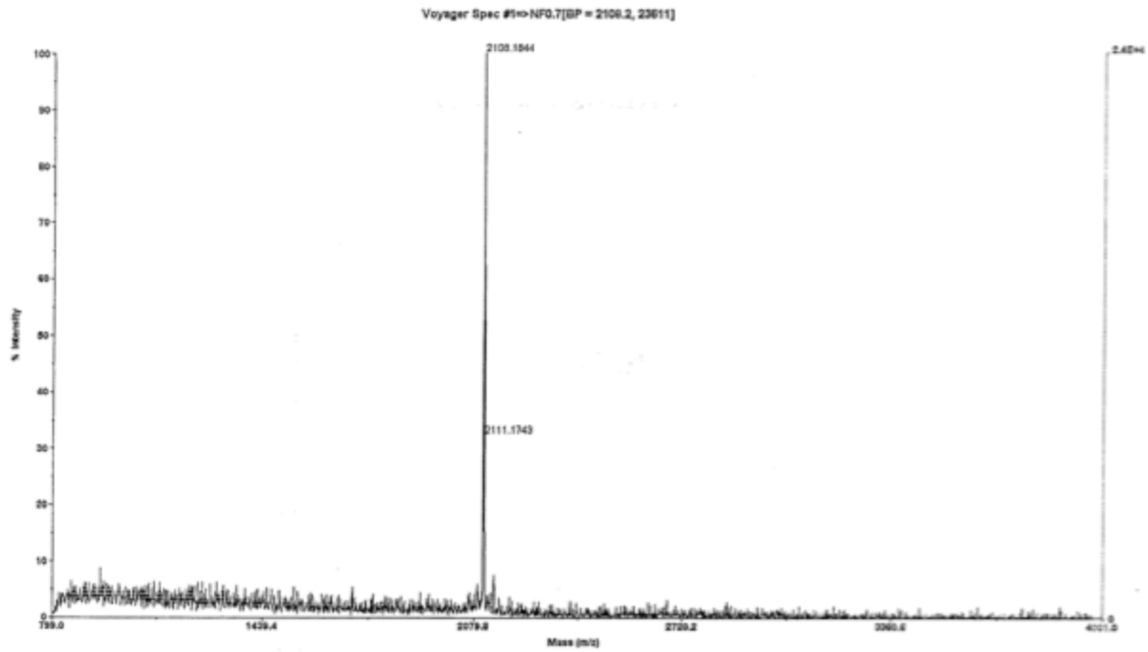

Figure S23: MALDI-TOF of purified peptide **31**. Indicated mass corresponds to 2108.1844 $[M+H]^+$ for the product of RCM.

HPLC traces of macrocycles 27 and 29

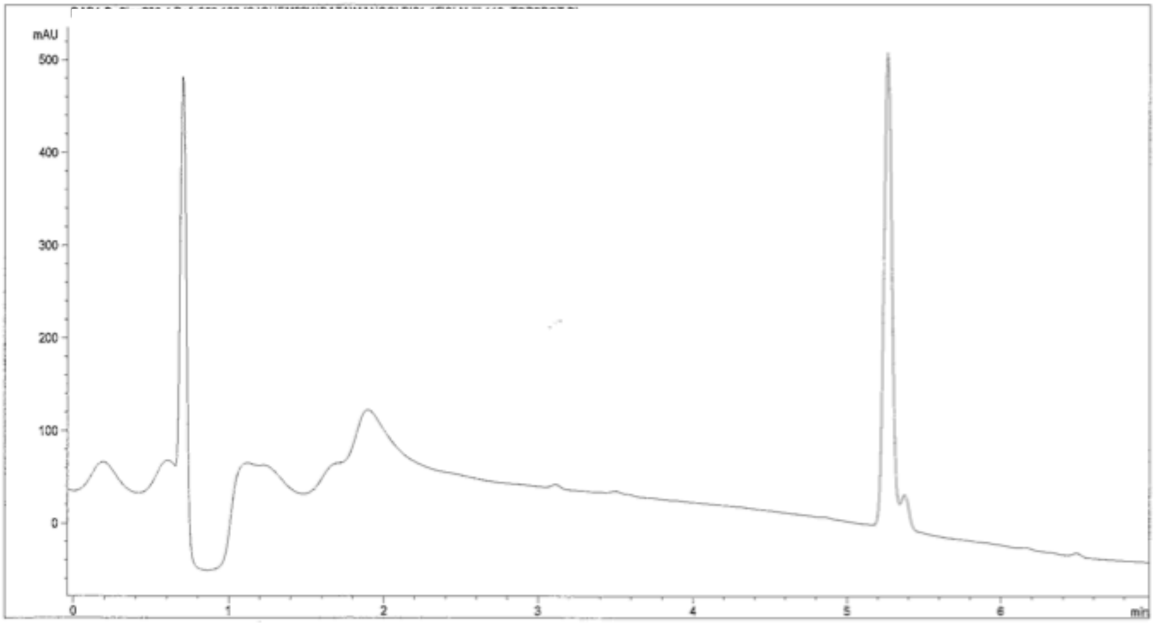

Figure S24: Analytical HPLC of peptide Z-**27** (Rt 5.221 min). Column conditions: 5 to 85% acetonitrile:H_2O + 0.1% TFA

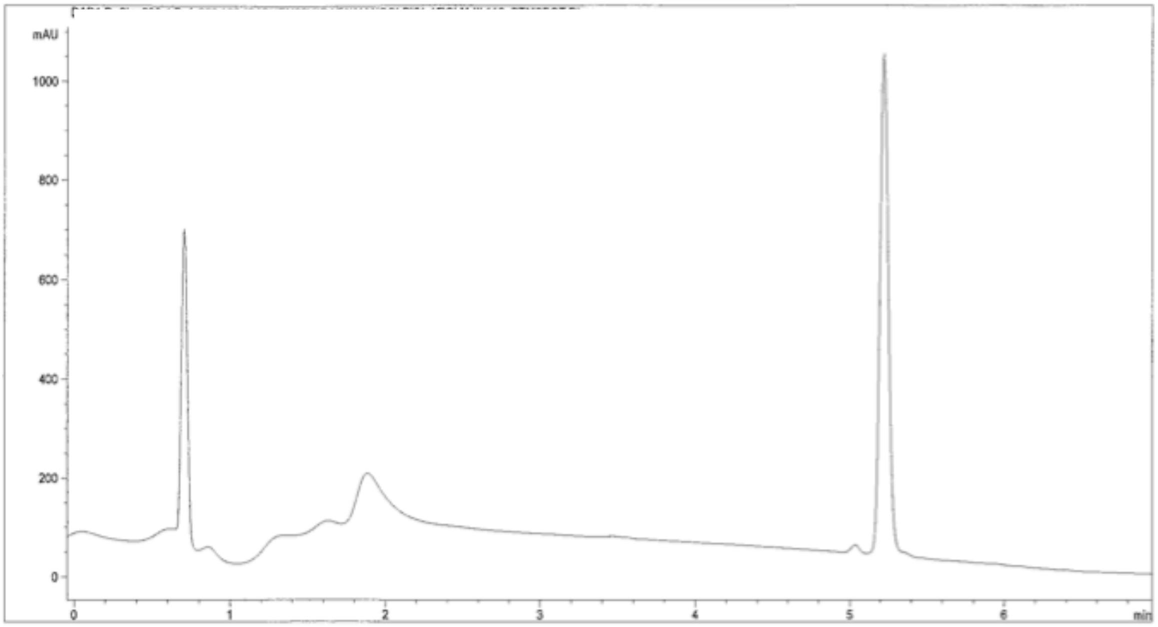

Figure S25: Analytical HPLC of peptide E-**27** (Rt 5.280 min). Column conditions: 5 to 85% acetonitrile:H_2O + 0.1% TFA

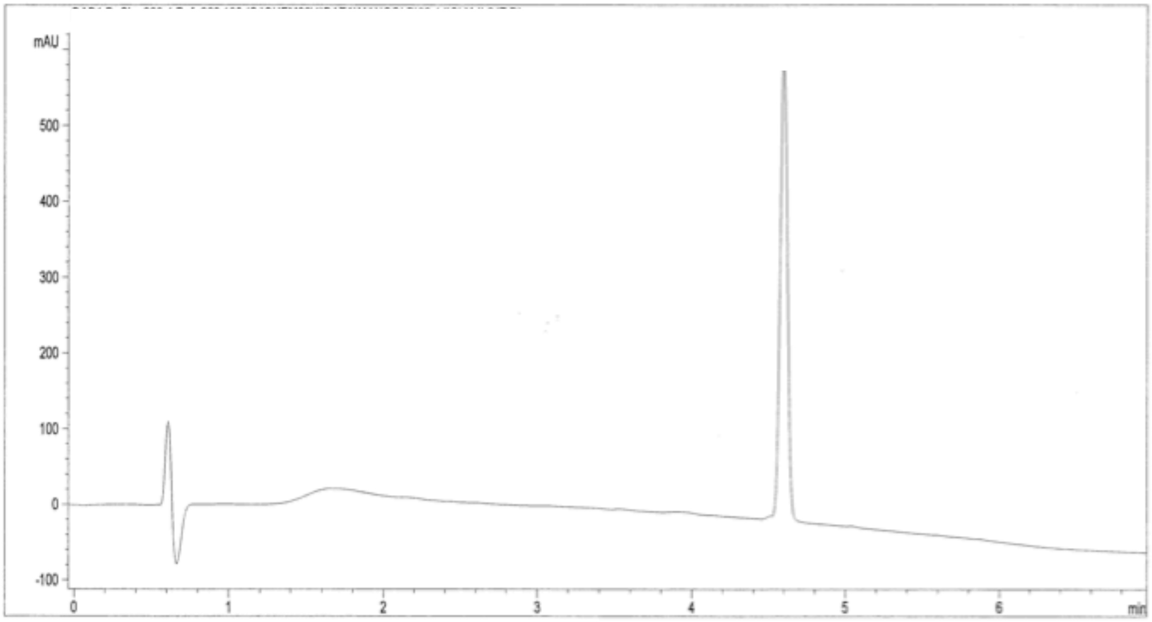

Figure S26: Analytical HPLC of peptide Z-**29** (Rt 4.630 min). Column conditions. 15 to 80% acetonitrile:H_2O + 0.1% TFA

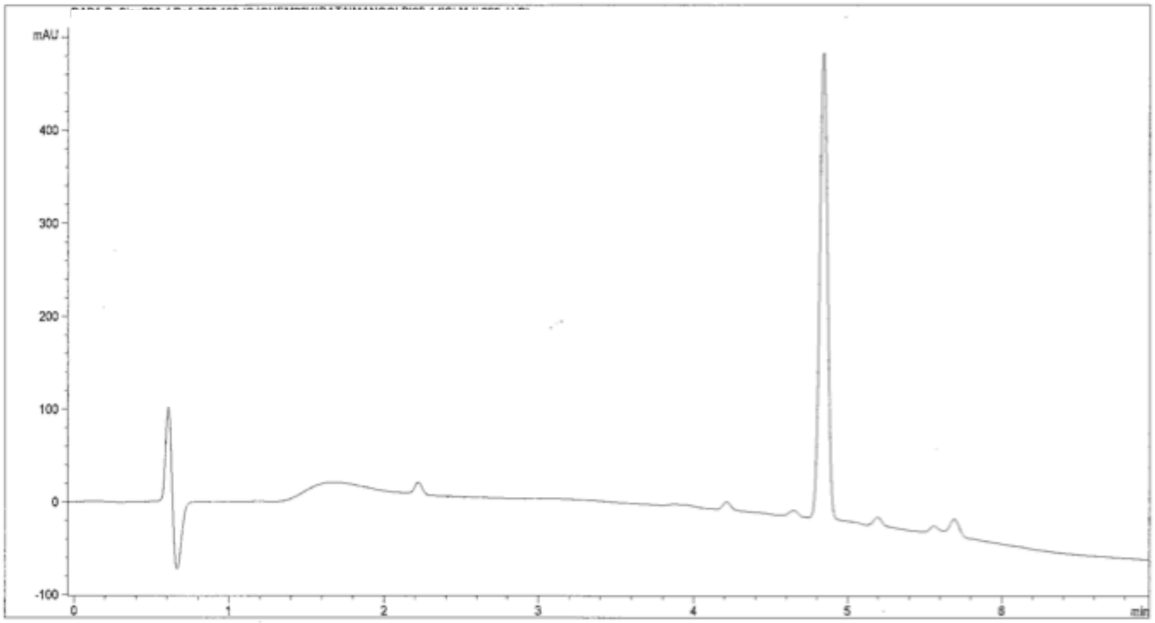

Figure S27: Analytical HPLC of peptide E-**29** (Rt 4.843 min). Column conditions: 15 to 80% acetonitrile:H_2O + 0.1% TFA

Circular Dichroism of α-helical peptides

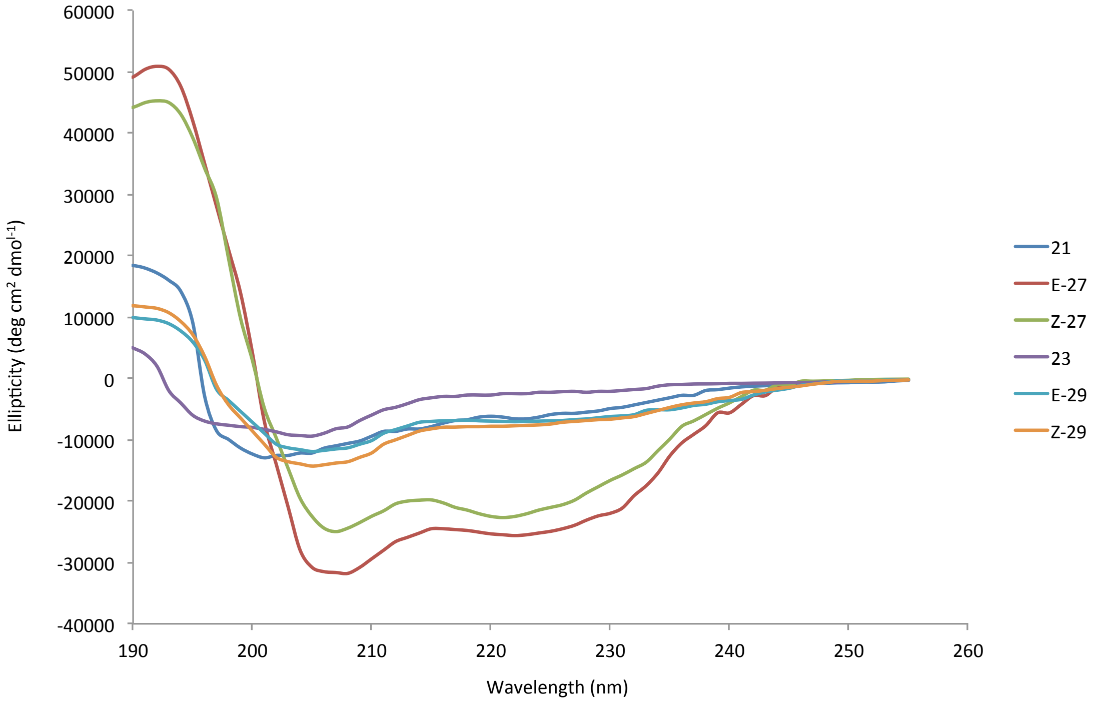

Figure S28: Circular dichroism of peptides before (**21** and **23**) and after (**27** and **29**) RCM. Both *E* and *Z* olefin isomers of macrocycles **27** and **29** were examined for their α-helicity. Parameters: 190 to 255 nm; 1 nm step resolution, averaging time 1 sec, 20 ^{o}C.

**Compound**	**α-helicity**
21	20.8
E-27	80.9
Z-27	71.0
23	7.5
E-29	21.2
Z-29	23.1

NMR Spectra: ^{1}H NMR (500 MHz, $CDCl_3$) spectrum of compound **S1**

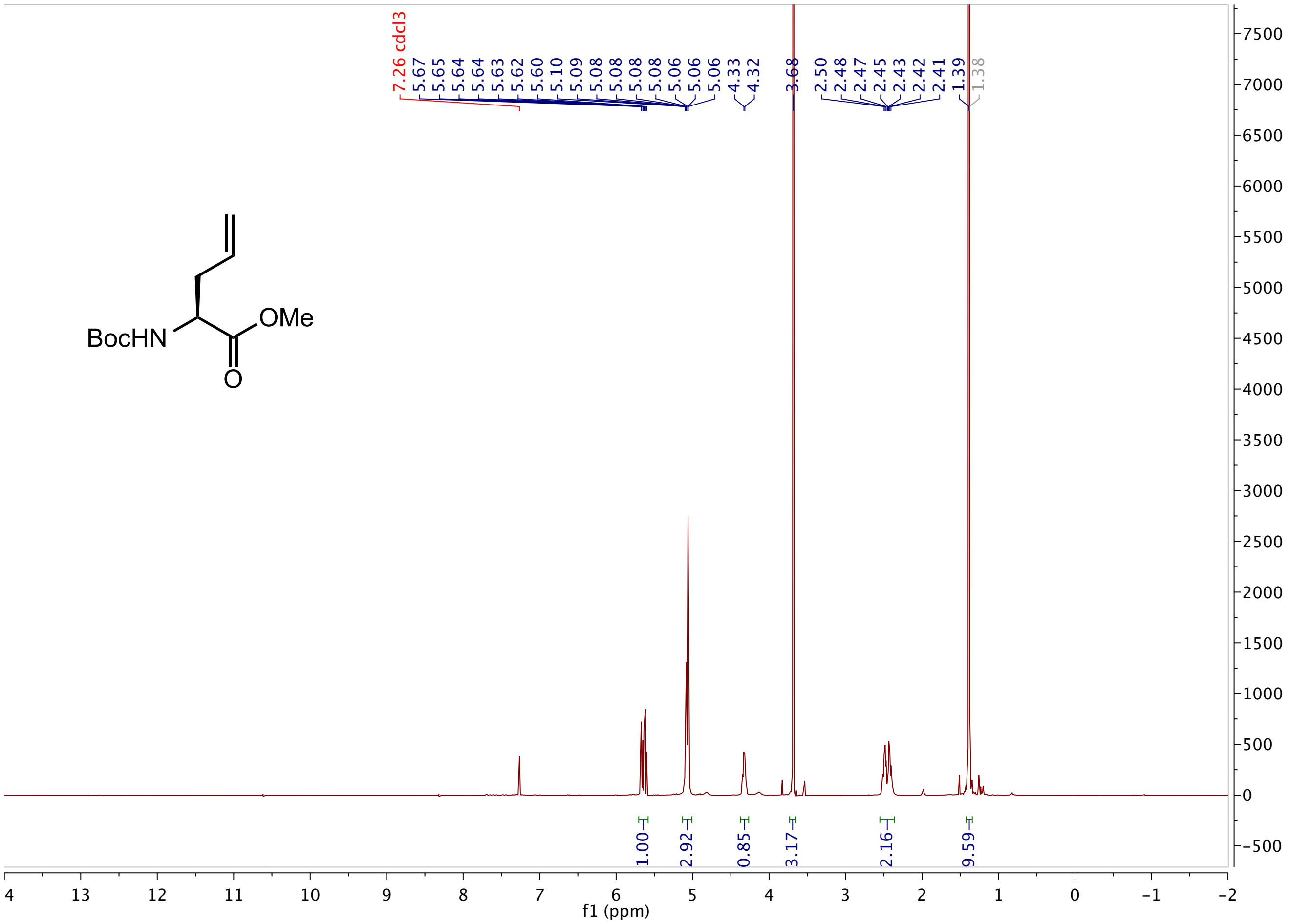

^{13}C NMR (126 MHz, $CDCl_3$) spectrum of compound **S1**

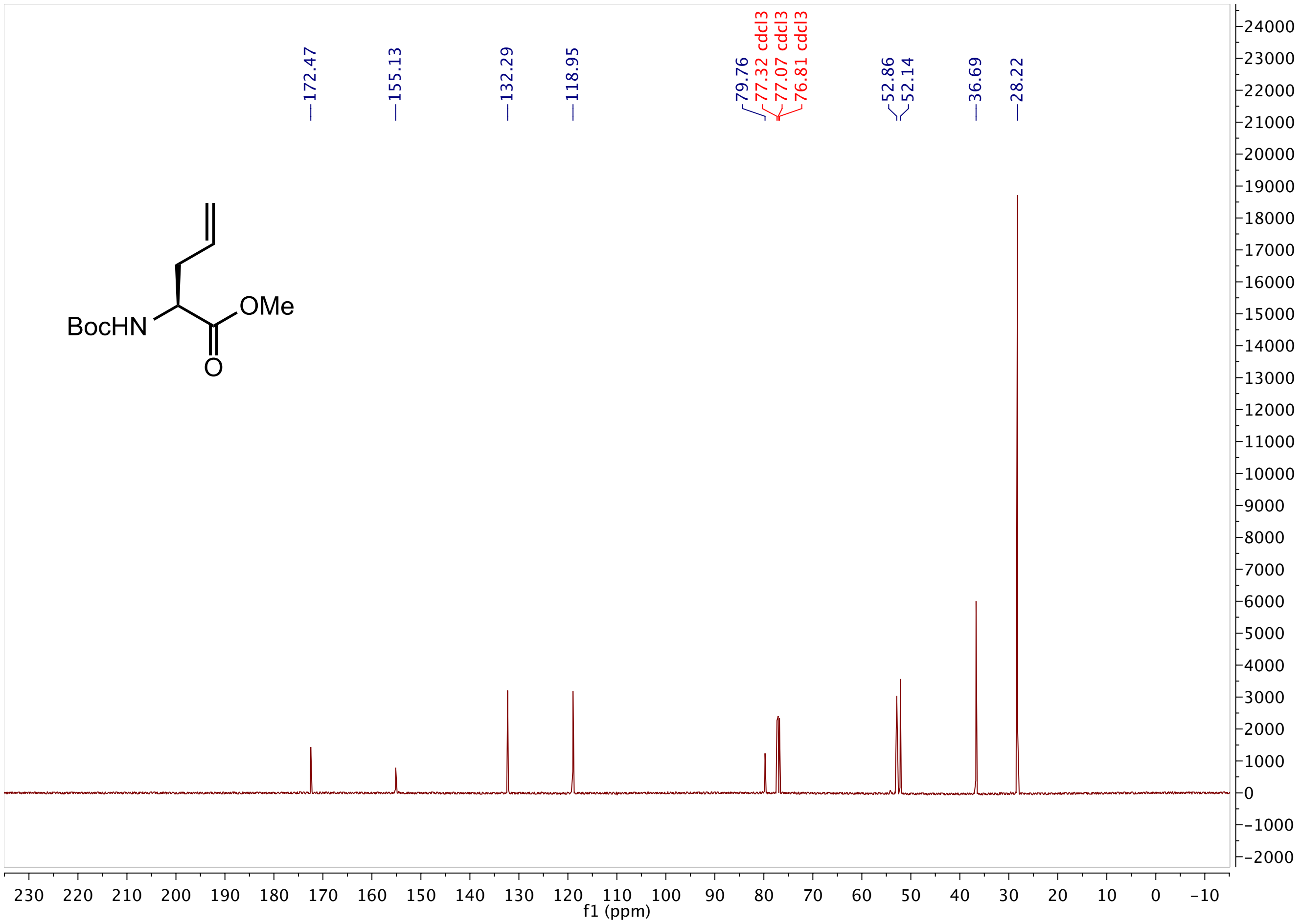

^{1}H NMR (500 MHz, $CDCl_3$) spectrum of compound **S2**

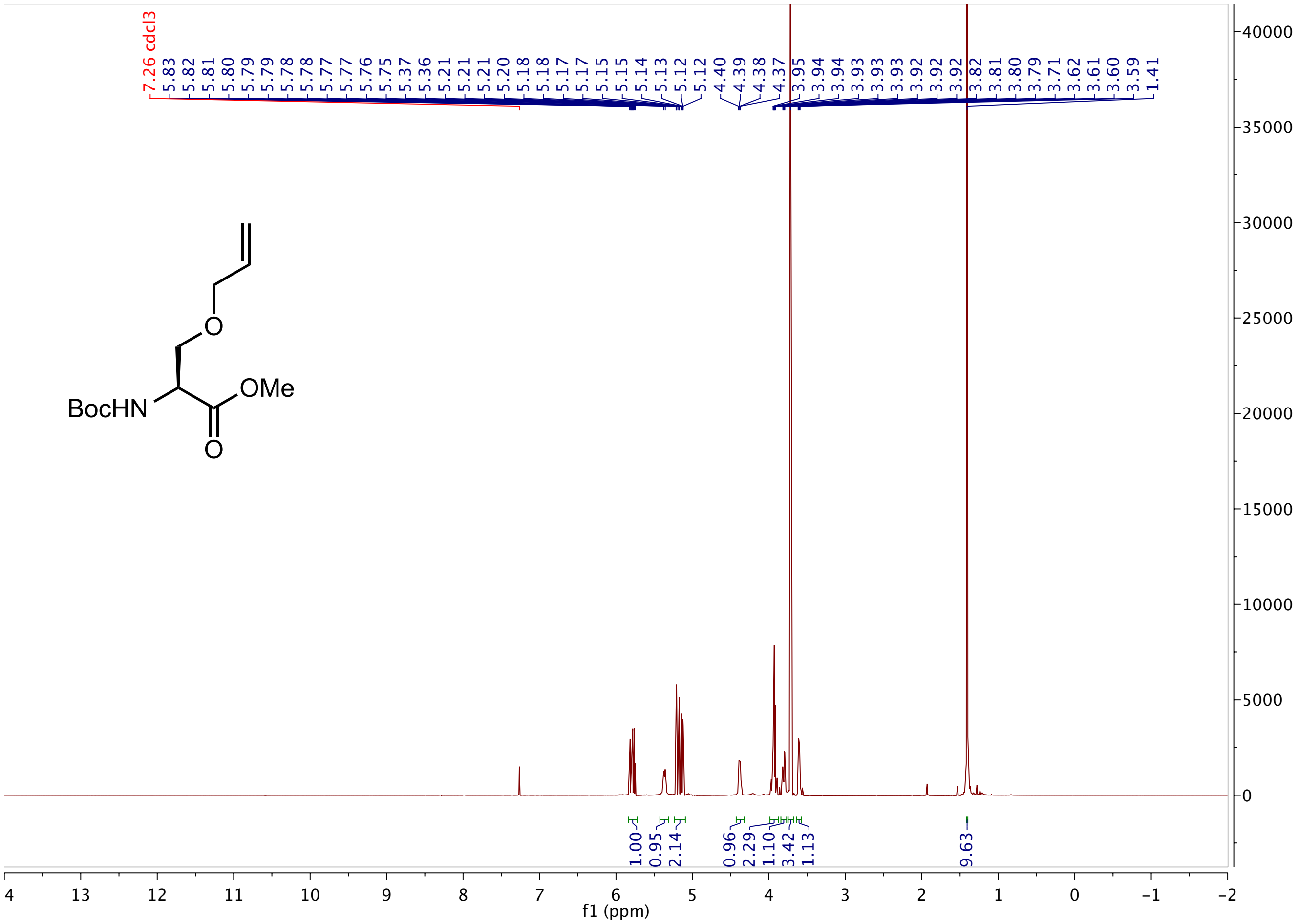

^{13}C NMR (126 MHz, $CDCl_3$) spectrum of compound **S2**

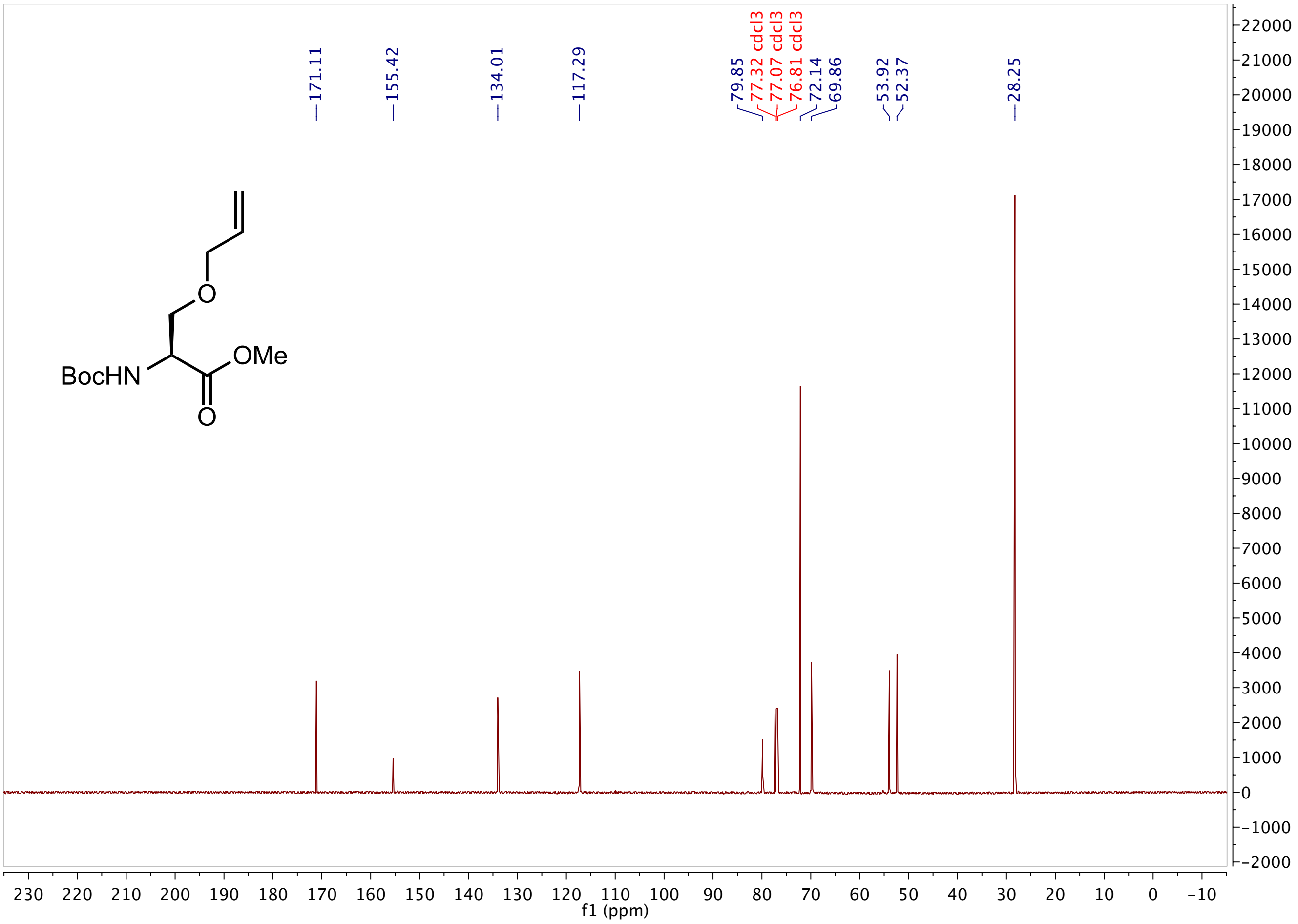

^{1}H NMR (500 MHz, $CDCl_3$) spectrum of compound **S3**

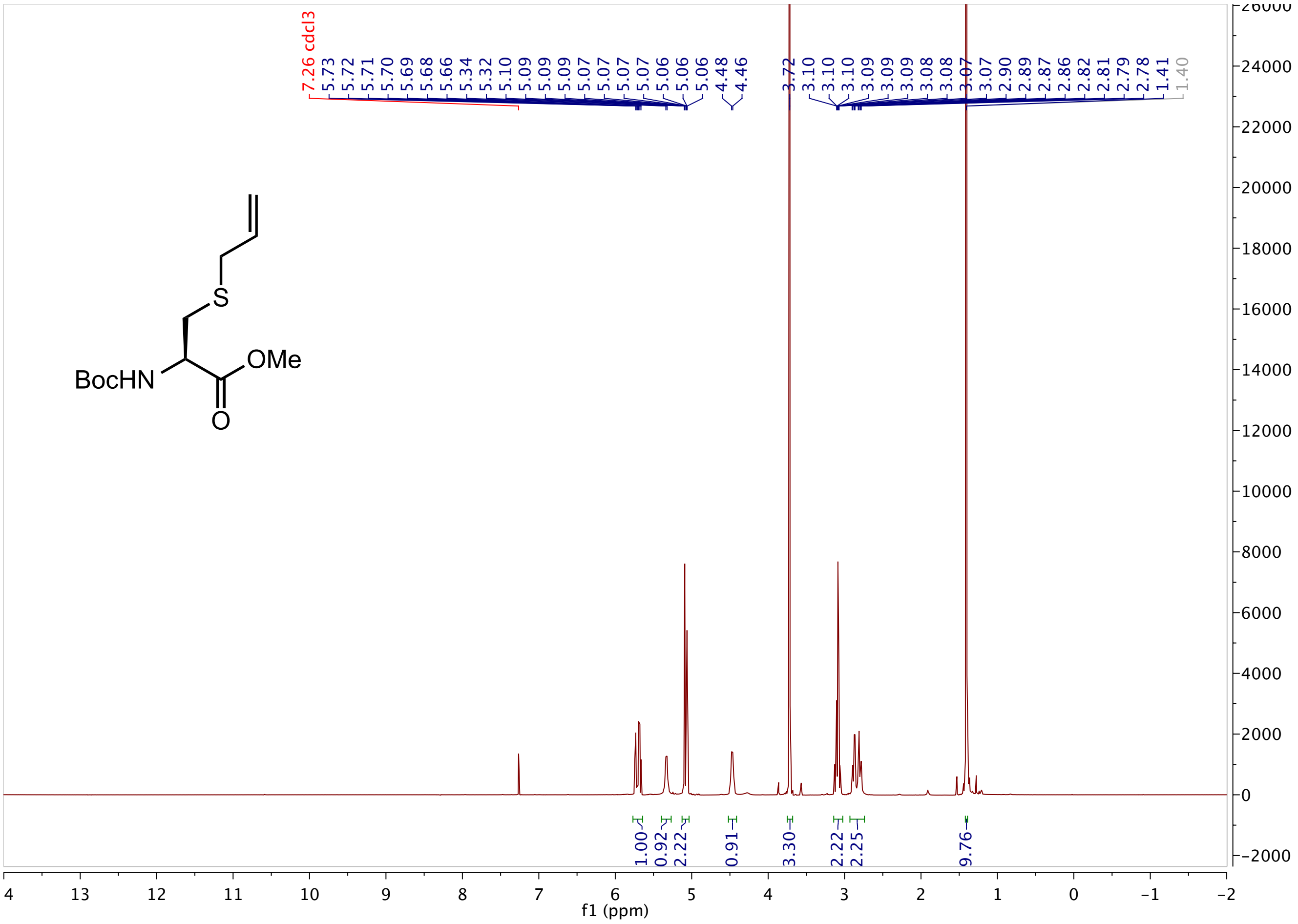

^{13}C NMR (126 MHz, $CDCl_3$) spectrum of compound **S3**

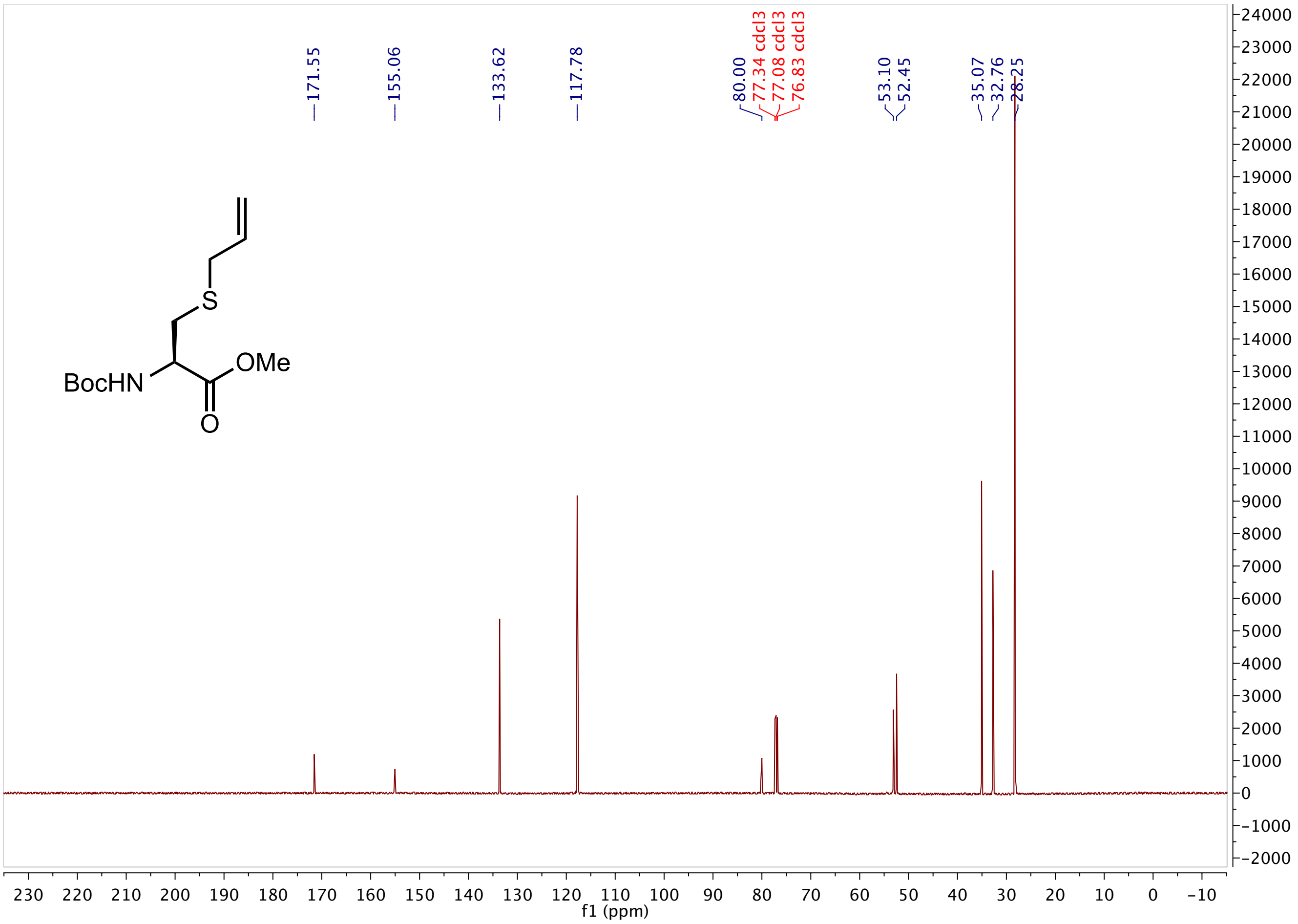

^{1}H NMR (500 MHz, $CDCl_3$) spectrum of compound **S4**

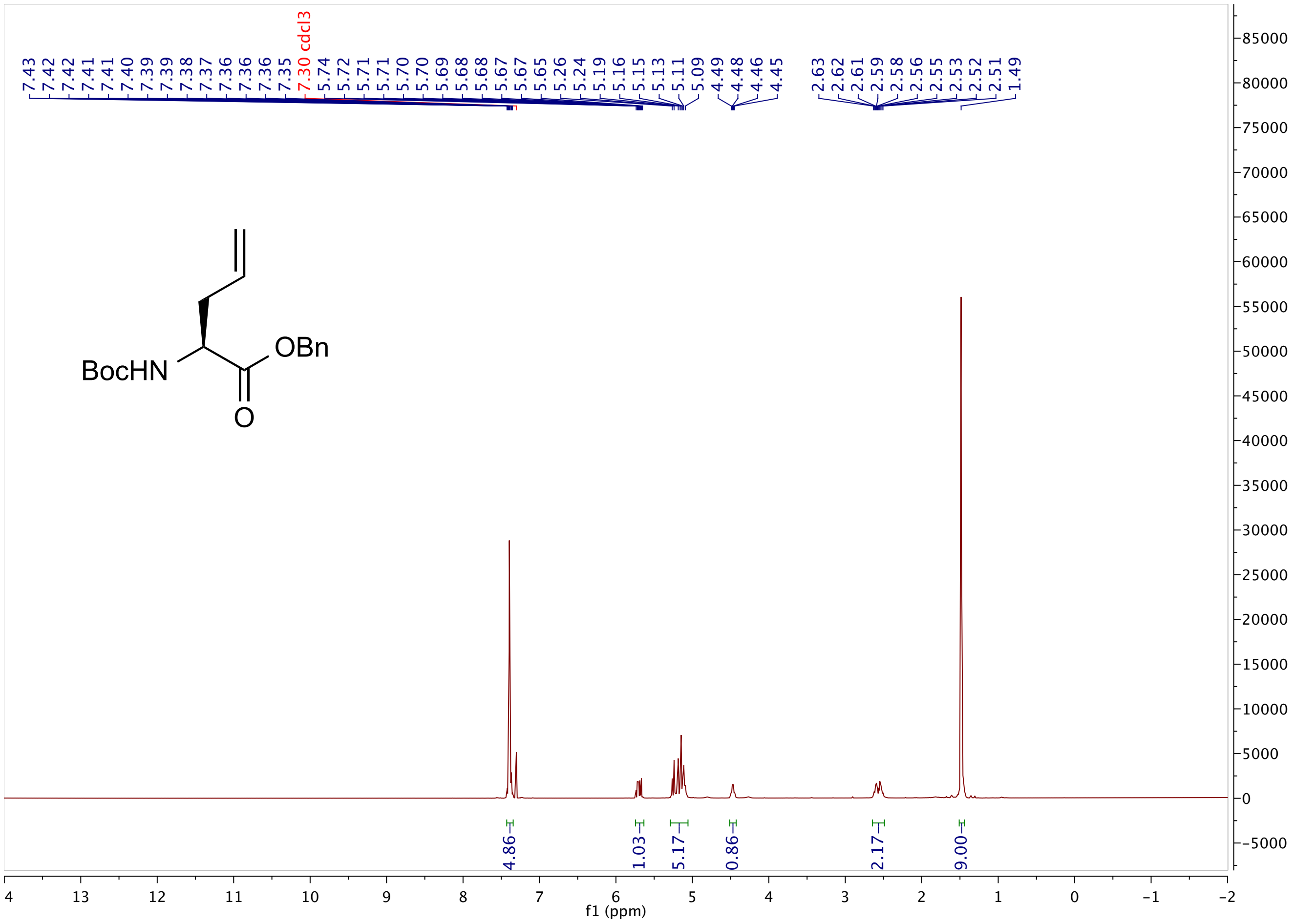

^{13}C NMR (126 MHz, $CDCl_3$) spectrum of compound **S4**

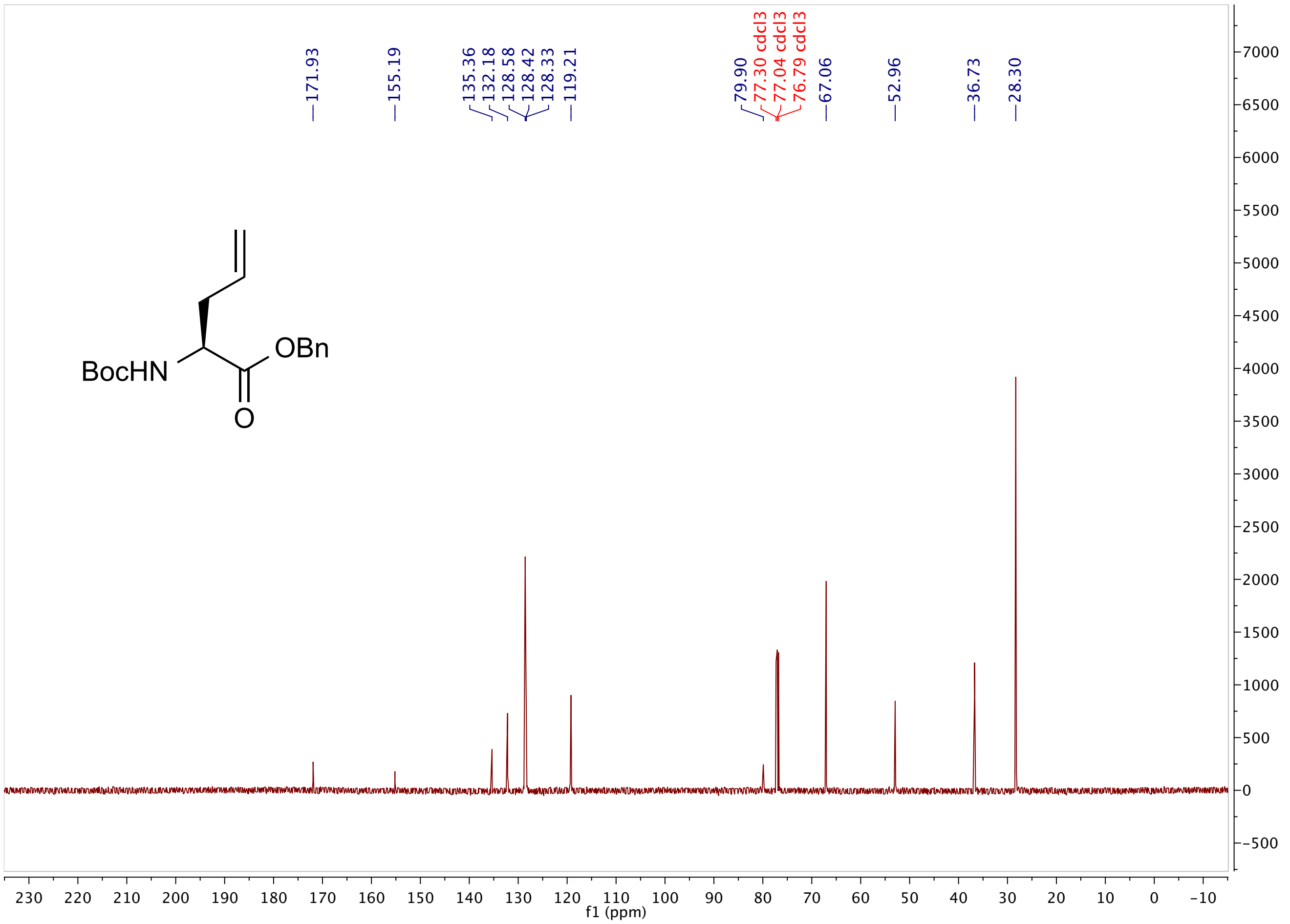

^{1}H NMR (500 MHz, $CDCl_3$) spectrum of compound **S5**

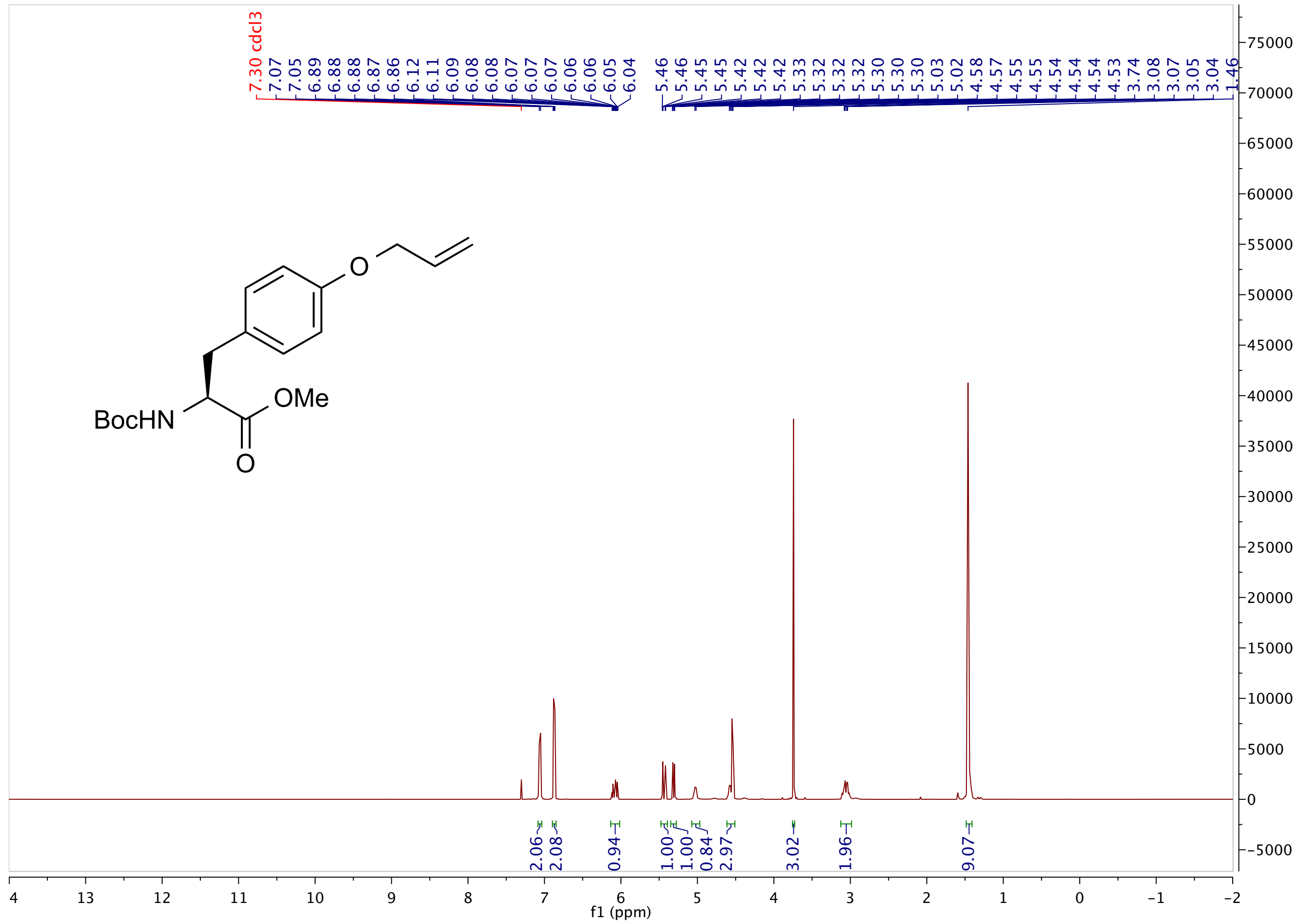

^{13}C NMR (126 MHz, $CDCl_3$) spectrum of compound **S5**

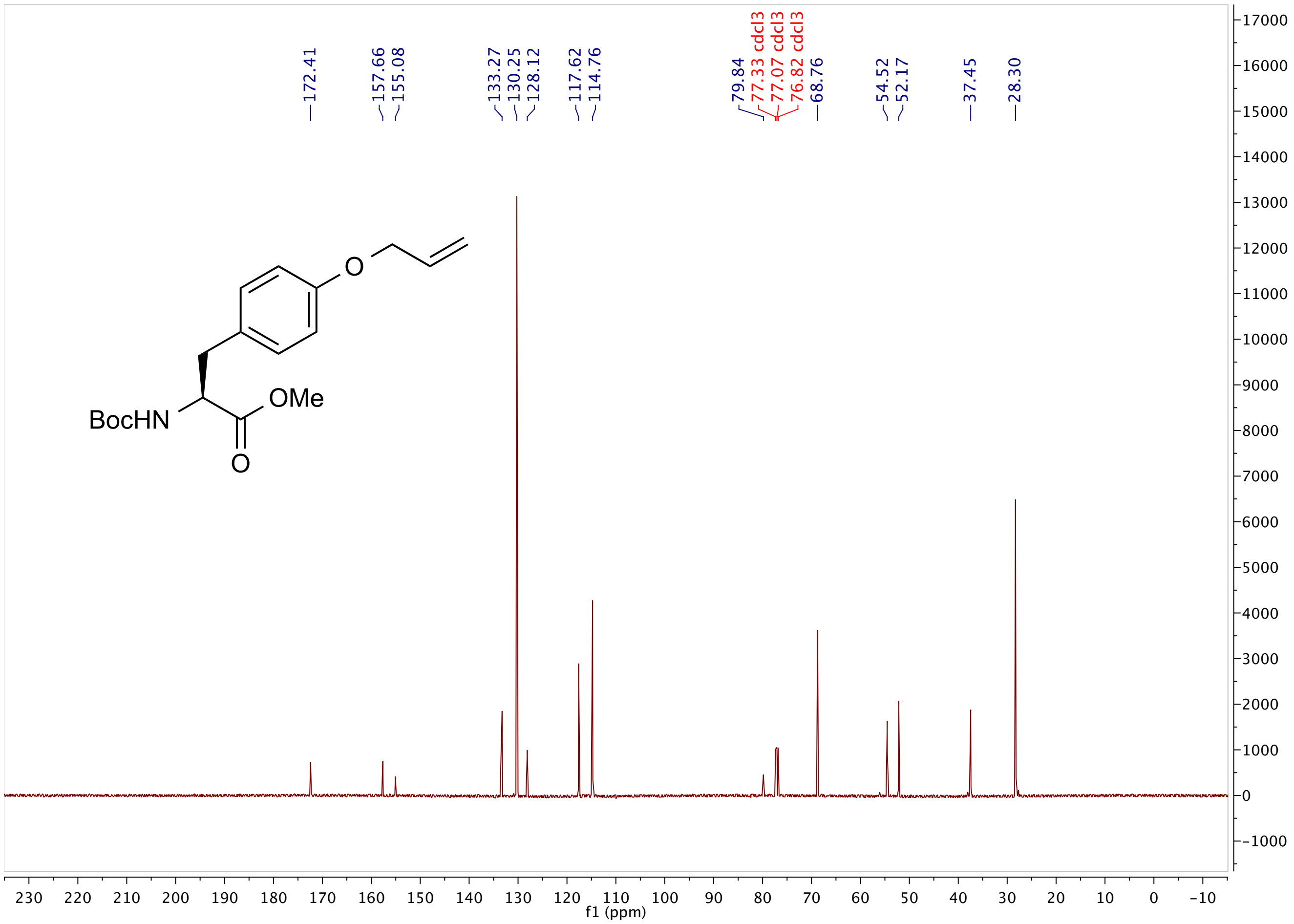

^{1}H NMR (500 MHz, $CDCl_3$) spectrum of compound **S6**

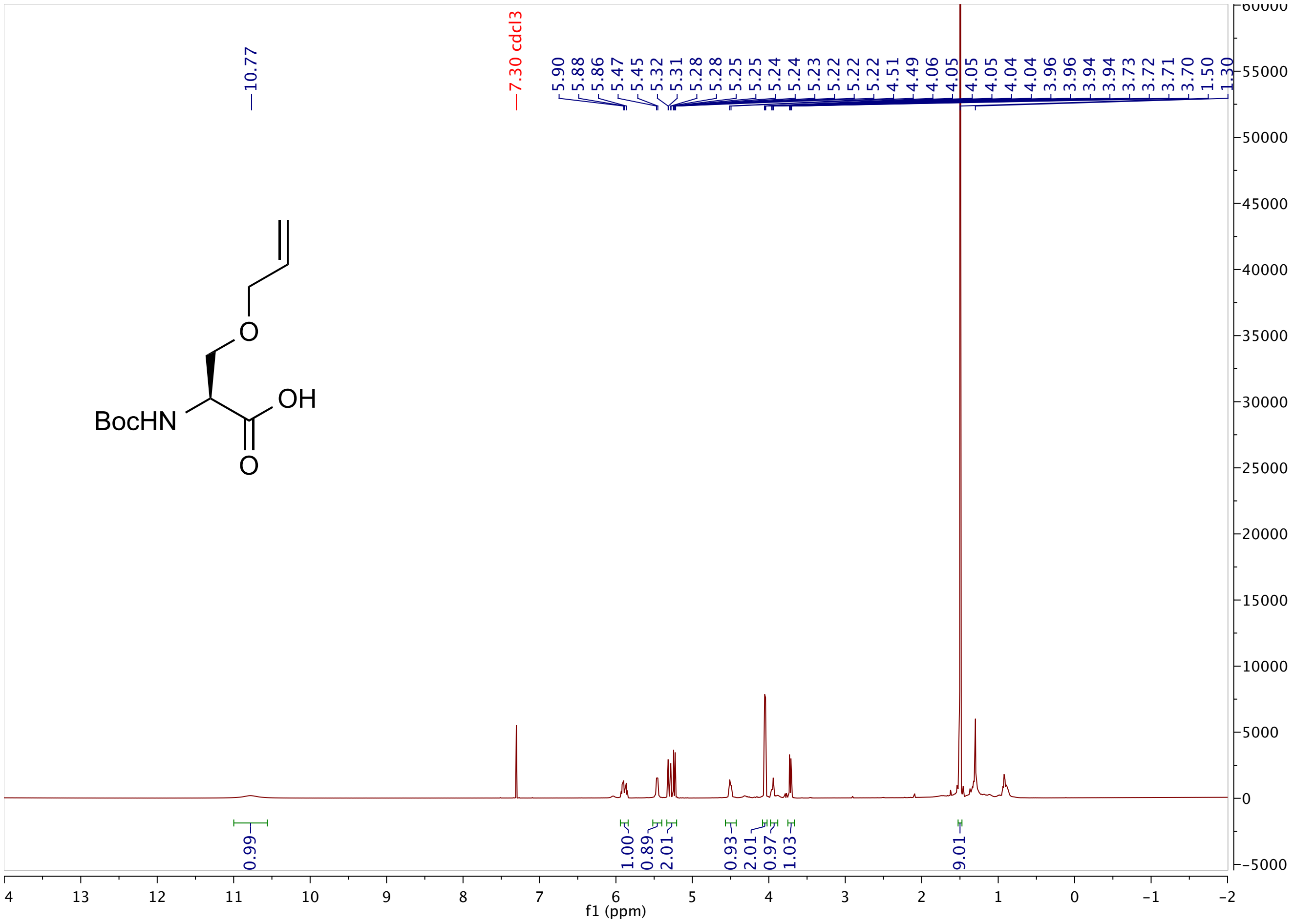

^{13}C NMR (126 MHz, $CDCl_3$) spectrum of compound **S6**

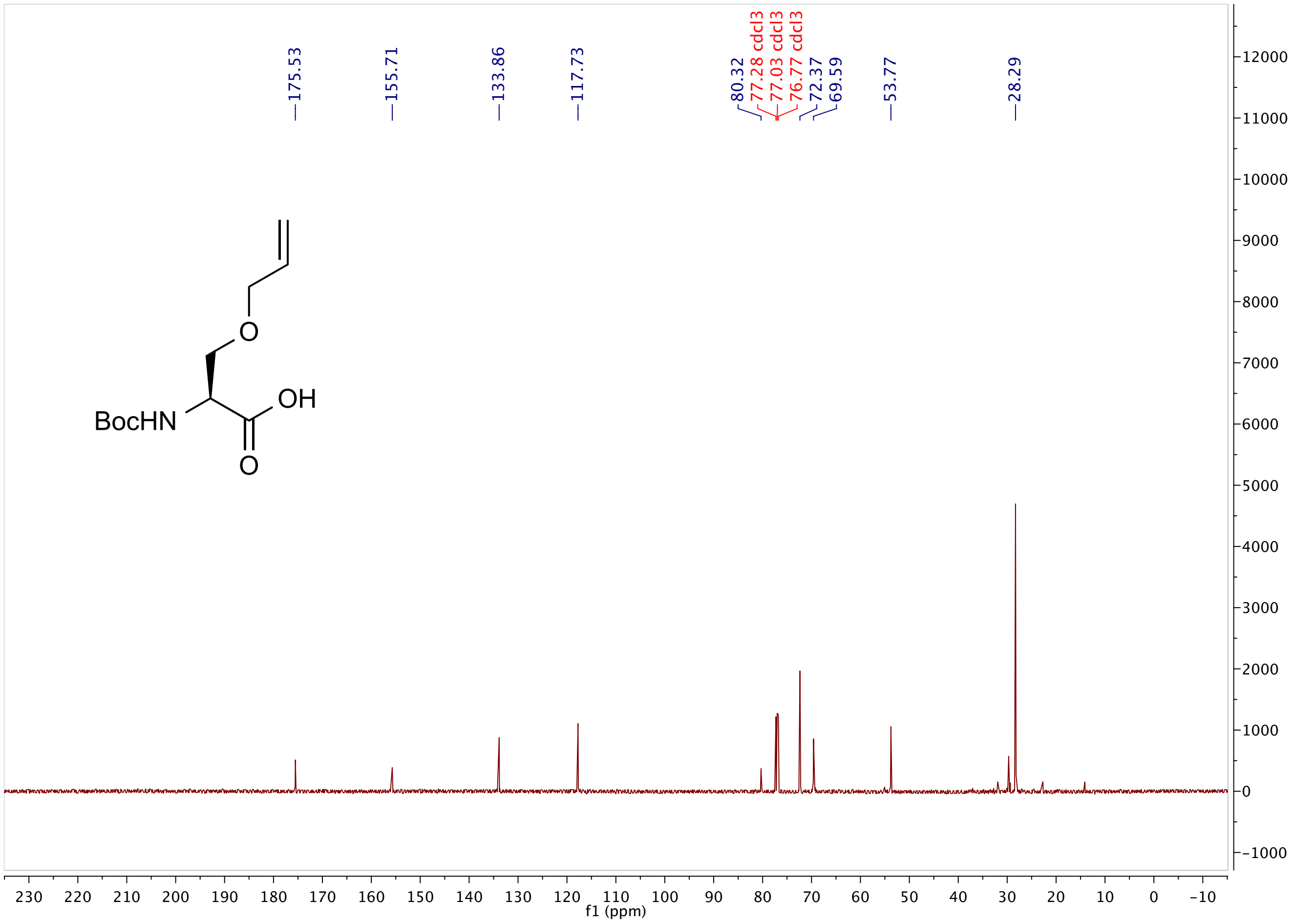

^{1}H NMR (500 MHz, $CDCl_3$) spectrum of compound **S7**

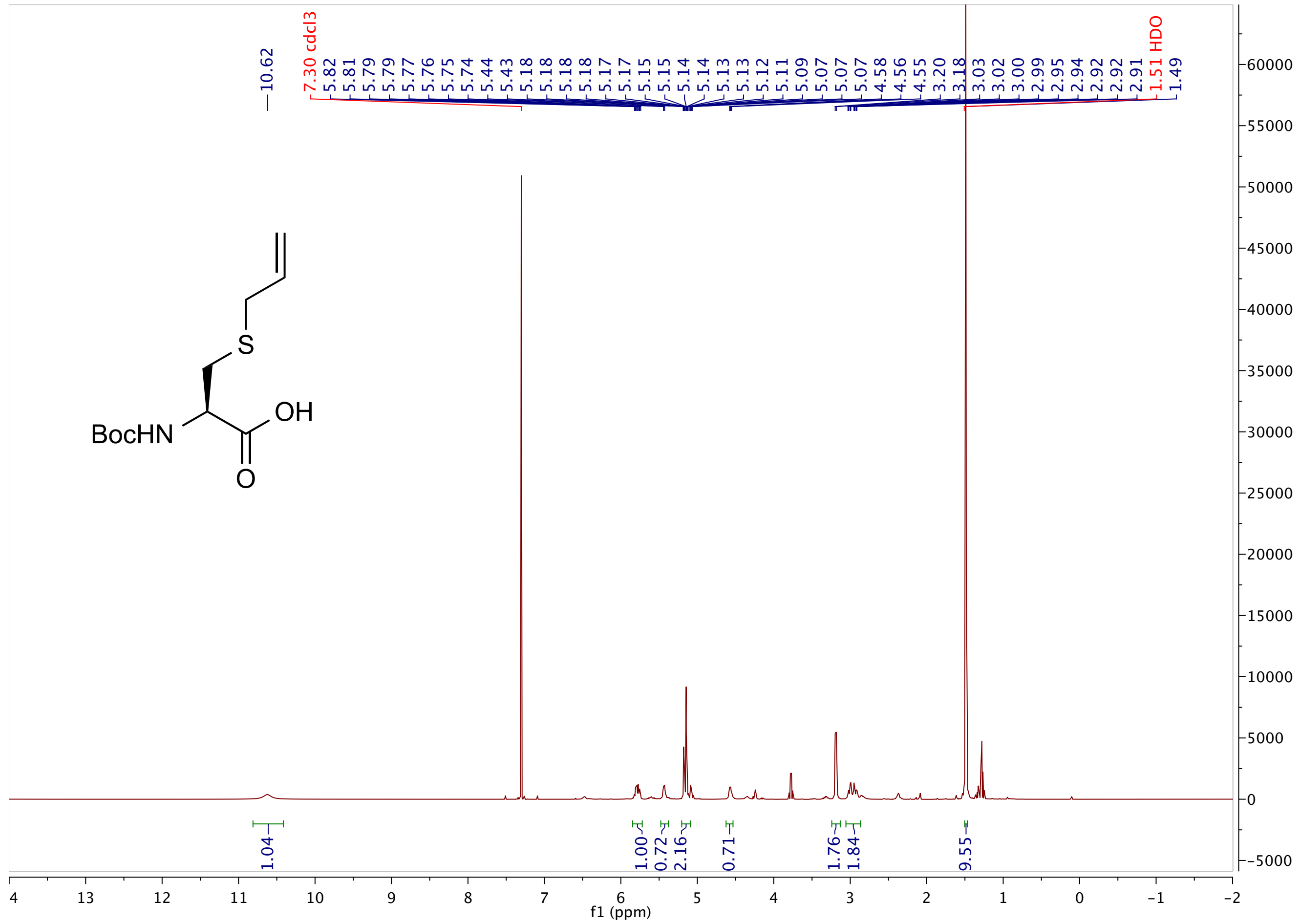

^{13}C NMR (126 MHz, $CDCl_3$) spectrum of compound **S7**

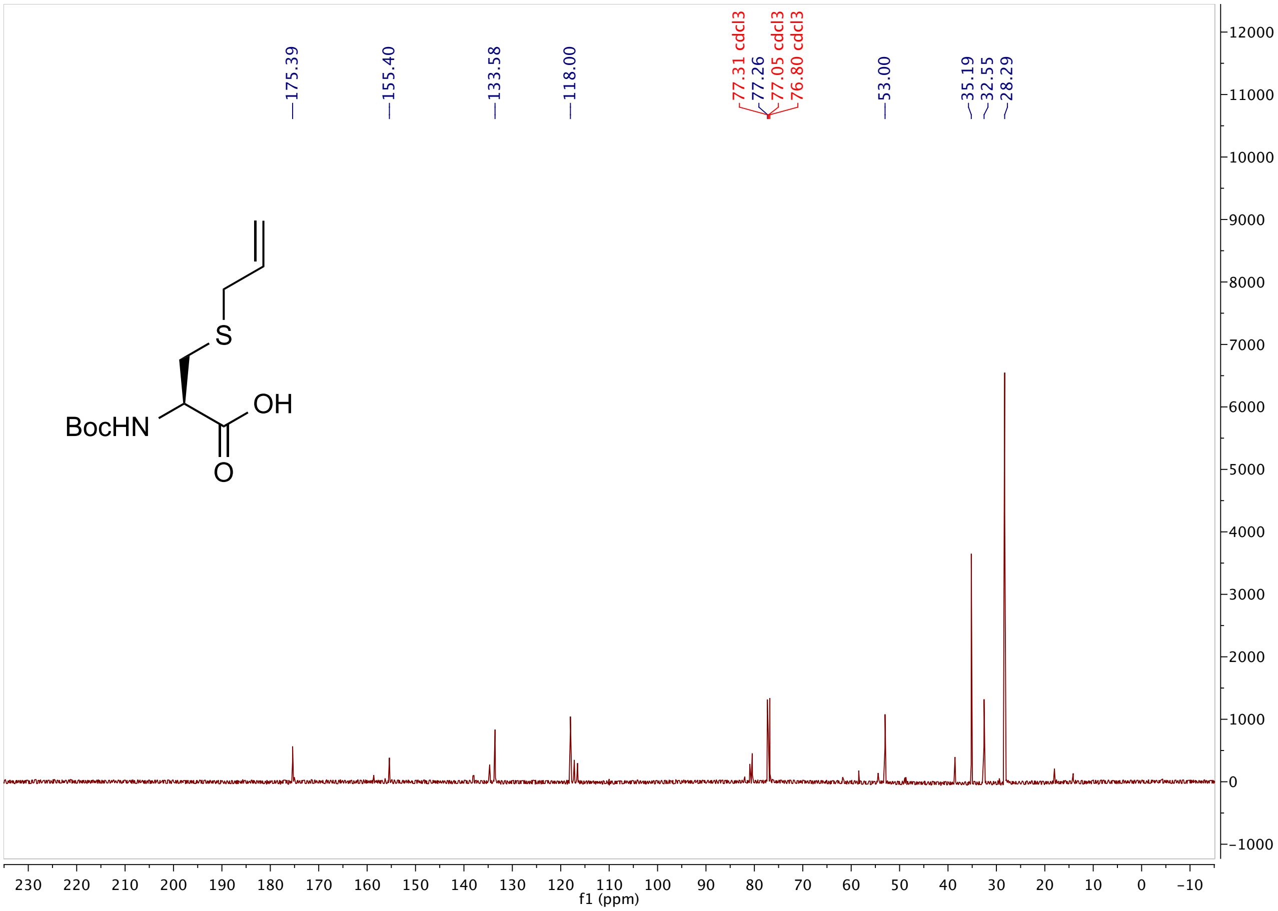

^{1}H NMR (500 MHz, $CDCl_3$) spectrum of compound **S8**

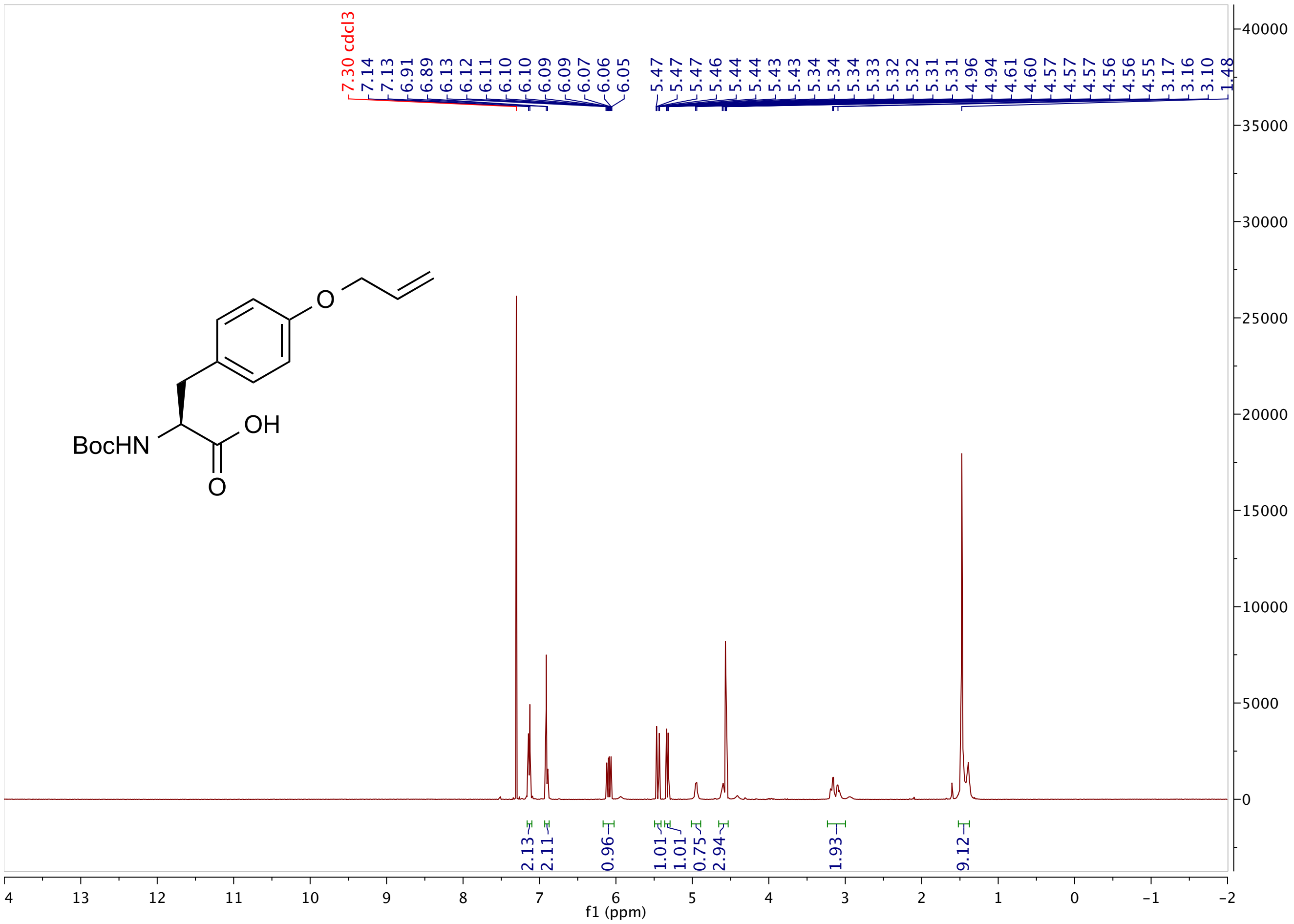

^{13}C NMR (126 MHz, $CDCl_3$) spectrum of compound **S8**

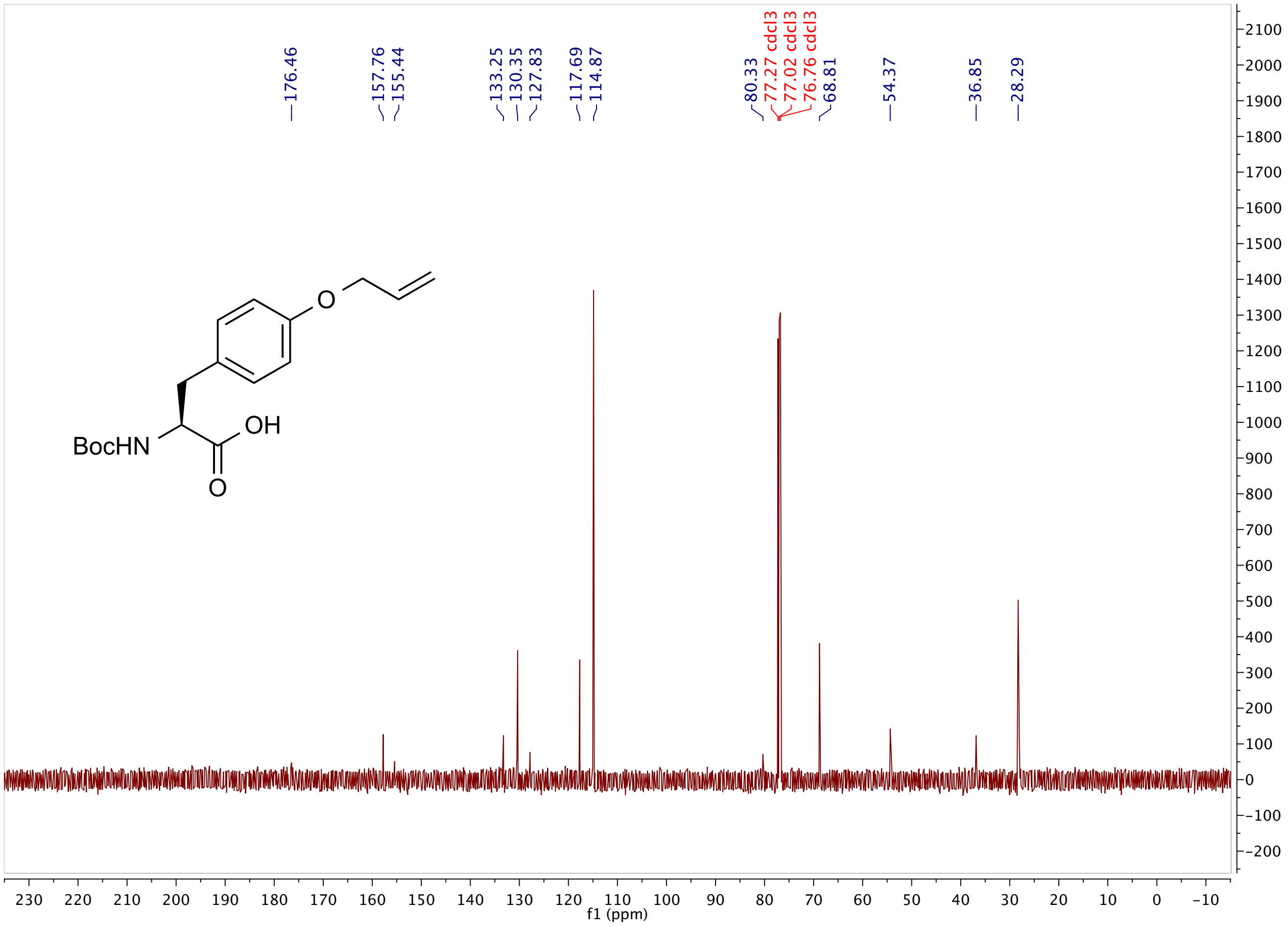

^{1}H NMR (500 MHz, $CDCl_3$) spectrum of compound **S11**

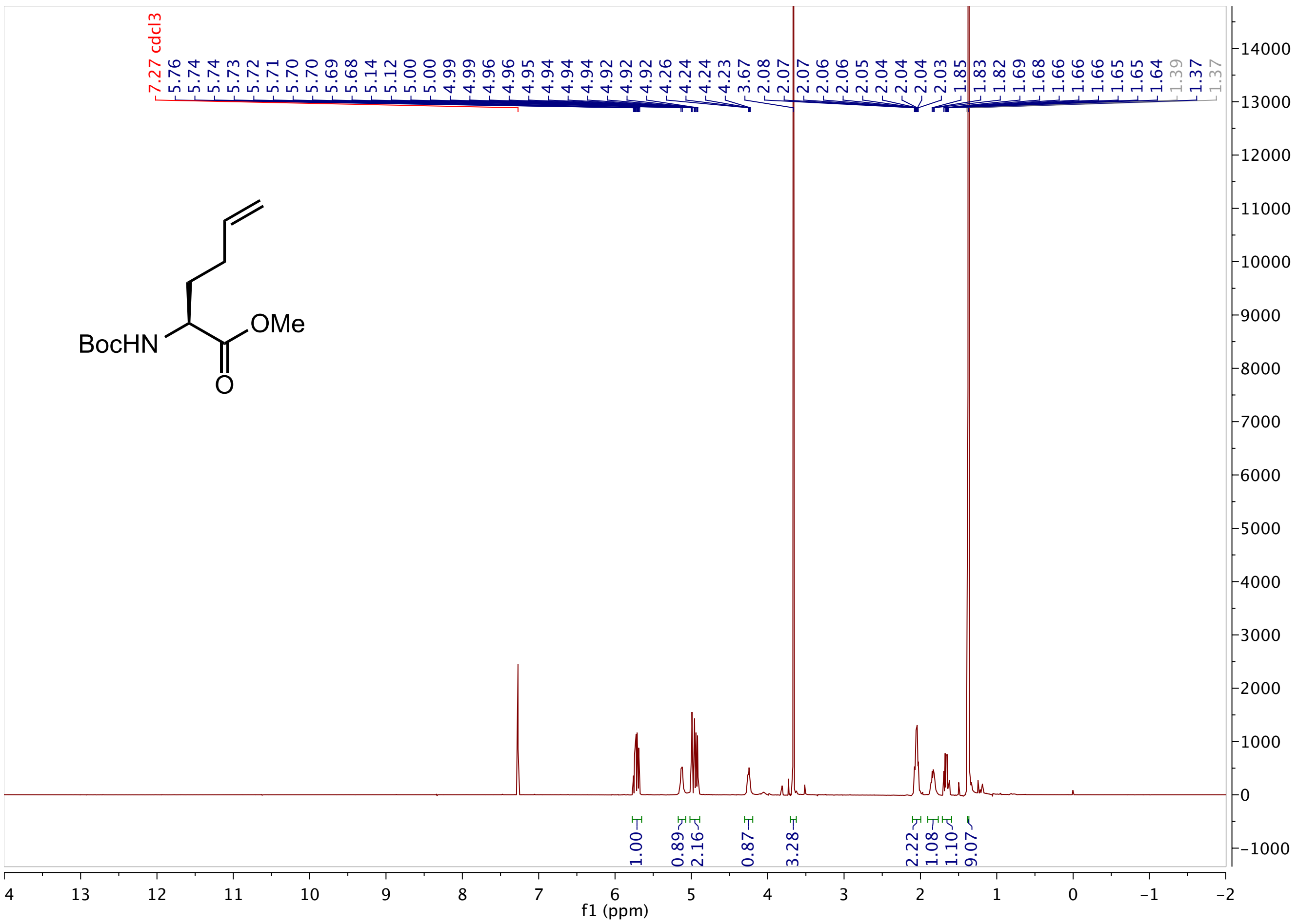

^{13}C NMR (126 MHz, $CDCl_3$) spectrum of compound **S11**

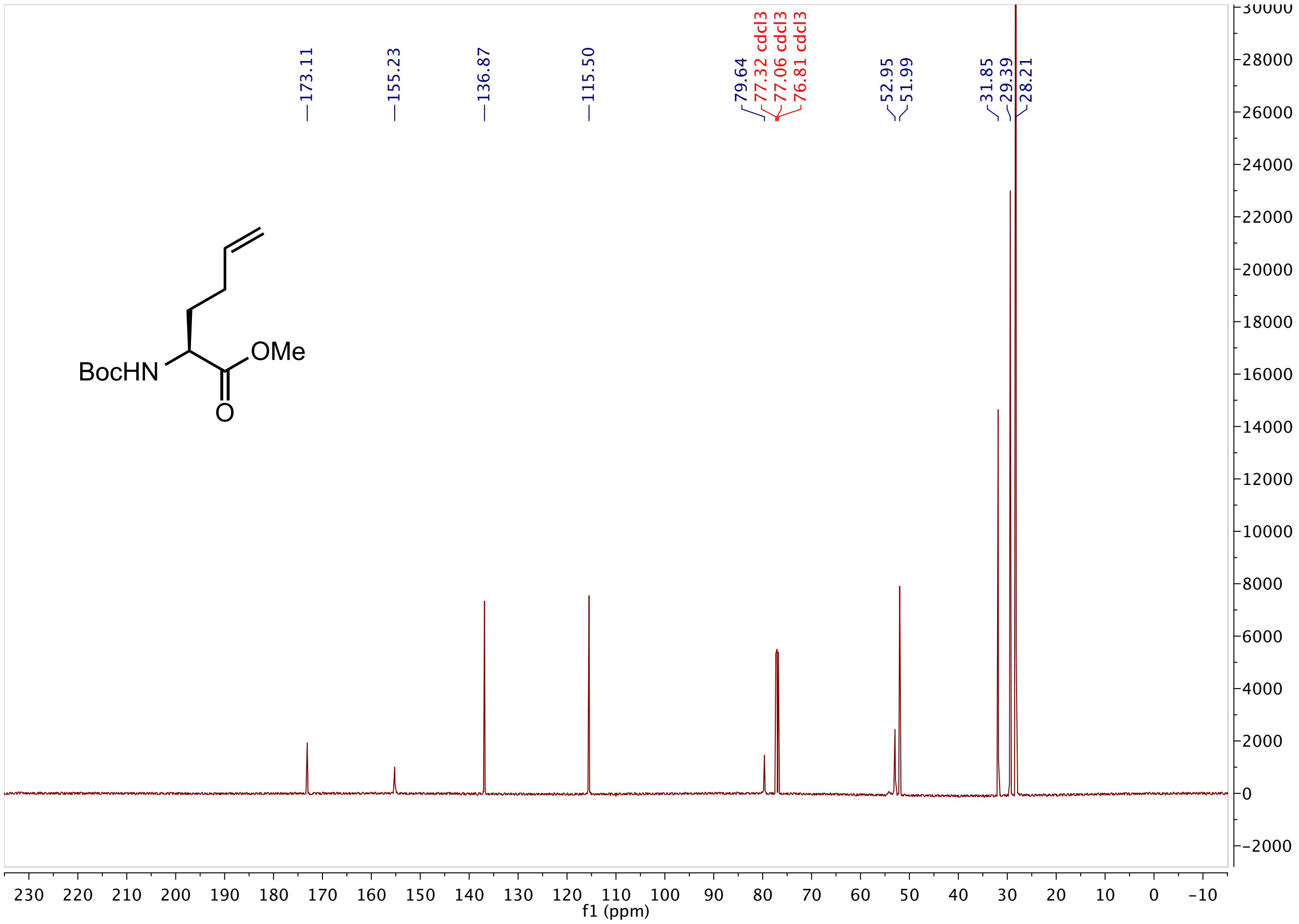

^{1}H NMR (500 MHz, $CDCl_3$) spectrum of compound **S12**

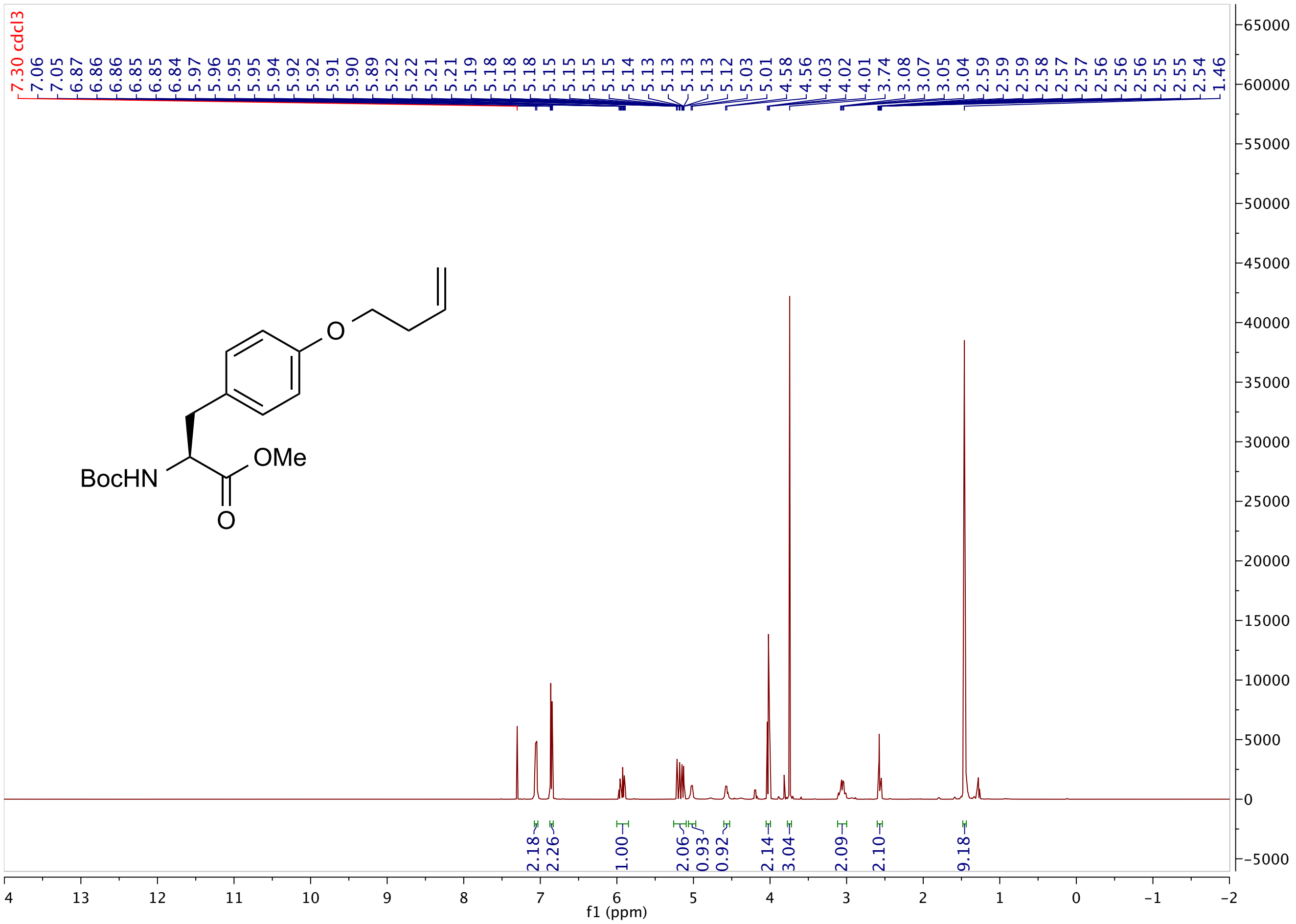

^{13}C NMR (126 MHz, $CDCl_3$) spectrum of compound **S12**

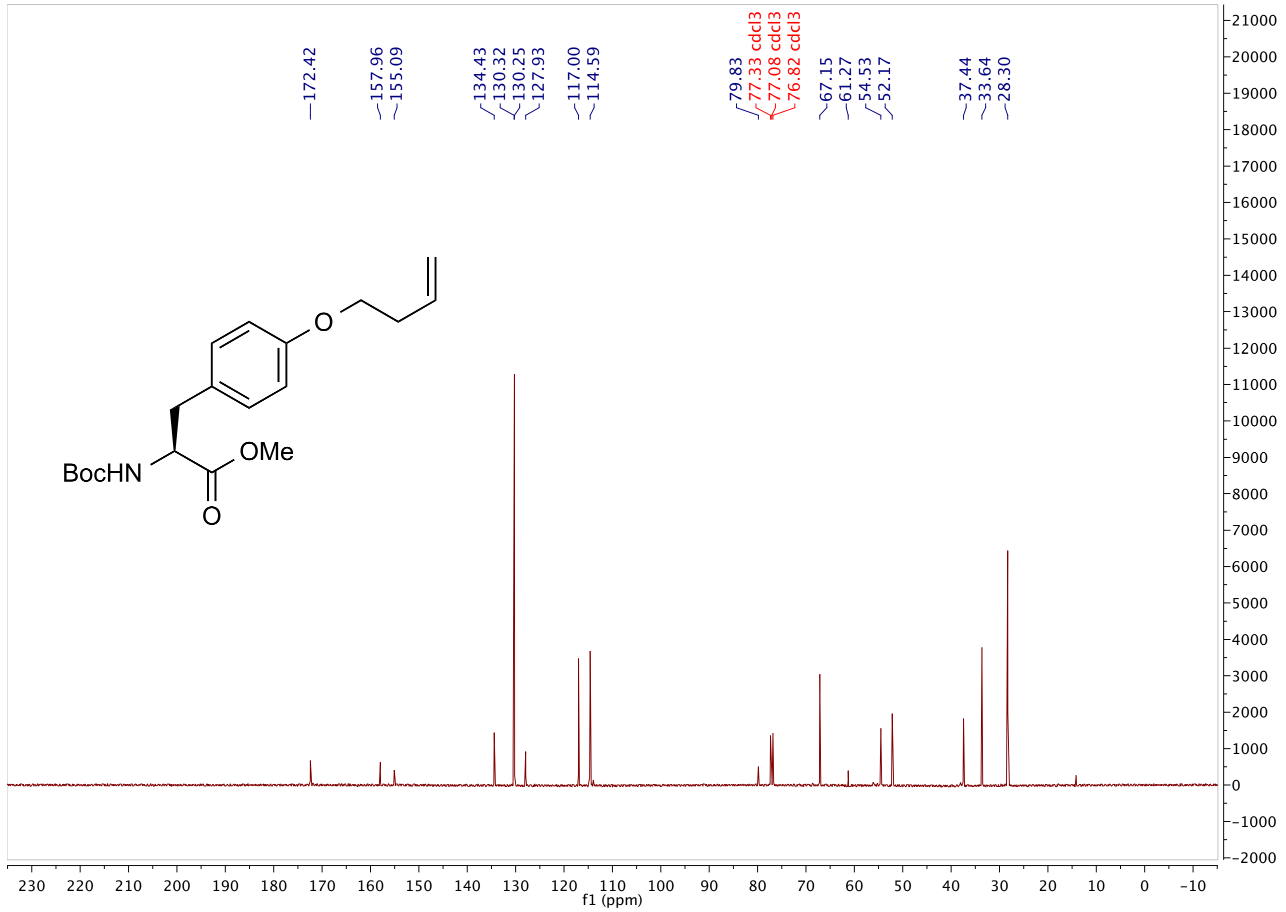

^{1}H NMR (500 MHz, $CDCl_3$) spectrum of compound **S13**

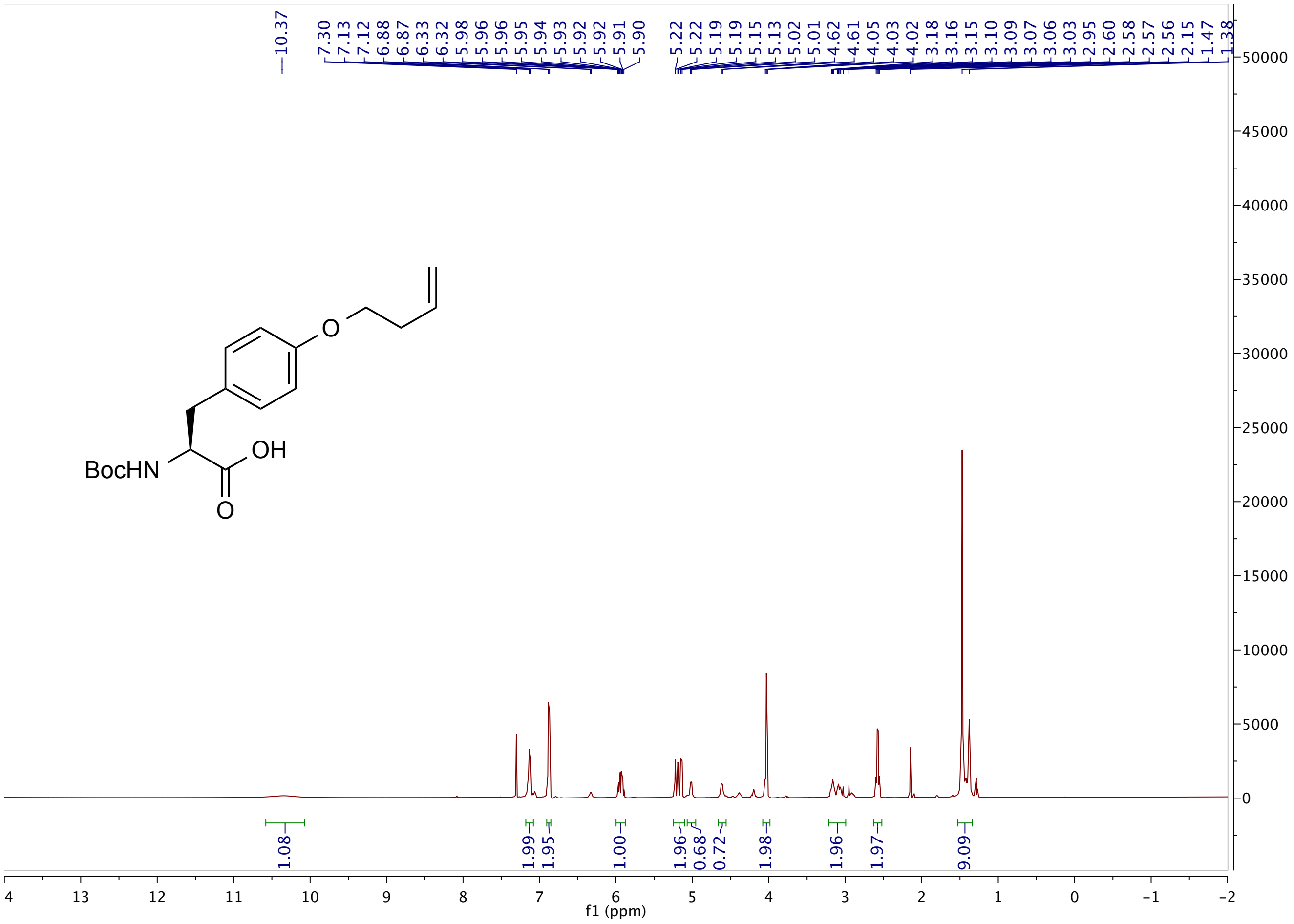

^{13}C NMR (126 MHz, $CDCl_3$) spectrum of compound **S13**

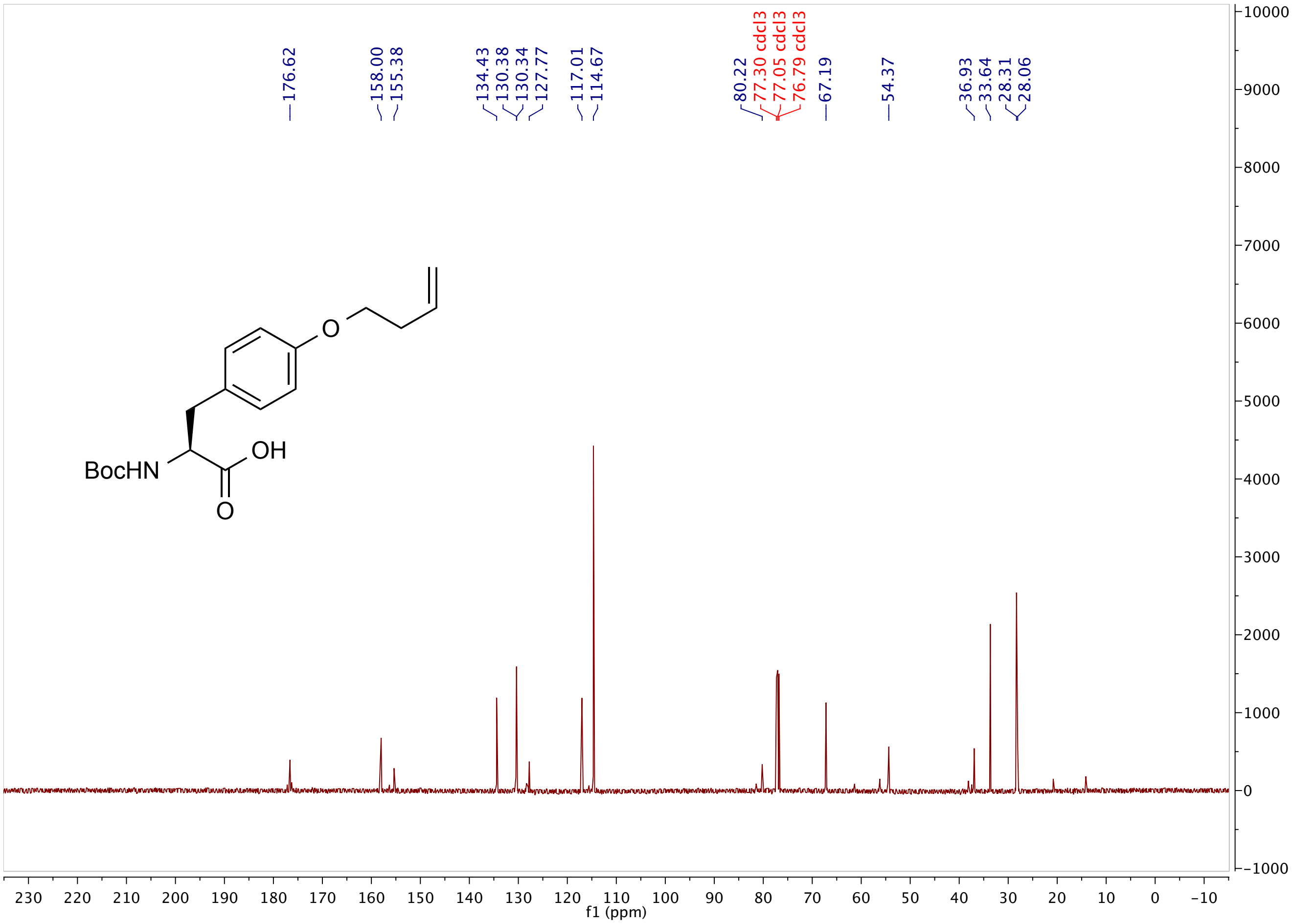

^1H NMR (500 MHz, $CDCl_3$) spectrum of compound **8a**

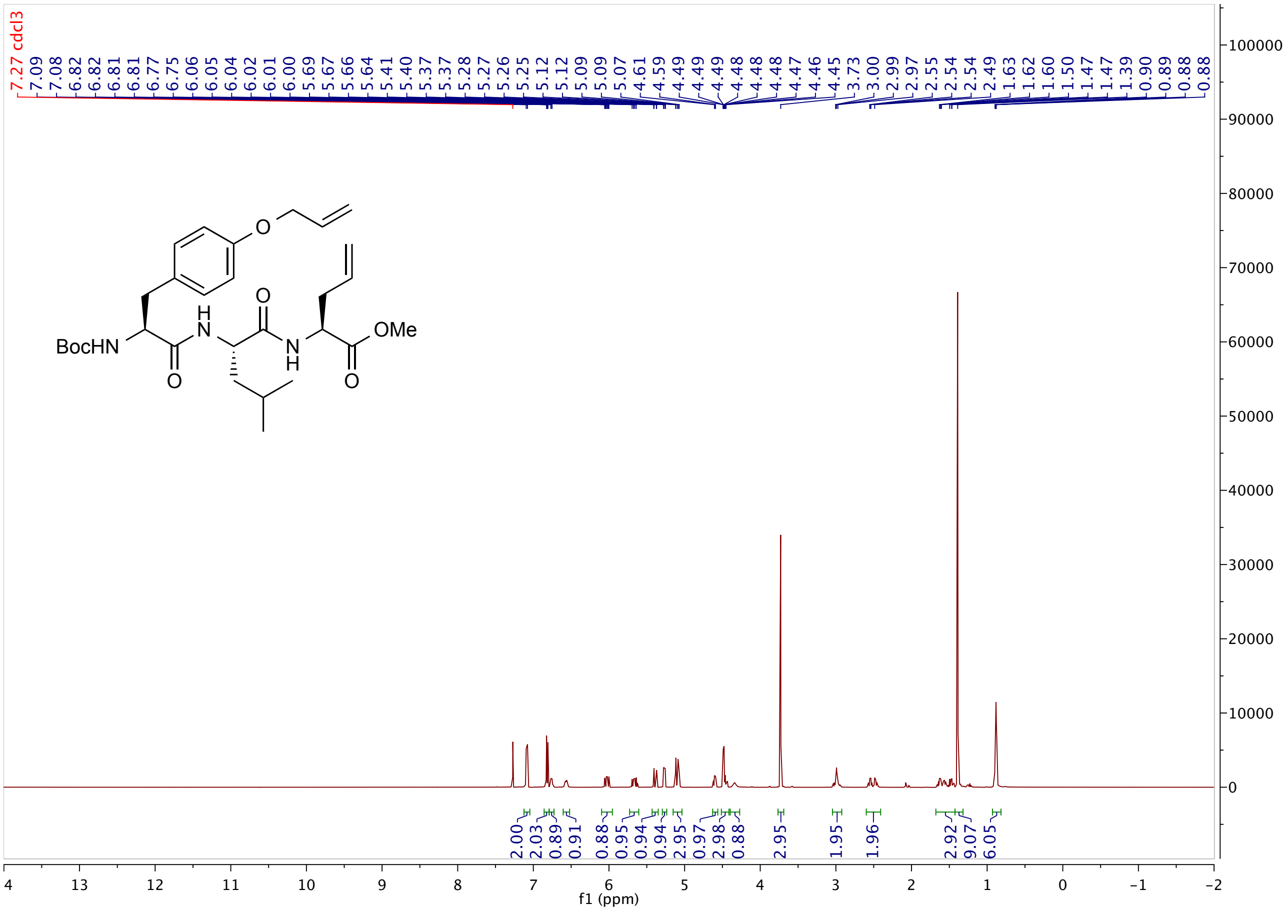

^{13}C NMR (126 MHz, $CDCl_3$) spectrum of compound **8a**

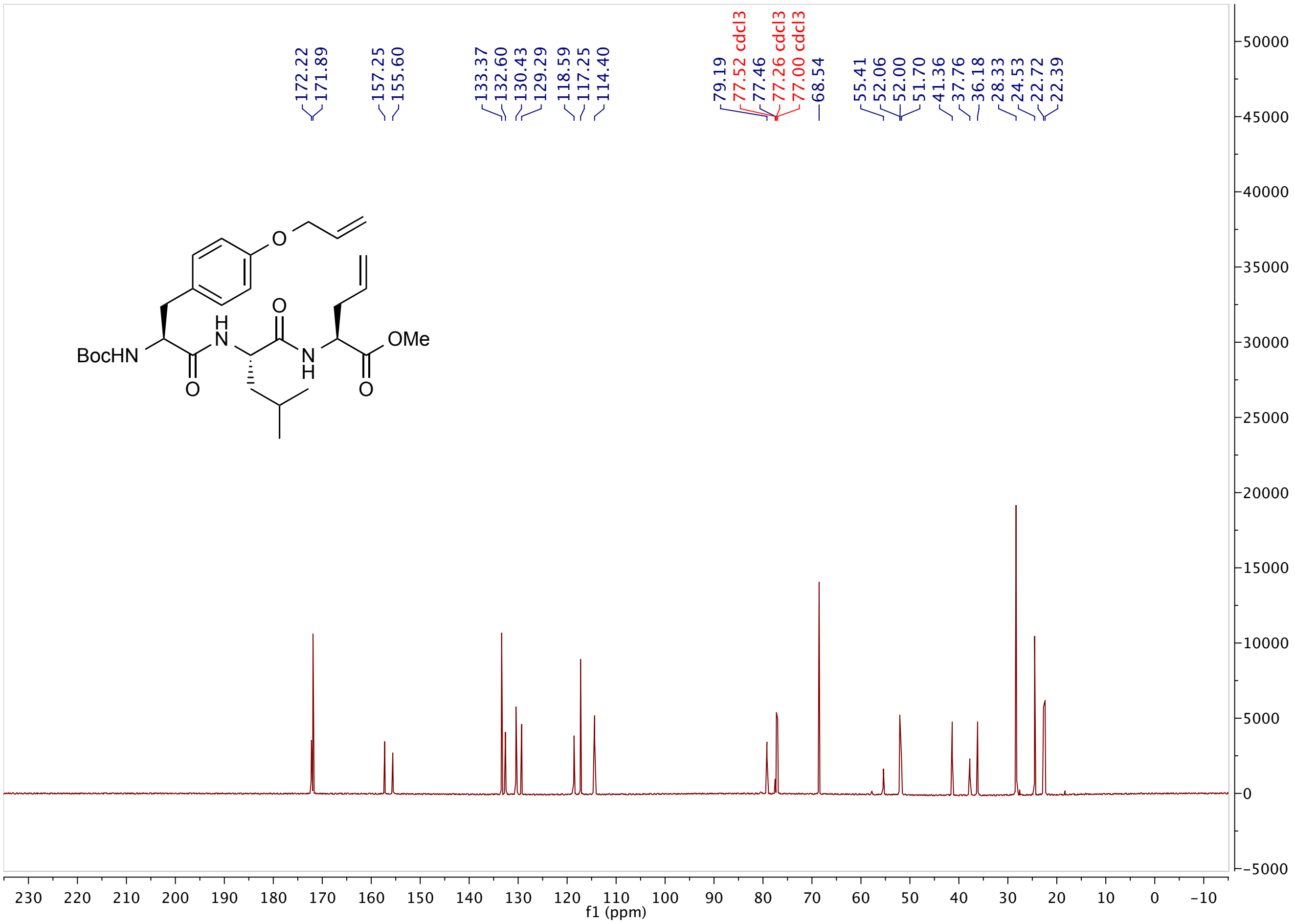

^1H NMR (500 MHz, $CDCl_3$) spectrum of compound **8b**

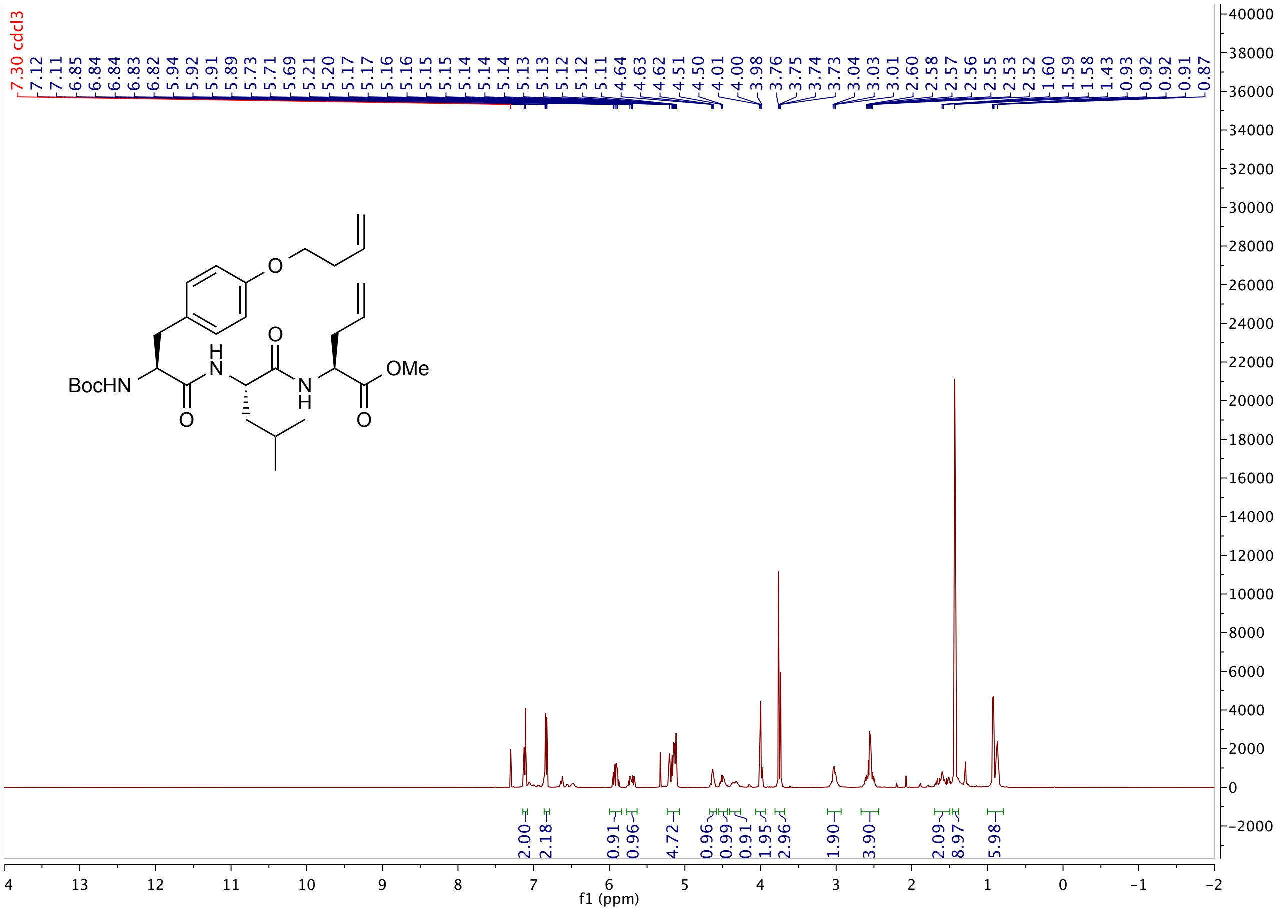

^{13}C NMR (126 MHz, $CDCl_3$) spectrum of compound **8b**

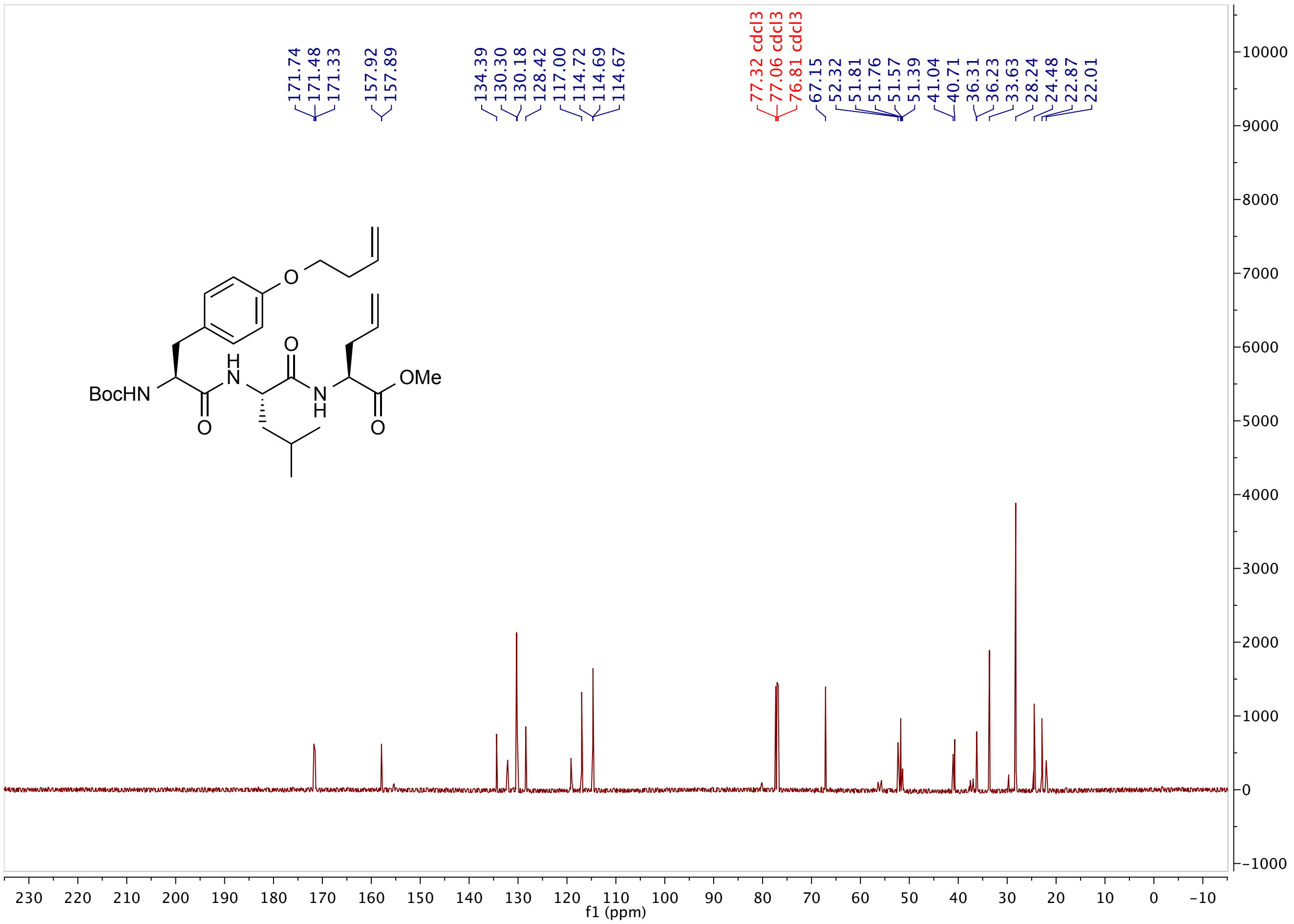

^{1}H NMR (500 MHz, $CDCl_3$) spectrum of compound **8c**

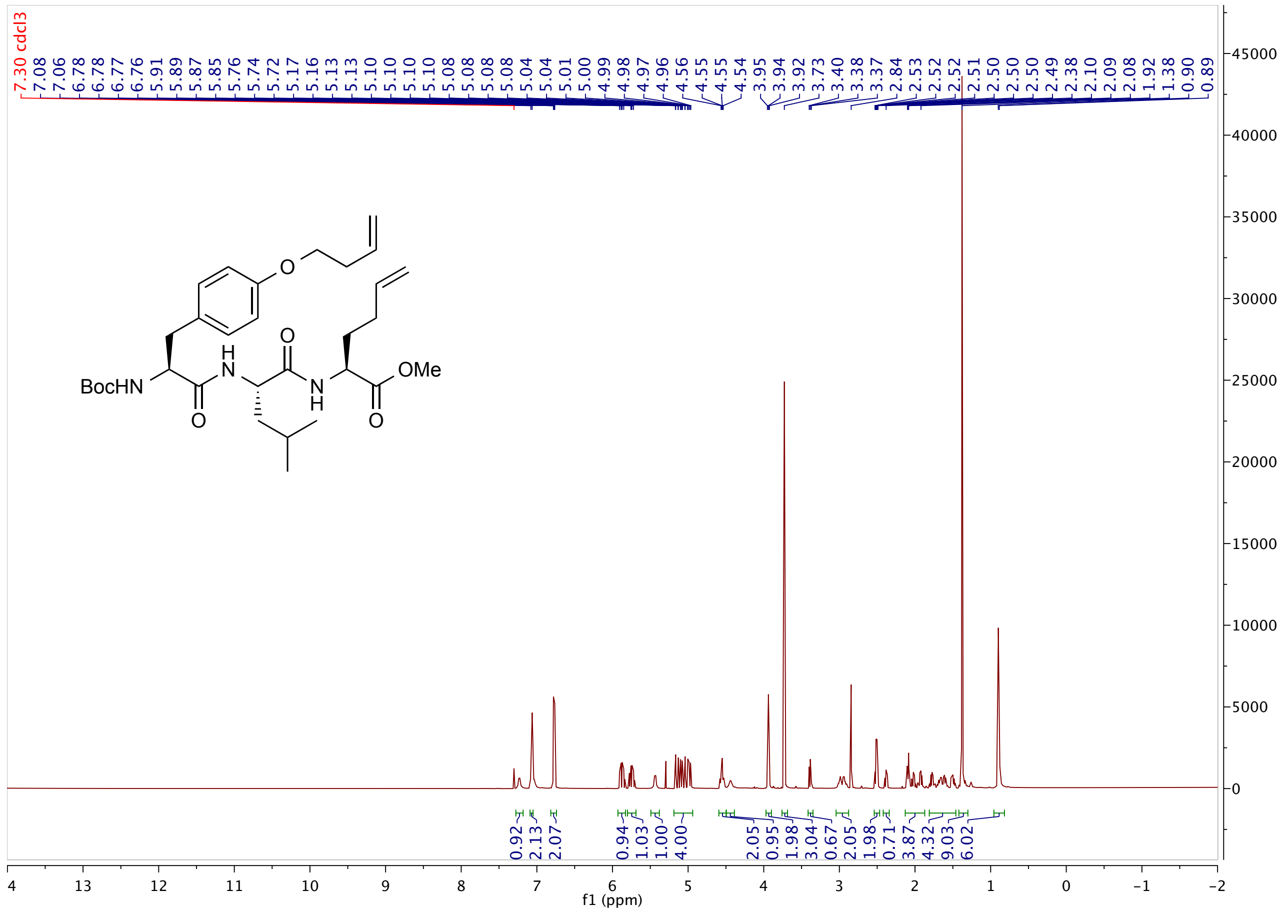

^{13}C NMR (126 MHz, $CDCl_3$) spectrum of compound **8c**

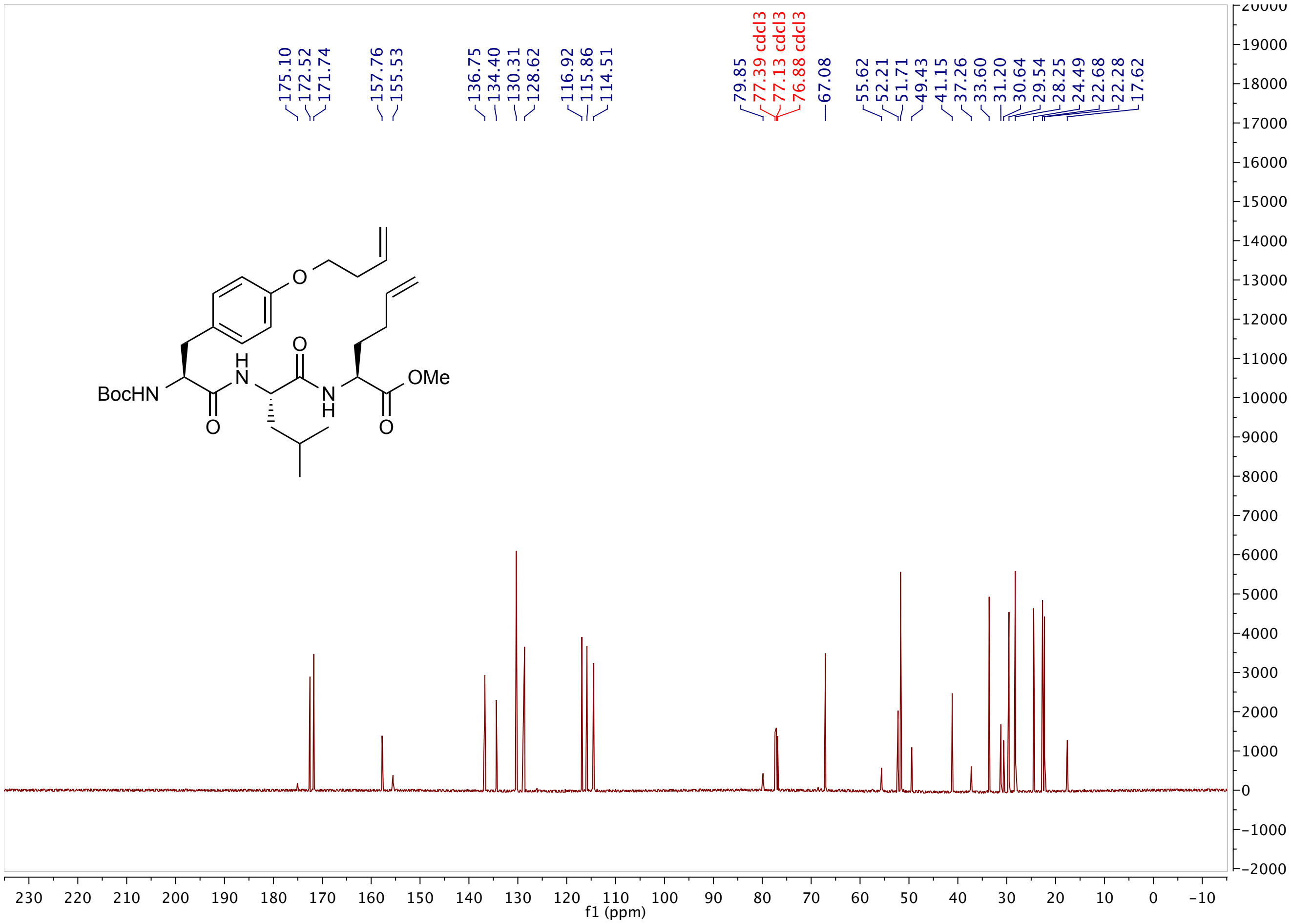

^{1}H NMR (500 MHz, $CDCl_3$) spectrum of compound **10**

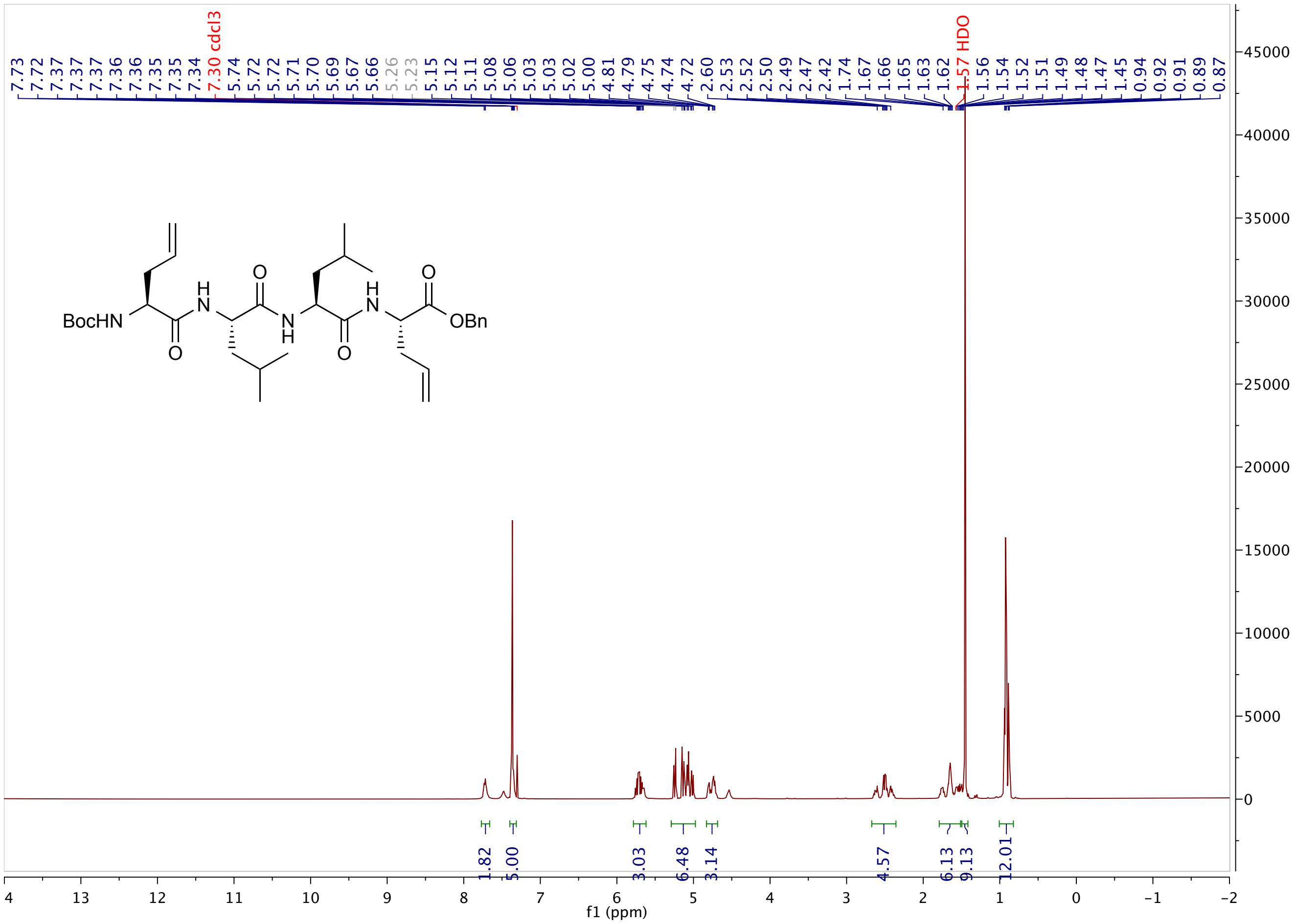

^{13}C NMR (126 MHz, $CDCl_3$) spectrum of compound **10**

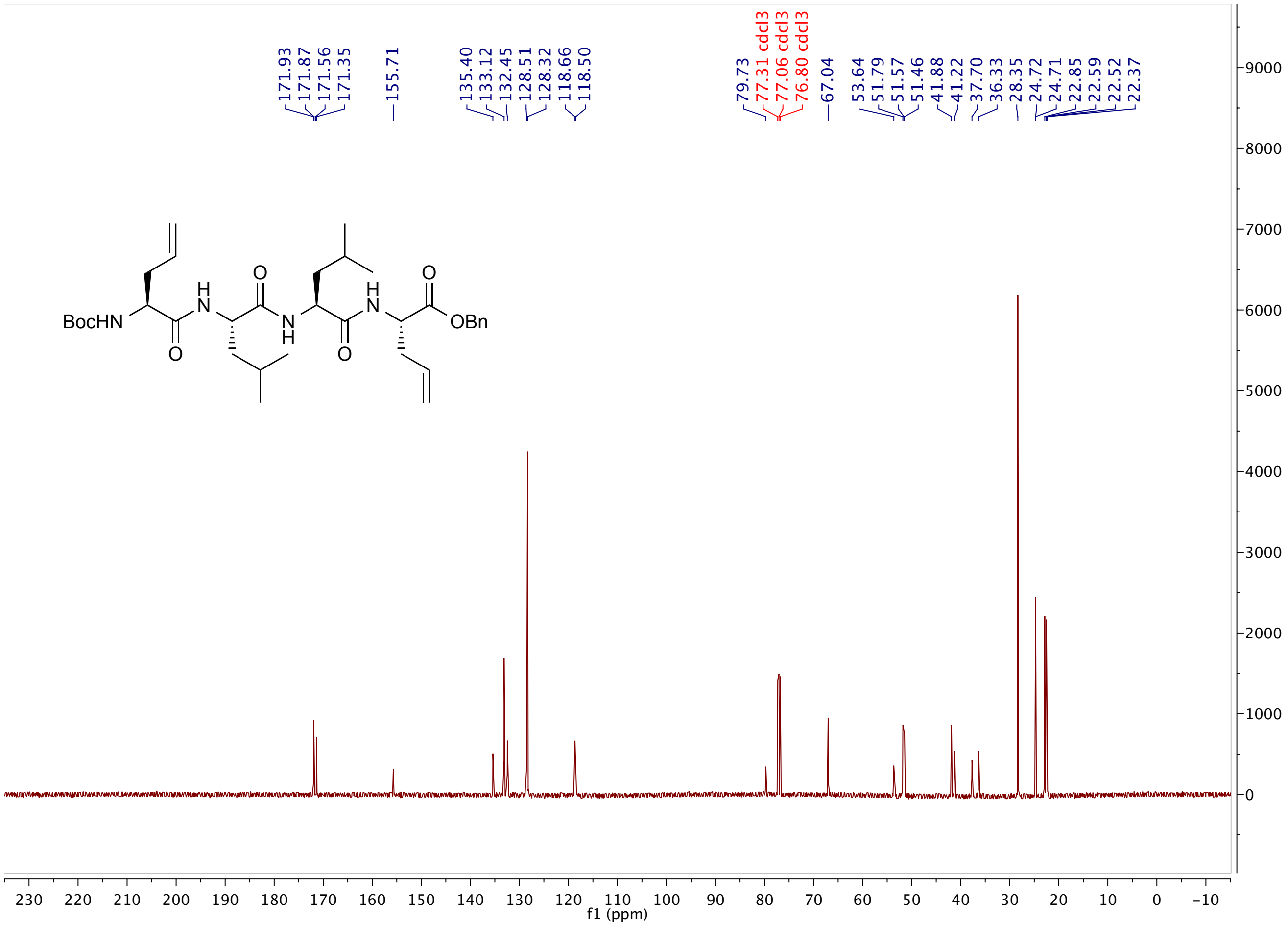

^{1}H NMR (500 MHz, $CDCl_3$) spectrum of compound **11**

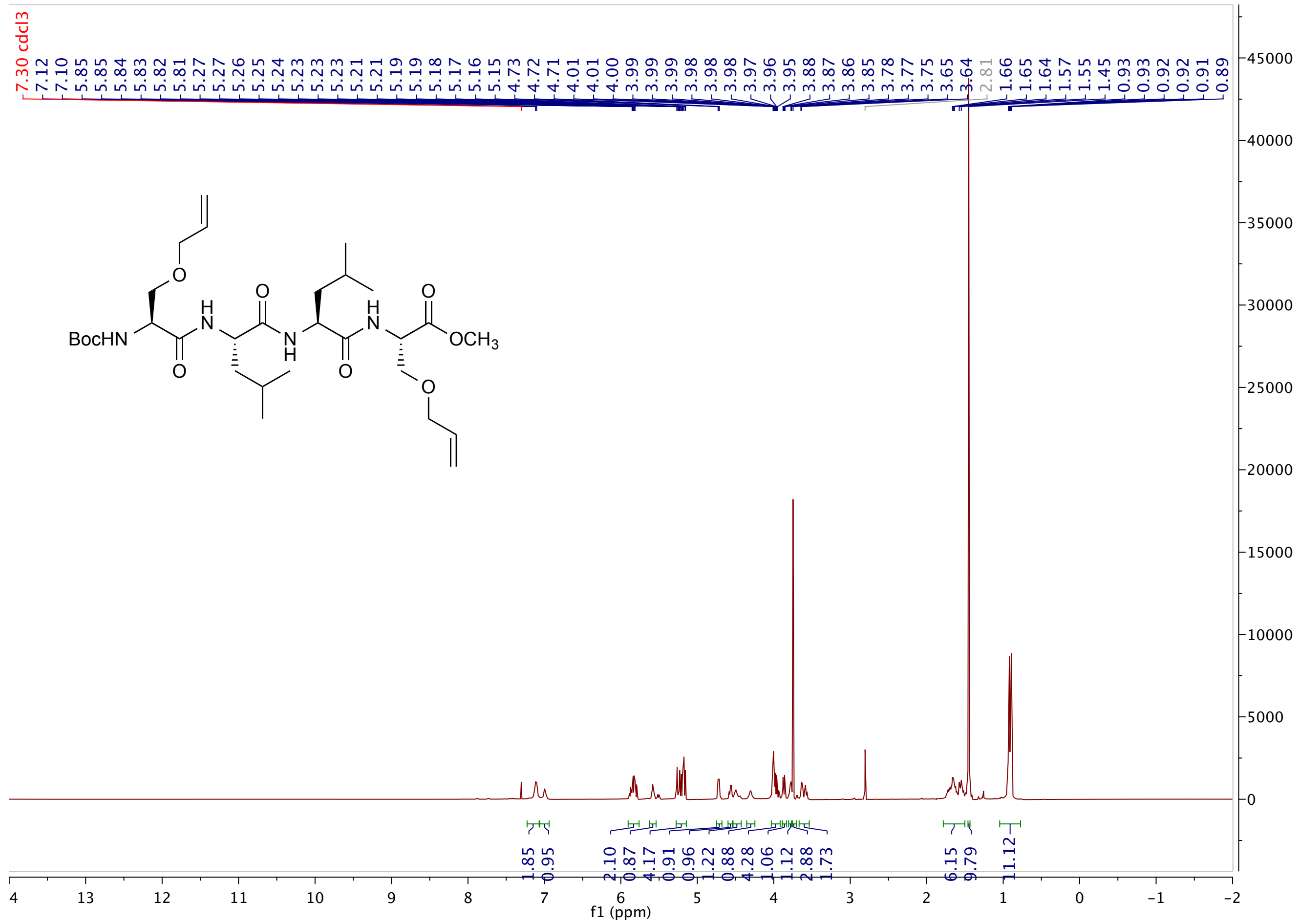

^{13}C NMR (126 MHz, $CDCl_3$) spectrum of compound **11**

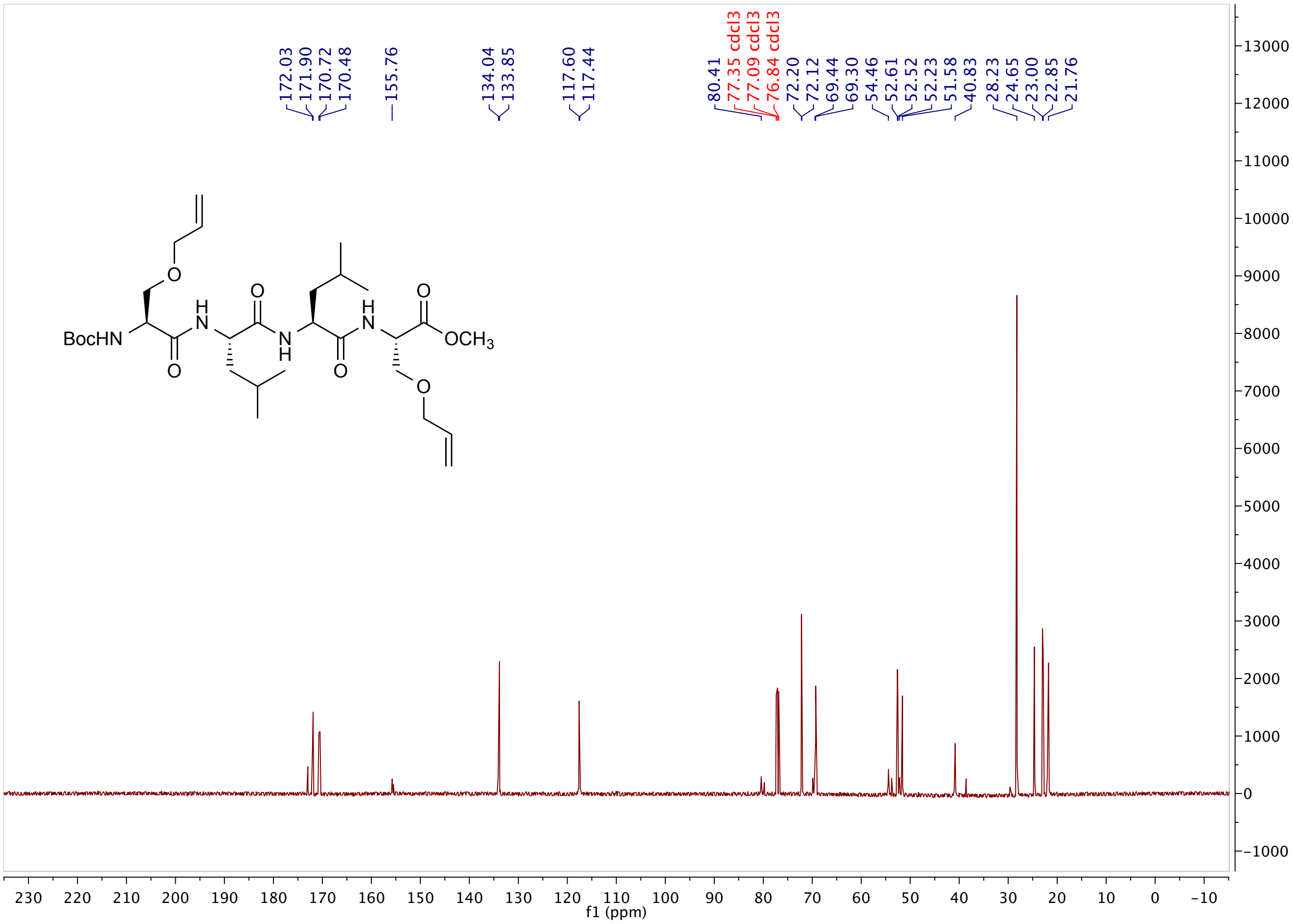

^{1}H NMR (500 MHz, $CDCl_3$) spectrum of compound **12**

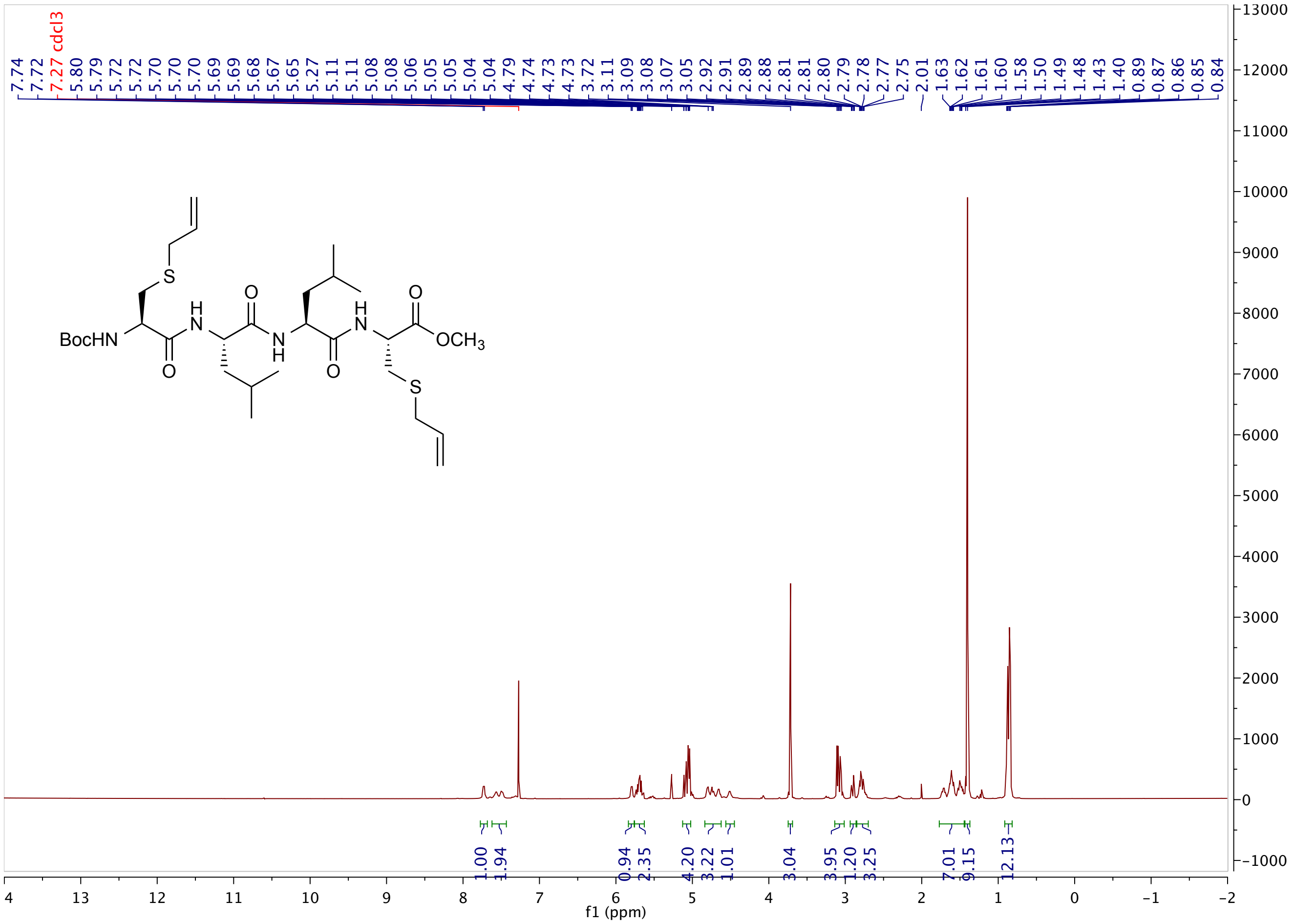

^{13}C NMR (126 MHz, $CDCl_3$) spectrum of compound **12**

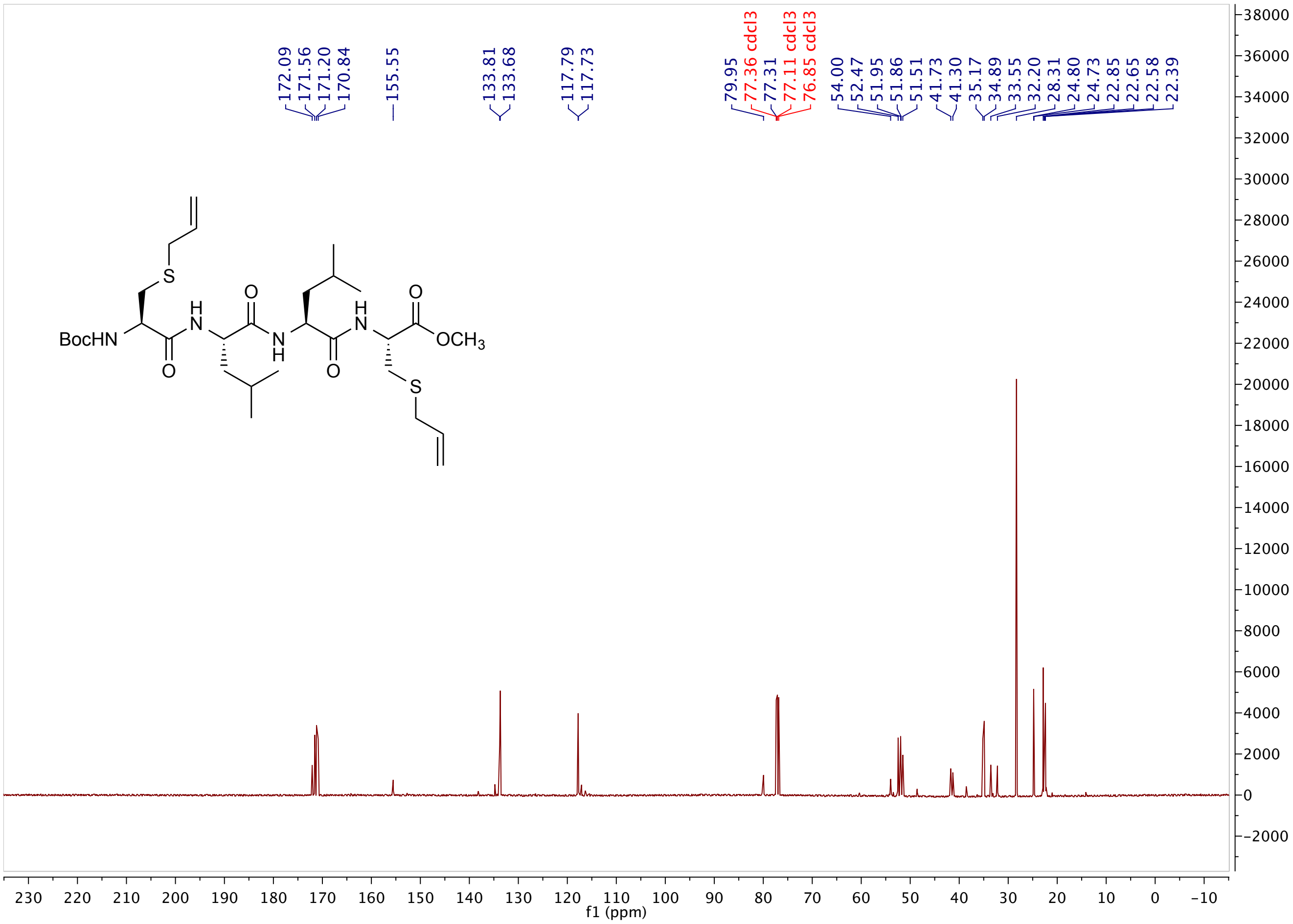

^1H NMR (500 MHz, $CDCl_3$) spectrum of compound **13**

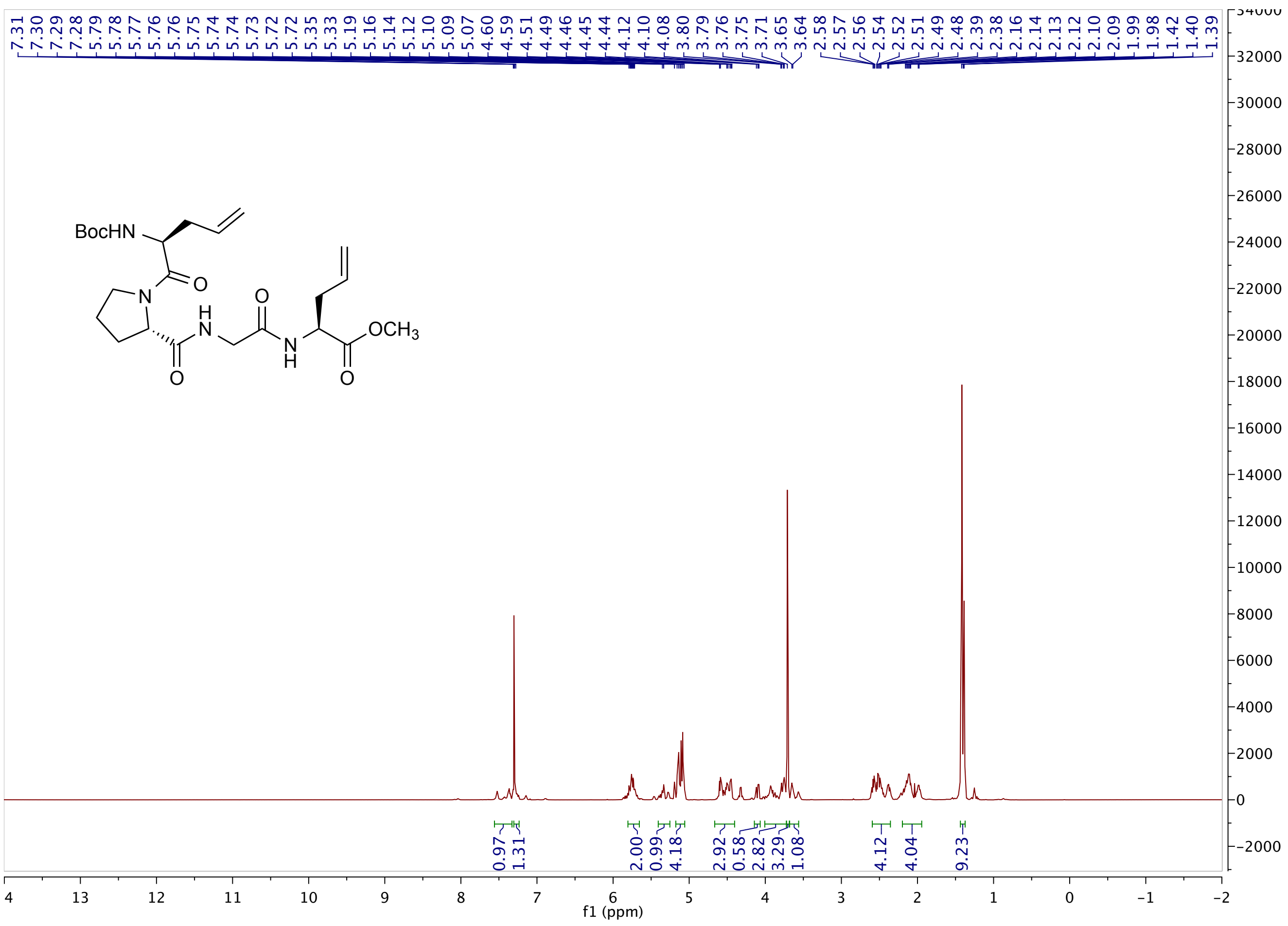

^{13}C NMR (126 MHz, $CDCl_3$) spectrum of compound **13**

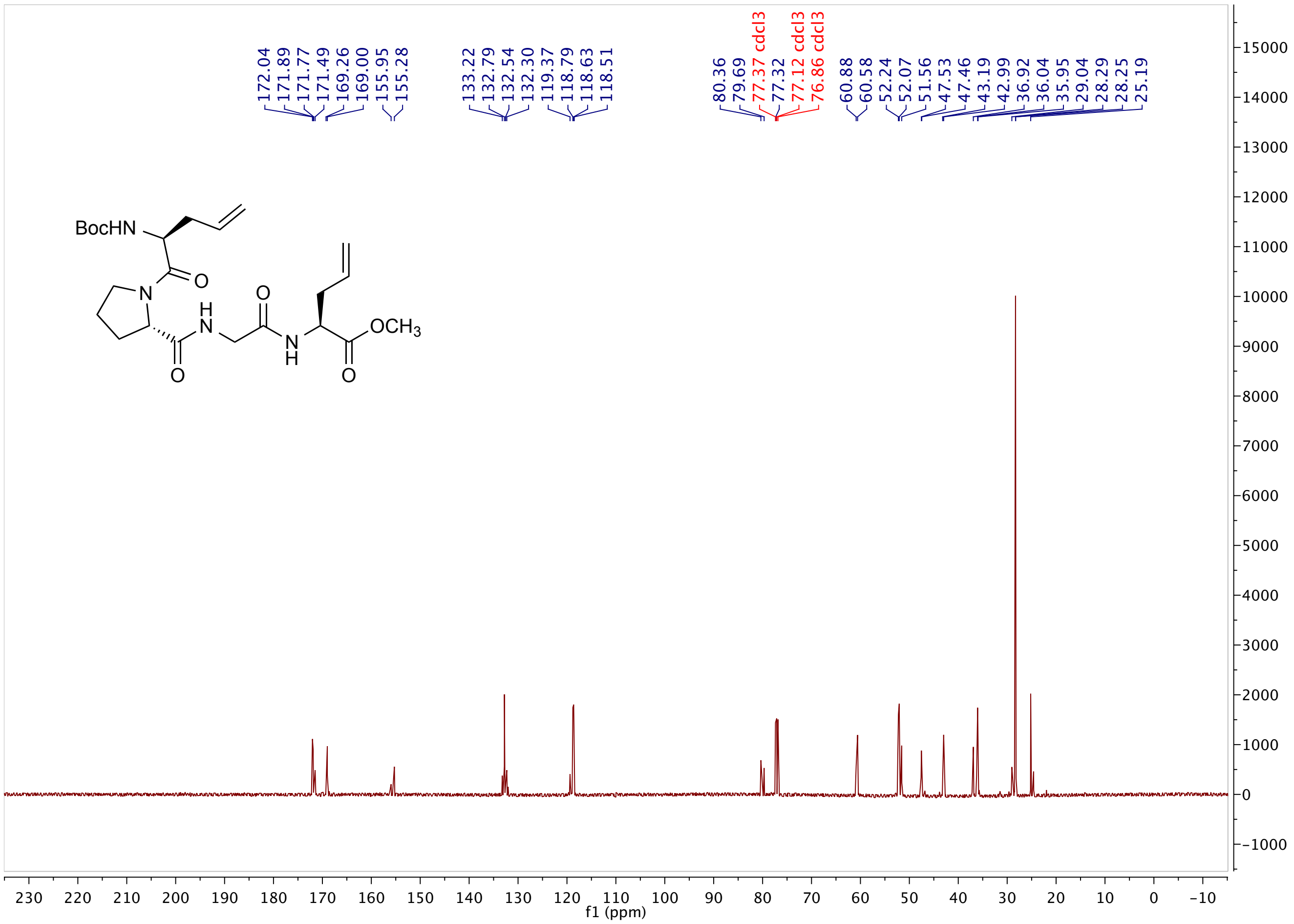

^1H NMR (500 MHz, $CDCl_3$) spectrum of compound **14**

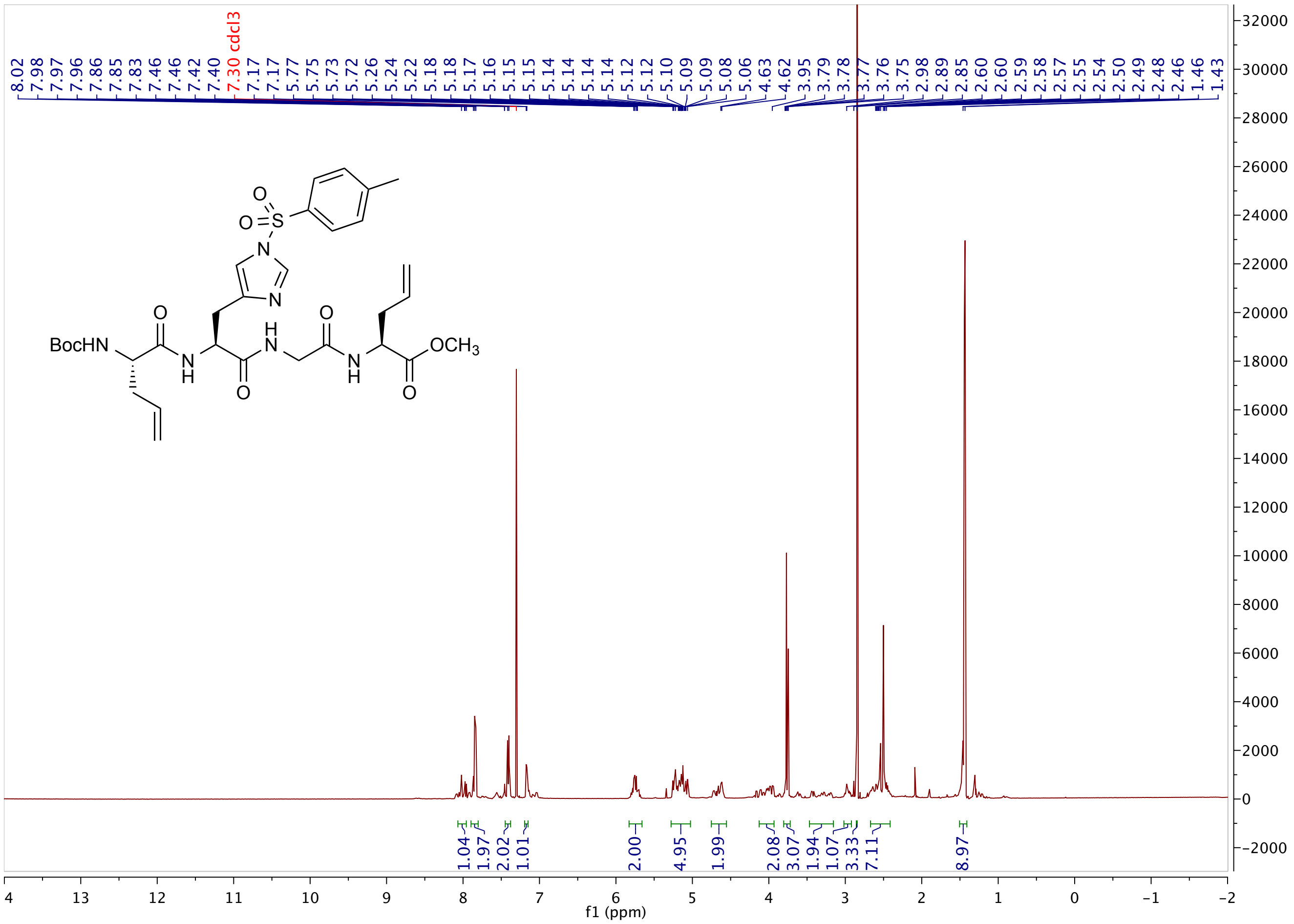

^{13}C NMR (126 MHz, $CDCl_3$) spectrum of compound **14**

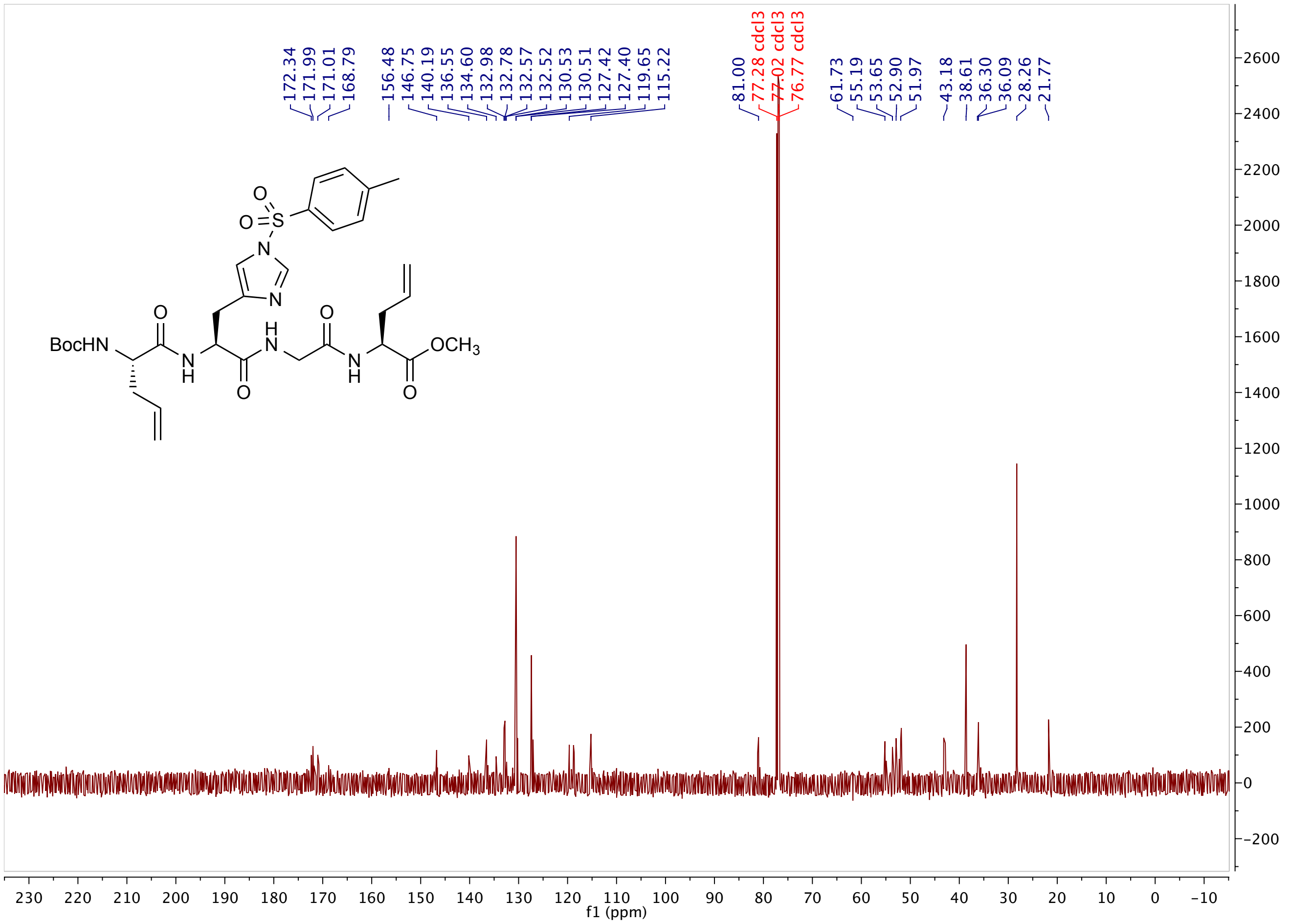

^{1}H NMR (500 MHz, $CDCl_3$) spectrum of compound **9a**

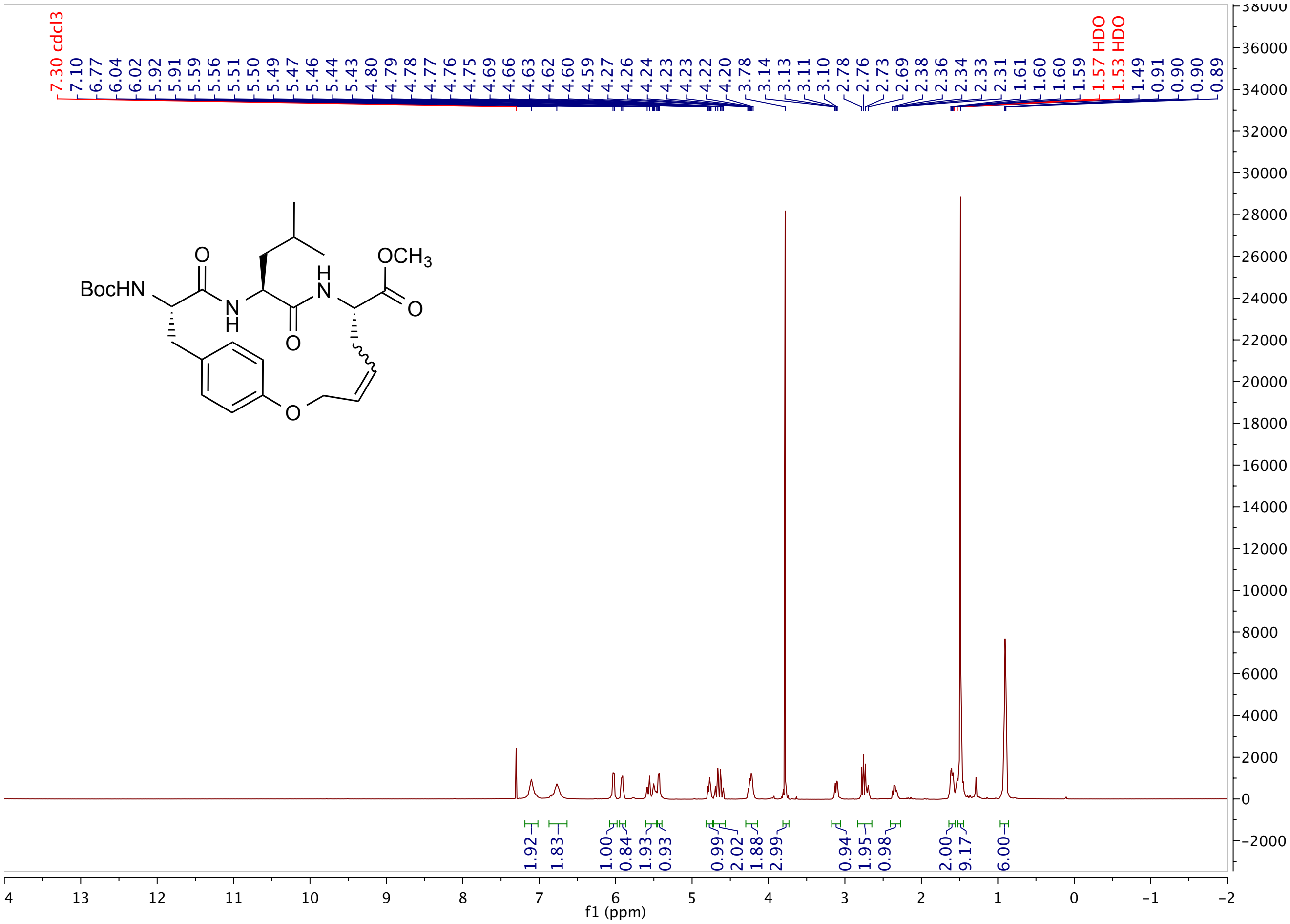

^{13}C NMR (126 MHz, $CDCl_3$) spectrum of compound **9a**

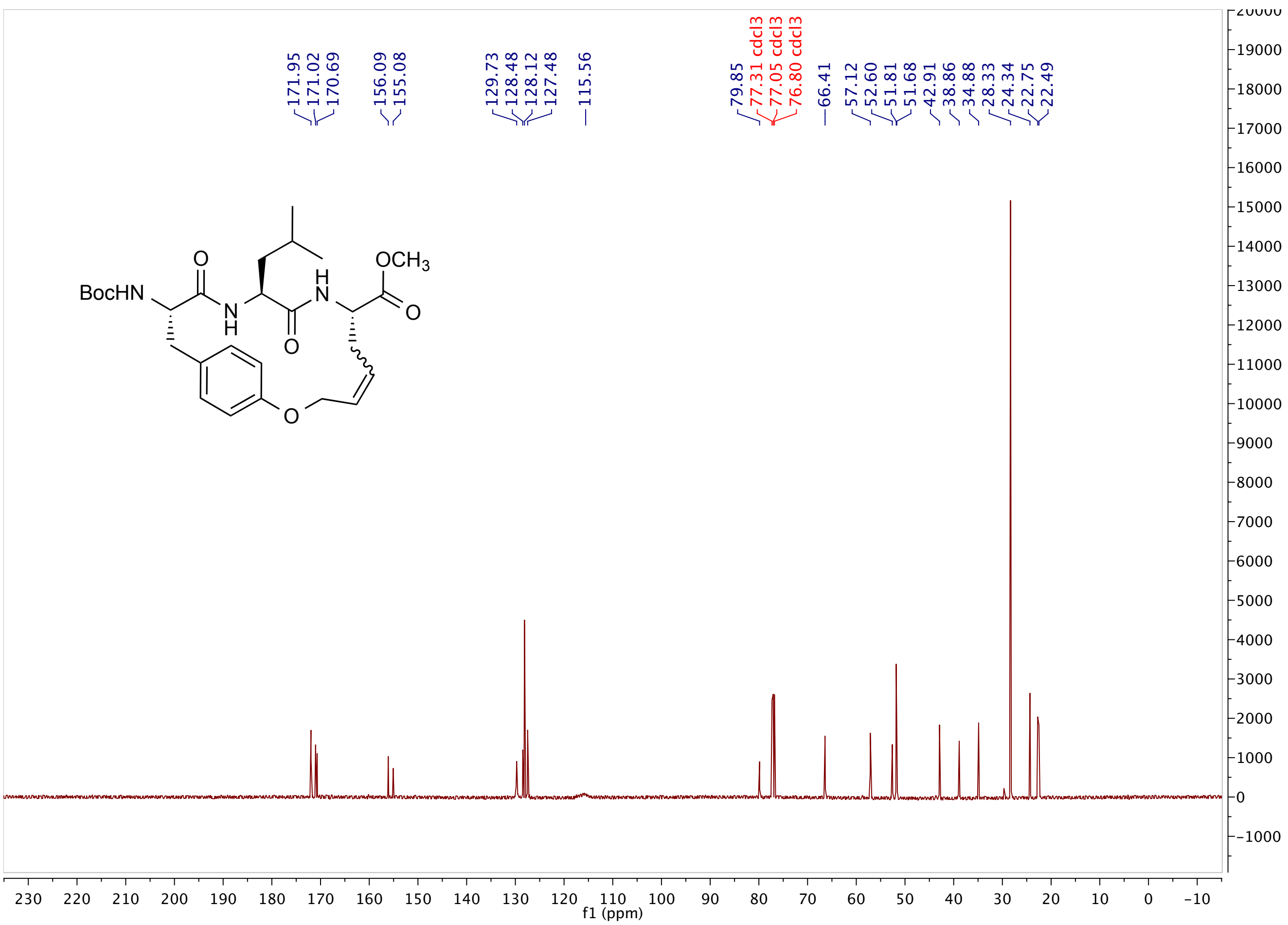

^{1}H NMR (500 MHz, $CDCl_3$) spectrum of compound **9b**

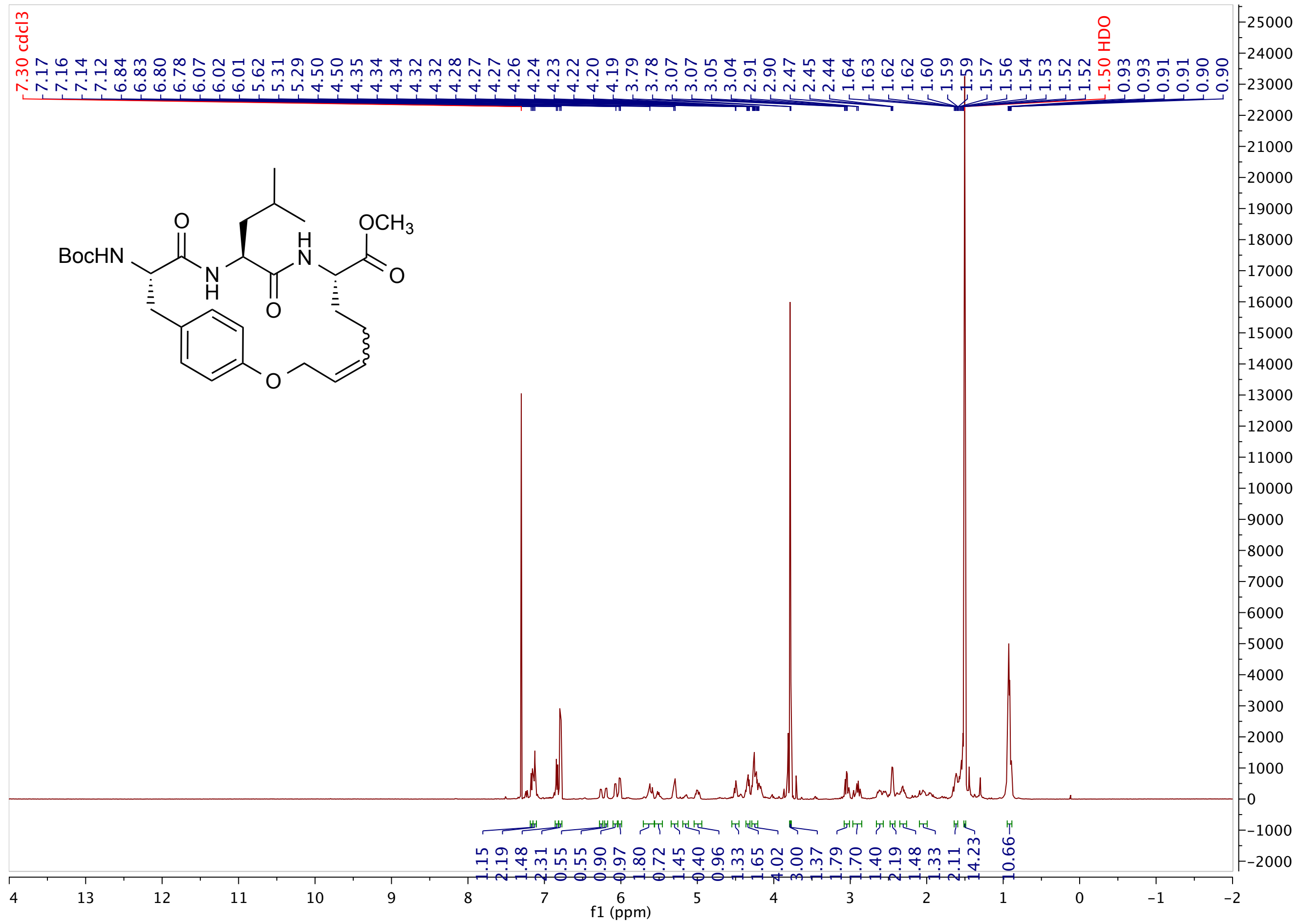

^{13}C NMR (126 MHz, $CDCl_3$) spectrum of compound **9b**

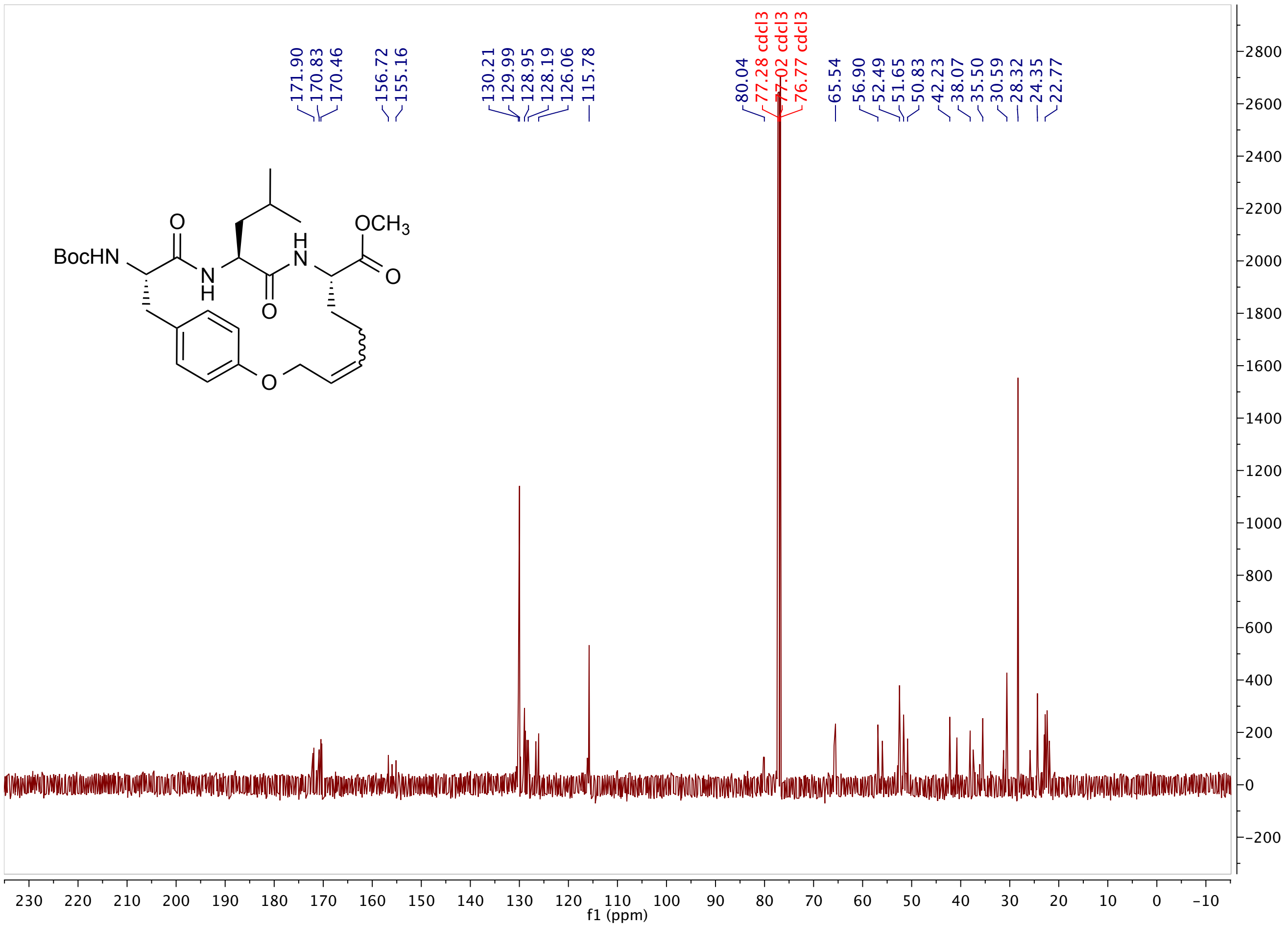

H NMR (500 MHz, $CDCl_3$) spectrum of compound **9c**

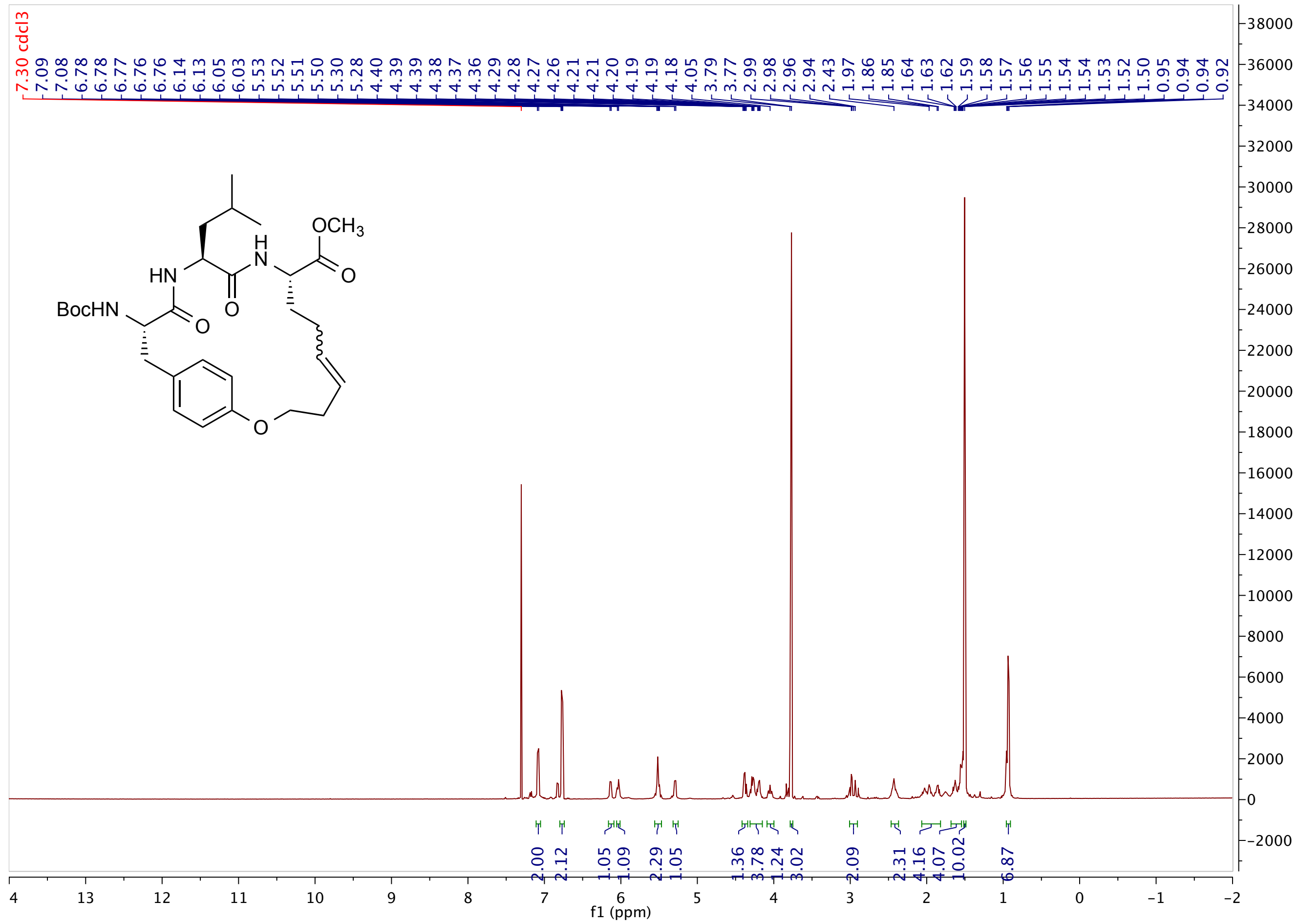

^{13}C NMR (126 MHz, $CDCl_3$) spectrum of compound **9c**

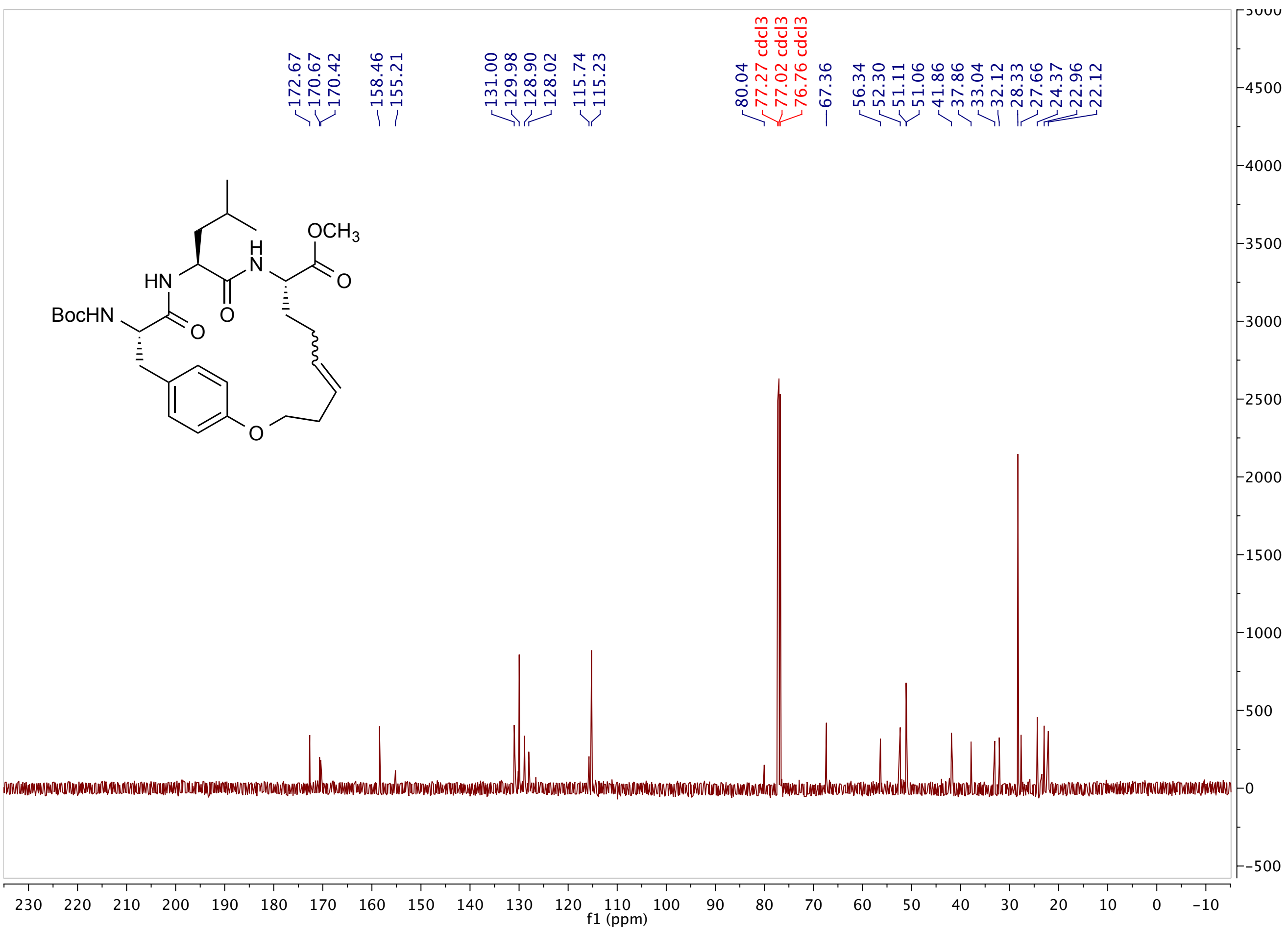

^1H NMR (500 MHz, $CDCl_3$) spectrum of compound **15**

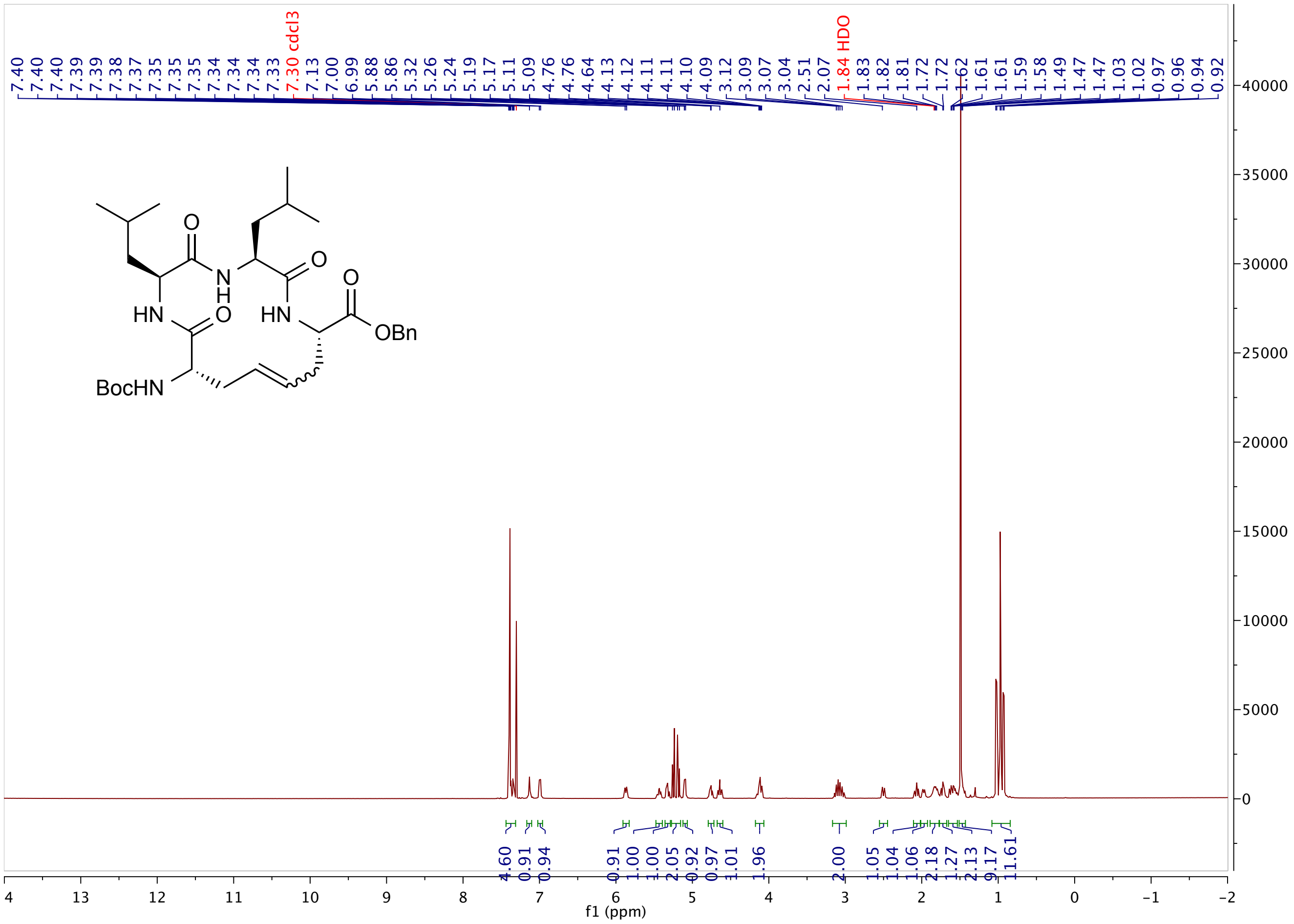

^{13}C NMR (126 MHz, $CDCl_3$) spectrum of compound **15**

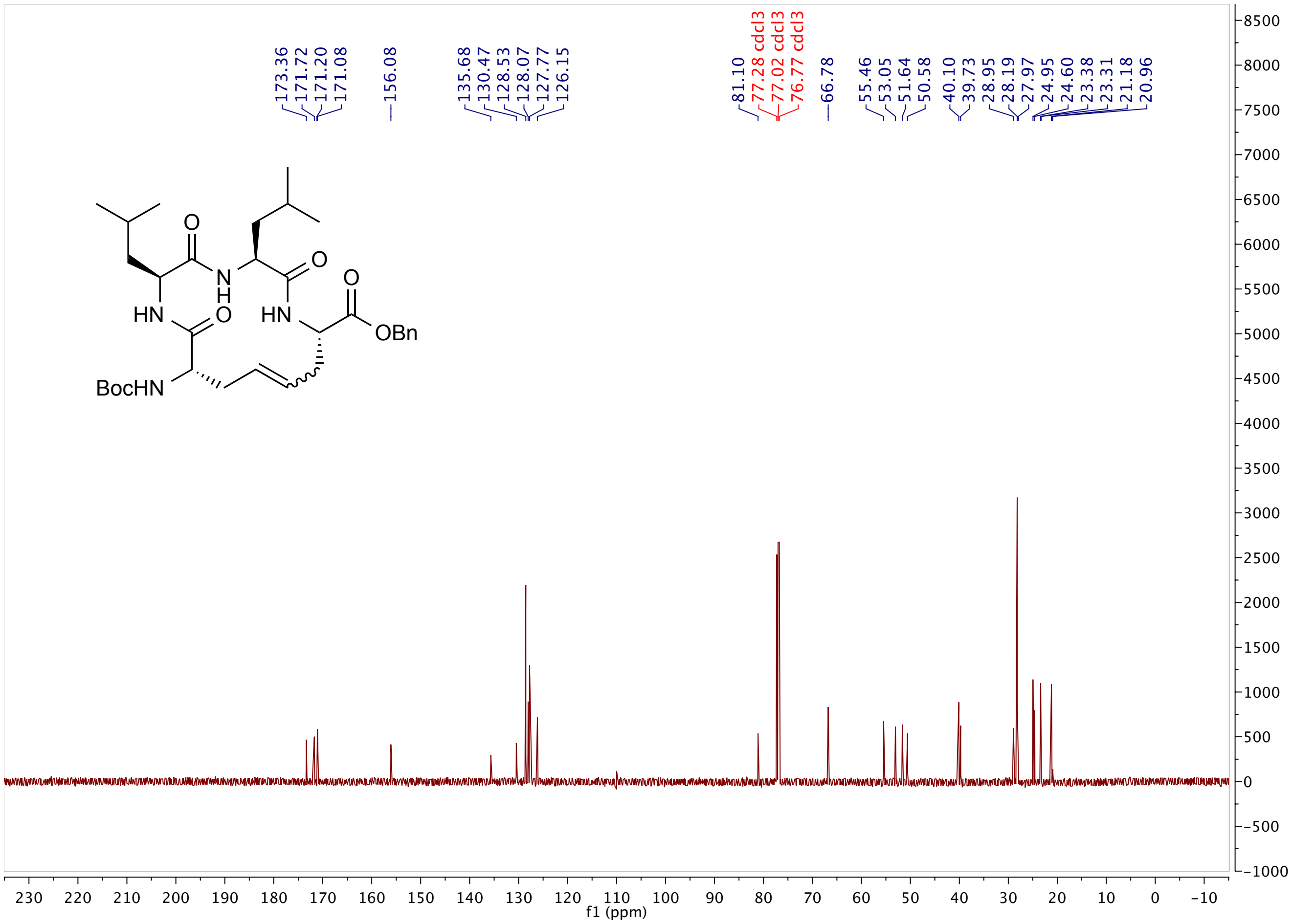

^1H NMR (500 MHz, $CDCl_3$) spectrum of compound **16**

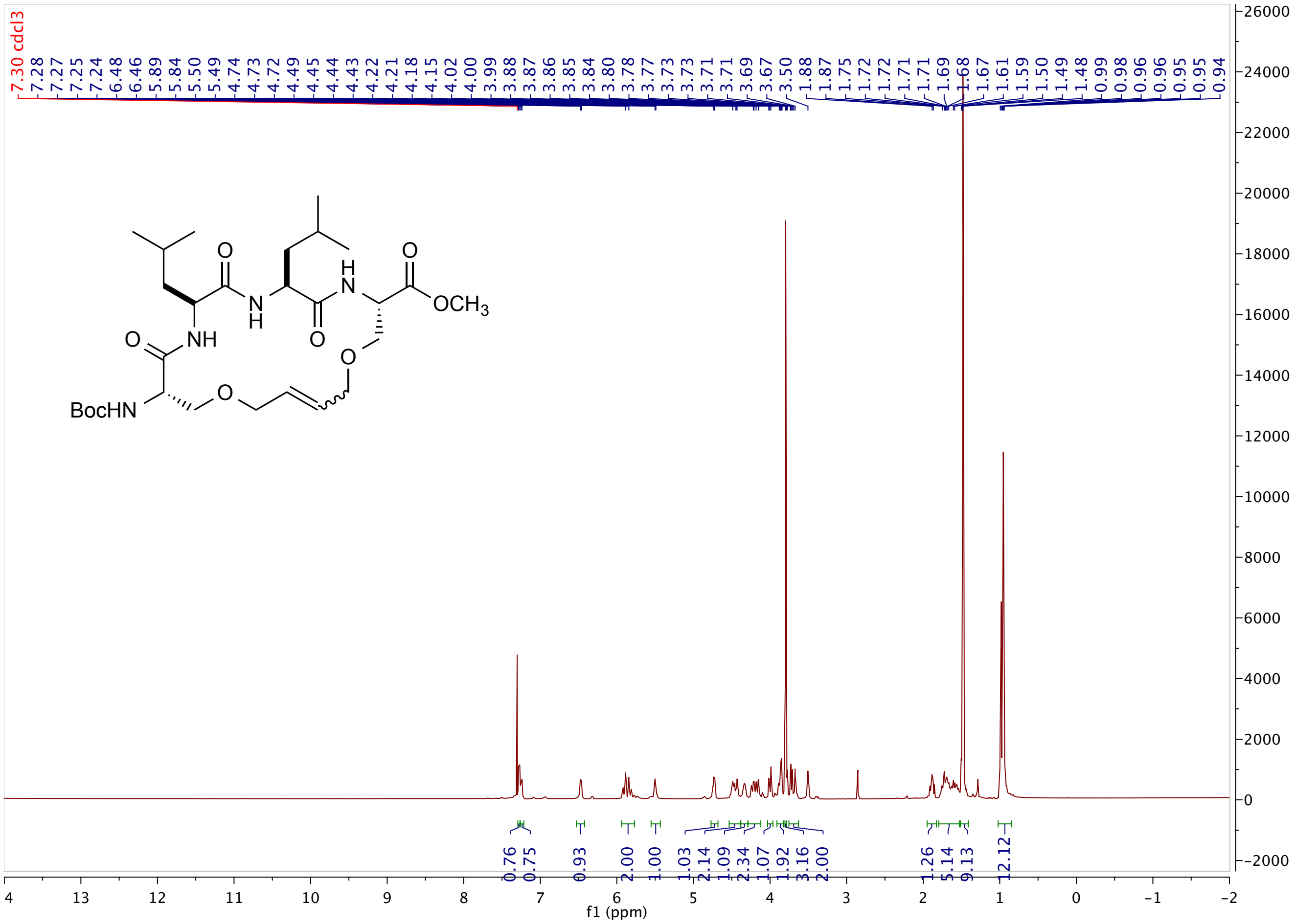

^{13}C NMR (126 MHz, $CDCl_3$) spectrum of compound **16**

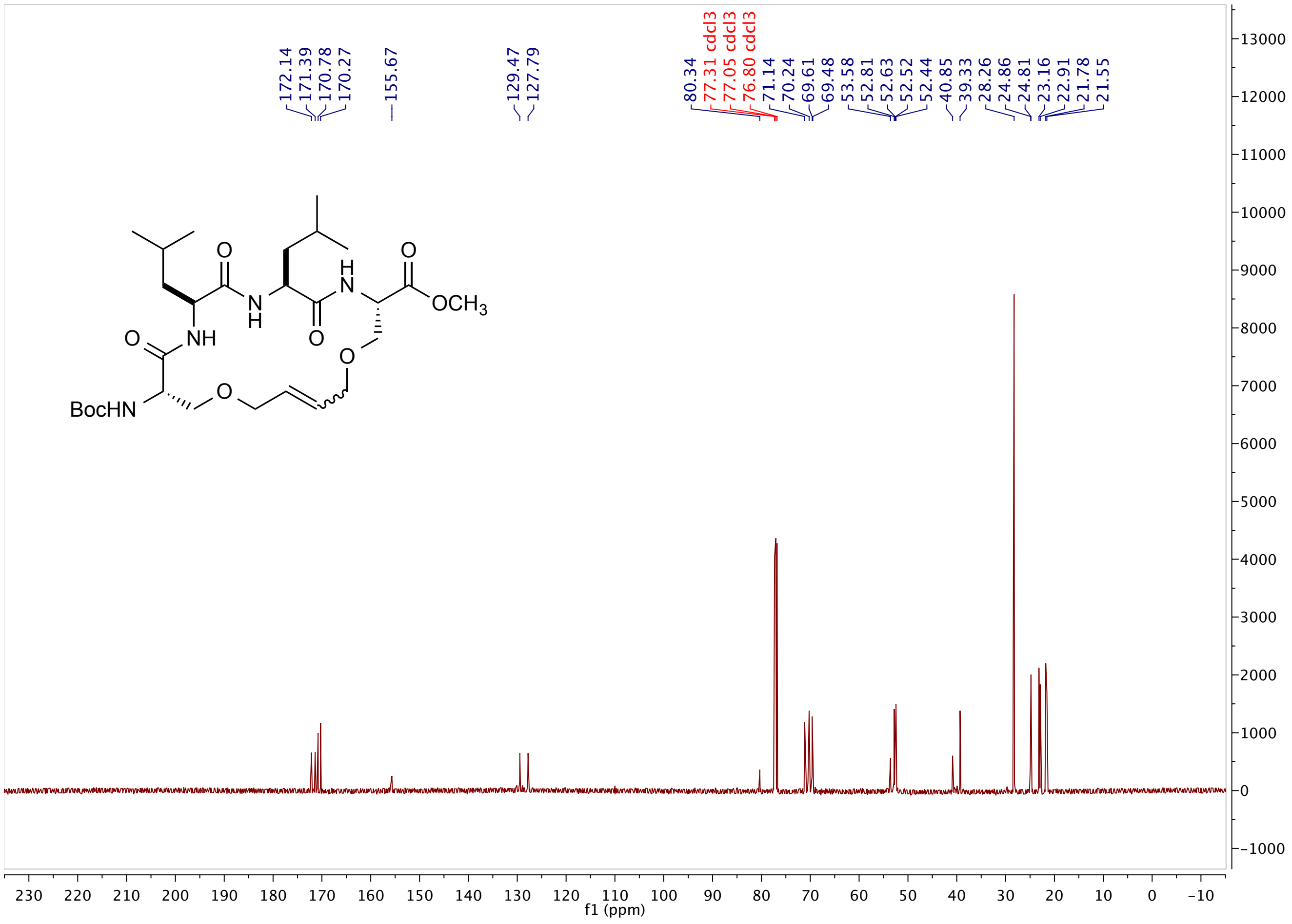

^{1}H NMR (500 MHz, $CDCl_3$) spectrum of compound **17**

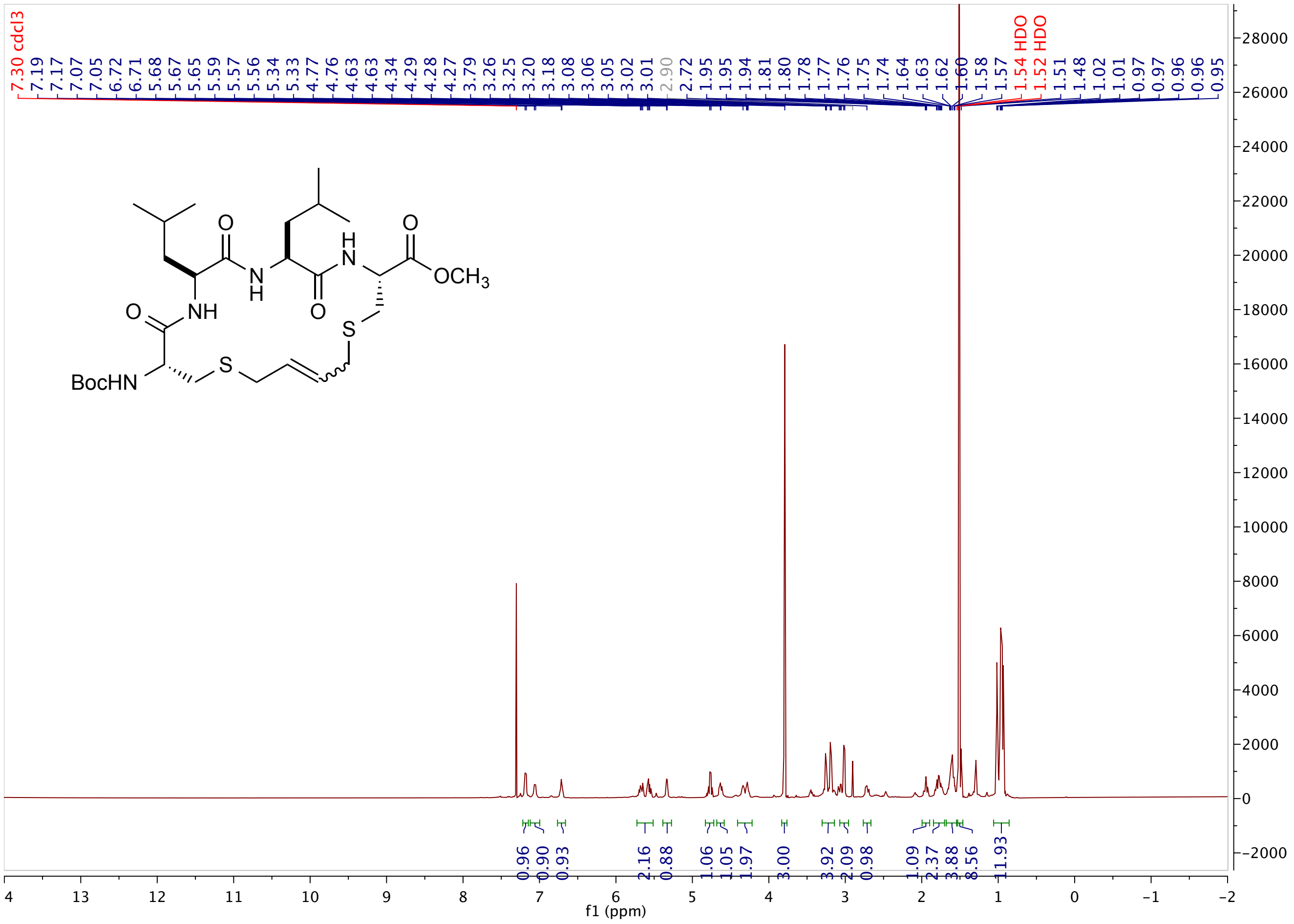

^{13}C NMR (126 MHz, $CDCl_3$) spectrum of compound **17**

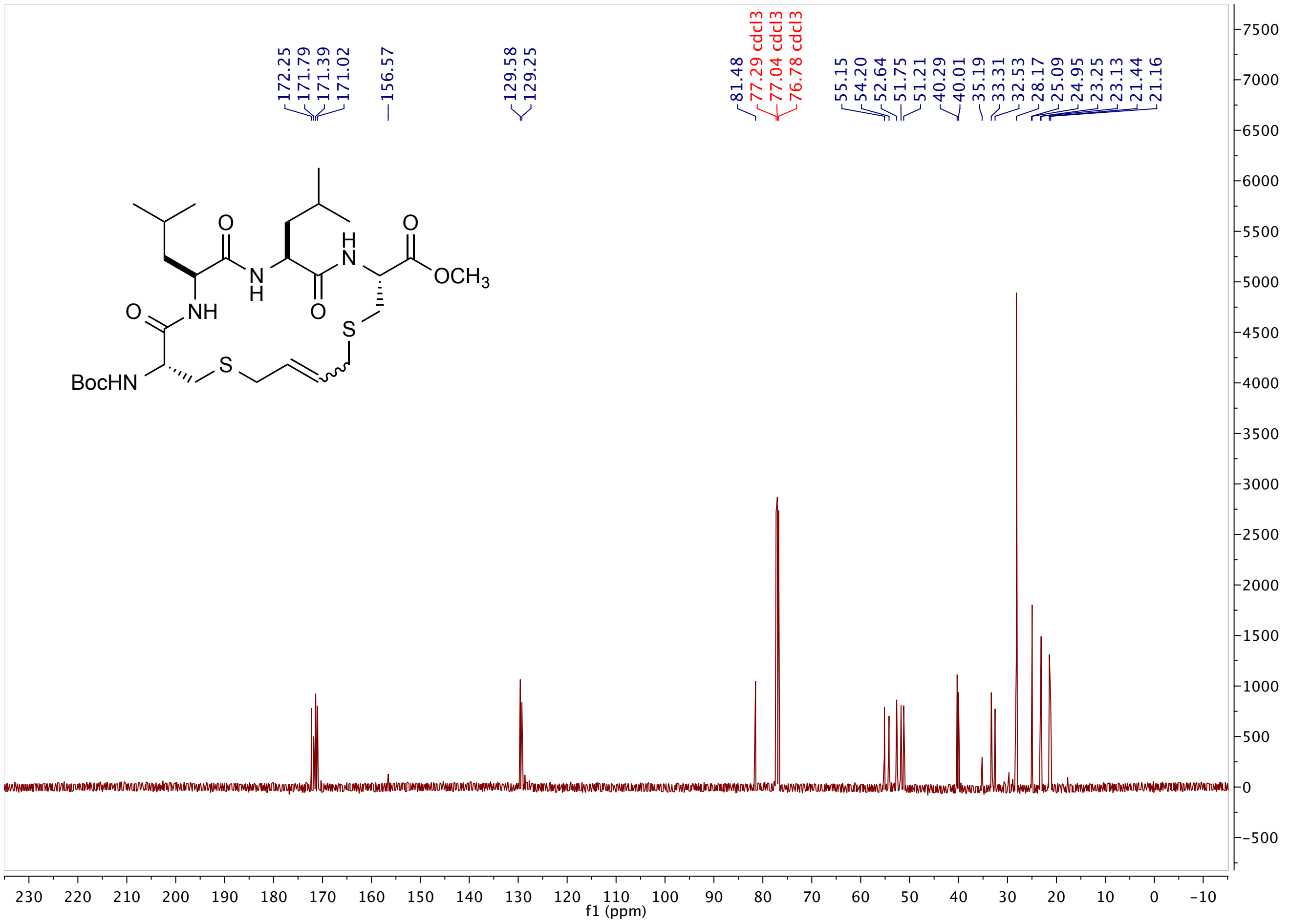

^{1}H NMR (500 MHz, $CDCl_3$) spectrum of compound **18**

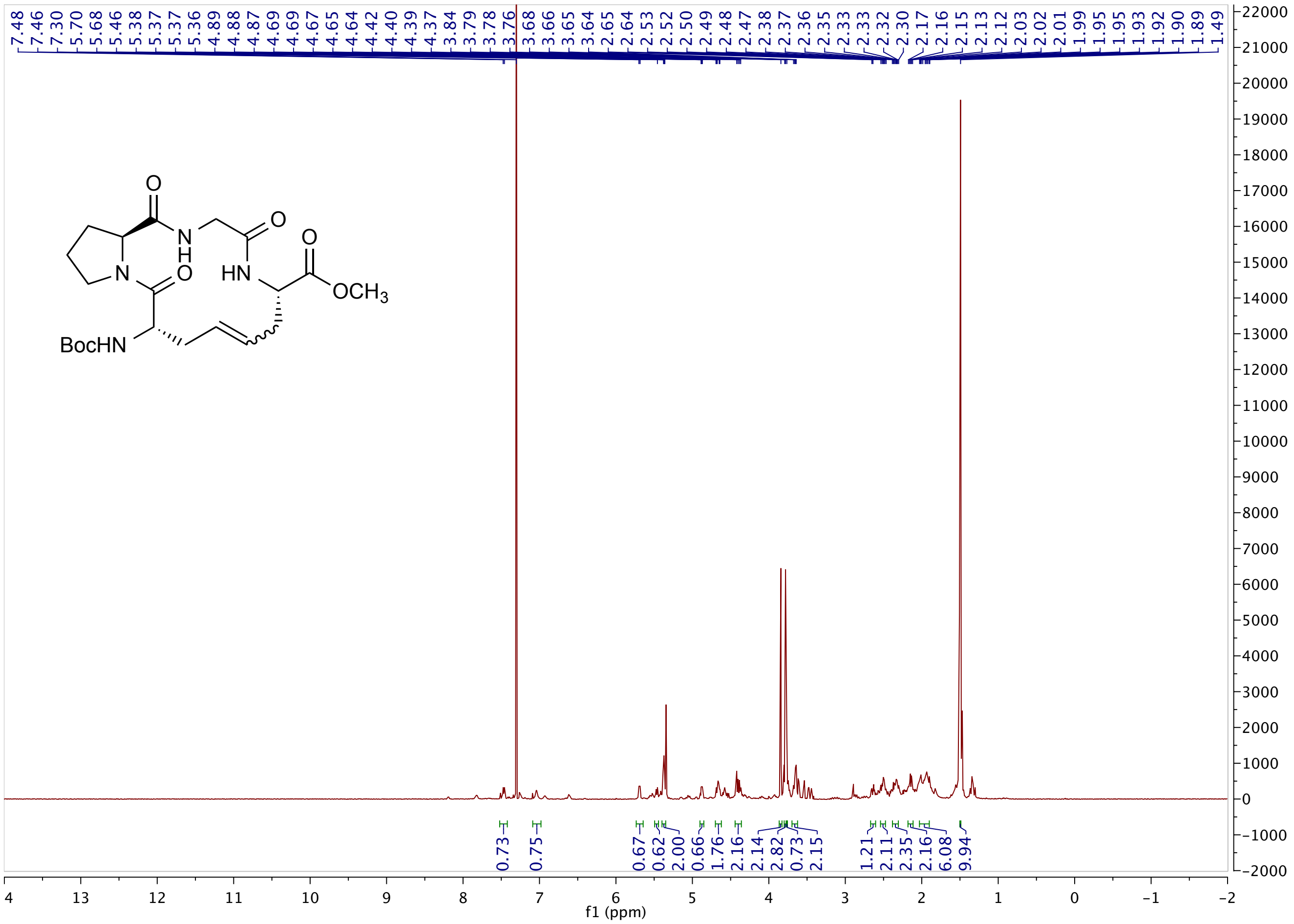

^{13}C NMR (126 MHz, $CDCl_3$) spectrum of compound **18**

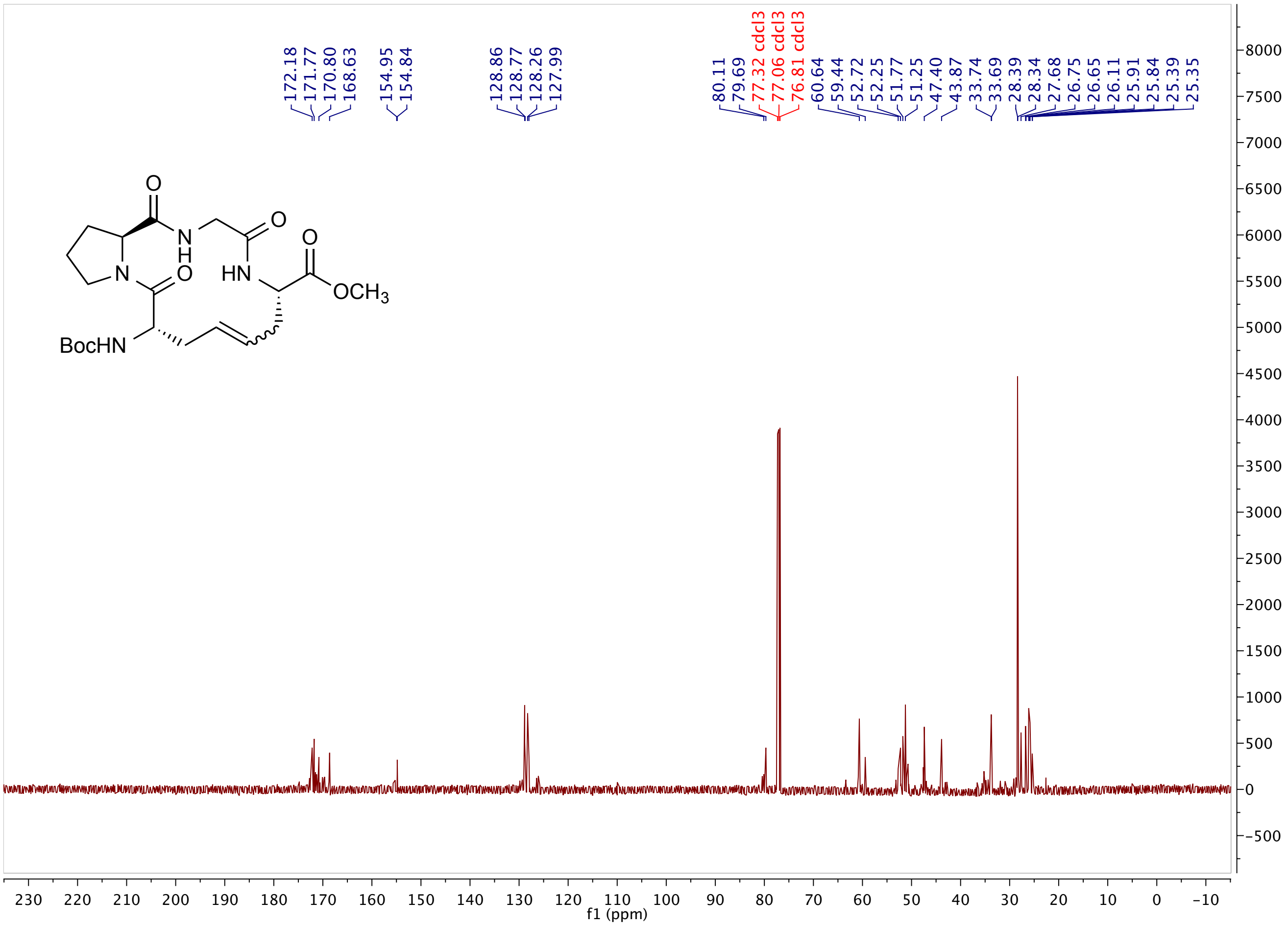

Supplement: Supplementary file 1 [file SC-006-C5SC01507C-s001.pdf]
